# Differential Evolution of the Epidermal Keratin Cytoskeleton in Terrestrial and Aquatic Mammals

**DOI:** 10.1093/molbev/msy214

**Published:** 2018-12-04

**Authors:** Florian Ehrlich, Heinz Fischer, Lutz Langbein, Silke Praetzel-Wunder, Bettina Ebner, Katarzyna Figlak, Anton Weissenbacher, Wolfgang Sipos, Erwin Tschachler, Leopold Eckhart

**Affiliations:** 1 Research Division of Biology and Pathobiology of the Skin, Department of Dermatology, Medical University of Vienna, Vienna, Austria; 2 Department of Genetics of Skin Carcinogenesis, German Cancer Research Center, Heidelberg, Germany; 3 Vienna Zoo, Vienna, Austria; 4 Clinical Department for Farm Animals and Herd Management, University of Veterinary Medicine Vienna, Vienna, Austria; 5 Division of Cell and Developmental Biology, Center for Anatomy and Cell Biology, Medical University of Vienna, Vienna, Austria; 6 Centre for Cell Biology and Cutaneous Research, Blizard Institute, Queen Mary University of London, London, United Kingdom

**Keywords:** keratin, skin, evolutionary innovation, phenotypic plasticity, gene family

## Abstract

Keratins are the main intermediate filament proteins of epithelial cells. In keratinocytes of the mammalian epidermis they form a cytoskeleton that resists mechanical stress and thereby are essential for the function of the skin as a barrier against the environment. Here, we performed a comparative genomics study of epidermal keratin genes in terrestrial and fully aquatic mammals to determine adaptations of the epidermal keratin cytoskeleton to different environments. We show that keratins K5 and K14 of the innermost (basal), proliferation-competent layer of the epidermis are conserved in all mammals investigated. In contrast, K1 and K10, which form the main part of the cytoskeleton in the outer (suprabasal) layers of the epidermis of terrestrial mammals, have been lost in whales and dolphins (cetaceans) and in the manatee. Whereas in terrestrial mammalian epidermis K6 and K17 are expressed only upon stress-induced epidermal thickening, high levels of K6 and K17 are consistently present in dolphin skin, indicating constitutive expression and substitution of K1 and K10. K2 and K9, which are expressed in a body site-restricted manner in human and mouse suprabasal epidermis, have been lost not only in cetaceans and manatee but also in some terrestrial mammals. The evolution of alternative splicing of K10 and differentiation-dependent upregulation of K23 have increased the complexity of keratin expression in the epidermis of terrestrial mammals. Taken together, these results reveal evolutionary diversification of the epidermal cytoskeleton in mammals and suggest a complete replacement of the quantitatively predominant epidermal proteins of terrestrial mammals by originally stress-inducible keratins in cetaceans.

## Introduction

The epidermis of the skin represents the main interface of the body and the environment. In mammals, the epidermis is a stratified epithelium formed by keratinocytes (Fuchs and Raghavan 2002; Simpson et al. 2011) in association with quantitatively minor cell types involved in pigmentation (melanocytes), immune defense (Langerhans cells, T-cells), and mechanosensation (Merkel cells). Keratinocytes proliferate exclusively in the basal layer of the epidermis and differentiate into barrier cells in the suprabasal layers, which results in significant changes in their gene expression profiles (Simpson et al. 2011; Joost et al. 2016). Upon the transition from the basal to the suprabasal layers, the cytoskeleton is remodeled and significantly enforced until eventually keratin intermediate filaments form the main portion of the cellular proteome providing high mechanical resilience to the epidermis (Feng et al. 2013; Homberg and Magin 2014; Jacob et al. 2018). Further essential skin barrier components are keratin-linked desmosomal junctions and tight junctions between the cells, proteins cross-linked by transglutamination and protein-bound ceramides at the cell surface and lipids secreted into the intercellular space of the outermost epidermal layers (Proksch et al. 2008). Although it is commonly accepted that adaptations of the epidermis facilitated the adaptation of vertebrates to new environments and lifestyles (Bereiter-Hahn et al. 1986; Alibardi 2003; Wagner 2014; Strasser et al. 2015), the molecular changes of the epidermis during the evolution of mammals have remained incompletely understood (Foote et al. 2015).

Keratins (abbreviated K for proteins and *KRT* for genes) are the main components of the intermediate filament cytoskeleton of epithelial cells. The human genome contains 54 keratin genes which are arranged in two clusters: the cluster of 27 type I keratins on chromosome 17q21 and the cluster of 26 type II keratins together with the type I keratin *KRT18* on chromosome 12q13 (Schweizer et al. 2006). Pairs of one type I and one type II keratin are co-expressed in different epithelial cell types and differentiation stages, allowing heterodimerization and subsequent formation of cell type- and differentiation-specific supramolecular filaments (Moll et al. 2008; Homberg and Magin 2014; Loschke et al. 2015). The great diversity of keratins mainly reflects the diversity of epithelial cell types, including simple epithelia (expressing K8 and K18), oral epithelium (K4 and K13), corneal epithelium (K3 and K12), and multiple epithelial cell layers of hair follicles (e.g., K32 and K82 in the hair cuticle; Bragulla and Homberger 2009). In the interfollicular epidermis, K5 and K14 dimerize in the basal layer, whereas K1 and K10 form dimers in the suprabasal layers (Fuchs and Cleveland 1998; Jacob et al. 2018). Besides these classical epidermal keratins, K2 is expressed in the outermost living (granular) layers of the epidermis in humans (supplementary fig. S1, Supplementary Material online) and in a body site-restricted, that is, ear, sole, and tail-specific, manner in the suprabasal epidermal layers of the mouse (Fischer et al. 2014), and K9 is expressed suprabasally on palms and soles only (Moll et al. 1987; Fischer et al. 2014). The type II keratin K77 is expressed in the suprabasal epidermis of embryos and to a lesser extent in postnatal skin (Langbein et al. 2013), K78 is expressed in basal and low suprabasal keratinocytes (Langbein et al. 2016), and K80 is present in the granular layer of adult epidermis (Langbein et al. 2010). In situations of wound healing and in hyperproliferative conditions of the epidermis, such as in psoriatic lesions, K1, K2, and K10 are substituted by K6, K16, and K17 (Leigh et al. 1995; McGowan and Coulombe 1998; Freedberg et al. 2001; Mazzalupo et al. 2003). Mutations of human keratin genes result in epidermal defects and skin diseases, ranging from hereditary bullous diseases to ichthyoses (Lane and McLean 2004; Toivola et al. 2015).

Here, we tested the hypothesis that alterations in the keratinocyte differentiation program during the evolutionary transition from terrestrial to fully aquatic life of cetaceans and sirenians was associated with gain or loss of suprabasal keratin genes.

## Results

### Comparative Genomics Suggests Loss of Suprabasal Epidermal Keratins in Cetaceans and Sirenians

First, we investigated which epidermal keratins were conserved or lost in fully aquatic mammals in comparison to closely related terrestrial mammals. We performed a comparative genomics study focused on the type I and type II gene loci using publically available genome sequences of cetaceans (minke whale, sperm whale, baiji, bottlenose dolphin, and orca), a sirenean (manatee), and terrestrial mammals (human, cattle, elephant). Gene annotations available in GenBank were scrutinized and improved by integrating additional data from whole genome shotgun sequences where necessary (supplementary tables S1–S6, supplementary fig. S2, Supplementary Material online). Several keratin gene segments of the manatee were amplified from genomic DNA and sequenced to confirm conclusions (supplementary fig. S3, Supplementary Material online).

**Fig. 1.**
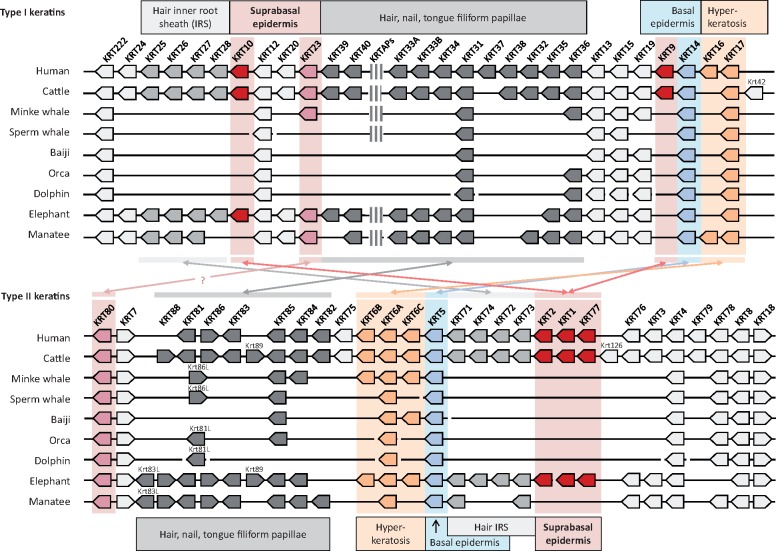
Comparative analysis of the keratin type I and type II gene clusters in fully aquatic and terrestrial mammals. Comparison of the type I and II keratin gene clusters of human, cattle, dolphin, orca, baiji, sperm whale, minke whale, manatee, and elephant indicate a loss of several keratin genes in fully aquatic mammals, including epidermal differentiation-associated type 1 keratins K10 and K9 and type 2 keratins K1, K2, and K77 (red arrows). The direction of arrows indicates the orientation of gene transcription. Only genes considered to be intact (encoding a functional protein) are shown. The position of a cluster of genes encoding keratin-associated proteins (KAPs) is indicated within the type I keratin gene cluster. Heterodimerization interactions between keratins of particular interest for the present study are indicated by double-headed arrows and the main expression sites are shown above and below the depiction of the gene clusters. Note that K222 has a unique structure and the orthology relationships of the K6 isoforms and of K81 and K86-like proteins are uncertain in cetaceans.

The number of keratin genes is strongly reduced in cetaceans due to the loss of cysteine-rich keratins implicated in the growth of claws and hairs. Claws are absent in cetaceans and hairs are reduced to modified vibrissae in some cetaceans (Berta et al. 2015; Drake et al. 2015) and entirely absent in others (Sokolov 1982). However, at least one pair of type I and type II hair keratins was conserved in all cetaceans, indicating possible roles in filiform papillae of the tongue or in rudimentary hair. The manatee has lost the pelage but retained hairs of sensory function, and most of the hair keratin genes have homologs in this species (fig. 1). Importantly, the classical suprabasal epidermal keratins K1, K2, K9, K10, and K77 are absent in all cetaceans investigated as well as in the manatee (fig. 1), suggesting that they are dispensable for the formation and maintenance of the epidermis in mammals that permanently reside in water.

In contrast to the suprabasal keratins, the basal epidermal keratins of terrestrial mammals, K5 and K14, are conserved in all aquatic species investigated (fig. 1). Likewise K78, a quantitatively minor keratin of the basal and low suprabasal epidermis (Langbein et al. 2016), and keratins K6 and K17 that are expressed in situations of keratinocyte hyperproliferation during wound healing or in hyperkeratotic skin diseases such as psoriasis (Leigh et al. 1995), are conserved in cetaceans and manatee.

**Fig. 2.**
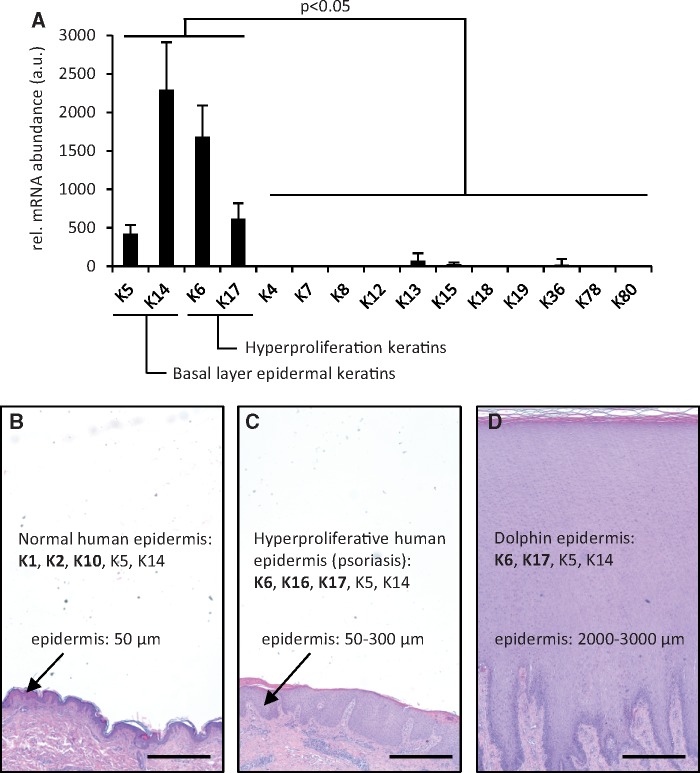
Analysis of transcriptomes of dolphin skin shows high abundance of hyperproliferation-associated keratins K6 and K17. (*A*) Published transcriptome data of bottlenose dolphin skin samples (*n* = 116; Neely et al. 2018) were analyzed for the expression levels of keratin genes. The number of RNA-seq reads were normalized to the expression level of *ALAS1* as a reference gene. a.u., arbitrary units. (*B*–*D*) Schematic comparison of epidermal thickness and keratin expression in normal (*B*) and psoriatic (*C*) human epidermis and cetacean (*D*) epidermis. Skin sections stained with hematoxylin and eosin are shown at equal magnifications to facilitate comparison of epidermal thickness. Keratins expressed in human (according to literature cited in the main text) and dolphin skin (this study) are indicated. Keratins K6 and K17 are induced when keratinocyte hyperproliferation during wound healing or in psoriasis leads to thickening of the epidermis. Scale bars: 500 μm.

To identify keratins that may substitute for the loss of classical suprabasal keratins in cetaceans, we analyzed the available transcriptomes of skin samples collected from dolphins (Neely et al. 2018). Keratin mRNA abundance was determined by the number of RNA-seq reads of the respective keratin relative to the number of reads corresponding to the reference gene *Alas1* (aminolevulinate synthase 1). These data showed that K5, K6, K14, and K17 were the predominant mRNAs in dolphin skin (fig. 2*A*), suggesting that besides the basal layer keratin pair K5/K14, K6 and K17 are coexpressed to allow for filament formation in the absence of K1/K10 and K2/K10 dimers. K6 and K17 are evolutionarily conserved keratins that are expressed under conditions of keratinocyte hyperproliferation, such as skin wound closure and psoriasis (Navarro et al. 1995; Mazzalupo et al. 2003), which are regularly associated with epidermal thickening (fig. 2*B*). Strikingly, the epidermis of cetaceans is characterized by deep papillae indicative of high cell proliferation in the basal layer and extreme thickening as compared with all terrestrial mammals (Hicks et al. 1985; fig. 2*B*).

### *Krt23* Is Absent in Most Cetaceans and Expressed in the Human Suprabasal Epidermis

Besides strictly epidermal keratins (K1, K2, K9, K10, K77) and hair follicle-associated keratins (see fig. 1, and K79; Langbein and Schweizer 2005; Veniaminova et al. 2013), K3, K23, K24, and K76 have been lost in all or some cetaceans (fig. 1). Screening of the published transcriptomes of differentiated epidermal keratinocytes (Toulza et al. 2007; Taylor et al. 2009) showed that among the latter keratins, only K23 was abundantly present. We therefore investigated K23 further to determine its expression pattern in human epidermis, representing terrestrial keratinization, and its evolutionary fate in cetaceans.

**Fig. 3.**
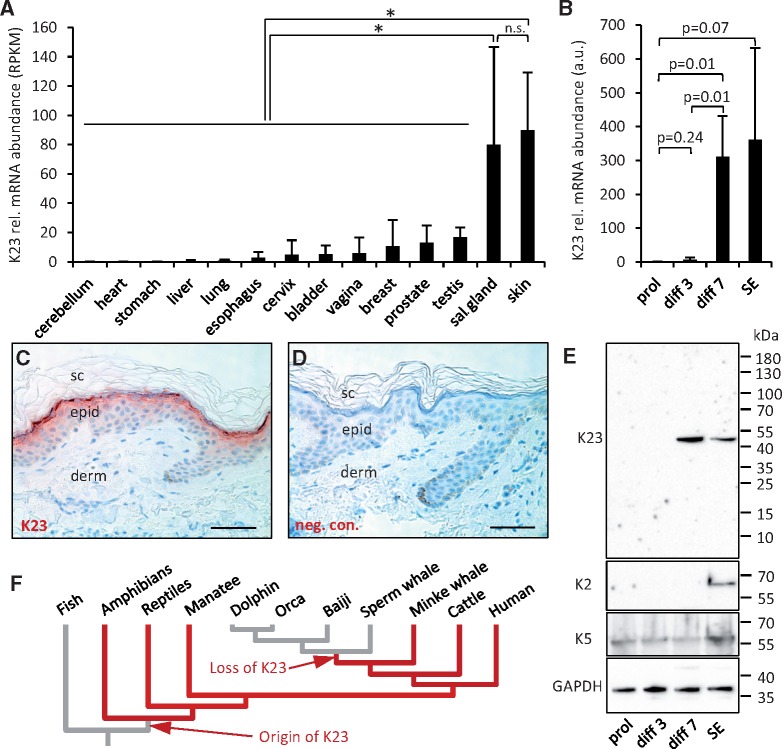
Keratin K23 is expressed in differentiated epidermal keratinocytes. (*A*) Expression levels of *KRT23* in human tissues were obtained from the GTEx database. Bars indicate means and error bars indicate standard deviations. *, *P* < 0.05 (*t*-test); n.s., not significant; sal., salivary; RPKM, reads per kilobase of transcript per million mapped reads. (*B*) Expression of *KRT23* during differentiation of primary human keratinocytes in vitro. Human epidermal keratinocytes were cultured in monolayer culture under subconfluent proliferation (prol)-enhancing conditions, under postconfluent differentiation (diff)-enhancing conditions for 3 and 7 days, and in skin equivalent (SE) cultures. *KRT23* expression was determined by quantitative real-time PCR using *ALAS1* as a reference gene for normalization. (*C*) Immunohistochemical analysis of K23 (red) demonstrated expression in the most differentiated living layers of human epidermis. (*D*) Replacing the primary antibody by immunoglobulin from nonimmunized guinea pigs abolished the staining and confirmed its specificity. sc, stratum corneum, derm, dermis, epid, epidermis. Scale bars: 50 µm. (*E*) Western blot analysis of K23 in human keratinocytes differentiating in vitro. Protein lysates from keratinocytes were consecutively analyzed with primary antibodies against K23, K2, K5, and GAPDH. The positions of molecular weight markers are shown on the right. kDa, kilo-Dalton. (*F*) Schematic summary of K23 evolution in vertebrates, inferred from the presence or absence of *Krt23* genes in modern species mapped onto a phylogenetic tree (Zhou et al. 2011).

Screening of a human gene expression database (GTEx Consortium 2015) showed that *KRT23* is predominantly expressed in skin and salivary glands and at lower levels in other tissues (fig. 3*A*). A similar expression pattern was found in the mouse (supplementary fig. S4*A* and *B*, Supplementary Material online). When epidermal keratinocytes were cultured in vitro and induced to differentiate according to a published protocol (Fischer et al. 2007), expression of *KRT23* increased with progressive differentiation (fig. 3*B*). Using a newly raised antibody against human K23, we detected K23 in differentiated keratinocytes of the human epidermis with highest abundance in the granular layer (fig. 3*C* and *D*). K23 was also detected in ductal cells of sebaceous glands and, at lower levels, in ducts of salivary glands, whereas negative control stainings did not yield signals (supplementary fig. S4*C*–*H*, Supplementary Material online). Western blot analysis demonstrated expression of K23 in differentiated keratinocytes in skin equivalents and, in contrast to K2, also in late stages of confluent monolayer culture of keratinocytes (fig. 3*E*). Together, these data establish K23 as a keratinocyte differentiation-associated keratin in human skin.

K23 is absent in Mysticeti (toothed whales and dolphins) but conserved in the minke whale and the manatee (fig. 1, supplementary fig. S4*I*, Supplementary Material online). BLAST searches in the unassembled whole genome shotgun sequence of the grey whale (Moskalev et al. 2017) and in the genome sequence reads of the humpback whale and the blue whale (Árnason et al. 2018) showed that K23 is generally conserved in baleen whales whereas the classical epidermal keratins K1, K2, and K10 are lost (supplementary fig. S5, Supplementary Material online). K80 is conserved in baleen whales but, in contrast to K23, also in Mysticeti (fig. 1). These differences in evolutionary fates suggest that keratin heterodimerization partners have changed during evolution.

### Alternatively Spliced *Krt10* mRNAs Encode Two Proteins with Different Carboxy-terminal Sequences in Terrestrial Mammals

**Fig. 4.**
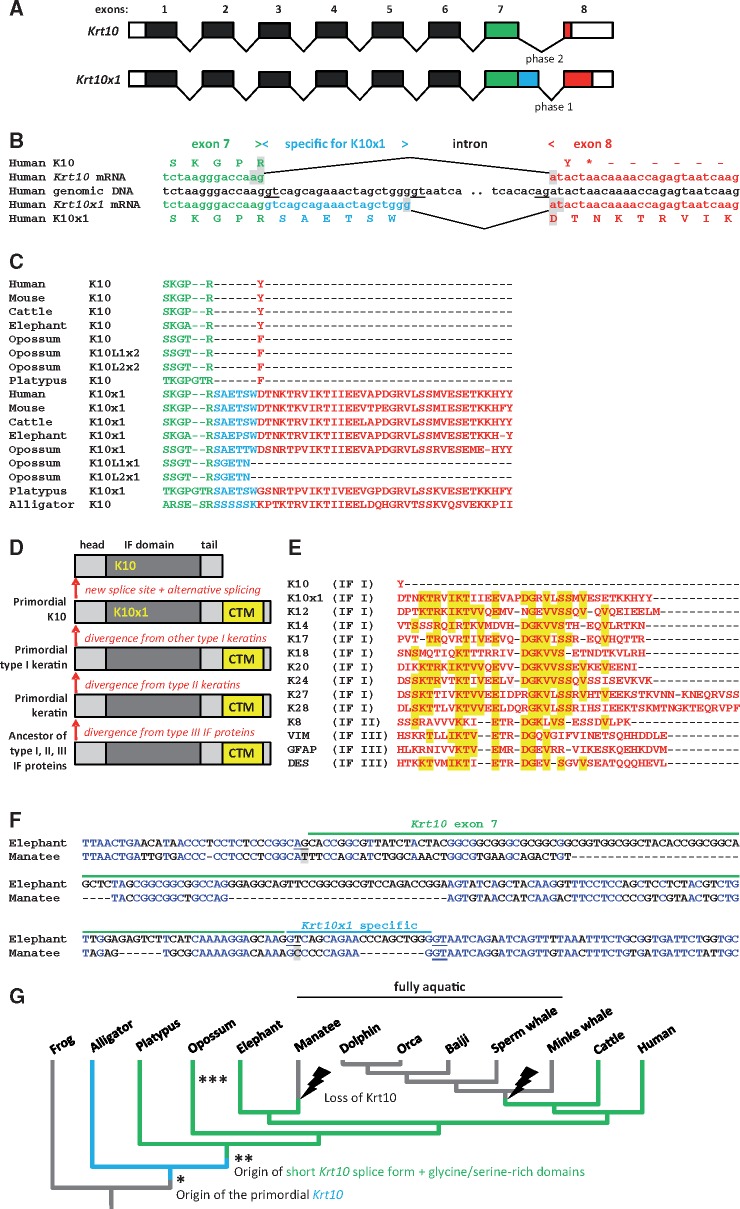
Two isoforms of K10 are generated by alternative splicing in terrestrial mammals. (*A*) Schematic depiction of the exon–intron organization of *Krt10* and splicing leading to K10 and K10x1 isoforms. (*B*) Alignment of nucleotide sequences around the two alternative splice donor sites of the *Krt10* gene and the two mRNA variants. Amino acid sequences encoded by the mRNAs are shown. *, end of protein. (*C*) Alignment of carboxy-terminal amino acid sequences of K10 variants from different species. (*D*) Schematic model of the evolution of K10. IF, intermediate filament domain, CTM, carboxy-terminal motif. (*E*) Alignment of carboxy-terminal amino acid sequences of human intermediate filament proteins. Residues belonging to the evolutionarily ancestral CTM are indicated by yellow shading. Note that the amino acid sequences begin with residue 579 of human K10 and K10x1 in panels *B* and *C*, and with residue 584 of human K10 and residue 590 of human K10x1 in panel *E*. (*F*) Nucleotide sequence alignment of *Krt10* exon 7 (indicated by green and blue lines) and adjacent introns of manatee and elephant. GT/AG splice site motifs are underlined. Substitutions of splice sites are indicated by grey shading. Also note sequence deletions (indicated by dashes) in the manatee. (*G*) The evolution of the *Krt10* gene is indicated on a phylogenetic tree (Zhou et al. 2011). Asterisks indicate the origin of new traits and bolt symbols indicate the loss of *Krt10*. ***, triplication of *Krt10* in the opossum.

During the screening of gene expression databases for mRNAs of suprabasal epidermal keratins, we noticed that two mRNA variants of human *KRT10* exist (supplementary fig. S6, Supplementary Material online). The variants differ by the alternative utilization of two splice donor sites at the 3′-end of exon 7 (fig. 4*A* and *B*). The mRNA encoding the conventional K10 protein, that is, the most abundant protein of the epidermis (Feng et al. 2013), is derived by splicing from the proximal splice donor site and the second, much rarer variant, denoted K10x1 in GenBank, is derived by splicing from the distal splice donor site of exon 7. The *KRT10* mRNA is spliced in phase 2 (between the second and third nucleotide of a coding triplet) whereas *KRT10x1* is spliced in phase 1 (between the first and second nucleotide of a coding triplet), resulting in the use of different reading frames in exon 8. Only one amino acid residue is encoded by exon 8 in the *KRT10* mRNA and 35 residues are encoded by exon 8 in the *KRT10x1* mRNA (fig. 4*B* and *C*). Of note, the last two exons of the keratin genes most closely related to *KRT10*, that is, *KRT12* and *KRT24*–*KRT28* (Vandebergh and Bossuyt 2012; Vandebergh et al. 2013), are spliced in phase 1, like *KRT10x1*.

Comparison of *Krt10* genes across vertebrates showed that the two splice sites at the end of exon 7 and the amino acid sequences encoded by the alternative reading frames of exon 8 were conserved in terrestrial mammalian species (fig. 4*C*). Interestingly, the genome of the oppossum contains one *Krt10* ortholog in which both splice sites are conserved and two further gene copies in which the distal splice donor site of exon 7 is absent (fig. 4*C*;supplementary fig. S7, Supplementary Material online). In contrast, in the putative homologs of *Krt10* of the reptiles, only the distal splice site was present, and the amino acid sequence encoded by exon 8 was similar to that of mammalian K10x1 (fig. 4*C*, supplementary fig. S8, Supplementary Material online).

The carboxy-terminus of K10x1 proteins, but not of K10 proteins, contains the sequence motif KTR*IKT(I/V)**E***DG*V(L/V)SS*V (where asterisks indicate variable single amino acid residues or gaps; fig. 4*D* and *E*) that is largely conserved in intermediate filament proteins and likely has originated in the ancestral gene from which type I, II, III intermediate filament genes evolved (Eckhart et al. 2009; Eckhart and Ehrlich 2018). This implies that the carboxy-terminus of K10x1 corresponds to the ancestral trait and the evolutionary appearance of a new (proximal) splice site led to the origin of the truncated protein which became the quantitatively predominant form of K10 in mammals (fig. 4*D*).

Among the fully aquatic mammals investigated, all cetaceans lack the entire *Krt10* gene (fig. 1) whereas a *Krt10* ortholog could be identified in the manatee. The manatee nucleotide sequence corresponding to *Krt10* exon 7 deviates strongly from that of *Krt10* in the closely related elephant, and the splice sites at the 5′ and 3′ ends of exon 7 are inactivated (fig. 4*F*). In addition, the reading frame of exon 8 of manatee *Krt10* is disrupted, suggesting that the *Krt10* gene does not encode a functional full-length keratin. Taken together, these data suggest that the evolutionary history of K10 involved the emergence of a unique alternative splicing pattern in phylogenetically basal mammals, independent gene inactivation in two clades that have assumed a fully aquatic lifestyle, and triplication of the gene in the opossum (fig. 4*G*).

### Suprabasal Epidermal Keratins That Are Expressed in a Body Site-restricted Manner Have Been Lost in Diverse Terrestrial Mammals

**Fig. 5.**
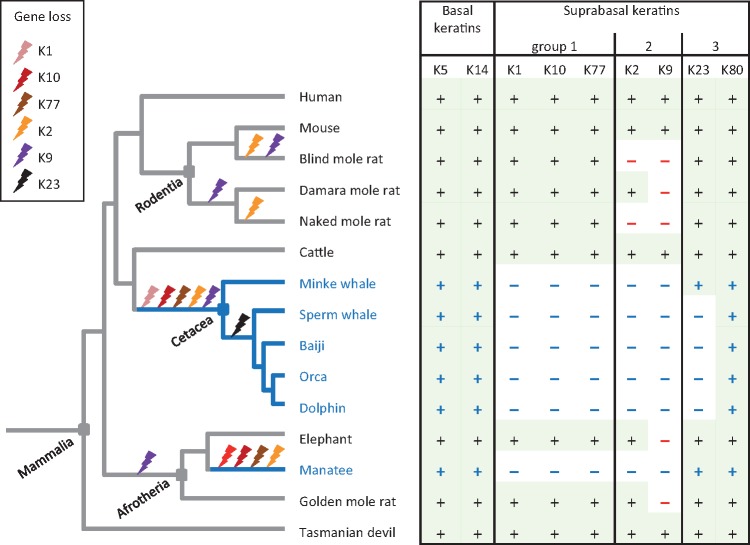
Schematic overview of epidermal keratin evolution. Phylogenetic relationships of mammalian species and the presence or absence of intact epidermal keratin genes (+ and – symbols in the table) are indicated. Gene inactivations that can be inferred from the species distribution of genes are indicated by bolt symbols on the phylogenetic tree (Zhou et al. 2011). The tasmanian devil is shown as a representative of marsupials. Blue lines and fonts indicate fully aquatic life.

To explore which keratin gene losses were associated with the land-to-water transition of mammals, we extended the comparative genomics analysis to phenotypically diverse terrestrial mammals. The type II keratins K1 (postnatal suprabasal epidermis) and K77 (embryonic suprabasal epidermis) and their type I binding partner, K10, were strictly conserved (figs. 1 and 5, supplementary table S7, Supplementary Material online, and data not shown). In contrast, suprabasal keratins that are not expressed continuously on the body surface, that is, K2 and K9, have been lost in at least two lineages of terrestrial mammals. In mice K2 is expressed exclusively on the external ears (pinnae), the tail and the palms (Fischer et al. 2014) and interestingly, this keratin has been lost in two rodent species, namely the blind mole rat and the naked mole rat (fig. 5) in which the pinnae have degenerated and the palms have been modified in the course of their adaptation to a fossorial lifestyle (supplementary fig. S9, Supplementary Material online). K9 is expressed in the epidermis on the palms of mice (Fischer et al. 2014; Fu et al. 2014; Fischer et al. 2016) and adjacent to sweat glands in human epidermis (Moll et al. 1987; Langbein et al. 2013). Our analysis of mammalian genome sequences showed absence of an intact *Krt9* gene in the elephant and, in line with a recent report (Partha et al. 2017), in three clades of phylogenetically diverse mole rats. The absence of K9 in the elephant and the manatee, which are representatives of the sister groups *Proboscidea* and *Sirenia*, respectively, indicates that the loss of K9 may have occurred prior to the land-to-water transition of sirenians (fig. 5).

In summary, comparative genomics of epidermal keratins showed strict conservation of basal epidermal keratins K5 and K14 and differential evolution of suprabasal keratins which accordingly can be classified in three groups (fig. 5). Group 1 suprabasal keratins (K1, K10, and K77) were lost in all fully aquatic but in no terrestrial mammals, group 2 suprabasal keratins (K2 and K9) were lost in all fully aquatic and in some terrestrial mammals, and group 3 suprabasal keratins (K23 and K80) were conserved at least in some fully aquatic mammals (fig. 5). These patterns suggest indispensable and optional roles of group 1 and 2 keratins, respectively, in terrestrial life of mammals and pleiotropic roles in the epidermis and other tissues of group 3 keratins.

## Discussion

Keratins are the essential components of the cytoskeleton that provides mechanical stability to the epidermis. The role as structural proteins and additional roles of keratins in epithelial homeostasis have been extensively studied in human patients carrying mutations in keratin genes as well as in mouse models, which replicate many but not all aspects of keratin biology relevant for human physiology (Feng et al. 2013; Fischer et al. 2014; Homberg and Magin 2014; Kumar et al. 2015; Lessard and Coulombe 2012; Jacob et al. 2018). Together with comparative studies of keratins in phylogenetically diverse vertebrates (Zimek and Weber 2005; Vandebergh and Bossuyt 2012; Vandebergh et al. 2013; Greenwold et al. 2014; Wu et al. 2015), these studies have led to the characterization of most keratins, to the identification of interspecies differences with regard to individual keratins such as K42 (Hesse et al. 2004) and to a basic understanding of the evolutionary history of keratin intermediate filaments in general and particularly of subgroups, such as hair keratins (Peter and Stick 2015; Eckhart et al. 2008; Eckhart and Ehrlich 2018). The results of the present study represent an extension of these lines of research by providing important new data on the evolution of keratins that control the mechanical stability of the outermost cell layers of the epidermis.

The results of this study extend previous studies on the evolution of keratin genes in cetaceans (Nery et al. 2014; Sun et al. 2017). These reports have suggested primarily a loss of hair keratin genes and refrained from drawing conclusions on epidermal keratins. The availability of updated genome sequences and the inclusion and careful evaluation of sequence contigs not present in the assembled genome sequences has allowed us to improve gene predictions. By investigating the entire keratin gene complement of five cetacean species (fig. 1) and possible epidermal keratins in additional species (supplementary fig. S5, Supplementary Material online), we have addressed problems with sequence gaps and possible annotation errors in draft assemblies of individual species of cetaceans, and the conclusions are based on genome sequence features consistently observed in more than one species. The finding that the cluster of genes encoding suprabasal epidermal type II keratins (*Krt1*, *Krt2*, *Krt77*) as well as the type I keratin *Krt10* are absent in all cetaceans clearly shows that these genes are dispensable in the aquatic environment. Independent loss of the same genes in the manatee supports this notion. On the basis of skin transcriptome data that are available for dolphins (fig. 2), we propose that the classical suprabasal keratins have been replaced by K6 and K17 in cetaceans. This evolutionary switch in suprabasal keratins resembles the inducible change of the keratin expression in terrestrial mammalian skin during the epidermal regeneration response to wounding or during pathological epidermal hyperproliferation in psoriasis (Leigh et al. 1995; Navarro et al. 1995; McGowan and Coulombe 1998; Mazzalupo et al. 2003; Morhenn et al. 2013). The change from K1/K10 to K6/K17 is accompanied by thickening of the epidermis in terrestrial mammals and the thickness of the epidermis has also strongly increased in cetaceans (Sokolov 1982; Hicks et al. 1985; fig. 2*D*). Thus, we put forward the hypothesis that 1) the phenotypic plasticity of the ancestral mammalian epidermis allowed two alternative keratinocyte differentiation programs characterized by expression of K1/K10 and K6/K17, respectively, 2) the K6/K17-positive hyperproliferative epidermal phenotype was advantageous in fully aquatic life when the skin was permanently exposed to high mechanical shear stress, and 3) eventually the K1/K10-positive “normal terrestrial” epidermal phenotype became dispensable. This scenario of phenotypic plasticity at the origin of the cetacean epidermis differs from other examples of phenotypic plasticity that are linked to alternative development programs (Crispo 2007; Pfennig et al. 2010; Moczek 2015). We propose that a stress response program of the adult organism was utilized for evolutionary innovation. Further studies will determine whether other originally stress-related genes are constitutively expressed in the epidermis of cetaceans and sirenians.

In a previous study we detected the loss of filaggrin, a putative keratin filament-aggregating protein of the suprabasal epidermis (Steinert et al. 1981; Blessing et al. 1993), in the minke whale and sperm whale whereas an intact filaggrin gene was identified in dolphins (Strasser et al. 2015). The present study shows that dolphins have lost K1, K2, K77, and K10, strongly suggesting that these canonical suprabasal keratins are not exclusive, if at all, targets of filaggrin. In correlation with the massive change of epidermal morphology, additional changes in the gene set determining keratinocyte differentiation have occurred in cetaceans (Strasser et al. 2015; Oh et al. 2015; Abbas Zadeh et al. 2017; Hecker et al. 2017). Thus, the evolutionary changes in the genetic regulation of epidermis during the evolution of cetaceans represents a promising model system for identifying interdependencies of genes implicated in epithelial biology.

Although we focused on the comparison of fully terrestrial to fully aquatic mammals, we have also obtained data that suggest limited diversification of epidermal keratins among terrestrial mammals. *Krt9*, which is expressed adjacent to sweat glands in human skin (Moll et al. 1987; Langbein et al. 2013) and exclusively on footpads in other mammals (Fu et al. 2014), has been lost not only in cetaceans, but also in terrestrial mammals such as mole rats (Partha et al. 2017), the horse (Balmer et al. 2017), and the elephant (fig. 1). Whereas cetaceans have entirely lost sweat glands and footpads, the adaption of the aforementioned terrestrial mammals to distinct lifestyles may have involved changes in mechanical stress on plantar skin or alterations in the function of sweat glands, leading to or tolerating the inactivation of *Krt9*. The *Krt2* gene is triplicated in the horse (Balmer et al. 2017), whereas it was lost in the blind mole rat and the naked mole rat, events that may correlate with the loss of pinnae, the main sites of K2 expression, except of the soles, in the mouse (Fischer et al. 2014). Together with triplication of K10 in the opossum (fig. 4), duplication of K10 in the horse (Balmer et al. 2017), loss of K3 in rodents (Chaloin-Dufau et al. 1993; Lu et al. 2006), loss of K16 in cattle and elephant (fig. 1), and loss of K42 in primates (Tong and Coulombe 2004), these data indicate that the set of keratin genes has undergone lineage-specific changes in mammals, and it appears likely that the investigation of further mammalian species will uncover further examples of gene loss or amplification. However, the changes in the two keratin loci in cetaceans are by far more extensive than any keratin changes observed in other mammals so far. The number of type I keratins is reduced to approximately one third in cetaceans (7–9 type I and 8 type II keratins) as compared with terrestrial mammals (human: 28 type I and 26 type II keratins). All keratins implicated in the formation of the inner rooth sheath of hair and hair follicle-associated keratins K75 and K79 are lost, but at least one classical hair keratin of type I and type II keratins has been retained in cetaceans. Most likely the retention of these cysteine-rich keratins is associated with the expression of “hair keratins” outside of hair and nails, particularly in the epithelium of the tongue. Interestingly, sequences corresponding to K31, K36, and K86 were identified by peptide mass fingerprinting in baleen of Mysticeti whales (Solazzo et al. 2017), suggesting that “hair keratins” have been coopted to the formation of baleen. Possible phenotype–genotype correlations associated with the loss of K3, K20, K24, and K76 in cetaceans (fig. 1) will be interesting subjects of future studies.

Our demonstration of K23 expression in the granular layer of the human epidermis and the identification of an evolutionarily conserved splice variant of K10 show that the genetic regulation of the suprabasal epidermal cytoskeleton is more complex than previously assumed (Bragulla and Homberger 2009). The immunodetection of K23 in keratinocytes close to the skin surface is compatible with the previous detection of K23 mRNA in differentiated keratinocytes (Toulza et al. 2007; Taylor et al. 2009; Mattiuzzo et al. 2011) and our finding that K23 mRNA increases from minute levels in proliferating to high levels in differentiated keratinocytes. Proteome studies of the cornified layer of the epidermis and of proteins extracted from the skin surface by sweating have detected K23 (Rice et al. 2013; Sakabe et al. 2014; Yu et al. 2017) but, consistently with the known predominance of K1, K2, and K10, the K23 abundance was low. Together with previous reports on K23 expression in stress-treated pancreas and liver cells (Zhang et al. 2001; Guldiken et al. 2016), our data (fig. 3, supplementary fig. S4, Supplementary Material online) show that K23 is expressed predominantly, but not exclusively in the epidermis of the skin. Consistent with this notion, K23 appears to have retained a function in some aquatic mammals including baleen whales and the manatee. A role of K23 in stress-inducible processes of extracutaneous epithelia (Guldiken et al. 2016) may have led to the conservation of K23 despite degeneration of the epidermal differentiation program in these species. The expression pattern of K23 in human epidermis resembles that of K80 (Langbein et al. 2010) and therefore, K23 represents a possible binding partner of K80. However, conservation of K80 despite loss of K23 in Mysticeti (fig. 5) demonstrates that K80 does not strictly depend on K23.

The identification of the K10x1 splice variant and its evolutionary conservation is surprising. Because of its high abundance in the epidermis, K10 is one of the most-investigated keratins. K10 proteins of different sizes have been reported to arise from an indel polymorphism within exon 7 of *Krt10* alleles (Korge et al. 1992) and one of these alleles was even considered to represent a distinct keratin, then named K11 (Moll et al. 1982) but later removed from the list of keratins (Schweizer et al. 2006). To the best of our knowledge, alternative splicing has not been reported for K10. Our data suggest that the quantitatively minor K10x1 form corresponds in structure to the evolutionarily ancestral K10 whereas the K10 variant predominant in the suprabasal epidermis of mammals is the evolutionarily derived variant. Interestingly, the type I keratin K80 is also alternatively spliced in several mammals but in that case the minor protein variant lacks the evolutionary ancient carboxy-terminus (Langbein et al. 2010). It remains to be investigated whether K10 and K10x1 play different roles—possibly in different cells—with one role depending on the carboxy-terminal sequence present in K10x1 and the other role requiring the absence of the carboxy-terminal sequence motif. It is conceivable that the function of the short K10 variant appeared in mammals and an ancestral function of the long K10 variant (K10x1) remained advantageous so that selection maintained both variants. The amplification of the *Krt10* gene in the oppossum and the apparent loss of the ability to encode long variants (K10x1-likes) in 2 *Krt10* gene copies supports the hypothesis that expression of a K10x1 protein from one *Krt10* gene has removed the selective constraints on the K10x1-like splice site and K10x1-like open reading frame in the two other *Krt10* genes. Two copies of *Krt10* with conserved alternative splice sites are present in the horse (Balmer et al. 2017). Further studies will address the role of K10x1 in mammals and the possible subfunctionalization of K10 isoforms in the oppossum.

In conclusion, this study reveals a significant evolutionary plasticity in the keratin composition of the mammalian epidermis and the surprising dispensability of classical suprabasal epidermal keratins in fully aquatic mammals. The epidermal keratin cytoskeleton underwent limited evolutionary changes in terrestrial mammals whereas it was entirely remodeled in fully aquatic mammals.

## Materials and Methods

### Comparative Genomics, Sequence Alignments, and Phylogenetic Analysis

The genome sequences of bottlenose dolphin (*Tursiops truncatus*), orca (*Orcinus orca*), baiji (Yangtze river dolphin) (*Lipotes vexillifer*), sperm whale (*Physeter catodon*), minke whale (*Balaenoptera acutorostrata scammoni*), cattle (*Bos taurus*), elephant (*Loxodonta africana*), manatee (*Trichechus manatus latirostris*), and human (*Homo sapiens*) (Lindblad-Toh et al. 2011; Zhou et al. 2013; Yim et al. 2014; Foote et al. 2015; Warren et al. 2017) were investigated for the presence or absence and sequence integrity of type I and type II keratin genes. In addition, the genome sequences of other species were used for sequence comparisons. The sequences were retrieved from the GenBank database of the National Center for Biotechnology Information (NCBI), USA (http://www.ncbi.nlm.nih.gov/, last accessed September 24, 2018). In addition, Basic Local Alignment Search Tool (BLAST; Altschul et al. 1990) was used to search for gene segments in whole genome shutgun (WGS) sequences. Nucleotide and amino acid sequences were aligned using Multalin (http://multalin.toulouse.inra.fr/multalin/, last accessed September 24, 2018) with the alignment parameters DNA-5-0 for nucleotide sequence alignments and Blossum62-12-2 for amino acid sequence alignments (Corpet 1988). Additional alignments were made with MUSCLE (https://www.ebi.ac.uk/Tools/msa/muscle/, last accessed September 24, 2018; Chojnacki et al. 2017). Molecular phylogenetic of keratins was performed by Neighbor-Joining analysis (model: Poisson, bootstrap with 10,000 replicates) and maximum likelihood analysis (model: JTT, bootstrap with 100 replicates) on the Seaview platform (Gouy et al. 2010).

### Sequence Analysis of Genomic DNA

DNA from manatee (*Trichechus manatus latirostris*), kindly provided by Dr Ralph Kühn, Zoo Nürnberg, Germany, was investigated by PCRs using the following primers: Tm-K1-s (5′-tctctgtcatggccaggaaa-3′) and Tm-K1-a (5′-ccaccaggtcctgtatgttct-3′) for *Krt1* (GeneID: 105756172). Tm-K2-s (5′-ctgagagtcttcccacagca-3′) and Tm-K2-a (5′-accacctccaaagtagcctc-3′) for *Krt2* (GeneID: 101351286), Tm-K77-s (5′-tccctggtctttgcttcctt-3′) and Tm-K77-a (5′-aagaaggaccaaagctccca-3′) for *Krt77* (GeneID: 101357457). Genomic DNA of the naked mole rat (*Heterocephalus glaber*) was prepared according to a standard protocol (Eckhart et al. 2006) from tissue kindly provided by the Vienna Zoo. The following primer sequences were used to amplify part of the *Krt2* gene: Hg-K2-s (5′-tcctggagcagcagaatcaa-3′) and Hg-K2-a (5′-gccaaagccttttatcaccaca-3′). PCRs were performed according to a published protocol (Eckhart et al. 1999) involving annealing temperatures between 59 and 64 °C. PCR products were sequenced by Microsynth AG, 6961 Wolfurt, Austria and sequences were deposited in GenBank (Accession numbers MH517025–MH517029).

### In Vitro Culture of Human Keratinocytes and Skin Models

Keratinocytes and fibroblasts were isolated from fresh abdominal skin samples obtained from plastic surgery. All donors provided written informed consent and the use of tissues and cells was approved by the Ethics Committee at the Medical University of Vienna (EK2011/1149). The epidermis and dermis were separated by overnight incubation with 2.4 U/ml dispase (Roche Applied Science, Basel, Switzerland) at 4 °C. Keratinocytes were isolated by incubation with trypsin (Lonza, Basel, Switzerland) in the presence DNase1 for 8 min at 37 °C. Keratinocytes were grown in keratinocyte growth medium 2 (Lonza). Cells were maintained in monolayer cultures or used for the preparation of three-dimensional skin equivalent models according to published protocols (Rendl et al. 2002; Fischer et al. 2007). The epidermal compartment of skin equivalents was peeled off from the dermal compartment using forceps.

### Detection of mRNAs in Tissues and Cells

mRNA levels in human tissues and cell types were retrieved from the GTEx (https://www.gtexportal.org/, last accessed September 24, 2018; GTEx Consortium 2015) and the Genevestigator (https://genevestigator.com/, last accessed September 24, 2018; Hruz et al. 2008) databases. mRNA levels in the skin of dolphins were estimated by counting RNA-seq reads in the transcriptomes of 116 bottlenose dolphins (Neely et al. 2018). The numbers of reads were normalized to the expression level of *ALAS1* as a reference gene.

RNA was purified from cultured human keratinocytes, the epidermal part of skin equivalents, and from mouse tissues using the Precellys system (VWR International, Radnor, PA) and TriFast (VWR International) according to the manufacturers’ instructions. The iScript Kit (Biorad, Hercules, CA) was used to reverse-transcribe RNA to cDNA. Quantitative PCRs were performed using the LightCycler 480 DNA SYBR Green I Master Kit (Roche Applied Science) and the LightCycler technology (LC480). The primer pairs K23-s (5′-gcctccgaaggaccttagac-3′) and K23-a (5′-agatcttccctgggacctgt-3′) and ALAS1-s (5′-ccactggaagagctgtgtga-3′) and ALAS1-a (5′-accctccaacacaaccaaag-3′) were used for the amplification of human *KRT23* and *ALAS1* (Beer et al. 2015), respectively. Mouse tissues were analyzed by quantitative RT-PCRs according to protocols described previously (Fischer et al. 2011) using primers for *Krt23*, mK23-s (5′-tcatgaagaaacgccatgag-3′) and mK23-a (5′-ggctgactgctctctgaacc-3′), and published primers for *B2m* and *Krt2* (Fischer et al. 2014). Quantities of target relative to reference transcripts were calculated according to a published mathematical model (Pfaffl 2001). The statistical significance of differences in expression levels was tested with the two-sided *t*-test with *P* < 0.05 being considered significant.

The K10x1 mRNA variant of the mouse (*Mus musculus*) was amplified from mouse sole skin cDNA using the primers Mm-K10x1-s (5′-aacgagattcagacctaccg-3′) and Mm-K10x1-a (5′-ttagtatcccagctagtttcagc-3′) according to a published PCR protocol (Eckhart et al. 1999) using an annealing temperature of 63 °C and 35 amplification cycles.

### Western Blot Analysis

Proteins were extracted from cells by homogenization with the Precellys system (VWR International, Radnor, PA) in Laemmli extraction buffer containing 2% SDS. The Micro BCA protein assay kit (Thermo Fisher Scientific) was used to determine protein concentrations. Ten micrograms protein per lane were electrophoresed through an ExcelGel SDS gradient 8–18% polyacrylamide gel (GE Healthcare Life Sciences, Chicago) and blotted onto a nitrocellulose membrane. The membrane was incubated with anti-K23 antibody (1:1,000) that had been raised by immunizing guinea pigs with the synthetic peptide GRLVLCQVNEIQKHA, corresponding to amino acid residues 408–422 of human K23, coupled to keyhole limpet protein. After overnight incubation at 4 °C and washing, the membrane was incubated with goat antiguinea pig IgG (1:10,000, Abnova) coupled to horseradish peroxidase for 1 h at room temperature. Subsequently, the K23 bands were visualized using the enhanced chemiluminescence system (SuperSignal West Dura Extended Duration Substrate, Thermo Fisher Scientific). To detect other proteins, membranes were washed and consecutively re-incubated with the following primary antibodies: mouse anti-K2 (Progen, 1:100), guinea pig anti-K5 (Covance, 50 µg in 1 ml), mouse anti-GAPDH (HyTest Ltd, 1:2,000), and sheep antimouse IgG (GE Healthcare Life Sciences, 1:10,000) coupled to horseradish peroxidase as secondary antibody.

### Histology, Immunohistochemistry, and Immunofluorescence Labeling

Human tissue samples were obtained from plastic surgery and pathology under approval by the Ethics Committee at the Medical University of Vienna (EK2011/1149). Skin samples were prepared from sacrificed mice (Fischer et al. 2014) and from a dead naked mole rat that had been maintained in the Vienna Zoo. Skin samples from a stranded bottlenose dolphin (*T. truncatus*) (SW1999/197) were kindly provided by Rob Deaville, Zoological Society of London, London, UK. The samples were fixed with 7.5% formaldehyde, embedded in paraffin and sectioned at 4 µm thickness. Hematoxylin and eosin staining were performed according to a standard protocol (Fischer et al. 2011).

Immunohistochemistry was performed according to published protocols (Jäger et al. 2007) with modifications. In brief, tissues were fixed with 7.5% formaldehyde, embedded in paraffin and sectioned at 4 µm thickness. Antigens were demasked with citrate buffer (pH 6), and the samples were incubated with an antiserum (dilution 1:1,000) against K23 that was produced by immunizing guinea pigs with the synthetic peptide C-REESKSSMKVSATPK, corresponding to amino acid residues 383–397 of human K23, coupled to keyhole limpet protein. The biotinylated goat anti-guinea pig IgG (Vector Labs, 1:100) was used as secondary antibody. Goat serum (10%) (DAKO) was added to the secondary antibody to suppress unspecific binding. For color development, the sections were incubated with streptavidin–biotin–horseradish peroxidase (HRP) complex and 3-amino-9-ethylcarbazole (DakoCytomation, Glostrup, Denmark). To test the specificity of the staining, the primary antibody was replaced by normal guinea pig IgG (400 µg/ml, Santa Cruz Biotechnology). In other experiments guinea pig anti-K1 (Progen, 1:1,000), mouse antiK2 (Acris, 1:100), rabbit anti-K10 (Covance, 1:1,000) were used as primary antibodies followed by suitable second step antibodies and HRP conjugates. Nuclei were counterstained with hematoxylin. No immunohistochemistry protocol could be established for the other K23 antibody. Immunofluorescence labeling was performed according to a published protocol (Fischer et al. 2014) with the following primary antibodies: rabbit anti-K1 (Covance, 1:1,000), mouse anti-K2 (Acris KS 2.342.7.4, 1:200), guinea pig anti-K10 (Progen, 1:2,000).

## Supplementary Material

Supplementary data are available at *Molecular Biology and Evolution* online.

## Supplementary Material

Supplementary Data
