# Supplementary material for: Differential Evolution of the Epidermal Keratin Cytoskeleton in Terrestrial and Aquatic Mammals
Source: Mol Biol Evol. 2018 Dec 4;36(2):328–40. doi: 10.1093/molbev/msy214 (PMC6367960; doi:10.1093/molbev/msy214)
Supplement: Supplementary Data [file msy214_supp.pdf]

**Supplementary Data: Supplementary Figures and Tables**

# Differential evolution of the epidermal keratin cytoskeleton in terrestrial and aquatic mammals

Florian Ehrlich, Heinz Fischer, Lutz Langbein, Silke Praetzel-Wunder, Bettina Ebner, Katarzyna Figlak, Anton Weissenbacher, Wolfgang Sipos, Erwin Tschachler, Leopold Eckhart

**Content**

Supplementary Figures S1-S9

Supplementary Tables S1-S7

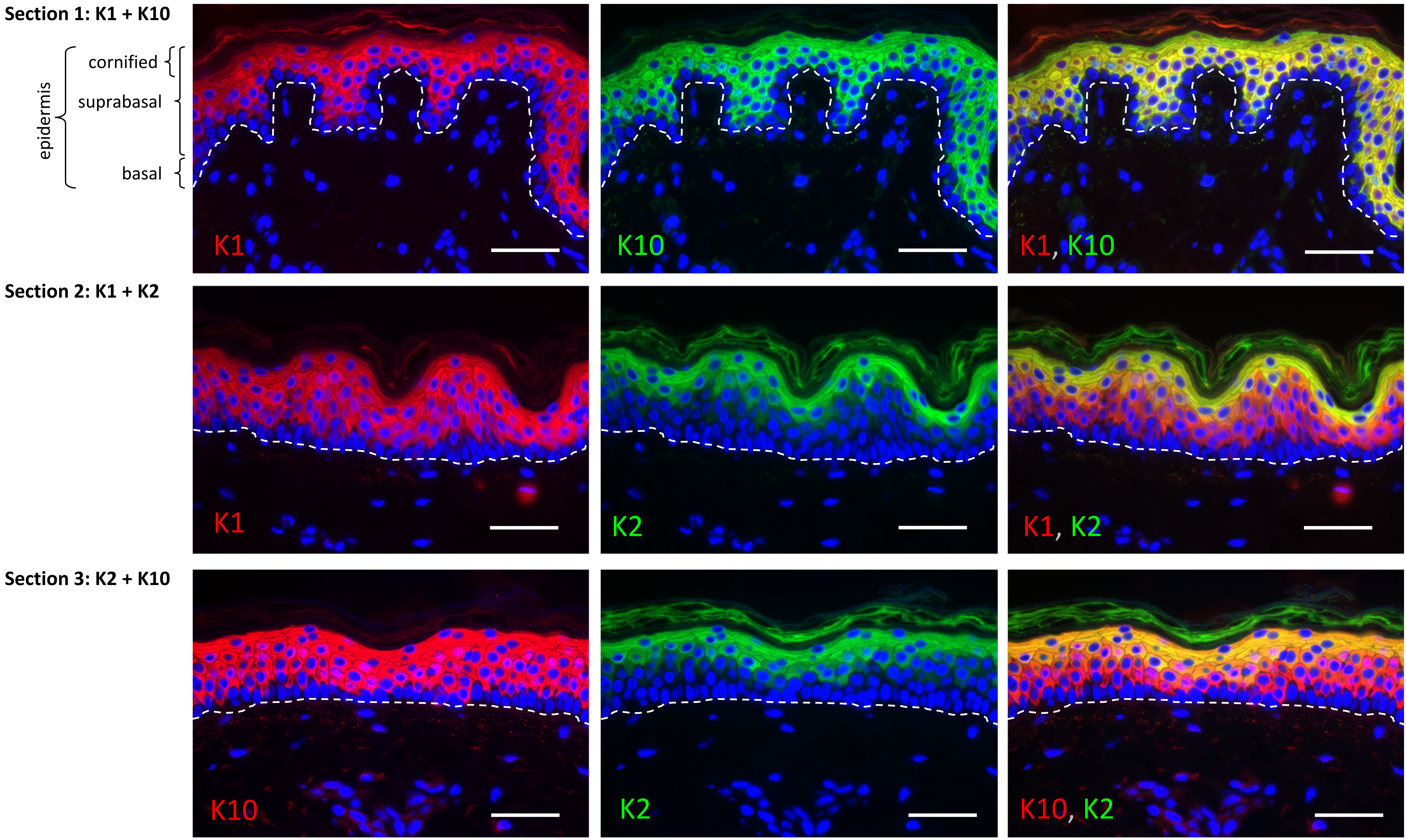


**Suppl. Fig. S1. Immunofluorescence labeling of the predominant suprabasal keratins (K1, K2, K10) in human epidermis.** Three sections of human abdominal skin were immunolabeled with antibodies against K1 and K10 (first row), K1 and K2 (second row), and K10 and K2 (third row). Nuclear DNA was labeled with Hoechst 33258 dye (blue). The dermo-epidermal junction is indicated by a broken line. Note that epitopes of antibodies are differentially masked by protein cross-linking in the cornified layer (stratum corneum) and therefore the labeling intensity in this part of the epidermis is not proportional to the amount of antigen. Scale bars, 50 µm.

# A

```
>Bas_K12
MSLSVRTSGLSQRLSSQSGTLGRARGISASSIGSSYGGSAFGFGGSCGGGFSAASMFGSSSGFGGGSGSS
FAGGLGAGYAGGRGGGFGSLGIGFGGSPGWGSLGILSGNDGGLLSGSGKETMQNLNDRLASYLDKVRALE
EANADLENKIREWYETRGPGTGDPRSQNDYSKYYLLIEDLRNKIISDSTANAQLLLQIDNAKLAAEDFRM
KYENELALRQNVEADINGLRRVLDEMTLARADLETQIETLNEELAYLRKNHEEELQSFRAGGPGQVSVEM
DAAPGVDLTSVLNDMRGQYEAIAEQNREDAEAWFIEKSGELRKEISSNTEELQCSKSVVTDLRRALQNLE
IELQSQFAMKKSLEDSLAETEGDYCGQLSQARQLIGSLEEQLLQVRADAERQSADYQLLLNIKARLELEI
ETYRRLLDGEAQGDSLDESSHVTASTSQAPSTDSSKDPTRARKIKTIVQEVVNGEVVSSQVQEVEELI

>Bas_K13-c
MSSFLQNSSASYGGGFGGGSCKLGGGRSISTGSTRFFSGGSAGGFGGGINCGFGGGAGSGLGGGYGGGLG
GGFGGGLGGGLGGGFGGGFGDFGGGDGSLLSGNEKITMQNLNERLASYLGKVRALEEANTELEVKIRDWY
LKKSPVSQERDYSPYFKTIEDLRGKILAATIDNNHIILEIDNSRLAADDFRLKYENELALRQSVEADTNG
LRRVMDELTLLKTDLEMQIENLIEELTYVKKNHEEEMKEFRNQVVGQVNVEMDATPGIDLTRVLTEMREQ
YEAMAEKNRRDAEEWFRGKSAELTKEVSSSTAIIQTSKTEITELRHTLQGLEIELQSQLSMKAGLESTLA
ETECRYALQLQHIQGLISNIEAQLSELRNEMECQNQEYKMLLDIKARLEQEIATYRSLLEGQDTRLPAFA
TGGSTIISTSGTTGSTRRLIEKNKP

>Bas_K14
MTSRQFTSSSSMKGTSGFGGGCSRGPSTLVGGSCRAPSAYGGQSSSRYNAGCAYGLGGGYGGGYSSSSSF
GGALGSGFSGGYGSGVGAGYSGGFGGGSGGGFSYGDGLLVGSEKVTMQNLNDRLASYLDKVRALEEANTD
LEVKIRDWYQKQRPTEVRDYSLYFKTIEDLRNKILAATVDNASVVLQIDNARLAADDFRTKYETELNLRL
SVEADINGLRRVLDELTLARADLEMQIESLKEELAYLRKNHEEEMNALRGQVGGDVNVEMDAAPGVDLSR
ILNEMRDQYEKMAEKNRKDAEDWFFSKTEELNHEVAANSELVQSSKTEISELRRTLQNLEIELQSQLSMK
SSLENSLEETKSRYCMQLGQIQELISNMEEQLAQLRCEMEQQNQEYKILLDVKTRLEQEISTYRRLLEGE
DTHLSTSQFSTGSQSSRDVTSSSRQIRTKVLDVHDGKVVSSHEQVIRTKN

>Bas_K15
MSTTFLQTSSSTFGGGSTWGGSLTAGGGGFAGGSLYGGGGSRNISASSARFVSSGSAGGYGGGFSGGAGS
GLGGGFGGGLGGGLGGGFGGGFGDFGGGGLLSGNEKLTMQNLNERLASYLEKVRALEEANADLEVKIRDW
YQKQRPTEIKDYSPYFKTIEELRDKILAATIDNSRVILEIDNARLAADDFRLKYENELALRQSVEADING
LRRVLDELTLAKTDLELQTESLNEELAYLRKNHEEEMKEFSNQLAGQVNVEMDAAPGVDLTRVLLEMREQ
YEAMAEKNRRDAEAWFFSKTEELNEEVASNTEIIQTSETEIVDLRRTMQGLEMELQSQLSMKAGLESTLA
ETECRYALQLQHIQGLISSVEAQLSELRSEMECQNQEYKMLLDIKTRLEQEIATYRSLLEGQDSRMAGIG
TREASLGGGGSGKVRINVEESVDGKVVSSRKRDI

>Bas_K17-h
MTTTNRQFFSSSSIKGSSGLGGGLSLTSRQQSGSLGAGSCRLGSAGSLGNALGDGSYSSCYSFGSGGGYG
SGGYGSGGYGSGGYGSGVYGSGSFGTGGYGSGLGGGDGLLVGSEKATMQNLNDRLASYLDKVHALEEANT
ELEVKIHNWYQKQTPGPATDYSHYFKTIEDLRNQILKATTDNANILLQIDNARLAADDFYTKFETEHALC
MSVEADTNGLRRVLDELTLARADLEMQIENYKEELAYLRKNHEEEMNALRGQVGGEISVEMDAAPGVDLS
RILNEMREQYEKMAEKNRKDAEDWFLSKTEELNREVTTNCELAQSSKCEVSELRRTMQALEIELQSQLSM
KASLENSLAETENRYCMQLGQIQGLICNVEEQLAQLRCETEQQNQEYKILLAVKTRLEQEIATYRRLLEG
EDSQLIHHKLRESATTRQVRTIVEEVKDGRVISSHEQVHQTTH

>Bas_K18
MSFSAQSTFSSYRSAGSVQSPGHRVRPVSSAASVYAGAGGSGSRISVSRSTSVRGGWGSGNLGAGMAGGL
VGVGGIQGEKETMQDLNDRLASYLERVRSLEADNRRLESKIREHLEKKGPQVRDWGHYLKTIEDLRAQIF
ASSVDNARIVLQIDNARLAADDFRVKYETELAMRQSVESDIHGLRKVIDDTNVTRLQLETEIEALKEELL
FMKKNHEEEVKGLQNQIANSGLTVELDAPKAQDLSKIMADIRAQYDELAQKNREELDKYWSHQIEESATV
VTSQTAEIGAAEMTLTELRRTVQSLEIDLDSMRNLKASLENSLREVEARYAMQMEQLNGVLLHLESELAQ
TRAEGQRQTQEYEALLNIKVKLEAEINTYRRLLEDGEDFSLGDALDNSNSMQTIQKTTTLRLVDGKVVSE
TSEAKVLRH

>Bas_K19
MTSYSLRQSSATSSFGGLGSGSMRFGAGGAFRAPSIHGGSGGRGVSVSSARFVSSSSSGGYGGGYAGALA
GSDGLLTGNEKVTMQNLNDRLASYLEKVRALEEANDDLEVKIRDWYQKQGPGPARDYSHYFKTIEDLRGQ
ILGTTIENSRIVLQIDNARLAADDFRTKFETEHALRVSVEADINGLRRLLDELTLARTDLEMQIEGLKEE
LAYLRKNHEEEISVLKGQVGGQVNVEVDSAPGIDLAKILSDMRSQHEVMAEKNRKDAEAWFTSQTEELNR
EVAGHTEQLQISKTEVTDLRRTLQGLEIELQSQLSMKAALEGTLAETEARFGAQLAHIQALISGVEAQLS
NVRADTERQNQEYQHLMDIKSRLEQEIATYRNLLEGQDAFYNNLPTPKVL

>Bas_K23
MNSSHSFSQTPSGSLYGTGSSWGQPGRFPRAPSVHGGAGGVCISLSFSSPRCPPPGGSWGSGRGSSLLGG
NGKAMMQNLNDRLASYLDKVRALEEANVKLESCILKWHQQRDPGSEQDYSQYEEKVSCLQEQIVDCKMTN
AQITLLIDNARIAVDDFSHKYENEHSVKKNLEIEVESLRNNLDDLTIVTTDLEQEVEGMREELILMKKRH
EQEMEEHHVPNDFKVNVKVDTAPGEDLIKVLEDMRQEYEFIIKKHQDWYKEQLAAMTQEVARPTAVQSSQ
SDIRELKRTFQALEIDLQAQCNKKSALENTLSETQSRYSCQLQDIQRIISHYEEELMQLRHDLECLSNEY
KVLLGIKTHLEKEISTYRQLLDGENGGTMEESKSSVKAPKIKVIIQESINGRIILSQVNEI

>Bas_K31
MPYSCSLPSLSCRSSCFSRPCVPPSCHGSTLPGACNIPANVGNSSWSCEGSFNGNEKETMQFLNGRLASY
LEKVRQLERENAELESRIRERSQQQDPLVCPNYQSYFRTIEELQQKILCTKAENTRLVVQIDNAKLAADD
FRTKYEMELGLRQLVESDINGLRRILDELTLCKSDLEVQVESLKEELTCLKSNHEEEVNSLRSQLGDRLN
VEVDAAPTEDLNRVLNETRCQYEALVESNRRDVEEWFTRQTEELNGQVVSSSEQLQSYQLEITELRRTVH
ALEVELQAQHNLRDSLENTLTEIEARYSSQLAQVQCLIGKVEAQLAEIRRDLEQQNQEYRVLLDVWARLE
GEINTYRGLLESEDCKLPCNPCATTNACVKTIMPCVSSACTPCAPATRFSTCVPHPNCGPCNSYVC
```

>Bas_K36
MASQLCSPIFSSGSIKGLCGTTGGVSRVSCVRSVGSCRVSGLSGVGGSASSVRLGLSGLGSCLPRSCLSS
GYHSSCFTGSGGWSCEGSFNGNEKETMQFLNGRLASYLEKVRQLERENAELESRIRERSQQQDPLVCPNY
QSYFRTIEELQQKILLTKADNARLVLQIDNAKLAADDFRTKYETELGMRQLVEADTNGLRRILDELALCK
ADLEMQVESLKEELMCLKKNHEEEVNTLRCQLGDRLNVEVDAAPPVDLNKILDEMRCQYEALVENNRRDV
EAWFNTQTDELNQQVVSSSEQLQCCQTEIIELRRTVNALEIELQAQHSMRNSLESTLAETEARYSSQLAQ
IQGLIGNVEAQLSEIRCDLERQSQEYQVLLDVKARLESEITTYRCLLEGEDCKLPTHPCAMECKPAVRVP
YISSVPCAPAPQLSTQIRTIKEEIRDGKVISSREHVQPL

>Pc_K12
MSLSVRTSGLPQWLSSQSGTLGRARGISASSIGSSYGGSALGFGGSYGGGFSAASMFGSSSGFGGGSGSS
FAGGLGAGYGGARGGGFGSLGIGFGGSPGGGSLGILSGNDGGLLSGSEKETMQNLNDRLASYLDKVRALE
EANADLENKIREWYETRGSGTGDPGSQNDYSKYYLLIEDLRNKIISDSTANAQLLLQIDNARLAAEDFRM
KYESELALCQNVEADINGLRRVLDEMTLARADLEMQIETLNEELAYQKKNHEEELQSFRAGGPGQVSVEM
DAAPGVDLTSLLNDMRGRYEAIAEQNREDAEAWFIEKSGELRKEISSNAEELQSSKSVVTDLRRALQNLE
IELQSQLAMKKSLEDSLAETEGDYYGQLSQMQQLIGSLEEQLQQVRADAERQSADYHLLLNIKARLELEI
ETYRRLLDGEAQGDCLDESSCVTASNSQTPSTDSSKDPTRARKIKTIVQEVVNGEVVSSQIQEIEELI

>Pc_K13
MSCFLQNSSASYGGGSCKLGGGRSISSGSTQFYSGGSAGGCGGGLSGGLGGAVGCGFGGGFGGGSFCGSF
GSGFADFGGGYGGGLGTCLSGGDGGLLYGNEKSTMQNLNDRLASYLDKVRTLEEANADLEAKIRDWYLKQ
RPVGPERDYSHYFRTIEDLRDKILAATIDNNQVILEIDNSRLAADDFRLKYENELALHQSVVADINGLRR
VLDELTLMKTDLEMQIESLKEELAYLRKNHEEEMKEFRNQVVGQVNVEMDATPGIDLSRVLAEMREQYEA
LAEKNRRDAEEWFHSKSTELTKEVSSSTAIIQTSKTTITELRHTLQSLEIELQSQLSMKAGLESTLADTE
CRYALQLQQIQGLISSIEAQLSELRSEMECQNQEYKMLLDVKTRLEQEIATYRSLLEGQDSKIPGFTTGG
NFSHGSTISTHRVSEKIKL

>Pc_K14
MTSRQFTSSSSIKGSCGIGGGSNLGSFVMAGGSCRAPSASGGLSVSSSRYSSGGVCGLGGSYGGNYSSSS
CFGGGLGSGVCGGYGGGLGVCYSGGLGGGLGGGDGLLGGSEKVTMQNLNDRLASYLDKVRALEEANADLE
VKIRDWYQKQRPTEIRDYSNYFRIIEDLRNKILAATVDNANVVLQIDNARLAADDFRTKYETELNLRASV
EADINGLRRVLDELTLSRADLEMQIESLKEELAYLRKNHEEEMTVLRGQVVGEVNVEMDAAPGVDLSRIL
NEMRDQYEKMAEKNRKDAEDWFFTKTEELNREVATNSELVQSSKSEVSELRHTLQSLEIELQSQLSMKTS
LENSLEETKGRYCMQLSQIQGMICSVEEQLAQLRCEMEQQSQEYQMLLDVKTRLEQEIATYRRLLEGEDT
HLSSSQFSSGSQSTRDVTSSSRQIRTKVLDMHDGKVVSSHEQVVRTKN

>Pc_K15-h-p
MSTTFLQTSSSTFGGGPTWRGSLMAGGGGFGGGSLYGGGGSRSISASSARFVSSGSAGGYGGGFSGGAGS
GLGGGFGGGFGDFGGGGLLSGNEKLTMQNLNDRLASYLEKVRALEEANADLEVKIRDWYQKQSPSIPEHD
YSPYFKTIEELRDKILAATIDNSRVILETDRARLAADDFRLKYENELALRVSVEADTHGLRRVLDELTLV
RTDLEMQLEGLKEELAYLRKNHEEEMKEFRNQVVGQVNVEMDATPGIDLSRVLAEMREQYEALAEKNRRD
AEEWFHSKVTELTKEVSSSTAIIQTSKTTITELRHTLQSLEIELQSQLSMKAGLESTLADTECRYALQLQ
QIQGLISSIEAQLSELRSEMECQNQEYKMLLDVKTRLEQEIATYRSLLEGQDSXXXXXXXXXASLGGGGS
GKVRINVEESVDGEVVSSRKRDI

>Pc_K17
MTTTNRQFSSSTSIKGSSGLGGGLSFTPCQLSGSLGNALGGGSYSSCYSFGSGGGYGSGGYGSGGYGSGG
YGSGGYGSGGFGTGGCGSGFGGGDGLLGGSEKATMQNLNDRLASYLDKVRILEEANRELEVKIHDWYQKQ
GPEPTCDYSSYFKTIEDLKNKILVATMDNANILLPIDNTRLAADDFRTKFETERALRASVEADISGLRRV
LDELTLARADLEMQIENLKEELAYLRKNHEEEMNVLRGQVGREINVEMDAAPGVDLSRILNEMREQYEKM
AEKNRKDAEDWFFCKTEELNREVATNSELVQSSKSEVSELRRTLQNLEIELQSQLSMKASLEGSLAETEN
RYCLQLSQIQGLICSVEEQLAQLRCEMAQQSQEYQMLLDVKTRLEQEIATYRRLLEGEDTQLPQYKSREP
VTTRQVRTIVEEVQDGRVISSREEVHQTTH

>Pc_K18
MSFSAQSTFSSYRSVGSVQPPGHRVRPVSSAASVYAGAGGSGPRLSVSRSTSVRGGWGSGILGAGMAGGL
VGVGGIQGEKETMQDLNDRLASYLERVRSLEADNRRLESKIREHLEKKGPQVRDWGHYLKTIEDLRAQIF
ASSVDNARIVLQIDNARLAADDFRVKYETELAMRQSVESDIHGLRKVIDDTNVSRLQLETEIETLKEELL
FMKKNHEEEVKGLQNQIANSGLTVELDAPKAQDLSKIMADIRAQYDELAQKNREELDKYWSQQIEESATV
VTSQTAEIGAAEMTLTELRRTVQSLEIDLDSMRNLKASLENSLREVEARYAMQMEQLKRALLHLELELAQ
TRAEGQRQTQEYEALLNIKVKLEAEINTYRRLLEDGEDFNLGDAVDSSNSMQTIQKTTTLRLMDGKVVSE
TSDTKVLRH

>Pc_K31
MPYSCSLPSLSCHSSCFSWPCVPPSWRGSTLPGSCNILAKVGNSGWFCEGSFNGNEKETMQFLNDRLASY
LEKVRQLERDNAELESRIRERSQQQDPLVCPNYQSYFRTIEELQQKILCTKAENTRLVVQIDNAKLAADD
FRSKYEMELGLRQLVESDINGLRRILDELTLCKSDLEVQVESLKEELICLKSNHEEEANSLRRQLGDRLN
VEVDAAPTVDLNDVLNETRSQYEALAESNRRDVQEWFTRQTEELNRQVVSSSEQLQSYQAEITELRRTAR
ALEVELQAQHNLRDSLESALAETEARYGAQLAQVQCLVSNVEAQLAEIRRDLERQSQEYQVLLDVRARLE
CEINTYRGLLEREDCKLPSNPCATTNACGKTIMPCVSSACTPCTPAARFSPCVPRPHCGPCNSYMC

>Lv_K12
MSLSVRTSGIPQWLSSQSGTLGRARAISASSIGSSYGGSAFGFGGSYGGGFSAASMFGSSSGFGGGSGSS
FAGGQGTGYGGARGGGFGSLGIGFGGSPGGGSLGILSGNDGGLLSGSEKETMQNLNDRLASYLDKVRALE
EANADLENKIREWYEIRGSGDPQNDYSKYYLLIEDLRNKIISDSTANAQVLLQIDNARLAAEDFRMKYEN
ELALCQNVEADINGLRKVLDEMTLARADLEMQIEILSEELAYRNKDHEEELQSFRAGGPGQVSVEMDAAP
GVDLTSLLNDMRGQYEAIAQQNREDAEAWFIEKSAELRKEISSNTEELQSSKSVVTDLRRVLQTLEIELQ
SQLATKKSLDDSLAETEGDYCSQLSQVRQHIGNLEEQLLQVRGDAERQSADYQLLLNIKARLELEIETYR
RLLDGEAQGDCLDESSRVTASNSQAPSTDSSKDPTRARKIKTIVQEVVNGEVVSSQVQEIEELI

>Lv_K13-c
MSCFLQNSSASYGGGSCKLGGGRSISSGSTRFCSGGSAGGFGGGISCGFGGGAGSGLGGGFGGGLGGGSF
GGCFGGGFGDFGGGYGGGLVVGLSGGGGGLLSGNEKLTMQNLNDRLASYLEKVRALEEANADLEVKIRDW
LLKQSPTSPERDYSAYFKTIEDLRDKILAATIDNNRIILEIDNSRLAADDFRLKYENELTLRQSVEADIN
GLRRVLDELTLMKTDLEIQIENLNEELAYMKKNHEEEMKELRNQVVGQVNVEMDATPGIDLSRVLAEMRE
QYEAMAEKNRRDAEEWFNSKSEELTKEVSSSTAIIQTSKTEITELRRTLQGLEIEMQSQLSMKAGLESTL
AETECRYALQLQQIQGFISSIEAQLSELRSEMECQNQEYKMLLDIKARLEQEIATYRSLLEGQDAKLPGY
TTGGTISRGSTISTSGTTSTRRIPEKNKP

>Lv_K14+
MTSRQGTSSSSMKGSYVIGGGPSCGSSVWAGGSYRAPSNYGGMSSSRYSSGGVCGLGGGYGCNYSSSSSF
GGAMGSGFGGGYGGGVGAGYGGGFGGGSGGGLGGGFGGGDGLLVASEKVTMQNLNDRLASYLEKVRALEE
ANADLEVKIRDWYQKQRPTEIRDYSAYFRIIEDLRNKIFTATVDNANVVLQIDNARLAADDFRTKYETEL
NLRLSVEADINGLRRVLDELTLARADLEMQIESLKEELAYLRKNHEEEMTILRGQVGGDVNVEMDAAPGV
DLSRILNEMRVEMDAAPGVDLSRILNEMREQYEKMAEKNRKDAEDWFFSKTEELNREVASNSEMVQSSKS
EISELRRTMQNLEIELQSHLSMKSSLENSLEETKNRYCLQLSQIQGLICNVEEQLAQLRCEMEQQSQEYK
ILLDVKTRLEQEIATYRRLLEGEDAHLSSSQFSSGSQSSRDVTSSSRQIRTKVLDVHDGKVVSSHEQVIR
TKN

>Lv_K15
MSTTFLQTSFSTFGGGSTWGGSLMAGGGGFGGGSLYGGGGSRSISTASVRFVSSGSAGGYGGGFSGGAGS
GLGGGFGGGFGGGFGDFGGGGLLSGNEKLTMQNLNDRLASYLEKVRALEEANADLEVKIRDWYQKQSPTR
PEHDYSPYFKTIEELRDKILAATIDNSRVILEIDNSRLAADDFRLKYENELALRQSMEANINGLHRVLDE
PTLTRADLELQTESLNEELAYLRKNHEEEMKELSNQLAGQVNVEMDAAPGVDPTRVRAEMREQYEAMXEK
NRRDAEAWLFSKTEELNKEVASNTEMIQTSKTEIVDLRRTVQGLEMELQSQLSKKAGLESTLAETECRYA
LQLQQIQGFISSIEAQLSELRSEMECQNQEYKMLLDIKTRLKREIATYRSLLEGQDARMASIGTREASLG
GGGSGKVLINVEESVDGKGVSSRKRDI

>Lv_K17
MTTTNRQFSSSNSIKGSSGLGGSLSFTSCQLSGSLGNAPGGGSYSSCYSFGSGGGCGSGGYGTGGYGSGF
GGGDGLLGGSEKATMQNLNDRLASYLEKVRALEEANADLEVKIRDWYQKQRPTEIRDYSAYFKTIEDLRN
KILMATTDNANVLLQIDNSRLAADDFRTKFETEQALRVSVETDINGLRRVLDELTLARADLEMQIENLKE
ELVYLRKNHEEEMNVLRGQVGGEINVEMDAAPGVDLSRILNEMREQYEKMAEKNRKDAEDWFFSKTEELN
REVASNSEMVQSSKSEISELRRTMQNLEIELQSHLSMKASLENSLAETENRYCLQLSQIQGLVCSVEEQL
AQLRCEMGQQSQEYKILLDVKTRLEQEIATYRRLLEGEDTQLTQQKPREPVTTRQVRTIVEEVQDGRVIS
SREQVHQTKN

>Lv_K18
MSFSAQSTFSSYRSLGSAQSPGHGVRPVSSAASVYAGAGGSGSRISVSRSTSVRGGWGSGNLGAGMAGGL
VGVGGIQGEKETMQDLNDRLASYLERVRSLEADNRRLESKIREHLEKKGPQVRDWGHYLKTIEDLRAQIF
ASSVDNARIVLQIDNARLAADDFRVKYETELAMRQSVESDIHGLRKVIDDTNVTRLQLETEIEALKEELL
FMKKNHEEEVKGLQNQIANSGLTVELDAPKAQDLSKIMADIRAQYDELAQKNREELDKYWSQQIEESTTV
VTSQTAEIGAAEMTLTELRRTVQSLEIDLDSMRNLKASLENSLREVEARYAMQMEQLNGVLLHLESELAQ
TRAEGQRQTQEYEALLTIKVKLEAEINTYRRLLEDGDDFNLGDAVDSSNSMQTIQKTTTLKFVDGKVVSE
TSDTKVLRH

>Lv_K19
MTSYSYRQSSATSSFGGLGSGSMRFGAGGAFRAPSIHGGSGGRGVSVSSARFVSSSSSGGYGGGYAGALA
SSDGLLAGNEKLTMQNLNDRLASYLQKVRALEEANGDLEVKIRDWYQKQGPGPARDYSPYFKTIENLRDQ
ILGATIENSKIVLQIDNARLAADDFRTKFETEHALRVSVEADINGLRRVLDELTLARTDLEMQIEGLKEE
LAYLKRTHEEEISVLKSQVGGQVSVEVDSAPGIDLAKILSNMRSQYEVMAEKNRKDAEGWFTSQTEELNR
EVAGHTEQLQISKTEVTDLRRTLQGLEIELQSQLSMKAALEGTLAETEARFGAQLAQIQALISGVEAQLS
DVRADTERQNQEYQHLMDIKSRLEQEIATYRNLLEGQDAYYNNLSIPKVL

>Lv_K31
MPYSCSLPSLSCSSSCFSWPCVPPSCHGSTLPWSCNIPVNVGNSGCFCEGSFNGNEKETMQFLNGRLASY
LEKVRQLERDNAELESRIQERSQQQDPLVCPDYQSRFRTIEELQQKILCTKAENTRLVVQIDNAKLAADG
FRTKYEMELGLRQLVESDINGLRRILDELTLCKSDLEAQVESLKEELLCLKSNHEEEANSLRRQLGDRLN
VEVDAVPTVDLNKVLNETRSQYEALVESNRRDVQEWFTRQTEELNGQVVSSLEQLQSYQAEITELRRTVH
ALEVELQAQHNLRDSLENTLTEMEARYSAQLAQVQCLISNVESQLAEIRRDLERQNQEYRVLLDVRARLE
CEINTYRGLLESEDCKLPCNPCATTNACGKTIMPCDSSACTPCAPAGRFSPCVPCPHCGPCNSYMC

>Oo_K12
MSLSAHTSGMPQWLSSQSVTLGRARGISASSIGSSYGGSAFGFGGSYGGGFTAASMFASSSGFGGGSGSS
FAGGLGAGYGGARGGGFGSLGIGFGGSPGGGSLGILSGNDGGLLSGSEKETMQNLNDRLASYLDKVRALE
EANADLENKIREWYEIRGSGDPPNDYSKYQLLIEDLRNKIISESTANAQLLLQIDNARLAAEDFRMKYEN
ELALCQNVEADINGLRKVLDEMTLARADLEMQIETLNEELAYRKKNHEEELQSCRAGGPGQVSVEMDAAP
GVDLTSLLNDMRGQYEAVAQQNREDAEAWFIEKSAELRKEISSNTEELQSSKSVVTDLRRVLQTLEIELQ
SQLATKKSLDDSLAETEGDYYSQLSQVRQLIGNVEEQLLQVRGDAERQSADYQLLLNIKAHLELEIETYR
RLLDGEAQSDCLDESSSVTASNSQAPSTDSSKDPTRAQKTKTIVQEVVNGEVVSSQVQESEEII

>Oo_K13
MSCFLQNSSASYGGGSCKLGGGRSISSGSTRFCSGGSAGGFGGGISCGFGGGAGSGLGGGFGGGLGGGSF
GGCFGGGFGDFGGGYGGGLGGSLSGGDGGLLSGNEKITMQNLNSRLASYLEKVRTLEEANADLEVKIRDW
HLKKSPTSPERDYSSYFKTIEDLRDKILAATIDNNRIILEIDNSRLAADDFRLKYENELALRQSVEADIN
GLRRVLDELTLMKTDLEMQIESLNEELAYLKKNHEEEMKELRNQVIGQVNVEMDATPGIDLSRVLAEMRE
QYEAMAEKNRRDAEEWFNSKSEELAKEVSSSTASIQTSKTEITELRRTLQGLEIELQSQLSMKAGLESTV
SETECRYALQLQQIQGFISSIEAQLSELRSEMECQSQEYKTLLDIKARLEQEIATYRSLLEGQDAKLPGF

PAGGTISTSSTTSTRRISEKTRP

>Oo_K14-c
MTSRQGTSSSSMKGSYVISGGSSCGSSARAGGSFRAPSNYGGLSSSRYSSGGVCGVGGGYGGNYSSSSSF
GGAMGSGFGGGYGGGAGGGYGGGFGGGSGGGVCGGFGGGDGLLVASEKVTMQNLNDRLASYLDKVRALEE
ANTDLEVKIRDWYQKQRPTEIRDYSAYFRTIEDLRNKILAATVDNANIVLQIDNARLAADDFRTKYETEL
NLRLSVEADINGLRRVLDELTLARADLEMQIESLKEELAYLRKNHEEEMANLRGQVGGDINVEMDAVPGV
DLSRVLNEMREQYEKMAEKNRKDAEDWFFSKTEELNREVASSSEMVQSSKSEISELRRTMQNLEIELQSH
LSMKSSLENSLEETKNRYCLQLSQIQGLICNVEEQLAQLRCEMEQQSQEYKILLDVKTRLEQEIATYRRL
LEGEDAHFSTSQFSSGSQSSRDVTSSSRQIRTKVLDVQDGKLVSSHEQIVRTKN

>Oo_K15
MSTTFLQTSSSTFGGGSTWGGSLMAGGGGFGGGSLYGGGGSRSISASSARFFSSGSAGGYGGGFSGGAGS
GLGGGFGGGFGDFGGGGLLSGNEKLTMQSLNDRLASYLEKVRALEEANADLEVKIRDWYQKQSPTSPERD
YSPYFKTIEELRDKILAATIDNSRVSLEIDNSRLAADDFRLKYENELALRQSMEADINGLRRVLDELTLA
RADLELQTESLNEELAYLRKNHEDEMKEFSNQLAGQVNVEMDAAPGVDLTRVLAEMREQYEAMAEKNRRD
AEAWFFSKTEELNKEVASNTEMIQTSKTEIVDLRRTVQGLEMELQSQLSKKAGLESTLAETECRYALQLQ
QIQGFISSIEARLSELRSEMECQSQEYKMLLDTKARLEQEIATYRSLLEGQDARMAGIGTREASLGGGGG
GKVLINVEESADGKVVSSRKRDI

>Oo_K17
MTTSNRQFSSSNSIRGSSGLGGSSSLTSRQLSGSLGNALGGGSYSGCYSFGSGGGYGSGGYGSGGYGIGS
VGTGGSGFGGGDALLGASEKATMQNLNDRLASYLEKVRALEEANTDLEVKIRDWYQKQTPGPTSDYSAYF
KTIEDLRNKILMATTDNANVLLQIDNTRLAADDFRTKFETEQALRMSVESDINGLRRVLDELTLARADLE
MQIENLKEELVYLRKNHEEEMKVLRGQVGGEINVEMDAAPGVDLSRILNEMREQYEKMAEKNRKDAEDWF
FSKTEELNREVASNSEMVQSSKSEISELRRTMQNLEIELQSHLSMKASLENSLAETENRYCLQLSQIQGL
VGSVEEQLAQLRCEMGQQSQEYQILLDVKTRLEQEIATYRRLLEGEDAQLTQHKSREPVTTRQVRTIVEE
VQDGRVISSREQVKQSNI

>Oo_K18
MSFSAQSTFSSYRSLGSAQSPGHGVRPVSSAASVYAGAGGSGSRISVSRSTSVRGGWGSGNLGAGMAGGL
VGVGGIQGEKETMQDLNDRLASYLENVRSLEADNRRLESKIREHLEKKGPQVRDWGHYLKTIEDLRLQIF
ASSVDNARIVLQIDNARLAADDFRVKYETELAMRQSVETDIHGLRKVIDDTNVTRLQLETEIEALKEELL
FMKKNHEEEVKGLQNQIANSGLTVELDAPKAQDLGKIMADIRAQYDELAQKNREELDKYWSQQIEESTTV
VTSKTAEIGAAEMTLTELRRTLQSLEIDLDSMRNLKASLENSLREVEARYAMQMEQLNGVLLHLESELAE
TRAEGQRQTQEYEALLNIKVKLEAEINTYRRLLEDGEDFSLADAVDSCNSMQTIQKTTTLKFVDGKVVSE
TSDTKVLRH

>Oo_K19
MTSYSYRQSSATSPFGGLGSGSMRFGVGGAFRAPSIHGGSGGRGVSVSSARFVSSSSSGGYGGGYAGALA
GSDGLLAGNEKLTMQNLNDRLASYLEKVRALEEANGDLEVKIRDWYQKQGPGPARDYSHYFKTIGALRDQ
ILGATVENSRMVLQIDNARLAADDFRTKFETEHALRVSVEADINGLRRLLDELTLARTDLEMQIENLKEE
LAYLKKNHEEEISVLKGQVGGQVSVEVDSSPGIDLGKILSDMRSQYEVMAEKNRKDAEAWFTSQTEELNR
EVAGHTEQLQSSKTEVTDLRRTLQGLEIELQSQISMKAALDGTLAETEARFGAQLAQIQALISGVEAQLS
DVRADTERQNQEYQHLMDIKSRLEQEIATYRNLLEGQDAYYNDLSTPKVL

>Oo_K31
MPYSCSLPSLSCSCFSWPCVPRSCHGSTLPWSCNLPVNVGNSGCLCEGSFNGNEKETMQFLNGRLASYLE
KVRQLERDNAELESRIREQSQQQDPLVCSDYQSHFRTIEELQQKILCTKAENTRLVVQIDNAKLAADGFR
TKYEMELGLRQLVESDINDLRRILDELTLCKSDLEVQVESLKEELLCLKSNHEEEANSLRRQLGDRLNVE
VDAVPTVDLNNVLNETRSHYEALVESNHRDVQEWFTRQTEELNGQVVSSLEQLQSYQAEITEQRRTVHAL
EVELQTQHNLRDSLENTLTEMEARYSAQLAQVQCLISNVESQLAEIRRDLERQNQEYRVLLDVRARLECE
INTYRGLLESEDCKLPCNPCATTNACGKTIMPCDSSACTPCAPAARFSPCVPRPHCGPCNSYTC

>Oo_K36
MATQLCSPIFSSGSIKGLCGTTGGVSQVSCVRPVGSCRVSCLPGVGGSASSVRLGLSGLGSCLPRSCPSS
GFPCSDFTGSGGWFCEGSLNGNEKETMHLLNGRLASYLEKVRQLEQDNAELECRIREWYKSQTPYIRPDY
QCFFKTIEELQQKILLSKTDNARMFLQIDNAKLAADDFRTKYDTELCMRQLVEADTKGLCCTLDELALCK
ADLEMQVESLKEELMCLKKNHEEEINKLRCQLGDRLNVEVDAAPPVDLNKILDEIRCQYETLVENNRRDL
EAWFNTQTDKLNQQVVSGSEQLQCCQKEIIELGRTVNALEIELQAQHNMRNSLESTRVETEGCYSSQLAQ
MQCLIGNVEAQLSEIRCDLGRQSQEYKVLLDVKARLESEIATYRCLLEGERLQLPARPCATECKPAVIVP
YIPSVPCAPAPQISTQICTFTEEIRDGKVISSRKHVQPL

>Tt_K12
MSLSAHTSGMPQWLSSQSVTLGRARGISASSIGSSYGGSAFGFGGSYGGGFTAASMFASSSGFGGGSGSS
FAGGLGAGYGGARGGGFGSLGIGFGGSPGGGSLGILSGNDGGLLSGSEKETMQNLNDRLASYLDKVRALE
EANADLENKIREWYEIRGSGDPPNDYSKYQLLIEDLRNKIISESTANAQLLLQIDNARLAAEDFRMKYEN
ELALCQNVEADINGLRKVLDEMTLARADLEMQIETLNEELAYRKKNHEEELQSFRAGGPGEVSVEMDAAP
GVDLTSLLNDMRGQYEAVAQQNREDAEAWFIEKSAELRKEISSNTEELQSSKSVVTDLRRVLQTLEIELQ
SQLATKKSLDDSLAETEGDYYSQLSQVRQLIGNVEEQLLQVRGDAERQSADYQLLLNIKAHLELEIETYR
RLLDGEAQSDCLDESSSVTASNSQAPSTDSSKDPTRARKIKTIVQEVVNGEVVSSQVQEIEEII

>Tt-K13
MSCFLQNSSASYGGGSCKLGGGRSISSSSTRFCSGGSAGGFGGGISCGFGGGAGSGLGGGFGGGLGGGSF
GGCFGGGFGDFGGGYGGGLGGSLGGGDGGLLSGNEKITMQNLNSRLASYLEKVRTLEEANADLEVKIRDW
HLKKSPTSPERDYSSYFKTIEDLRDKILAATIDNNRIILEIDNSRLAADDFRLKYENELALRQSVEADIN
GLRRVLDELTLMKTDLEMQIESLNEELAYLKKNHEEEMKELRNQVIGQVNVEMDATPGIDLSRVLAEMRE
QYEAMAEKNRRDAEEWFNSKSEELAKEVSSSTASIQTSKTEITELRRTLQGLEIELQSQLSMKAGLESTV
SETECRYALQLQQIQGFISSIEAQLSELRSEMECQSQEYKMLLDIKARLEQEIATYRSLLEGQDAKLPGF
PAGGTISTSSTTTRRISEKTRP

```
>Tt_K14-c
MTSRQGTSSSSMKGSYVISGGSSCGSSARAGGSFRAPSAYGGLSSSRYSSGGVCGVGGGYGGNYSSSSSF
GGAMGSGFGGGYGGGAGGGYGGGFGGGSGGGVCGGFGGGDGLLVASEKVTMQNLNDRLASYLDKVRALEE
ANTDLEVKIRDWYQKQRPTEIRDYSAYFRTIEDLRNKILAATVDNANIVLQIDNARLAADDFRTKYETEL
NLRLSVEADINGLRRVLDELTLARADLEMQIESLKEELAYLRKNHEEEMANLRGQVGGDINVEMDAVPGV
DLSRVLNEMREQYEKMAEKNRKDAEDWFFSKTEELNREVASSSEMVQSSKSEISELRRTMQNLEIELQSH
LSMKSSLENSLEETKNRYCLQLSQIQGLICNVEEQLAQLRCEMEQQSQEYKILLDVKTRLEQEIATYRRL
LEGEDARFPTSQFSSGSQSSRDVTSSSRQIRTKVLDVQDGKLVSSHEQIVRTKN

>Tt_K15-h-p
MSTTFLQTSSSTFGGGSTWGGSLMAGGGGFGGGSLYGGGGSRSISASSARFFSSGSAGGYGGGFSGGAGS
GLGGGFGGGFGDFGGGGLLSGNEKLTMQNLNDRLASYLEKVRALEEANADLEVKIRDWYQKQSPTSPERD
YSPYFKTIEELRDKILAATIDNSRVSLEIDNSRLAADDFRLKYENELALRQSMEADINGLRRVLDELTLA
RADLELQTESLNEELAYLRKNHEEEMKEFSNQLAGQVNVEMDAAPGVDLTRVLAEMREQYEAMAEKNRRD
AEAWFFSKTEELNKEVASNTEMIQTSKTEIVDLRRTVQGLEMELQSQLSKXXXXXXXXXXXXXXXXXXXX
XXXXXXXXXXXXXXXXXXXXXXXXXXXXXXXXXXXXXXXXXXXXXXXXXXXXXXXXXXXXXXXXXXXXXX
XXXXXXXXXXXXXXXXXXXXXXX

>Tt_K17-p
XXXXXXXXXXXXXXXXXXXXXXXXXXXXXXXXXXXXXXXXXXXXXXXXXXXXXXXXXXXXXXXXXXXXXX
XXXXXXXXXXXXXXXXXXXXXXXXXXXXXXXXXXXXXXXXXXXXXXXXXXXXXXXXXXXXXXXXXXXXXX
XXXXXXXXXXXXXXXXXXXXXXXXXXXXXXXXXXXXXFETEQALRVSVESDINGLRRVLDELTLARADLE
MQIENLKEELVYLRKNHEEEMKVLRGQVGGEINVEMDAAPGVDLSRILNEMREQYEKMAEKNRKDAEDWF
FSKTEELNREVASNSEMVQSSKSEISELRRTMQNLEIELQSHLSMKASLENSLAETENRYCLQLSQIQGL
VGSVEEQLAQLRCEMGQQSQEYQILLDVKXXXXXXXXXXXXXXXXXXXXXXXXXXXXXXXXXXXXXXXXX
XXXXXXXXXXXXXXXXXX

>Tt_K18-h
MSFSAQSTFSSYRSLGSAQSPGHGVRPVSSAASVYAGAGGSGSRISVSRSTSVRGGWGSGNLGAGMTGGL
VGVGGIQGEKETMQDLNDRLASYLENVRSLEADNRRLESKIREHLEKKGPQVRDWGHYLKTIEDLRLQIF
ASSVDNARIVLQIDNARLAADDFRVKYETELAMRQSVENDIHGLRKVIDDTNVTRLQLETEIEALKEELL
FMKKNHEEEVKGLQNQIANSGLTVELDAPKAQDLGKIMADIRAQYDELAQKNREELDKYWSQQIEESTTV
VTSKTAEIGAAEMTLTELRRTLQSLEIDLDSMRNLKASLENSLREVEARYAMQMEQLNGVLLHLESELAE
TRAEGQRQTQEYEALLNIKVKLEAEINTYRRLLEDGEDFSLADAVDSCNSMQTIQKTTTLKFVDGKVVSE
TSDTKVLRH

>Tt_K19-h
MTSYSYRQSSATSSFGGLGSGSMRFGVGSAFRAPSIHGGSGGRGVSVSSARFVSSSSSGGYGGGYAGALA
GSDGLLAGNEKLTMQNLNDRLASYLEKVRALEEANGDLEVKIRDWYQKQGPGPARDYSHYFKTIGALRDQ
ILGATVENSRMVLQIDNARLAADDFRTKFETEHALRVSVEADINGLRRLLDELTLARTDLEMQIENLKEE
LAYLKKNHEEEISVLKGQVGGHVSVEVDSAPGIDLAKILSDMRSQYEVMAEKNRKDAEAWFTSQTEELNR
EVAGHTEQLQSSKTEVTDLRRTLQGLEIELQSQISMKAALDGTLAETEARFGAQLAQIQALISGVEAQLS
DVRADTERQNQEYQHLMDIKSRLEQEIATYRNLLEGQDAYYNDLSTPNVL

>Tt_K31-h
MPYSCSLPSLSCSCFSWPCVPRSCHGSTLPWSCNIPVNVGNSGCLCEGSFNGNEKETMQFLNSRLASYLE
KVRQLERDNAELESRIRERSQQQDPLVCPDYQSHLRTIEELQQKILCTKAENTRLVVQIDNAKLAADGFR
TKYEMELGLRQLVESDINDLRRILDELTLCKSDLEVQVESLKEELLCLKSNHEEEANSLRRQLGDRLNVE
VDAVPTVDLNNVLNETRSHYEALVESNHRDVQEWFTRQTEELNGQVVSSLEQLQSYQAEITEQRRTVHAL
EVELQTQHNLRDSLENTLTEMEARYSAQLAQVQCLISNVESQLAEIRRDLERQNQEYRVLLDVRARLECE
INTYRGLLESEDCKVPCNPCATTNACGKTIMPCDSSACTPCAPTARFSPCVPRPHCGPCNSYTC

>Tt_K36
MATQLCSPIFSSGSIKGLCGTTGGVSQVSCVRPVGSCRVSCLPGVGGSASSVRLGLSGLGSCLPRSCPSS
GFPCSDFTGSGGWFCEGSLNGNEKETMHLLNGRLASYLEKVRQLEQDNAELECRIREWYKSQMPYIRPDY
QCFFKTIEELQQKILLSKTDNARMFLQIDNAKLAADDFRTKYETELGMRQLVEADTKGLCCTLDELALCK
ADLEMQVESLKEELMCLKKNHEEEINKLRCQLGDRLNVEVDAAPPVDLNKILDEIRCQYETLVENNRRDL
EAWFNTQTDKLNQQVVSGSEQLQCCQKEIIELGRTVNALEIELQAQYNMRNSLESTRVETEGCYSSQLAQ
MQCLIGNVEAQLSEIRCDLGRQSQEYKVLLDVKARLESEIATYRCLLEGERVQLPARPCATECKPAVIVP
YIPSVPCAPAPQSSTQICTITEEIRDGKVISSRKHVQPL


>Hs_K9
MSCRQFSSSYLSRSGGGGGGGLGSGGSIRSSYSRFSSSGGGGGGGRFSSSSGYGGGSSRVCGRGGGGSFG
YSYGGGSGGGFSASSLGGGFGGGSRGFGGASGGGYSSSGGFGGGFGGGSGGGFGGGYGSGFGGFGGFGGG
AGGGDGGILTANEKSTMQELNSRLASYLDKVQALEEANNDLENKIQDWYDKKGPAAIQKNYSPYYNTIDD
LKDQIVDLTVGNNKTLLDIDNTRMTLDDFRIKFEMEQNLRQGVDADINGLRQVLDNLTMEKSDLEMQYET
LQEELMALKKNHKEEMSQLTGQNSGDVNVEINVAPGKDLTKTLNDMRQEYEQLIAKNRKDIENQYETQIT
QIEHEVSSSGQEVQSSAKEVTQLRHGVQELEIELQSQLSKKAALEKSLEDTKNRYCGQLQMIQEQISNLE
AQITDVRQEIECQNQEYSLLLSIKMRLEKEIETYHNLLEGGQEDFESSGAGKIGLGGRGGSGGSYGRGSR
GGSGGSYGGGGSGGGYGGGSGSRGGSGGSYGGGSGSGGGSGGGYGGGSGGGHSGGSGGGHSGGSGGNYGG
GSGSGGGSGGGYGGGSGSRGGSGGSHGGGSGFGGESGGSYGGGEEASGSGGGYGGGSGKSSHS

>Hs_K10
MSVRYSSSKHYSSSRSGGGGGGGGCGGGGGVSSLRISSSKGSLGGGFSSGGFSGGSFSRGSSGGGCFGGS
SGGYGGLGGFGGGSFRGSYGSSSFGGSYGGSFGGGSFGGGSFGGGSFGGGGFGGGGFGGGFGGGFGGDGG
LLSGNEKVTMQNLNDRLASYLDKVRALEESNYELEGKIKEWYEKHGNSHQGEPRDYSKYYKTIDDLKNQI
LNLTTDNANILLQIDNARLAADDFRLKYENEVALRQSVEADINGLRRVLDELTLTKADLEMQIESLTEEL
```

AYLKKNHEEEMKDLRNVSTGDVNVEMNAAPGVDLTQLLNNMRSQYEQLAEQNRKDAEAWFNEKSKELTTE
IDNNIEQISSYKSEITELRRNVQALEIELQSQLALKQSLEASLAETEGRYCVQLSQIQAQISALEEQLQQ
IRAETECQNTEYQQLLDIKIRLENEIQTYRSLLEGEGSSGGGGRGGGSFGGGYGGGSSGGGSSGGGHGGG
HGGSSGGGYGGGSSGGGSSGGGYGGGSSSGGHGGSSSGGYGGGSSGGGGGGYGGGSSGGGSSSGGGYGGG
SSSGGHKSSSSGSVGESSSKGPRY

>Hs_K12
MDLSNNTMSLSVRTPGLSRRLSSQSVIGRPRGMSASSVGSGYGGSAFGFGASCGGGFSAASMFGSSSGFG
GGSGSSMAGGLGAGYGRALGGGSFGGLGMGFGGSPGGGSLGILSGNDGGLLSGSEKETMQNLNDRLASYL
DKVRALEEANTELENKIREWYETRGTGTADASQSDYSKYYPLIEDLRNKIISASIGNAQLLLQIDNARLA
AEDFRMKYENELALRQGVEADINGLRRVLDELTLTRTDLEMQIESLNEELAYMKKNHEDELQSFRVGGPG
EVSVEMDAAPGVDLTRLLNDMRAQYETIAEQNRKDAEAWFIEKSGELRKEISTNTEQLQSSKSEVTDLRR
AFQNLEIELQSQLAMKKSLEDSLAEAEGDYCAQLSQVQQLISNLEAQLLQVRADAERQNVDHQRLLNVKA
RLELEIETYRRLLDGEAQGDGLEESLFVTDSKSQAQSTDSSKDPTKTRKIKTVVQEMVNGEVVSSQVQEI
EELM

>Hs_K13
MSLRLQSSSASYGGGFGGGSCQLGGGRGVSTCSTRFVSGGSAGGYGGGVSCGFGGGAGSGFGGGYGGGLG
GGYGGGLGGGFGGGFAGGFVDFGACDGGLLTGNEKITMQNLNDRLASYLEKVRALEEANADLEVKIRDWH
LKQSPASPERDYSPYYKTIEELRDKILTATIENNRVILEIDNARLAADDFRLKYENELALRQSVEADING
LRRVLDELTLSKTDLEMQIESLNEELAYMKKNHEEEMKEFSNQVVGQVNVEMDATPGIDLTRVLAEMREQ
YEAMAERNRRDAEEWFHAKSAELNKEVSTNTAMIQTSKTEITELRRTLQGLEIELQSQLSMKAGLENTVA
ETECRYALQLQQIQGLISSIEAQLSELRSEMECQNQEYKMLLDIKTRLEQEIATYRSLLEGQDAKMIGFP
SSAGSVSPRSTSVTTTSSASVTTTSNASGRRTSDVRRP

>Hs_K14
MTTCSRQFTSSSSMKGSCGIGGGIGGGSSRISSVLAGGSCRAPSTYGGGLSVSSSRFSSGGAYGLGGGYG
GGFSSSSSSFGSGFGGGYGGGLGAGLGGGFGGGFAGGDGLLVGSEKVTMQNLNDRLASYLDKVRALEEAN
ADLEVKIRDWYQRQRPAEIKDYSPYFKTIEDLRNKILTATVDNANVLLQIDNARLAADDFRTKYETELNL
RMSVEADINGLRRVLDELTLARADLEMQIESLKEELAYLKKNHEEEMNALRGQVGGDVNVEMDAAPGVDL
SRILNEMRDQYEKMAEKNRKDAEEWFFTKTEELNREVATNSELVQSGKSEISELRRTMQNLEIELQSQLS
MKASLENSLEETKGRYCMQLAQIQEMIGSVEEQLAQLRCEMEQQNQEYKILLDVKTRLEQEIATYRRLLE
GEDAHLSSSQFSSGSQSSRDVTSSSRQIRTKVMDVHDGKVVSTHEQVLRTKN

>Hs_K15
MTTTFLQTSSSTFGGGSTRGGSLLAGGGGFGGGSLSGGGGSRSISASSARFVSSGSGGGYGGGMRVCGFG
GGAGSVFGGGFGGGVGGGFGGGFGGGDGGLLSGNEKITMQNLNDRLASYLDKVRALEEANADLEVKIHDW
YQKQTPTSPECDYSQYFKTIEELRDKIMATTIDNSRVILEIDNARLAADDFRLKYENELALRQGVEADIN
GLRRVLDELTLARTDLEMQIEGLNEELAYLKKNHEEEMKEFSSQLAGQVNVEMDAAPGVDLTRVLAEMRE
QYEAMAEKNRRDVEAWFFSKTEELNKEVASNTEMIQTSKTEITDLRRTMQELEIELQSQLSMKAGLENSL
AETECRYATQLQQIQGLIGGLEAQLSELRCEMEAQNQEYKMLLDIKTRLEQEIATYRSLLEGQDAKMAGI
GIREASSGGGGSSSNFHINVEESVDGQVVSSHKREI

>Hs_K16
MTTCSRQFTSSSSMKGSCGIGGGIGGGSSRISSVLAGGSCRAPSTYGGGLSVSSRFSSGGACGLGGGYGG
GFSSSSSFGSGFGGGYGGGLGAGFGGGLGAGFGGGFAGGDGLLVGSEKVTMQNLNDRLASYLDKVRALEE
ANADLEVKIRDWYQRQRPSEIKDYSPYFKTIEDLRNKIIAATIENAQPILQIDNARLAADDFRTKYEHEL
ALRQTVEADVNGLRRVLDELTLARTDLEMQIEGLKEELAYLRKNHEEEMLALRGQTGGDVNVEMDAAPGV
DLSRILNEMRDQYEQMAEKNRRDAETWFLSKTEELNKEVASNSELVQSSRSEVTELRRVLQGLEIELQSQ
LSMKASLENSLEETKGRYCMQLSQIQGLIGSVEEQLAQLRCEMEQQSQEYQILLDVKTRLEQEIATYRRL
LEGEDAHLSSQQASGQSYSSREVFTSSSSSSSRQTRPILKEQSSSSFSQGQSS

>Hs_K17
MTTSIRQFTSSSSIKGSSGLGGGSSRTSCRLSGGLGAGSCRLGSAGGLGSTLGGSSYSSCYSFGSGGGYG
SSFGGVDGLLAGGEKATMQNLNDRLASYLDKVRALEEANTELEVKIRDWYQRQAPGPARDYSQYYRTIEE
LQNKILTATVDNANILLQIDNARLAADDFRTKFETEQALRLSVEADINGLRRVLDELTLARADLEMQIEN
LKEELAYLKKNHEEEMNALRGQVGGEINVEMDAAPGVDLSRILNEMRDQYEKMAEKNRKDAEDWFFSKTE
ELNREVATNSELVQSGKSEISELRRTMQALEIELQSQLSMKASLEGNLAETENRYCVQLSQIQGLIGSVE
EQLAQLRCEMEQQNQEYKILLDVKTRLEQEIATYRRLLEGEDAHLTQYKKEPVTTRQVRTIVEEVQDGKV
ISSREQVHQTTR

>Hs_K18
MSFTTRSTFSTNYRSLGSVQAPSYGARPVSSAASVYAGAGGSGSRISVSRSTSFRGGMGSGGLATGIAGG
LAGMGGIQNEKETMQSLNDRLASYLDRVRSLETENRRLESKIREHLEKKGPQVRDWSHYFKIIEDLRAQI
FANTVDNARIVLQIDNARLAADDFRVKYETELAMRQSVENDIHGLRKVIDDTNITRLQLETEIEALKEEL
LFMKKNHEEEVKGLQAQIASSGLTVEVDAPKSQDLAKIMADIRAQYDELARKNREELDKYWSQQIEESTT
VVTTQSAEVGAAETTLTELRRTVQSLEIDLDSMRNLKASLENSLREVEARYALQMEQLNGILLHLESELA
QTRAEGQRQAQEYEALLNIKVKLEAEIATYRRLLEDGEDFNLGDALDSSNSMQTIQKTTTRRIVDGKVVS
ETNDTKVLRH

>Hs_K19
MTSYSYRQSSATSSFGGLGGGSVRFGPGVAFRAPSIHGGSGGRGVSVSSARFVSSSSSGAYGGGYGGVLT
ASDGLLAGNEKLTMQNLNDRLASYLDKVRALEAANGELEVKIRDWYQKQGPGPSRDYSHYYTTIQDLRDK
ILGATIENSRIVLQIDNARLAADDFRTKFETEQALRMSVEADINGLRRVLDELTLARTDLEMQIEGLKEE
LAYLKKNHEEEISTLRGQVGGQVSVEVDSAPGTDLAKILSDMRSQYEVMAEQNRKDAEAWFTSRTEELNR
EVAGHTEQLQMSRSEVTDLRRTLQGLEIELQSQLSMKAALEDTLAETEARFGAQLAHIQALISGIEAQLG
DVRADSERQNQEYQRLMDIKSRLEQEIATYRSLLEGQEDHYNNLSASKVL

>Hs_K20
MDFSRRSFHRSLSSSLQAPVVSTVGMQRLGTTPSVYGGAGGRGIRISNSRHTVNYGSDLTGGGDLFVGNE

KMAMQNLNDRLASYLEKVRTLEQSNSKLEVQIKQWYETNAPRAGRDYSAYYRQIEELRSQIKDAQLQNAR
CVLQIDNAKLAAEDFRLKYETERGIRLTVEADLQGLNKVFDDLTLHKTDLEIQIEELNKDLALLKKEHQE
EVDGLHKHLGNTVNVEVDAAPGLNLGVIMNEMRQKYEVMAQKNLQEAKEQFERQTAVLQQQVTVNTEELK
GTEVQLTELRRTSQSLEIELQSHLSMKESLEHTLEETKARYSSQLANLQSLLSSLEAQLMQIRSNMERQN
NEYHILLDIKTRLEQEIATYRRLLEGEDVKTTEYQLSTLEERDIKKTRKIKTVVQEVVDGKVVSSEVKEV
EENI

>Hs_K23
MNSGHSFSQTPSASFHGAGGGWGRPRSFPRAPTVHGGAGGARISLSFTTRSCPPPGGSWGSGRSSPLLGG
NGKATMQNLNDRLASYLEKVRALEEANMKLESRILKWHQQRDPGSKKDYSQYEENITHLQEQIVDGKMTN
AQIILLIDNARMAVDDFNLKYENEHSFKKDLEIEVEGLRRTLDNLTIVTTDLEQEVEGMRKELILMKKHH
EQEMEKHHVPSDFNVNVKVDTGPREDLIKVLEDMRQEYELIIKKKHRDLDTWYKEQSAAMSQEAASPATV
QSRQGDIHELKRTFQALEIDLQTQYSTKSALENMLSETQSRYSCKLQDMQEIISHYEEELTQLRHELERQ
NNEYQVLLGIKTHLEKEITTYRRLLEGESEGTREESKSSMKVSATPKIKAITQETINGRLVLCQVNEIQK
HA

>Hs_K24
MSCSSRASSSRAGGSSSARVSAGGSSFSSGSRCGLGGSSAQGFRGGASSCSLSGGSSGAFGGSFGGGFGS
CSVGGGFGGASGSGTGFGGGSSFGGVSGFGRGSGFCGSSRFSSGATGGFYSYGGGMGGGVGDGGLFSGGE
KQTMQNLNDRLANYLDKVRALEEANTDLENKIKEWYDKYGPGSGDGGSGRDYSKYYSIIEDLRNQIIAAT
VENAGIILHIDNARLAADDFRLKYENELCLRQSVEADINGLRKVLDDLTMTRSDLEMQIESFTEELAYLR
KNHEEEMKNMQGSSGGEVTVEMNAAPGTDLTKLLNDMRAQYEELAEQNRREAEERFNKQSASLQAQISTD
AGAATSAKNEITELKRTLQALEIELQSQLAMKSSLEGTLADTEAGYVAQLSEIQTQISALEEEICQIWGE
TKCQNAEYKQLLDIKTRLEVEIETYRRLLDGEGGGSSFAEFGGRNSGSVNMGSRDLVSGDSRSGSCSGQG
RDSSKTRVTKTIVEELVDGKVVSSQVSSISEVKVK

>Hs_K25
MSLRLSSASRRSCPRPTTGSLRLYGGGTSFGTGNSCGISGIGSGFSSAFGGSSSGGNTGGGNPCAGFTVN
ERGLLSGNEKVTMQNLNDRLASYLDSVHALEEANADLEQKIKGWYEKFGPGSCRGLDHDYSRYFPIIDDL
KNQIIASTTSNANAVLQIDNARLTADDFRLKYENELALHQSVEADVNGLRRVLDEITLCRTDLEIQYETL
SEEMTYLKKNHKEEMQVLQCAAGGNVNVEMNAAPGVDLTVLLNNMRAEYEALAEQNRRDAEAWFNEKSAS
LQQQISEDVGATTSARNELTEMKRTLQTLEIELQSLLATKHSLECSLTETESNYCAQLAQIQAQIGALEE
QLHQVRTETEGQKLEYEQLLDIKLHLEKEIETYCLLIGGDDGACKSGGYKSKDYGSGNVGSQVKDPAKAI
VVKKVLEEVDQRSKILTTRLHSLEEKSQSN

>Hs_K26
MSFRLSGGSRRICSRTGSGRLSGGGTGFVAGNVCVGSGARSSFSCTLEGISSGGSFCNSGGGLGSGACAG
FLGNEHSLLSGNEKVTMQNLNDRLASYLDHVHALEEANADLEQKIKGWYEKCEPGSSREHDHDYSRYFSV
IEDLKRQIISATICNASIVLQNDNARLTADDFRLKYENELALHHSVEADTSGLRRVLDELTLCTTDLEIQ
CETLSEELTYLKKSHEEEMEVLQYTAGGNVNVEMNATPGVDLTVLLNNMRAEYEDLAEQNRKDAEAWFNE
RSATLQQQISDHEGAATAARNELTELKRNLQTLEIELQSLMAVKHSYECSLAETEGNYCNQLQQIQDQIG
VMEEQLQQIRTETEGQKLEYEQLLDVKIFLEKEIDIYCNLLDGEERKSKSTCYKSKGYRPVNSGNQAKDS
TEETIVKTVVEELDQIGNLLSLRVHSVEEKSSKISNITVEQRVPSKAP

>Hs_K27
MSVRFSSTSRRLGSCGGTGSVRLSSGGAGFGAGNTCGVPGIGSGFSCAFGGSSSAGGYGGGLGGGSASCA
AFTGNEHGLLSGNEKVTMQNLNDRLASYLENVRALEEANADLEQKIKGWYEKFGPGSCRGLDHDYSRYFP
IIDELKNQIISATTSNAHVVLQNDNARLTADDFRLKFENELALHQSVEADINGLRRVLDELTLCRTDLEI
QLETLSEELAYLKKNHEEEMKALQCAAGGNVNVEMNAAPGVDLTVLLNNMRAEYEALAEQNRRDAEAWFN
EKSASLQQQISDDAGATTSARNELIEMKRTLQTLEIELQSLLATKHSLECSLTETESNYCAQLAQIQAQI
GALEEQLHQVRTETEGQKLEYEQLLDIKVHLEKEIETYCLLIDGEDGSCSKSKGYGGPGNQTKDSSKTTI
VKTVVEEIDPRGKVLSSRVHTVEEKSTKVNNKNEQRVSS

>Hs_K28
MSLQFSNGSRHVCLRSGAGSVRPLNGGAGFAGSSACGGSVAGSEFSCALGGGLGSVPGGSHAGGALGNAA
CIGFAGSEGGLLSGNEKVTMQNLNDRLASYLDNVRALEEANAELERKIKGWYEKYGPGSCRGLDHDYSRY
HLTIEDLKNKIISSTTTNANVILQIDNARLAADDFRLKYENELTLHQNVEADINGLRRVLDELTLCRTDQ
ELQYESLSEEMTYLKKNHEEEMKALQCAAGGNVNVEMNAAPGVDLAVLLNNMRAEYEALAEQNRKDAEAW
FNEKSASLQQQISHDSGAATFARSQLTEMRRTLQTLEIQLQSLMATKHSLECSLTETESNYCTQLAQIQA
QIGALEEQLHQVRTETEGQKLEYEHLLDVKVHLEKEIETYCRLIDGDGNSCSKSKGFGSGSPGNSSKDLS
KTTLVKTVVEELDQRGKVLSSRIHSIEEKTSKMTNGKTEQRVPF

>Hs_K31
MPYNFCLPSLSCRTSCSSRPCVPPSCHSCTLPGACNIPANVSNCNWFCEGSFNGSEKETMQFLNDRLASY
LEKVRQLERDNAELENLIRERSQQQEPLLCPSYQSYFKTIEELQQKILCTKSENARLVVQIDNAKLAADD
FRTKYQTELSLRQLVESDINGLRRILDELTLCKSDLEAQVESLKEELLCLKSNHEQEVNTLRCQLGDRLN
VEVDAAPTVDLNRVLNETRSQYEALVETNRREVEQWFTTQTEELNKQVVSSSEQLQSYQAEIIELRRTVN
ALEIELQAQHNLRDSLENTLTESEARYSSQLSQVQSLITNVESQLAEIRSDLERQNQEYQVLLDVRARLE
CEINTYRSLLESEDCNLPSNPCATTNACSKPIGPCLSNPCTSCVPPAPCTPCAPRPRCGPCNSFVR

>Hs_K32
MTSSCCVTNNLQASLKSCPRPASVCSSGVNCRPELCLGYVCQPMACLPSVCLPTTFRPASCLSKTYLSSS
CQAASGISGSMGPGSWYSEGAFNGNEKETMQFLNDRLASYLTRVRQLEQENAELESRIQEASHSQVLTMT
PDYQSHFRTIEELQQKILCTKAENARMVVNIDNAKLAADDFRAKYEAELAMRQLVEADINGLRRILDDLT
LCKADLEAQVESLKEELMCLKKNHEEEVGSLRCQLGDRLNIEVDAAPPVDLTRVLEEMRCQYEAMVEANR
RDVEEWFNMQMEELNQQVATSSEQLQNYQSDIIDLRRTVNTLEIELQAQHSLRDSLENTLTESEARYSSQ
LAQMQCMITNVEAQLAEIRADLERQNQEYQVLLDVRARLEGEINTYRSLLENEDCKLPCNPCSTPSCTTC
VPSPCVPRTVCVPRTVGMPCSPCPQGRY

>Hs_K33a

MSYSCGLPSLSCRTSCSSRPCVPPSCHGCTLPGACNIPANVSNCNWFCEGSFNGSEKETMQFLNDRLASY
LEKVRQLERDNAELENLIRERSQQQEPLVCASYQSYFKTIEELQQKILCSKSENARLVVQIDNAKLASDD
FRTKYETELSLRQLVESDINGLRRILDELTLCRSDLEAQVESLKEELLCLKQNHEQEVNTLRCQLGDRLN
VEVDAAPTVDLNQVLNETRSQYEALVETNRREVEQWFATQTEELNKQVVSSSEQLQSYQAEIIELRRTVN
ALEIELQAQHNLRDSLENTLTESEARYSSQLSQVQRLITNVESQLAEIRSDLERQNQEYQVLLDVRARLE
CEINTYRSLLESEDCKLPSNPCATTNACDKSTGPCISNPCGLRARCGPCNTFGY

>Hs_K33b
MPYNFCLPSLSCRTSCSSRPCVPPSCHGYTLPGACNIPANVSNCNWFCEGSFNGSEKETMQFLNDRLASY
LEKVRQLERDNAELENLIRERSQQQEPLLCPSYQSYFKTIEELQQKILCSKSENARLVVQIDNAKLAADD
FRTKYQTEQSLRQLVESDINSLRRILDELTLCRSDLEAQMESLKEELLSLKQNHEQEVNTLRCQLGDRLN
VEVDAAPAVDLNQVLNETRNQYEALVETNRREVEQWFATQTEELNKQVVSSSEQLQSYQAEIIELRRTVN
ALEIELQAQHNLRYSLENTLTESEARYSSQLSQVQSLITNVESQLAEIRSDLERQNQEYQVLLDVRARLE
CEINTYRSLLESEDCKLPSNPCATTNACEKPIGSCVTNPCGPRSRCGPCNTFGY

>Hs_K34
MLYAKPPPTINGIKGLQRKERLKPAHIHLQQLTCFSITCSSTMSYSCCLPSLGCRTSCSSRPCVPPSCHG
YTLPGACNIPANVSNCNWFCEGSFNGSEKETMQFLNDRLASYLEKVRQLERDNAELEKLIQERSQQQEPL
LCPSYQSYFKTIEELQQKILCAKAENARLVVNIDNAKLASDDFRSKYQTEQSLRLLVESDINSIRRILDE
LTLCKSDLESQVESLREELICLKKNHEEEVNTLRSQLGDRLNVEVDTAPTVDLNQVLNETRSQYEALVEI
NRREVEQWFATQTEELNKQVVSSSEQLQSCQAEIIELRRTVNALEIELQAQHNLRDSLENTLTESEAHYS
SQLSQVQSLITNVESQLAEIRCDLERQNQEYQVLLDVRARLECEINTYRSLLESEDCKLPCNPCATTNAS
GNSCGPCGTSQKGCCN

>Hs_K35
MASKCLKAGFSSGSLKSPGGASGGSTRVSAMYSSSSCKLPSLSPVARSFSACSVGLGRSSYRATSCLPAL
CLPAGGFATSYSGGGGWFGEGILTGNEKETMQSLNDRLAGYLEKVRQLEQENASLESRIREWCEQQVPYM
CPDYQSYFRTIEELQKKTLCSKAENARLVVEIDNAKLAADDFRTKYETEVSLRQLVESDINGLRRILDDL
TLCKSDLEAQVESLKEELLCLKKNHEEEVNSLRCQLGDRLNVEVDAAPPVDLNRVLEEMRCQYETLVENN
RRDAEDWLDTQSEELNQQVVSSSEQLQSCQAEIIELRRTVNALEIELQAQHSMRDALESTLAETEARYSS
QLAQMQCMITNVEAQLAEIRADLERQNQEYQVLLDVRARLECEINTYRGLLESEDSKLPCNPCAPDYSPS
KSCLPCLPAASCGPSAARTNCSPRPICVPCPGGRF

>Hs_K36
MATQTCTPTFSTGSIKGLCGTAGGISRVSSIRSVGSCRVPSLAGAAGYISSARSGLSGLGSCLPGSYLSS
ECHTSGFVGSGGWFCEGSFNGSEKETMQFLNDRLANYLEKVRQLERENAELESRIQEWYEFQIPYICPDY
QSYFKTIEDFQQKILLTKSENARLVLQIDNAKLAADDFRTKYETELSLRQLVEADINGLRRILDELTLCK
ADLEAQVESLKEELMCLKKNHEEEVSVLRCQLGDRLNVEVDAAPPVDLNKILEDMRCQYEALVENNRRDV
EAWFNTQTEELNQQVVSSSEQLQCCQTEIIELRRTVNALEIELQAQHSMRNSLESTLAETEARYSSQLAQ
MQCLISNVEAQLSEIRCDLERQNQEYQVLLDVKARLEGEIATYRHLLEGEDCKLPPQPCATACKPVIRVP
SVPPVPCVPSVPCTPAPQVGTQIRTITEEIRDGKVISSREHVQSRPL

>Hs_K37
MTSFYSTSSCPLGCTMAPGARNVFVSPIDVGCQPVAEANAASMCLLANVAHANRVRVGSTPLGRPSLCLP
PTSHTACPLPGTCHIPGNIGICGAYGKNTLNGHEKETMKFLNDRLANYLEKVRQLEQENAELETTLLERS
KCHESTVCPDYQSYFRTIEELQQKILCSKAENARLIVQIDNAKLAADDFRIKLESERSLHQLVEADKCGT
QKLLDDATLAKADLEAQQESLKEEQLSLKSNHEQEVKILRSQLGEKFRIELDIEPTIDLNRVLGEMRAQY
EAMVETNHQDVEQWFQAQSEGISLQAMSCSEELQCCQSEILELRCTVNALEVERQAQHTLKDCLQNSLCE
AEDRYGTELAQMQSLISNLEEQLSEIRADLERQNQEYQVLLDVKARLENEIATYRNLLESEDCKLPCNPC
STPASCTSCPSCGPVTGGSPSGHGASMGR

>Hs_K38
MTSSYSSSSCPLGCTMAPGARNVSVSPIDIGCQPGAEANIAPMCLLANVAHANRVRVGSTPLGRPSLCLP
PTCHTACPLPGTCHIPGNIGICGAYGENTLNGHEKETMQFLNDRLANYLEKVRQLEQENAELEATLLERS
KCHESTVCPDYQSYFHTIEELQQKILCSKAENARLIVQIDNAKLAADDFRIKLESERSLRQLVEADKCGT
QKLLDDATLAKADLEAQQESLKEEQLSLKSNHEQEVKILRSQLGEKLRIELDIEPTIDLNRVLGEMRAQY
EAMLETNRQDVEQWFQAQSEGISLQDMSCSEELQCCQSEILELRCTVNALEVERQAQHTLKDCLQNSLCE
AEDRFGTELAQMQSLISNVEEQLSEIRADLERQNQEYQVLLDVKTRLENEIATYRNLLESEDCKLPCNPC
STSPSCVTAPCAPRPSCGPCTTCGPTCGASTTGSRF

>Hs_K39
MDTKGCTTTNSPSTPCQNCSRITNVSTISSNNGCHPGGLTVNNCQPAGHVLRIPWDQGCQPTPRFCRKPI
YLMNNFNARFSLDDCSWYGEGINSNEKETMQILNERLANYLQKVRMLERENAELESKIQEESNKELPVLC
PDYLSYYTTIEELQQKILCTKAENSRLVSQIDNTKLTADDLRAKYEAEVSLRQLVESDANGLKQILNVLT
LGKADLEAQVQSLKEELLCLKNNHKEEINSLQCQLGERLDIEVTAAPSADLNQVLQEMRCQYEPIMETNR
KDVEQWFNTQIEELNQQVVTSSQQQQCCQKEIIELRRSVNTLEVELQAQHRMRDSQECILTETEARYTAL
LTQIQSLIDNLEAQLAEIRCALERQNQEYEILLDVKSRLECEITTYRSLLESSDGKRPCYPRATKCEPSP
WTSCKSGAIESTAPACTSSSPCSLKEHCSACGPLSRILVKICTITKEIKDGKVISSYEHVQPCFIIRPAK
V

>Hs_KRT40
MTSDCSSTHCSPESCGTASGCAPASSCSVETACLPGTCATSRCQTPSFLSRSRGLTGCLLPCYFTGSCNS
PCLVGNCAWCEDGVFTSNEKETMQFLNDRLASYLEKVRSLEETNAELESRIQEQCEQDIPMVCPDYQRYF
NTIEDLQQKILCTKAENSRLAVQLDNCKLATDDFKSKYESELSLRQLLEADISSLHGILEELTLCKSDLE
AHVESLKEDLLCLKKNHEEEVNLLREQLGDRLSVELDTAPTLDLNRVLDEMRCQCETVLANNRREAEEWL
AVQTEELNQQQLSSAEQLQGCQMEILELKRTASALEIELQAQQSLTESLECTVAETEAQYSSQLAQIQCL
IDNLENQLAEIRCDLERQNQEYQVLLDVKARLEGEINTYWGLLDSEDSRLSCSPCSTTCTSSNTCEPCSA
YVICTVENCCL

>Bt_K9

MSCRQFSSSSRSHRSGGGGGGCGGSFKSSSRFSSLGAGGGGSRFSSTSSYVVGSSGACGRGGSGSLSSSY
GRGSGGGFSVGSFTGGLGGCIGGAVGGSGGLWGFGGGIGSAVGGFGGAVGGGDAGILPADEKTTMQDLNS
RLASYLDKVQKLEKENTDLETKIRDWYDRQGPKNVKKDYSCYYDTIEDLKNQIVQIMVENNKILVDIDNS
RMTMDDFRVKFEMEQSLRQAVEADINGLRKVLDDLTMQKSDLEMQCESLQEELVALKKNHEEEMSQLCGQ
STGDVSVEMNATPGVDLTKILNEMREDYEKLSAKNRSDIEQQYETQMRQIEQEVTTCSQEVESSNKEVTK
LRHTVQELEIELQSQFSMKSALEKSLEDTENRYSGQLQQIQGQISILEGQLTDIRAEIECQNQEYSLLLS
TKMRLEQEIKTYCSLLEGGQEDFESHGGGQTGFGNSKRSGGGSSYGRGSRGGSGGSCGGGSGGSSGGGSS
SGGGSGGSCEEGSGRTSQSQSYSSKSGGCDTQGHQTRY

>Bt_K10-c
MSVRYSSSKQYSSSRSGGGGGGGGSSLRISSSKGSLGGGYSSGGFSGGSFSRGSSAGGCFGGSSSIYGGGL
GSGFGGGYGSSFGGSYGGSFGGGYGGGGFGGGSFGGGSFGGGLGGGFGDGGLISGNQKITMQNLNDRLAS
YLDKVRALEESNYELEVKIKEWYEKYGNSRQREPRDYSKYYQTIDDLKNQIFNLTTDNANILIQVDNARL
AADDFRLKYENEVTLRQSVEADINGLRRVLDELTLTKTDLEMQIESLTEELAYLKKNHEEEMRDLQNVST
GDVNVEMNAAPGVDLTELLNNMRSQYEQLAEKNRRDAEAWFNEKSKELTTEINSNLEQVSSHKSEITELR
RTIQGLEIELQSQLALKQSLEASLAETEGRYCVQLSQIQSQISSLEEQLQQIRAETECQNAEYQQLLDIK
IRLENEIQTYRSLLEGEGSSGGGGRGGGSFGGGYGGGSSGGGSSGGGHGGGHGGSSGGGYGGGSSGGGSS
GGGYGGGSSSGGHGGSSSGGYGGGSSGGGGGGYGGGSSGGGSSSGGGYGGGSSSGGHKSSSSGSVGESSS
KGPRSAETSWDTNKTRVIKTIIEELAPDGRVLSSMVESETKKHYY

>Bt_K12
MSLSVRTSGLPQWLSSQSGTLGRARGMSASSIGSGYGGSAFGFGGNSGGGFSAASMFGSSSGFGSSSGFG
GACGSSFAGGLGAGYGGAGGGGFGGLGIGFGGSSGGGSLGILPGIDGGLISGSEKETMKNLNDRLASYLD
KVRALEEANTDLETKIREWYETRGSGTGDPGSQNDYSKYYPLIEDLRNKIISANIENAQLILQIDNARLA
ADDFRMKYENELALCQNVEADINGLRRVLDELTLARADLETQIETLNEELAYLKKNHEEELQSCRAGGPG
QVSVEMDAAPGVDLTRLLNDMRAQYETIAEQNRKDAEAWFIEKSGELRKEISSNTEQLQSSKSEVTDLRR
ALQNLEIELQSQLAMKKSLEDSLAETEGDYCGQLSQVQQLIGSLEEQLLQVRADAERQSADYQRLLNVKA
RLELEIETYRRLLDGEAQGDSLVESSYVTASTSQAPSTDSSQDPNKARKIKTIVQEVVNGEVVSSQVKEI
EELM

>Bt_K13
MSCRLQSSSASYGGGFGGGSCQLGGGRSISTCSTRFVSGGSAGGFGGGVSCGFGGGAGSGYGGGFGGSFG
GGFGGGFGGGFGGGFGDFGGGDGGLLSGNEKITMQNLNDRLASYLEKVRALEEANADLEVKIRDWHLKQT
PTSPERDYSPYFKTIDELRDKILAATIDNNRIILEIDNARLAADDFRLKYENELTLRQSVEADINGLRRV
LDELTLTKTDLEMQIESLNEELAYLKKNHEEEMKEFSKQMVGQVNVEMDATPGIDLTRVLAEMREQYEAM
AEKNRRDAEEWFHSKSAELNKEVTSSTALIQTSKTEITELRRTLQGLEIELQSQLSMKAGLESTLAETEC
RYALQLQQIQGLISSIEAQLSELRSEMECQNQEYKMLLDIKTRLEQEIATYRSLLEGQDSKLIGFTTGGN
SGGNSSRRVPESRP

>Bt_K14
MTTCSRQYTSSSSIKSSGGIGGGSSRISSVLAGGSCRAPSAYGGLSVSSSRYSSGGVCGLGGGYGGGFSS
SSSFGGALGSGFGGGYGGGLGAGFGGGFGGGVGGGFGGGFGVGDGLLAGSEKVTMQNLNDRLASYLDKVR
ALEEANADLEVKIRDWYQRQRPAEIKDYSPYFKTIEDLRNKILTATVDNANVVLQIDNARLAADDFRTKY
ETELNLRLSVEADINGLRRVLDELTLARADLEMQIESLKEELAYLRKNHEEEMNSLRGQVGGDVNVEMDA
APGVDLSRILNEMRDQYEKMAEKNRKDAEDWFFSKTEELNREVATNSELVQSGKSEISELRRTLQNLEIE
LQSQLSMKASLENSLEETKGRYCMQLAQIQELISSVEEQLAQLRCEMEQQNQEYKILLDVKTRLEQEIAT
YRRLLEGEDAHLSSSQFSSGSQSSRDVSSSRQVRTKVVDVHDGKVVSTHEQIVRTKN

>Bt_K15
MATTFLQTSSSTFGGGSTRGGSLLAGGGGFGGGSLYGGGGSRTISASSARFVSSGSAGGYGGGFGGGAGS
GYGGGFGGGFGGGFGSGFGDFGGGDGGLLSGNEKITMQNLNDRLASYLEKVRALEEANADLEVKIRDWYQ
RQSPTSPERDYSPYFKTIDELRDKILAAAIKNSQVILEIDNARLAADDFRLKHENEMALRQSVEADINGL
RRVLDELTLTKTDLEMQIESLNEELAYLKKNHEEEMKEFSNQLAGQVNVEMDAAPAVDLTRVLSEMREQY
EAMAEKNRRDAEAWFFSKTEELNKEVASNTEMIQTSKTEITDLRRTIQGLEIELQSQLSMKAGLESTLAE
TECRYAAQLQQIQGLISSIEAQLSELRSEMECQNQEYKMLLDIKTRLEQEIATYRSLLEGQDSRMAGIGT
REASLGGGGGGKVRINVEESVDGKVVSSRKREI

>Bt_K17
MTTTIRHFSSGSIKGSSGLAGGSSRSCRVSGSLGGGSCRLGSAGGLGSGLGGSSYSSCYSFGSGGSYGSG
GYVSGGYGGGFGGVDGLLVGGEKATMQNLNDRLASYLDKVRALEEANTELELKIRDWYQKQAPGPAPDYS
SYFKTIEDLRNKIHTATVDNANLLLQIDNARLAADDFRTKFETEQALRVSVEADINGLRRVLDELTLARA
DLEMQIENLKEELAYLRKNHEEEMKALRGQVGGEINVEMDAAPGVDLSRILNEMRDQYEKMAEKNRKDAE
DWFFSKTEELNREVATNSELVQSGKSEISELRRTLQALEIELQSQLSMKASLEGSLAETENRYCMQLSQI
QGLIGSVEEQLAQLRCEMEQQNQEYKILLDVKTRLEQEIATYRRLLEGEDAHLTQYKTKEPVTTRQVRTI
VEEVQDGRVISSREQVHQTSH

>Bt_K18
MSFSTQSTFSNYRSLGSVQSSGHRVRPVSSAASVYAGAGGSGSRISVSRTTSVRGGWGSGNLGAGMAGGL
VGVGGIQGEKETMQDLNDRLASYLEKVRSLEADNRRLESKIREHLEKKGPQVRDWAHYLKIIEDLRAQIF
ANSVDNARIVLQIDNARLAADDFRVKYETELAMRQSVESDIHGLRKVIDDTNVTRLQLETEIEALKEELL
FMKKNHEEEVKGLQNQIANSGLTVELDAPKPQDLSKIMADIRAQYDELAQKNREELDKYWSQQIEESTTV
VTSQTAEIGAAEMTLTELRRTVQSLEIDLDSMRNLKASLENSLREVEARYAMQMEQLNGVLLHLESELAQ
TRAEGQRQTQEYEALLNVKVKLEAEINTYRRLLEDGEDFSLGDALDSSNSRQTIQKTTTLRLVDGKVVSE
TSDTKVLRH

>Bt_K19
MTSYSYRQSSSTSSFGGMGGGSMRFGAGGAFRAPSIHGGSGGRGVSVSSARFVSSSSGGYGGGYGGALAT
SDGLLAGNEKLTMQNLNDRLASYLEKVRALEEANGDLEVKIRDWYQKQGPGPARDYSHYFKTIEDLRDQI
LGATIENSKIVLQIDNARLAADDFRTKFETEQALRMSVEADINGLRRVLDELTLARTDLEMQIEGLKEEL
AYLKKNHEEEMSVLKGQVGGQVSVEVDSAPGIDLAKILSDMRSQYEVIAEKNRKDAEAWFISQTEELNRE

VAGHTEQLQISKTEVTDLRRTLQGLEIELQSQLSMKAALEGTLAETEARFGAQLAQIQALISGIEAQLSD
VRADTERQNQEYQHLMDIKTRLEQEIATYRNLLEGQDAYFNDLSLAKAL

>Bt_K20
MSSSIYRRGVAPSVYGGAGGHGTRISTSRHLANYGSDPAGGNLFAGNEKMTMQNLNDRLASYLERVRSLE
QSNSHLEQQIKHWYETNTPSTGRDHSAYMGQIKELRDQIKDAQLQNARCVLQIDNAKLAMEDFRLKYEAE
RGICQTVVADLHGLKRVFDELTLTKADLEIQIEELTKDLHLLQKEHEEEVRSLRAHLGNNVNVEVDAPPS
LNLGAIMNEMRQKYDAMAQENLQKAKEQFEIQVIALQQQVTVSTEELKGTKDQIKEQRHTYQVLELELQS
LLNMKEALEHTLEETNARYGSHLAKIQARLNSLEGQLVQVRTDTERQIHEYNILLDIKIRLEQEIATYRR
LLEGEDVKTREYQLSTLDEKDIKKTRKIKTVVQEVVDG

>Bt_K23
MNSNQSFSQKRSGSLYATGGSWGRPGSFLRAPSVHGGAGGVRISLSFSSPSCPLPEGSWRSGRGSSLLGG
NEKEMMQNLNDRLATYLEKVRALEEANVKLESCILKWHQQRDSGNKQDYSQYEESISHLQEQIADGKVNN
AQIILLIDNARMAVDDFSLKYENEHSFKKDLEIEVEGLRKTLDDLTIVTTDLEQEVEGMRKELILMKKRH
EQEVEEHRVPNDFKVNVKVETTPGEDLIKVLEDMRQEYEFIIKKKHQDLDTWYKQQSAAVAQEAASPTGV
QSSQSDLHELKRTFQALEIDLQAQCSKKSALENMLSETQSRYSCQLQDMQQIISHYEEELTQLRHDLERQ
NNEYKVLLGIKTHLEKEIATYRQLLEGENGGKMEESKSNVKAPKIKAITQESVNGRIILSQVNEI

>Bt_K24
MSCSSRVSSSRAGGSSSVRVSAGGSSFSSGSRIGLGGGSARGFRGGAGSYGLSGGSSGGFGGGFGSCSVG
GGFGGASGSGIGFGGGSSFGGGSSFGGGSSFGGGSSFGGGSGFGGSAGYAGGASGGFYSYAGGVGGGVGG
GLGDGGLFSGGEKQAMQNLNDRLANYLDKVRALEEANADLENKIKEWYEKFGPRSGDGGAGRDYSKYYPV
IEDVRTQIITATIENAGIVLQIDNARLAADDFRLKYENELHLRQTVEADINGLRKVLDDLTMTRSDLEMQ
IESLTEELAYLKKNHEEEMKSMQGSSRGDVTVEMNAAPGTDLTKLLNDMRAQYEELAEQNRREAEEQFNK
QSASLQAQISTDAGAANSAKNEITELKRTLQALEIELQSQLAMKSSLEGTLADTEANYMVQLSQIQMQIS
SLEEQICQIRGETECQNAEYEQLLDIKTRLEMEIETYRRLLDGEGGGSGFGGSDYRRSGSRNMGSRDSSM
SDGSRSGTSVQGRDPSKSRVTKTIVEEVVDGKVISSQVSNVSEVKLK

>Bt_K25
MSLRLPSGSRRASPRPTTGSLRLSSGGASFGAGNACSMPGIGSSFSCAFGGSSSGGNALGGNPCAGFTVN
EGGLLSGNEKVTMQNLNDRLASYLENVRALEEANADLEQKIKGWYEKFGPGSCRGLDHDYSRYFPIIEDL
KNQIIASTTSNANAVLQIDNARLTADDFRLKYENELALHQSVESDVNGLRRVLDEITLCRTDLEIQYETL
SEELTYLKKNHKEEMQVLQCAAGGNVNVEMNAAPGVDLTVLLNNMRAEYEALAEQNRRDAEAWFNEKSAS
LQQQITEDVGATTSARNELTEMKRNLQTLEIELQSLLATKHSLECSLTETEGNYCAQLAQIQAQIGALEE
QLHQVRTETEGQKLEYEQLLDIKVHLEKEIETYCLLIGGDDGACKSGGYKSKDYGAGNVGNQMKDPVKAI
VVKKVLEEVDQRSKILTPRLHSLEEKSQSN

>Bt_K26
MSFRLSSGSRRLCSPAGSGQLTGGRTGFRAGNACGGLGAGSSFSGPLGSVSSRGSFSHGGGGLGSGVCTG
FLENEHGLLPGNEKVTLQNLNDRLASYLDHVCTLEEANADLEQKIKGWYEKYGPGSGRQLAYDCSKYFSV
TEDLKRQIISVTTCNASIALQNENARLTADDFRLKYENELALNQSVEADINGLHRVMEELTLCTSDLEIQ
CEALSEELTCLKKNHQEEMKVMQGAAGGNVNVEINAAPGVDLTVLLNNMRAEYEDLAEQNREDAEAWFNE
KSTSLHQQISDDAGAATAARNELMELKRNLQTLEIELQSLMAMKHSYECSLAETESNYCHQLQQIQEQIG
ATEDQLQQIRMETEGQKLEHERLLDVKIFLEKEIEMYCKLIDGEGRKSKSTYCKSEGRGPKNSENQVKDS
KEEAVVKTVVGELDQLGSVLSLRVHSVEEKSSKISNITMEQRLPSKVPQ

>Bt_K27
MSVRFSSASRRLGSCGGAGSVRLSGGGAGFGVGSTGSVPGFGSGFTCAFGGSSSAGSYSGGLGGGSASCT
AFTGNEHGLLSGNEKVTMQNLNDRLASYLDNVRALEEANADLEQKIKGWYEKFGPGSCRGLDHDYSRYFT
VIDDLRNQIISATTSNANIVLQNDNARLTADDFRLKFENEQALHQSVDADVSSLRRVLDELTLCRTDLEI
QLETLSEELAYLKKNHEEEMKALQCAAGGNVNVEMNAAPGVDLTVLLNNMRAEYEALAEQNRRDAEAWFN
EKSASLQQQISDDAGATTSARNELTEMKRNLQTLEIELQSLLATKHSLECSLTETEGNYCAQLAQIQAQI
GALEEQLHQVRTETEGQKLEYEQLLDIKVHLEKEIETYCRLIDGEDGSCAKSKGYGGPGHQIKDPSKATV
VKTIVEEIDPRGKVLSSRVHSVEEKSTKVNNVKSEQRVPS

>Bt_K28
MSLRFSSGSRHICLRSGTESVRPSSGGTGFAGSNVYGNSGAGCGFSYALGGGLGSLPGGDHAGGIPGSGT
CVGFAGSEGGLFSGNEKVTMQNLNDRLASYLDNVRALEEANAELERKIKSWYEKHGPGSCHGLDHDYSRY
HLTIEDLKNKIISSTTANANVILQIDNARLAADDFRLKYENELALHQNTEADINGLRRVLDELTLCRTDQ
ELQYESLSEEMTYLKKNHEEEVKALQCVAGGNVNVEMNAAPGVDLTLLLNNMRAEYEDLAEQNRRDAEAW
FNEKSASLQQQISDDAGAASSARGELTEMKRTVQTLDIELQSLLATKHSLECSLMETEGNYCAQLAQIQA
QIGALEEQLHQVRTETEGQKLEYEQLLDIKVHLEKEIETYCRLIDGDRNSCSKSKGFGSGSPGNSSKDLS
RTTLVKTVVEEIDQRGKVLSSRVQSIEEKTSKMTNGKTKQRVPF

>Bt_K31
MSYNFCLPNLSFRSSCSSRPCVPPSCCGTTLPGACNIPANVGSCNWFCEGSFNGNEKETMQFLNDRLASY
LEKVRQLERENAELESRILERSQQQEPLLCPNYQSYFRTIEELQQKILCAKSENARLVVQIDNAKLAADD
FRTKYETELGLRQLVESDINGLRRILDELTLCKSDLEAQVESLKEELICLKSNHEEEVNTLRSQLGDRLN
VEVDAAPTVDLNRVLNETRAQYEALVETNRRDVEEWYIRQTEELNKQVVSSSEQLQSCQTEIIELRRTVN
ALEVELQAQHNLRDSLENTLTETEARYSCQLAQVQGLIGNVESQLAEIRSDLERQNQEYQVLLDVRARLE
CEINTYRGLLDSEDCKLPCNPCATTNACGKTITPCISSPCTPCAPAAPCTPCVPRSRCGPCNSYVR

>Bt_K32
MSCQPEWYLGYICQPGMCVPSLSVSTTYYPTSSIFSSMGSNSLFYESVFSSNERETLQLLNNRLAAYLER
VRQLEQENVELERQLQEACEFQEPTVGPNYFRYFQIIEELQQKILYSKAENARIVVQIDNTKLAADDFRS
KYEMELGLRQLVEADTNGLRRILDELTLCKADLEMQVESLKEELMCLKKNHEEEVGALRCQLGDRLNIEV
DAVSPVDLNKVLEEMRCRYEALVETNRRDVEEWFNKQMEELNQQVVTSSEQLQSYQSDIIDLKRTVNTLE
IELQTQHSLRDSLENTLTETEARYGSQLAQMQGLVTNVESQLAEIRCDLERQNQEYRVLLDVRARLEAEI
NTYRGLLDSEDCKLPGNPCSTPSQPPCAPAPSVPRTLCVPRTVCVPCVPCHPSCH

>Bt_K33b
MSYSCCLPNLSFRSSCSSRPCVPSSCCGTTLPGACNIPANVGSCNWFCEGSFNGSEKETMQFLNDRLASY
LEKVRQLERDNAELESRILERSQQQEPLVCPNYQSYFRTIEELQQKILCSKSENARLVVQIDNAKLASDD
FRTKYETEVSLRQLVEADLNGLRRILDELTLCKSDLEARVESLKEELICLKQNHEQEVNTLRSQLGDRLN
VEVDAAPTVDLNRVLNETRAQYEALVETNRRDVEEWYIRQTEELNKQVVSSSEQLQSYQAEIIELRRTVN
ALEVELQAQHNLRDSLENTLTETEARYSCQLAQVQGLIGNVESQLAEIRSDLERQNQEYQVLLDVRARLE
CEINTYRGLLDSEDCKLPCNPCATTNACERPCISNPCVSRARCGPCNTFVH

>Bt_K33a
MSYSCCLPNLSFRSSCSSRPCVPSSCCGTTLPGACNIPANVGSCNWFCEGSFNGSEKETMQFLNDRLASY
LEKVRQLERDNAELESRILERSQQQEPLVCPNYQSYFRTIEELQQKILANKAENARLVVQIDNAKLAADD
FRTKYQTELGLRQLVESDINGLRRILDELTLCKSDLEAQVESLKEELICLKQNHEQEVNTLRSQLGDRLN
VEVDAAPTVDLNRVLNETRAQYEALVETNRRDVEEWYIRQTEELNKQVVSSSEQLQSYQAEIIELRRTVN
ALEVELQAQHNLRDSLENTLTETEARYSCQLAQVQGLIGNVESQLAEIRSDLERQNQEYQVLLDVRARLE
CEINTYRGLLDSEDCKLPCNPCATTNASSVGSCVTNPCTPCGPRSRFGPCNTFGC

>Bt_K34
MPYSCCLPTLSYRSSCSSRPCVPHSCRGTTLPGACNIPANVGSCNWFCEGSFNGNEKETMQFLNDRLASY
LEKVRQLERDNAELESRILERSQQQEPLLCPNYQSYFRTIEELQQKILCAKSENSRLVIQIDNAKLAADD
FRTKYETERSLRQLVESDINSLRRILDELTLCKADLEAQVESLKEELLCLKKNHEEEANSLRSQLGDRLN
VEVDAAPTVDLNRVLNETRAQYEALVETNRRDVEEWYIRQTEELNKQVVSSSEQLQSYQAEIIELRRMVN
ALEVELQAQHNLRDSLENTLTETEARYSCQLAQVQGLIGNVESQLAEIRSDLERQNQEYQVLLDVRARLE
CEINTYRGLLDSEDSKLPCNPCATTNASSISCRSSAQNRCC

>Bt_K35
MASKCLKASFSSGSLKVPGGAGGGSARVSTIFSSSSCKLPSFSRGPRSFSACSVGLGKSSCRAASCLPAL
CLPSGGFATSYGMAGGWFGEGILTGNEKETMQFLNDRLASYLEKVRQLERENAELESRIHEWCEQQVPYL
CPDYQSYFQTIEELQKKTLCTKSENARLVVQIDNAKLAADDFRTKYETEVSMRQLVESDMNGLRRILDDL
TLCKADLEAQVESLKEELLCLKKNHEEEVNSLRCQLGDRLNVEVDAAPPVDLNRVLNEMRCQYETLVENN
RREAEDWFNTQTEELNQQVVSSSEQLQSYQAEIIELRRTVNALEIELQAQHSMRDALESTLAETEARYSS
QLAQMQGLIGNVESQLAEIRCDLERQNQEYQVLLDVRARLECEINTYRGLLDSEDCKLPCNPCAPDHSPS
KSCLPCLPAASCGPGMARTTCSPRPICVPCPGSRF

>Bt_K36
MASQFCSPIFSSGSIRGHCGATSGISRVSCVRSVGSCRGPSLAGSASSVRLGLSSLGSCLPGSFLSSGFH
SSGFAGAGGWFCEGSFNGNEKETMQFLNDRLASYLEKVRQLERENAELESRIREWYESQAPYICPDYQCY
FKTIEELQQKILLTKADNARLVLQIDNAKLAADDFRTKYETELGMRQLVEADTNGLRQILDELTLCKADL
EMQVESLKEELICLKKNHEEEVNTLRCQLGDRLNVEVDAAPPVDLNKILDEMRCQYETLVENNRRDVEAW
FNTQTEELNQQVVSSSEQLQCCQTEIIELRRNVNALEIELQAQHSMRNSLESTLAETEARYSSQLAQMQG
LIGNVEAQLAEIRCDLERQNQEYQVLLDVKARLESEIATYRRLLEGEDCKLPAHPCATECKPAVRVPYVS
TGPCAPAPQLSTQIRTITEEIRDGKIISSREHVQPL

>Bt_K37*
MLPFWDSRPPWHMPSRVRVGTTPLGQPSLCLPHSCNTACPLPGTCDIPGNIGICGTFSEGFFNSHEKKTM
QFLNDRLASYLEKVRRLERDNAELESQIRELSKCPESTVCPDYQSHFRIIEELQQKILCSKAENTRLIIQ
VDNAKLAADDFRIKXESERSLRMVVEADLFGLHKILDDMSLAKADLEAQQESLKEEQLCLKSNHEQEVNT
LKCQLGDKLKIELDVVPTVDLGRVLEEMRGQYEAMVETSLRDVEQWFQTQSEGISLQAMSCSEELQCCQS
EILELRRTVNALEVELQAQHNLKDCLQNSLCESEACFGTELVNVEEQLSEIRADLEQQNQEYQVLLDVKA
RLECEINTYQNLLESEDCRLPCNPCSTPAS

>Bt_K38
MTSISCSSLLSGPLSQAGAAPLGLSATLAHANRVRVGTTPLGRPSLCLRHSCNAACPLPGTCDIPGNSGI
CGTFGEGFFNSHEKETMQFLNDRLASYLERVRRLERDNAELESQIRELSKCPESTVCPDYQSHFRIIEEL
QQKILCVKSENNKLVVQIDNAKLAADDFRTKFQTEHSLRQLVEADVCGMRRALDDLTLAKADLEAQLESR
KEELLCLKKNHEQEVHTLKCQLGDKLKIELDVEPTVDLGRVLEEMRGQYEAMVETNLRDVEQWFQTQSEG
ISLQAMSCSEELQCCQSEILELRRTVNALEVELQAQHTLKDCLQNSLCESEARFGTELAQMQSLISNIEE
QLSEIRADLERQNQEYQVLLDVKARLECEIATYRNLLENEDCKLPCNPCATPVSSTCVGSSACTSCYPCL
SGPAGSCSSPRC

>Bt_K39
MSSATPCQSCSGITNLTTILPNTSCQHGGLKANSCQPTGHDLRTLQSQDDEPTPCFSLTPLCLISNFHTC
PFLDDCGWCDGSINSNEKETMKILNNRLTKYLEKVRMLERENAALECKIQEECNKELPVICPDYLSYYAT
IEELQQKILCTKAENSRLVSQIDNTKLTADDVRAKYEAEVSLRQLVEADANGLQQILNALTLGKADLEAQ
VQSLKEELLCLKNDHEQEISSLQSQLGDRLNIEVTSAPSVDLNRVLREMRCQYESIMETNRKDVEEWFNT
QMEELNQQVVTSTQQQQCYQKEIIELRRTMNALEIELHAQHRMRDSQECALTETEARYAALLTQIQCLID
NLEAQLAEIRGALERQNREYEILLDVKSRLECEIATYHSLLESSDGKLPCHPCAIKHEPSACISSKARTM
ECTAPVHTSSLGPCGIHESRSACSILPRILVKICTITEEIKDGKVISSHKHVQPCFITRTAKA

>Bt_K40
MASDCSPTGCSSESSARASDCALASTCSVETTCLPSACATSSCQTPSFPSGARLPTGCLPPACFAGSCNI
PCVVGNCAWCEDGVFNSNEKETMQFLNDRLASYLEKVRGLEELNAELECRIREQCEEDVPLVCPDYQCYF
DTIEDLQQKILCTKAENCRLAVQLDNCKLAADDFRSKYESELSLRQLVETDISGLRGILGELTVCRSDLE
AHVESLKDDLLCLKKSHEEEVNVLRGQLGDRLSVELDTAPTTDLNRVLDEMRCQYETVLANNRRDVEEWF
AAQTEELNQQQLSSAEQLQGCQTEILELKRTANTLEIELQAQQSLTESLECTVAETEAQYSSELAQIQCL
IDNVENQLAEIRCDLERQNQEYRVLLDTKARLECEINTYQGLLDSEDSRLPCNPCSATSMSNDTCEPCSA
YVICTVENSCP

>Bt_K42

MAATTTSIRQFSTSGSVKGLCMPSGGFSRMSSIRVGGACRAPSLLGVGGSGNMSMSSSRFSMGLGGGYGG
GYTCNLGGGFGSSFGTVDNLLGGSEKETMQNLNDRLASYLDKVRALEEANADLEVKIHDWYKKQGPGPAR
DYSHYFKTIEELRSKILAATIDNASLVLQIDNARLAADDFRTKYETELNLRMSVEADINGLRRVLDELTL
ARADLEMQIENLKEELAYLRKNHEEEMNALRGQVGGDVNVEMDAAPGVDLSRILNEMRDQYEKMAEKNRK
DAEDWFFSKTEELNREVATNTEALQSSRTEITELRRTVQNLEIELQSQLSMKASLEGSLAETEARYGAQL
AQLQGLISSIEAQLSELRCDMERQNHEYQVLLDVKTRLEQEISTYRRLLEGEDAHLSTQYSSSLASQPTR
EGTVTTRQVRTIMEEVQDGKVVSSREQVHRSTH

# B

>Bas_K4-p
MLIRQQCGRGGPRGFSCGSAVVGGGKKSAFSSTSMSGGTGCFSSGGFSSRSLCNLGGNKSISIGTAGCWR
GTGFGAAGGFGAAGGFGAAGGFGAGGFGPGFGGSSGGWGGAGFPVCPPGGIQEVTINQSLLTPLHMEIDP
EIQKVRTEEREQIKTLNDRFVSFIDKVRFLEQQNKVLETKWNLLQQQTTTTSSKNLDPFFEAYLSALRKQ
LDNLTNNKGRLQCELKIMQDSVEDFKAKYEDEINKRTAAENDFVVLKKDVDATYMSKTELEAKVDARNDE
INFLRVLYAAELDQMQTHVSDTSVILSMDNTRDLNLDGIIAEVRAQYEEIAQRSKAEAEALYQTKVQQLQ
ASVEQHGDSLRNTKNEISELNRMIQRLRAEIENVKKQVCTLQTSVADAEQRGEVALKDAYSKRTELEVAL
QKAKEELARMLHEYQELMSVKLALDVEIATYRKLLEGEECRXXXXXXXXXXXXXXXXXXXXXXXXXXXXX
XXXXXXXXXXXXXXXXXXXXXXXXXXXXXXXXXXXXXXXXXX

>Bas_K5
MSRQSSVSFRSGGSRSFSTASAITPSVSRTSFTSVSRSGGGGGGFGRVGPGGAYGAGGYGSRSLYNLGGS
KRISIGSSGGGFRNRVCATGAGGSYGFGGGTGSGFGFGSGAGGGFGFGGGSGFGGGYGGPGMAVCPPGGI
QEVTINQSLLTPLNLQIDPTIQRVRTEEREQIKTLNNRFASFIDKVRFLEQQNKVLDTKWTLLQEQGTRT
VKQNLEPLFEQYINNLRRQLDGILGERGRLDSELRNMQDLVEDFKNKYEDEINKRTTAENEFVMLKKDVD
AAYMNKVELEAKVDALMDEINFLKMFFEAELSQMQTHISDTSVVLSMDNNRCLDLDSIIAEVKAQYEEIA
NRSRTEAESWYQTKYEELQQTAGRHDDDLRNTKHEISEMNRMIQRLRSDIDNVKKQCANLQNAIADAEQR
GELALKDARNKLTELEDALQKAKQDMARLLKDYQELMNTKLSLDVEIATYRKLLEGEECRLSGEGVGPVN
ISFVTNTVSSAYGSGGSFGGGFGSGLGGGLGGGLGSGLGGGSGGFYSSSSGGVGLGSGLGVGGSGFSASS
GRSLGFGSGGGSSSNVKFVSTTSSSRKSFKS

>Bas_K6-l2
MTSKSTVKSQSSSRRVFSAGSARVPGVSRSGFSSVPVCRSRGSGGLAGVGGGAGFGSRSLYGVGGSKRIS
LGGGSCALGGGYGGSAGGGYSVGGGAGSGFGFGSGAGGGFGLGGGAGFGIGYWGPGMPVCPPGGIQEVTI
NQSLLTPLNLQIDPTIQRIKTEEREQIKTLNNRFASFIDKVRFLEQQNKVLETKWTLLQEQGTKTVRHNL
EPLFEQYINNLRRQLDSLVTERSRLDSEFRGMQDTVEDFKKKYEDEINRRMSAEHEFVNLKKDVDTAYMN
KVELETKADALIDEINFLRALYEAELAQMQTHISDTSVVLSMDNNRTLDLDSIIAEVKAQYEVIACRSRE
EAECWYKCKYEELQLTAGRHGDDLRNTKQEITEINRMIQRLRSEIDHVKKQCANLQSAIADAEQRGELAL
RDAKNKLAELENALQKAKQDMAQLVKDYQELMNVKLSLDVEIATYRKLLEGEECRLNGEGVGQVNISVVQ
STVSSGYGGAGGVSGGLGLGGGSGYSYSSGHGIGGGFSSGGGFSSGSGRAIGGGLSSSGGSSSAIKYTTT
SSSSRKGYRH

>Bas_K6-l3
MTSKSTVKSQSSSRRVFSAGSARVPGVSRSGFSSVPVCRSRGSGGLAGVGGGAGFGSRSLYGVGGSKRIS
LGGGSCALGGGYGGSAGGGYSVGGGAGSGFGFGSGAGGGFGLGGGAGFGIGYWGPGMPVCPPGGIQEVTI
NQSLLTPLNLQIDPTIQRIKTEEREQIKTLNNRFASFIDKVRFLEQQNKVLETKWTLLQEQGTKTVRQSL
EPLFEQYIYNLRSQLDSLVTERSRLDSEFRGMQDMVEDFKKKYEDEINRRMSAEHEFVNLKKDVDTAYMN
KVELETKADALIDEINFLRALYEAELAQMQTHISDTSVVLSMDNNRTLDLDSIIAEVKAQYEVIACRSRE
EAECWYKCKYEELQLTAGRHGDDLRNTKQEITEINRMIQRLRSEIDHVKKQCANLQSAIADAEQRGELAL
RDAKNKLAELENALQKAKQDMAQLVKDYQELMNVKLSLDVEIATYRKLLEGEECRLNGEGVGQVNISVVQ
STVSSGYGGAGGVSGGLGLGGGSGYSYSSGHGIGGGFSSGGGFSSGSGRAIGGGLSSSGGSSSAIKYTTT
SSSSRKGYRH

>Bas_K6-l4
MTSKSTVKSQSSSRRVFSAGSARVPGVSRSGFSSVPVCRSRGSGGLAGVGGGAGFGSRSLYGVGGSKRIS
LGGGSCALGGGYGGSAGGGYSVGGGAGSGFGFGSGAGGGFGLGGGAGFGIGYWGPGMPVCPPGGIQEVTI
NQSLLTPLNLQIDPTIQRIKTEEREQIKTLNNRFASFIDKVRFLEQQNKVLETKWTLLQEQGTKTVRQSL
EPLFEQYIYNLRSQLDSLVTERSRLDSEFRGMQDTVEDFKKKYEDEINRRMSAEHEFVNLKKDVDTAYMN
KVELETKADALIDEINFLRALYEAELAQMQTHISDTSVVLSMDNNRTLDLDSIIAEVKAQYEVIACRSRE
EAECWYKCKYEELQLTAGRHGDDLRNTKQEITEINRMIQRLRSEIDHVKKQCANLQSAIADAEQRGELAL
RDAKNKLAELENALQKAKQDMAQLVKDYQELMNVKLSLDVEIATYRKLLEGEECRLNGEGVGQVNISVVQ
STVSSGYGGAGGVSGGLGLGGGSGYSYSSGHGIGGGFSSGGGFSSGSGRAIGGGLSSSGGSSSAIKYTTT
SSSSRKGYRH

>Bas_K7-h
MSIHFSSQVFSSRSSAFPGRGTQVRLNSVGPGGFGGSSISLYGLGASRPRVAARSSYGGPAGAGIREVTI
NQSLLTPLQVNIDPSIQQVRQEEREQIKTLNNKFASFIDKVQFLEQQNKLLETKWALLQEQKSTQSSCLL
GIFEAQIAGLRKQLEVLQLDGGRLEVELRNTQDVVEDFKNKYEDEINRRTAAENEFVLLKKDVDVAYMSK
VELEAKVDALNDEINFLRTLYEEELKELQSEVSDTSVVLSMDNSRSLDLDGIIAEVKAQYEEITNRSRAE
AEAWYQTKFETLQAQAGKHGDDLRNTRNEIAELNRAVQKLQAEIDSIKKQRAKLEAAIAEAEERGELAV
KDARAKQEELEAALQRAKQDMARQLRDYQELMSTKLALDIEIATYRKLLEGEESWMTGAGVGAVNISVVS
STGGAGSRLTFGGTMGSNALRFSSGGGPGAPKAYSIRTTSTPGRSTRN

>Bas_K8
MSIRVTQKSYKVSTSGPRSFSSRSYTSGPGARISSSAFSRVGSSSSFRGGLGSSMSLAGGYSGAPGLGGL
TAVTVNQSLLSPLKLEVDPDIQAVRTQEKEQIKTLNNKFASFIDKVRHLEQQNKILETKWNLLQQQKTAR
SNIDNMFESYINNLRRQLETLAQEKLKLEVELGNMQGLVEDFKTKYEEEIQKRTDMENEFVIIKKDVDEA
YMNKVELESRLEGLTDEINFYRQLYEEEIREMQSQISDTSVVLSMDNSRSLDLDGIIAEVKAQYEEIANR

SRAEAETMYQTKYEELQTLAGKHGDDLRRTKTEISEMNRNINRLQAEIEGLKGQRASLEAAIADAEQHGE
LAVKDAQAKLAELEAALRTAKQDMARQLREYQELMNVKLALDVEIATYRKLLEGEESRLESGMQNMSIHT
KTTSGYSGGLTSAYGTPGLNYGLSSYQSSLGSGGASGSFSRTSSKAVVVKKIETRDGKLVSESSDILPK

>Bas_K78
MSLSPCRAQRGFSARSACSARSGGRGRVNVSSRSLSSFVGCQGGSRGRAWGSRGRLGVRYGEGIGGPGLS
PCPPGGIQEVAINWNLLTPLKIEIDPQFQVVQTQETQQIRTLNNQFASFIDKVRFLEQQNKVLETKWDLL
QQQELSDSPQALHSFFEAYLVQLRKQLEQLQRERGSLDAELKSCQDQQEEYKTKYEREAHKCATLEKDFV
VLKKDVDGVLSSKMELEGMVEALKEYICFLRHLYEEGLGQLQTQARDMSVVLSMENNRRLDCRDLIAEVC
ARYEEIARTSKAEAEMLYQTKYRELQVSAQLHEDQMKGTKVQITQLQQVIQKLQSQTENLKKQNANLQAA
IAHAEHRGELALKDAQAKLAELEAALRTSKQDMARQLREYQELMSSKLSLDVEIATYCRLLEGEECRMSG
ECASQVTISVGEGSTIVSAGAGGGLVGTCGLGGGNGSFGSGCSSIVTGDSNVILGSGQGPFLGSCAVSGS
GSSSTCHTILKKTVESSLKTSITY

>Bas_K80x1
MACRCCIVGFGNLNSCEVTQASSPWPGTSGWSNYRAPEPGFSSGSLTGCLTASTIPKVTVNPSLLVPLDL
KVDPAIQQQKSQKKEEMKVLNDKFASLIGKVQALEQRNQLLETHWHFLRSRDLAACDLGHLYEEYQDRLQ
EELRKVNQEGAQLEAKLLQELAMVQKFQIRYEDEVSKRTDMEFTAVQLKKDLNAERLRQTQLETKLKGLK
SFAVMMKSIYEQELKDLAAQVKDVSVTVGMGSRCHTDLSGIVEKVMAQYGAVVARSLEEAKAYSRSQVRS
SAACSAESGNRLQRSRSKINDLSARIQKLRSQILSIKSHCLKLEENIKAAENQGELAFQDAKAKLAQLEA
ALQQAKKDMARQLCDYQELMNTKLVLDIEIATYGKLVEGEESRMDLPAATVISCMQSRSRTAASKYGLSR
SPSRKKKNREHPVIKITETSEEFLSQESEVSQ

>Bas_K84-p
MSCRSYTVSSGRRVGSFSSCSAMTPQNLNHFRASSVSCRSGRSFQGLSGFGSRSVIRFGSCSPRIAAVCP
RPIRYGVGFGLGSGMAFGFGDGSGAGLGFGAGSGLGYGFSGPGFGYRVGGAGVPAAPSITAVTVNESLLT
PLSLEIDPNVQRVKKHEKEQIKTLNNKFASFIDKVQFLEQQNKLLETKWNFLQEQKCARSNLEPLFENYI
TNLQRQLDVVNSERARLEAERNSTQDVLEGFKKKYEEEVGLRANAENEFVALKKDVDTVFLNKSDLEANV
DTLTQDIDFLKTLYMAEIQLLQSHISETSVIVKMDNSRDLNFDGIIADIKAQFEEIARRSRADAEAWYQT
KYEKMWVTAGQHCDNLRNTRNEINELTRLIQRLKAEIEHAKAQRCKLEAAVAEAEQRGEAALSDAKCKLA
ELEAALQKAKQDMAWQLKEYQELMNVKLALDIEIATYKRLLEGEEIRIYEGVGPVDISVSRSRGGLVCGP
EPPVTTCSLSRGGVTIXXXGVTISGRSSIRSSGFCGSSVGGAQVVGGGDVLSAGSRGGSVLVGETCAPSV
PCPLPTEGGFSSRGSSVRLVSTTTSRRTKY

>Bas_K85-c-p
MTCRSYRISPGCGVTRTFSSCSAVAPKTGSRCCISAAPYRGVSCYRGPTGFGSRSVSALGSCXXXRVAGG
GFRAGSCGRSFGYRSGGVCGPSPPCITTVSVNESLLVPLNLEIDPNAQCVKHEEKEQIKCLNSRFAAFID
KVRFLEQQNKLLETKWQFYQNRQCSESNLEPLFNGYIETLRREAERVEADNGRLASELNHMQEVLEGYRK
KYEEEVALRATAENEFVVLKKDVDCAYLQKSDLEANVEALVEECSFLKRLYDEELQVLHAHISDTSVLVK
MNNSRDLNMDCFVAEIKAQYDDVASRSRAEAESWYRSKCEEMKATVIRHGETLRRTKEEINELNHMIQSQ
IENAKCQRAKLEAAVAEAEQQGEAALNDARCKLAGLEEALQKAKQDMACLLKEYQEVMNSKLGLDIEIAT
YRRLLEGEEHRLCEGVGSVKVCVSSSRGGVTWGPTCIAPPQAARLPLAPWPLGAASQ

>Bas_K86
MTCGSGFGGRSFSCASACGPRPGRCCVTAAPYRGISCYRGITGGFGSRSLCGGFRAGSCGRSFGYRSGGV
CGPSPPCITTVSVNESLLAPLNLEIDTNAQCVKHEEKEQIKCLNSRFAAFIDKVRFLEQQNKLLETKWQF
YQNRQCSESNLEPLFNGYIETLRREAERVEADNGRLASELNHVQEVLEGYKKKYEGEVNLRATAENEFVT
LKKDVDCAYLCKSDLEANSEALIQEIDFLRQLYEEEIRVLHAHISDTSVIVKMDNSRDLNMDSIVAEIKA
NYDDVASCSRAEAESWYRSKCEEIKATVIQHGETLRRTKEEINELTRMIQRLTAEVENAKCQNSKLEAAV
TQAEQQGEAALNDAKCKLAGLEEALQKAKQDMACLLKEYQEVMNSKLGLDIEIATYRRLLEGEEHRLCEG
VGAVNVCVSSCRGGVVCGDLCASGAAPAVTTSVCSAPCSGNVVVGTADACGPCSGLGYSIVGSKRC

>Pc_K4
MLIRQQYARGGPRGFSCGSAIVGGGKKAAFSSISMSGGTGCYSSRGFSSRSLCNLGGNKSISVGTAGCRQ
GAGFGAAGGFGAGSFGLGFRSSFGGRGGAGFPVCPAGGIQEVTVNQSLLTPLHLEIDPEIQKIRTEEREQ
IKNLNDRFASFIDKVRFLEQQNKVLETKWNLLQQQTTTTSSKNFDPFFEAYLSALRKQVDTLTNNKGRLQ
CELKIMQDSVEDFKAKYEDEINKRTAAENDFVVLKKDVDATYMNKAELEAKAETLNDEINFLRVLYAAEL
SQMQTHVGDTSVVLTMDNNRNLDLDSIITEVRAQYEEIAQRSKAEAEALYQTKVQQLQASVEQHGDSLKN
TKNEISELNRMIQRLRAEIENVKKQCQTLQESVADAEQRGEVALKDAHSKRTELEVALQKAKEELARMLH
EYQELMSVKLALDIEIATYRKLLEGEECRMSGECQSAVSISVVGGAASAGGVGGGLGSCSGFGLGSGSRS
GFGFGGSVVVGGSSSSKIISTTTLAKRSHR

>Pc_K5
MSRHSSVSFRSRGGHSFSTASAITPSVSRTSFTSVSRSGGGGGGLGRVSLGGAGGVGGYGSRSLYNLGGS
KRISISSGGGGFRNRVCATGVGGSYGSGGGAGSGYVFGSGAGGFGLSGGAGFGGGYSGSCFPVCPPGGIQ
EVTINQSLLTPLNLQIDPTIQRVKTEEREQIKTLNNRFASFIDKVRFLEQQNKVLETKWALLQEQGTKIV
KQNLEPLFEQYTNNLRRQLDGILGERGRLDSELRNMQDLVEDFKNKYEDEINKRTTAENEFVMLKKDVDA
AYMNKVELEAKVDALMDEINFLKMFFEAELCQIQTHISDTSVVLSMDNNRCLDLDSIIAEVKAQYEEIAN
RSRTEAESWYQTKYEELQQTAGRHGDDLRNTKQEISEMNRMIQRMRSDIDNVKKQCTSLQSAIADAEQRG
ELALKDAKSKLAGLEEALQKAKQDMARLLKDYQELMNTKLSLDVEIATYRKLLEGEECRLSGEGVGPVNI
SYVTNTISSGYGSGSGLGGGLGVGMGSGFGGGSNFYSSRGGVIGSGSGLSMGGSSFSESSGRNLGFGSGG
GSGSSIKYVSTTSSSRKSFKS

>Pc_K6-l2-p
XXXXXXXXXXXXXXXXXXXXXXXXXXXXXXXXXXXXXXXXXXXXXXXXXXXXXXXXXXXXXXXXXXXXXX
XXXXXXXXXXXXXXXXXXXXXXXXXXXXXXXXXXXXXXXXXXXXXXXXXXXXXXXXXXXXXXXXXXXXXX
XXXXXXXXXXXXXXXXXXXXXXXXXXXXXXXXXXXXXXXXVRFLEQQNKVLETKWALLQEQCTKIVRHNL
EPLFEQYTNNLRRQLDCLLTERGCLDSEFRGMQEMVEDFKKKYEDEINKRTSAENEFVDLKKDVDAAYMN
KVELQAKADALMDEINFLRALYDLELTQMQSHISDTSVVLSMDNNRTLDLDSIIAEVKAQYEEIAHRSRE
EAESWYKCKYEELQVAACRHGDDLRDTKQEIAELNRMIQRMRSEIDHVRKQCTSLQSAVADAEQRGELAL

KDARSKLVELENALQKAKQDMAQLVKDYQELMNTKLSLDVEIATYRKLLEGEECRLIGEGVGQVNISVVQ
SSTCGGYGGAGGVSSGLGLGVCGGYSYGSGHGYGSGFSSSSGRATGGGLCSSGGGSSTVKYTTTSSSSKK
SYRN

>Pc_K7
MSIHFSSQVFSSRSSAFPGRGTQVRLNSVRPGGFGGSSSSLYGLGASRPRVAPRSSYGGPVGAGIHEVTI
SQSLLTPLQVNIDPSIQQVRQEEREQIKTLNNKFASFIDKVRFLEQQNKLLETKWALLQEQGSTKNSCLL
GIFEAQIAGLRKQLEALQLDGGRLEVELRNMQDVVEDFKNKYEDEINRRTAAENEFVVLKKDVDVAYTNK
VELEAKADTLNDEINFLRTLYEEELKELQSKVSNTSVVLSMDNNRSLDLDGIIAEVKAQYEEIANRSRAE
AEAWYQTKFETLQAQAGKHGDDLRNTRNEIAEMNRAVQRLQAEIDSNKNQSAKLEAAIAEAEERGELAVK
DARAKQEELEAALQRAKQDMARQLREYQELMSTKLALDIEIATYRKLLEGEESRMTGAGVGAVNISVVSS
TGGAGSRLTFGGSMGSNALRFSSGGGPGAPKA

>Pc_K8
MSIRVTQKSYKVSPSGPRSFSSRSYTSGPGARISSSAFSRVGSSSSFRGGLGSSMSLAAGYSGAPGLGGI
TAVTVNQSLLSPLKLEVDPDIQAVRTQEKEQIKTLNNKFASFIDKVRHLEQQNKILETKWNLLQQQKTAR
SNIDNMFESYINNLRRQLETLAQEKLKLEVELGNMQGLVEDFKTKYEEEIQKRTDMENEFVIIKKDVDEA
YMNKVELESRLEGLTDEINFYRQLYEEEIHEMQSQISDTSVVLSMDNSRSLDLDGIIAEVKAQYEEIANR
SRAEAETMYQIKYEELQTLAGKHGDDLRRTKTEISEMNRNINRLQAEIEGLKGQRASLEAAIADAEHRGE
LALKDAQAKLAELEAALRTAKQDMARQLREYQELMNVKLALDVEIATYRKLLEGEESRLESGMQNMSIHT
KTTSGYSGGLTSAYGTPGLSYGLSSYQSSLGSGGGSGSFSCSSSKAVVVKKIETRDGKLVSESSDILPK

>Pc_K78
MSLSPCRAQRGYSARSACSARSGGRGRVNFSSRSLSSFGGCRGGSRGRAWGSRGRLGVRYREGIGGPGLS
PCLPGGIQEVAINQNLLTPLKIEIDPQFQVVQTQETQLIRTLNNQFASFIDKVRFLEQQNKVLETKWDLL
QQQELSDSPQALQSFFEAYLVQLRKQLEQLQRERGSLEAELKSCQDQQEEYKAKYEREAHKRTTVEKDFV
VLKKDVDGVLSSKMELEGKMEALNEYICFLRHLYEEELGQLQTQASGMSVVLSMENNRRLDFRDLIAEVR
ARYEEIAGTSKAEAEMLYQTKYRELQVSAQLHGDQMKGTKVQITQLQQAIQKLQSQTENLKKQNANLQAA
ITHAEHRGELALKDAQTKLAELEAALRTAKQDMARLLREYQELMSSKLSLDVEIATYRRLLEGEESRTSG
ECASQVTISVGEGGTIVSGGADGGLVGTCGLGGGNGSFGSSCSSIVTGGDSNVILGSGQGPVLGSCSVSG
SGSSSTCHIILKKTVESSLKTSITY

>Pc_K80
MACRSCIVGSGNLNSCVVTQASSPWPGTSGWSNYRAPELGFSSSSLTGCLTASTIPKVTVNPSLLVPLDL
KVDPAIQQQKSQKKEEMKVLNDKFATLIGKVQALEQRNQLLETRWRFLQSRDSAAFDLGHLYEEYQGRLQ
EELRKVNQEGAQLEAKLLQQLEMVKEFQIRYEDEISKRTDMEFTSVQLKKDLESECLRRTELETKLKCLK
SFVVLMKSIYEQELKDLAAQVKDVSVTVSMDSRCHIDLSSIVEEVMAQYNAVMARSLEEAKAYSRSQLGE
RAACSAESGNSLQRSRGEIADLNVHIQKLRSQILSIKSHCLKLEENIKASENQGELAFQDAKAKLAQLED
ALQQAKKDMARQLRDYQELMNTKLALDIEIATYRKLVEGEESWMDLPSATVISSMQSRCRTAASKYGLSR
AASRKKKNREDLVIKITETSEEFLSQEPELSQ

>Pc_K85-c
MTCRSYRISPGCGVTRTFSSCSAVAPQTGSRCCISASPYRGVSCYRGLTGFGSRSVSALGSCGPRMAGGG
FRTGSRGRSFGYRSGGVCGPSPPCITTVSVNESLLAPLNPNAQCVKHEEKEQIKCLNSRFAAFIDKVRFL
EQQNKLLETKLQFYQNRQCCESNLEPLFQCYIQTLRQEAERVEADSGRLASELSHVQEVLEGYKEKYEEE
GALRATAENEFVVLKKDVDCASLRKSDLEANLGALVEESSFLKRLYDEELQVLHSHISDTSVFIKMDNSW
DLNMDCFVAEIKAQYDDVARRSRAEAESWYRSKCEEMKATVIRHRETLRCTKEEISELNRLIQRLTAEIE
NAKCQRAKLEAAVAKAEQQGEAALNDARCKLAGLEEALQKAKRDMTCLLKEYQEVMNSKLGLDIEIATYR
RLLEGEEHRLCEGVGSVNVCVSSSRGGVTWGASCVASPRAARLPLAPWPLGAASQ

>Pc_K86
MTCGSGFGGRAFSCVSACGPRPVRCCITAAPYRGISCYRGLTGGFGSRSLCGDFRAGSRGRSFGYRSGGV
CGPSPPCITTVSVNESLLAPLNLEIDPNAQCVKHEEKEQIKCLNDRFAAFIDKVRFLEQQNKLLETKLQF
YQNRQCCESNLEPLFQRYIQALRREAERVEADSGRLASELSHVQEVLEGYKEKYEAEVALRATAENEFVA
LKEDVDCAYLCKSDLEANSEALIQEIDFLRRLYEEEIRVLHSHILDTSVIVKMDNSRDLNMDSIVAEIKA
NYDDIVGRSRAEAESWYSSKCEEIKATVIWHGETLRRTKEEINELNRMIQRLTAEVENAKCQNSKLEAAV
TQAEQQGEAALNDARCKLAGLEEALQKAKRDMACLLKEYQEVMNSKLGLDIEIATYRRLLEGEEHRLCEG
VGAVNVCVSSCRGGVVCGDLCASGAAPAVTTSVCSAPCSGNLVVGTADVCSPCSGLGCSIVGSKTC

>Lv_K4
MLIRQQCVRGGPQGFSCGSAIVGGGKKAVFSSISMSGGTGCCSSGGFSSRSLCNLGGNKSIPFGTAGCRR
GAGFGAAGGLGAGCFGLGFGGSFGGQGGAGFAVCPAGGIQEVTINQSLLTPLHMEIDPEIQKVRTEEREQ
IKTLNDRFASFIDKVRFLEQQNKVLETKWNLLQQQATTTSSKNLDPFFEVYLSALRKQLDALTNNKGRLQ
YELKIMQDSVEDFKAKYEDEINKRTAAENDFVVLKKDVDATYMNKAELEAKVEARNDEVNFLRVLYAAEL
SQMQTHVSDTSVVLSMDNNRNLDLDGIIAEVRAQYEDIAQRSKAEAEALYQTKVQQLQASVEQHGDSLKS
TKNEISELNRMIHRLQAEIENVKKQCQTLQASVADAEQRGEVALKDAYSKRTELEVALQKAKEELARMLQ
EYQELMSVKLALDIEIATYRKLLEGEECRMSGECQSAVSISVVGGAAGAGGIGGGLGSCSGFGLGSGSGS
GFGFGGSVGVGGSSSSKIISTTTLAKRSHR

>Lv_K5
MSRQSSVSFRSGGGRSFSTASAITPSVSRTSFTSVSRSGSGGGGGGFGRVSLGGAYGMGGYGSRSLYNLG
GSKRISISSSGGGFRNRGCATGAGGGYGFGGGAGSGFGFGGGAGGGFGLGGGAGFGGGYGGSGSLVCPPG
GIQEVTINQSLLTPLNLQIDPTIQRVRTEEREQIKTLNNRFASFIDKVRFLEQQNKVLETKWALLQEQST
KTVRQNLEPLFEQYTNNLRRQLDGILGERGRLDSELRNMQDLVEDFKNKYEDEINKRTTAENEFVMLKKD
VDAAYMNKVELEAKVDALMDEINFLKMFFEAELSQIQTHVSDTSVVLTMDNNRCLDLDSIIAEVKAQYEE
IANRSRTEAESWYQTKYEELQQTAGRHGDDLRNTKQEISEMNRMIQRMRSDIDNVKKQCANLQNAVADAE
QRGELALKDARSKLAGLEDALQKAKQDMARLLKDYQELMNTKLALDVEIATYRKLLEGEECRLSEGIGPV
NISFVTNTVSSAYGSGGSFGSGLGGGLGGGLGGGSSGFYSSSSGGVGLGGGLGGGGSSFSASSGRGLGFG
SGGGSSSNVKFVSTTSSSRKSFKS

>Lv_K6-l2
MTSKSTVKSQSSSRQIFSAGSARVPGVNRSGFSCMPVCRSRGSGGFPGVGGGVSFGSRSLYGVGGSKRIS
LGGGSCALGGGYGGRAGGGLGFGGGAGSGFGFGGGAGGGFGLGGGAGFGGGYGGSGSLVCPPGGIQEVTI
NQSLLTPLKLQIDPNIHQIKTEEREQIKTLNNRFASFIDKVRFLEQQNKVLETKWALLQEQGTKTVTGIL
DPLFEQYTNNLRRQLDSLVTERSRLDSELRGMQDMVEDFKKKYEDEINKRMSAENEFVNLKKDVDTAYMN
KVDLQAKADALMDEINFLRALYEAELAQVQTHVSDTSVVLSMDNNRTLDLDSIIAEVKAQYEEIAHRSRE
EAESWYKCKYEELQVSAFRYGDDLRNTKQEISEINRMIQRLRSEINHVKKQCTNLQSAIADAEQRGDMAL
RDAKNKLIELENALQKAKQDMAQLLRDYQELMNVKLSLDVEIATYRKLLEGEECRLYGEGAGQVNISVVQ
STTSSGYGGAGGVSSGLGMGGGSGYSCGSGYSVGGGFSSGSGRAICGGLSSSGGSSSTVKFTTTSSSGRK
GYKP

>Lv_K6-l3
MISKSTVKSQSSSRQIFSAGSARVPGVNRSGFSCMPVRRSRGSGGLTGVGAGASFGSCSLYGVGGSKRIS
LGGGSCALGGGYGGRAGGGLGFGGGAGSGFGFGGGAGGGFGLGGGAGFGGGYGGSGSLVCPPGGIQEVTI
NQSLLTPLKLQIDPNIHQIKTEEREQIKTLNNRFASFIDKVRFLEQQNKVLETKWALLQEQGTKTVTGIL
DPLFEQYTNNLRRQLDSLVMERGRLDSELRGMQDTVEDFKKKYEDEINKRMSAENEFVNLKKDVDTAYMN
KVDLQAKADALMDEINFLRALYEAELAQVQTHVSDTSVVLSMDNNRTLDLDSIIAEVKAQYEEIAHRSRE
EAESWYKCKYEELQVSAFRYGDDLRNTKQEISEINRMIQRLRSEINHVKKQCTNLQSAIADAEQRGDMAL
RDAKNKLIELENALQKAKQDMAQLLRDYQELMNVKLSLDVEIATYRKLLEGEECRLYGEGAGQVNISVVQ
STTSSGYGGAGGVSSGLGMGGGSGYSCGSGYSVGGGFSSGSGRAICGGLSSSGGSSSTVKFTTTSSSGRK
GYKP

>Lv_K7
MSIHFSSQVFSSRSSAFPGRGTQVRLNSVRPGGFGGSSSSLYGLGASRPRVAARSSSGGPVGAGIREVTI
NQSLLTPLQVNIDPSIQQVRQEEREQIKTLNNKFASFIDKVQFLEQQNKLLETKWALLQEQKSTKSSRLL
GIFEAQTAGLRKQLEALQLDGGRLEVELRNMQDVVEDFKNKYKDEIDRRTAAENEFVVLKTDVDVAYMNK
VELEAKVDTLNDEINFLRTLYEEELEELQSKVSDTSVVLSMDNSRSLDLDGIIAEVKAQYEEIANRSRAE
AEAWYQTKFETLQAQAGKHGDDLRNTRNEIAELNRAVQRLQAEIDSNKNQRAKLEAAIAEAEERGELAVK
DARAKLVDLEEALQKAKQDMARQLREYQELMSTKLALDIEIATYRKLLEGEESRMTGAGVGAVNISVVGS
TGGAGSRLTFGGTMGSDALRFSSGGGPGAPKAYSIRTTSAPGRCTRN

>Lv_K8
MSIRVTQKSYKVSTSGRQSFSSRSYTSGPGARISSSAFSRVGSSSSSFRGGLGSSMSLASGYSGAPGLGG
ITAVTVNQSLLSPLKLEVDPDIQAVRTQEKEQIKTLNNKFASFIDKVRHLEQQNKILETKWNLLQQQKTA
RSNIDNMFESYINNLRRQLETLAQEKLKLEVELGNMQGLVEDFKTKYEEEIQKRTDMENEFVIIKKDVDE
AYMNKVELESRLEGLTDEINFYRQLYEEEIREMQSQISDTSVVLSMDNSRSLDLDGIIAEVKAQYEEIAN
RSRAEAETMYQIKYEELQTLAGKHGDDLRRTKTEISEMNRNISRLQAEIEGLKGQRASLEAAIADAEQRG
ELAVKDAQAKLAELEAALRTAKQDMARQLREYQELMNVKLALDVEIATYRKLLEGEESRLESGMQNMSIH
TKTTSGYSGGLTSAYGNPGFNYGLSSYQSSLGSGGGSGSFSRTSSKAVVVKKIETRDGKLVSESSDVLTK

>Lv_K78
MSLSPCWAQRGFSARSACSARSGVRGKVNFSSRSLSSFGGCQGSSRGRAWGSRGRLGVRYGEGISGPGLS
PCPPGGIQEVVINQNLLTPLKIEIDPQFQVVQTQQTQQIRTLNNQFASFIDKVRFLEQQNKVLETKWDLL
QQQELSDSPQALQSFFEAYLVQLRKQLEQLQRERGSLDAELKSCQYQQEEYKAKYEREAHRCATLEKDFV
VLKKDVDGVLSSKMELEGKVEALKEYICFSRRLYKEELGQLQTQASDMSVVLSMENNRRLDFRDLIAEVR
ARYQEIARTSKAEAEMLYQTKYRELQVSAQLHGDQMKGTKVQITELQQAIQKLQSQTENLKKQNAKLQAA
IAHAEQRGELAHKDAQTKLAELEATLRTAKQDMARLLREYQELMSSKLSLDVEIATYCRLLEGEESRTSG
ECASQVTISVGEGSTIVSGEADGGLVGTCGLGGGDGSFGSSCSSIVTGDSNVILGSGQGPVLGSCSVSGS
GSRSTCHTILRKTVESSLKTSITY

>Lv_K80
MACRSCIVGFGNLNSCEVTQASGPWPGTSGWSNCRAPEPGFSSSSLTGCLTASAIPKVTVNPSLLIPLDL
KVDPAIQQQKSQKKEEMKVLNDRFASLIGKVRALEHRNQLLETRWRFLQSRDLAAFDLGHLYEEYQGRLR
EKLHKVNQERGQLETKLLQELETVKAFQIRYEDEISKRTDMEFTFAQLKKDLDAECLRRTGLETKLKGLK
NFVVLMKSIYEQELKDLAAQVKDVSVTVGMDSRCHIDLSGIVEEVMAQYDPVMARSLEEAKAYSQSQVRK
RAACSAESGNSLQRRRGDIADLKVHIQKLRSQILSIKSHCLKLEENIKAAEKQGELAFQGAKAKLAQPEA
ALQQAKQDMARQLRDCQELMSTKLALDVEIATYRKLVEGEESRMDLPSATVISSMQSRSRNAASKYGLSR
APSRKKKTREDLVIKITETSEEFLSQESELSQ

>Lv_K85-c
MTCRSYRISPGCGVTRTFSSCSAVAPKTGSHCCISAAPYRGVSCYRGLTGFGSLSVSALGSCGPRIAGGG
FGAGSCGRSFGYRFGGVCGPSPPCITTVSVNERLLTPLNLEIEPNAQCVKHEEKEQIKCLNSRFAAFIDK
VRFLEHQNKLLETKWQFHQNRQRCDSNLEPLFQGYVQTLRREAERVEADSGRLASELSHVQEVLEGYREK
YEEEGALRATAENEFMVLKKDVDCAYLRKSDLEANVEALVEESSFLKRLYDEELQVLHAHISDTSVFIKM
DNSRDLNMDCFVAEIKAQYDDVASRSRAEAESWYRSKCEEMKATVIQHREALRRTKEEISELSRTSRRLT
AEVENAKCQRAKLEAAVPKAEQQGEAALNDARCKLAGLEEALQKAKRDMACLLKEYQEVMSSKLGLDIEI
ATYRRLLEGEEHRLCEGVGSVNVCVSSSRGGVTWGASCVAPPRDAGLPLAPWPLGAASQ

>Oo_K4-c
MLIRQQCVRGGPQGFSCGSAIVGGGKKPAFSSISMSGGTGSCSGGFSSRSLCNLGGNKSISIGTAGCWRG
AGFGAAGGLGAGCFGLGFGGSFGGRGGAGFPVCPAGGIQEVTINQSLLTPLHMEIDPEIQKVRTEEREQI
KTLNDRFASFIDKVRFLEQQNKVLETKWNLLQQQATTTSSKNLDPLFEAYLSALRKQLDTLTNNKGGLQY
ELKIMQDSVEDFKAKYEDEINKRTAAENDFVVLKKDVDATYMNKAELEAKVEARNDEINFLRVLYAAELS
QMQTHVSDMSVVLSMDNNRNLDLDGIIAEVKAQYEDIAQRSKAEAEALYQTKVQQLQASVEQHGDSLKST
KNEISELNRMIQRLQAEIKNVKKQCQTLQASMAGAEQRGEAALKDAHGKRTEMEGALQKAKEELVRMLQE
YQELMSVKLALDIEIATYRKLLEGEECRMSGECQSAVSISVAGGAASAGGVGGGLGSCSGFGLGSGSGSG
FGFGGSVGVGGSSSSKIISTTTLAKRSHR

>Oo_K5
MSRQSSVSFRSGGNRSFSTASAITPSVSRTSFTSVSRSGGGGGGGGFGRVSLGGAYGTGGYGSRSLYNLG

GSKRISISSSGGGFRNRGCATGAGGGYGFGGGAGGGFGFGGGAGGGFGLSGGAGFGGGYGGSGFSVCPPG
GIQEVTINQSLLTPLNLQIDPNIQRVRTEEREQIKTLNNRFASFIDKVRFLEQQNKVLETKWALLQEQST
KTVRQNLEPLFEQYTNNLRRQLDGILGERGRLDSELRNMQDLVEDFKNKYEDEINKRTTAENEFVMLKKD
VDAAYMNKVELEAKVEALMDEINFLKMFFEAELSQIQTHISDTSVVLSMDNNRCLDLDSIIAEVKAQYEE
IANRSRTEAESWYQTKYEELQLTAGRHGDDLRNTKQEISEMNRMIQRMRSDIDNVKKQCSNLQNAVADAE
QRGELALKDARNKLSGLEDALQKAKQDMARLLKDYQELMNTKLALDVEIATYRKLLEGEECRLSEGIGPV
NISFVTNTVSSAYGGGGSFGSSLGGGLGGGLGGGSSGFYSSSSGGTGLGSGLGVGGSSFSAGSGRSLGFG
SGGGSSSNVKFVSTTSSSRKSFKS

>Oo_K6-p
MTSSSTVRSQSGSRRVFSAGSARVPGVNRSGFSSMSVCRSRGSGGFTGVGAGASFGSRSLYGIGGSKRIS
LGGGSCAFGGGYGGRAGGGLGFGGGAGSGFGFGSGADGGFGLGGGAGFGGGYGGSGFSICPPGGIQEVTI
NQSLLTPLNLQIDPTIQRIKTEEREQIKTLNNRFASFIDKVRFLEQQNKVLETKWALLQEQGTKTVRQSL
EPFFEQYINNLRGQLDCLVSERSRLDSELRGMQDTVEDFKKKYEDEINKRMSAENEFVNLKKDVDVAYMS
KVDLQAKADALIDEINFLRALYEAELAQMQTHVSDTSVVLSMDNNRTLDLDSIIAEVKAQYEEIARRSRE
EAESWYKCKXXXXXXXXXXXXXXXXXXXXXXXXXXXXXXXXXXXXXXXXXXXXXXXXCTSLQSAIADAEQRGELAL
RDAKNKLAELENALQKAKQDMAQLLKDYQELMNVKLALDVEIATYRKLLEGEECRLYGEGAGQVNIAVVQ
STTSSGYGGAGGAGGVSSGYGMSGGGVSSGYGVGGGSGYSYGSGLSVGGGFSSSSGRAIGGGLSSSGGSS
STVKFSTTTSSSGRKSYRP

>Oo_K7
MSIHFSSQVFSSRSSAFPGRGTQVRLNSVRPGGFGGSSSSLYGLGASRPRVAARSSYGGPVGAGIREVTI
NQSLLTPLQVNIDPSIQQVRQEEREQIKTLNNKFASFIDKVRFLEQQNKLLETKWALLQEQKSTKRSCLP
GIFEAQTAGLRKQLEALQLDGGRLEVELRNMQDVVEDFKNKYEDEINRHTAAENEFVVLKKDVDVAYMNK
VELEAKVDTLKDEINFLRTLYEEELKELQSKVSDTSVVLSMDNSRSLDLDGIIAEVKAQYEEIANRSRAE
AEAWYQTKFETLQAQAGKHGDDLRRSRNEIAELNRAVQRLQAEIDNNKNQRAKLEAAISEAEERGELAVK
DARAKLVDLEEALQKAKQDMARQLREYQELMSTKLALDVEIATYRKLLEGEESRMTGAGVGAVNISVVGS
TGGAGSRLTFRGTMGSNALRFSSGGGPGAPKAYSISTTSAPGRRTHN

>Oo_K8
MSIRVTQKSYKVSTSGPRSFSSRSYTSGPGTRISSSAFSRVGSSGSFRGGLGSSMSLAAGYSGAPGLGGI
TAVTVNQSLLSPLKLEVDPDIQAVRTQEKEQIKTLNNKFASFIDKVRHLEQQNKILETKWNLLQQQKTAR
SNIDNMFESYINNLRRQLETLAQEKLKLEVELGNMQGLVEDFKTKYEEEIQKRTDMENEFVIIKKDVDEA
YMNKVELESRLEGLTDEINFYRQLYEEEIREMQSQISDTSVVLSMDNSRSLDLDGIIAEVKAQYEEIANR
SRAEAETMYQMKYKELQTLAGKHGDDLRRTKTEISEMNRNINQLQAEIEGLKGQRASLEAAIADAEQRGE
LAVKDAQAKLAELEAALRTAKQDMARQLREYQELMNVKLALDVEIATYRKLLEGEESRLESGMQNMSIHT
KTTSGYSGGLTSPYGNPGFNYGLSSYQSSLGSGGGSGSFSRTSSKAVVVKKIETRDGKLVSESSDILPK

>Oo_K78
MSLSPCRAQRGFSSCSACSARSGGRGRVNFSSRSLSSFGGCQGGSRGRAWGSRGRLGVRYGEGIGGPGLS
PCPPGGIQEVAINQNLLTPLKIEIDPQFQVVQTQQTQQIRTLNNQFASFIDKVRFLEQQNKVLETKWDLL
QQQELSDSPQALQSFFEACLFQLRKQLEQLQGERVSLDAELKSCQYQQEEYKAKYEREAHRCATLEKDFV
VLKKDVDGVLSSKMELEGNVEALKEYICFLRRLYEEELGQLQTQASDMSVVLSMENNRHLDFRDLITEVC
ARYEEIAGTSKAEAEMLYQTKYRELQVSAQLHGDRMKGTKVQITQLQQAIQKLQSQTENLKKQNAKLQAA
IAHAEQRGELAVKDAQTKLAELEAALRTAKQDVARLLREYQELMSSKLSLDVEIATYRRLLEGEESRTSG
EYTGQVTISVGQGSTIVSGGADGGLVGTCGLGGGNGSFGSSRSSIVTGDSNVILGSGQGPVLGSCSVSGS
GSSSTCHTILKKTVESSLKTSITY

>Oo_K80
MACRSCVVGFGNLTSCEVTQASGAWPGTSGWSNYRAPEPGFSSSSLTGCLTASTIPRVTVNPSLLVPLDL
KVDPAMQQQESQKKEEMKVLNDKFASLIGKVRALEHRNQLLETRWRFLQSRDSAAFDLGHLYEEHQGRLR
EKLREVNQEGGQLETKLRQELETVKAFQIRYEDEISKRTDMEFTFAQLKKDLDAECLQRTGLENKLKGLK
SFVVLMKSIYEQELKDLAAQVKDVSVTVSMDSRCHVDLSGIMEEVMAQYDAVAARSLEEGKAYSQSQVEE
RAACSAEAGNSLQRSRSEIADLKVHIQKLRSQILSTKSHCMKLEENIKAAESQGELTLQDAKAKLAQLEA
ALQQAKQDMARQLRDYQELMSTKLALDVEIATYRKLVEGEESRMDLPSATMIRSMQSSSRTAASRHGLSR
APSWKKKTRGDLVIKITETSEEFLSQESELSQ

>Oo_K81
MTYGSGFGGRAFSCFSACGTRPSRCSITAAPYRGISCYRGLTGGFGSRSLCEGFRTGSCGRSFGYRSGGV
CGPSPPCITTVSVNERLLAPLNLEIDPNVQCVKHEEKEQIKCLNNRFAAFIDKVRFLEQQNKLLETKWQF
YQNRQCCESNLEPLFQGYIQTLRREAECVEADSGRLASELSHMQEVLEGYKQKYEAEVALRVTAENEFVA
LKKDVDCAYLCKSDLEANSEALIQEIDFLRRLYEEEIRVLHTHISDASVIIKMDNSRDLNMDSIVAEIKA
NYDDIAGRSRAEAESWYRSKCEEMKATVLRHGETLRRTKEEINELNRVIQRLTAEVENAKCQNSKLEATV
TQAEQQGEAALNDARCKLAGLEEALQKAKRDMACLLKEYQEVMNSKLGLDIEIATYRRLLEGEEHRLCEG
VGAVNVCVSSSRGGVVCGDLCVSGSRPVTGSVCSGPCSGNLAVSTGLCGPCGPCSSITSCGVGSRASSCR
KC

>Oo_K85-c
MTCRSYRISPGCGVTRTFSSCSAVAPQTGSRCCISAAPYRGVSCYRGVTGFGSRSVSVLGSCGPRIAWGG
FRAGSCGRSFGYRFGGVCGPSPPCITTVSVNERLLAPLNLEIDPNAQCVKHEEKEQIKCLNSRFAAFIDK
VRFLEQQNKLLETKWQFHQNLQCCESNLEPLFQGYIQTLRREAERVEAESGRLASELSHVQEVLEGYKEK
YEEEGALRATAENEFMVLKKDVDCAYLRKSDLEANVEALVEESSFLKRLYDEELQVLHAHISDTSVFVKM
DNSRDLNMDCFVAEIKAQYDDVASHSRAEAESWYRSKCEEMKATVLRHRETLRRTKEEIGELNRVIQRLT
AEVENAKCQRAKLEAAVPKAEQQGEAALNDARCKLAGLEEALQKAKRDMACLLKEYQEVMNSKLGLDIEI
ATYRRLLEGEEHRLCEGVGSVNVCVSSSRGGVMYSTTPGRQIASGPVATGGSITVMAPESCAPVSPASPA
SAAGAGPSAFPRVRGPACVPRARNGV

>Tt_K4-h-p
MLIRQQCVRGGPQGFSCGSAIVGGGKKPAFSSISMSGGTGSCSGGFSSRSLCNLGGNKSISIGTAGCWRG
AGFGAAGGLGAGCFGLGFGGSFGGRGGAGFPVCPAGGIQEVTINQSLLTPLHMEIDPEIQKVRTEEREQI

KTLNDKFASFIDKVRFLEQQNKVLETKWNLLQQQATTTSSKNLDPLFEAYLSALRKQLDTLTNNKGGLQY
ELKIMQDSVEDFKAKYEDEINKRTAAENDFVVLKKDVDATYMNKAELEAKVEARNDEINFLRVLYAAELS
QMQTHVSDMSVVLSMDNNRNLDLDGIIAEVKAQYEDIAQRSKAEAEALYQTKVQQLQASVEQHGDSLKST
KIEISELNRMIQRLQAEIENVKKQCQTLQASVAGAEQRGEAALKDAHGKRTEMEGALQKAKEELVRMLQE
YQELMSVKLALDIEIATYRKLLEGEECRXXXXXXXXXXXXXXXXXXXXXXXXXXXXXXXXXXXXXXXXXX
XXXXXXXXXXXXXXXXXXXXXXXXXXXXX

>Tt_K5-c
MSRQSSVSFRSGGNRSFSTASAITPSVSRTSFTSVSRSGGGGGGGGGFGRVSLGGAYGTGGYGSRSLYNLG
GSKRISISSSGGGFRNRGCATGAGGGYGFGGGAGGGFGFGGGAGGGFGLSGGAGFGGGYGGSGFSVCPPG
GIQEVTINQSLLTPLNLQIDPNIQRVRTEEREQIKTLNNRFASFIDKVRFLEQQNKVLETKWALLQEQGT
KTVRQNLEPLFEQYTNNLRRQLDGILGERGRLDSELRNMQDLVEDFKNKYEDEINKRTTAENEFVMLKKD
VDAAYMNKVELEAKVEALMDEINFLKMFFEAELSQIQTHISDTSVVLSMDNNRCLDLDSIIAEVKAQYEE
IANRSRTEAESWYQTKYEELQLTAGRHGDDLRNTKQEISEMNRMIQRMRSDIDNVKKQCSNLQNAVADAE
QRGELALKDARNKLSGLEDALQKAKQDMARLLKDYQELMNTKLALDVEIATYRKLLEGEECRLSEGIGPV
NISFVTNTVSSAYGGGGSFGSGLGGGLGGGLGGGSSGFYSSSSGGAGLGSGLGAGGSSFSAGSGRSLGFG
SGGGSSSNVKFVSTTSSSRKSFKS

>Tt_K6-l2-h-c
MTSRSTVRSQSGSRRVFSAGSARVPGVNRSGFSSMSVCRSRGSGGFTGVGGGASFGSRSLYGIGGTKRIS
LGGGSCALGGGYGGRAGGGLGFGVGAGSGFGFGSGAVGGIGLGGGAGGGFGLGGGAGFSGGYGGSGFSIC
PPGGIQEVTINQSLLTPLNLQIDPTIQRIKTEEREQIKTLNNRFASFIDKVRFLEQQNKVLETKWALLQE
QGTKTVRQSLEPYFEQYINNLRRQLDCLVSERSRLDSELRGMQDTVEDFKKKYEDEINKRTSAENEFVNL
KKDVDVAYMTKVDLQANADALIDEINFLRALYEAELAQMQTHVSDTSVVLSMDNNRTLDLDSIIAEVKAQ
YEEIARRSREEAESWYKCKYEELQSSACRYGDDLCNTKQEIAEINRMIQRLRSEIEHVKKQCTSLQSAIA
DAEQRGELALRDAKNKLAELENALQKAKQDMAQLLKDYQELMNVKLALDVEIATYRKLLEGEECRLYGEG
AGQVNISVVQSTTSSGYGSAGGVSSGYGMGGGGVSSGYGMGGGSGYSYSSGLSVGGGFSSSSGRAIGGGL
SSSGGSSSTVKFSTTSSSGRKSYRP

>Tt_K7-h-p
MSIHLSSQVFSSRSSAFPGRGTQVRLNSVRPGGFGGSSSSLYGLGASRPRVAARSSYGGPVGAGIREVTI
NQSLLTPLQVNIDPSIQQVRQEEREQIKTLNNKFASFIDKVRFLEQQNKLLETKWALLQEQKSTKRSCLP
GIFEAQTAGLRKQLEALQLDGGRLEVELRNMQDVVEDFKNKYEDEINRHTAAENEFVVLKKDVDCAYLCK
SDLEANSEALIQEIDFLRRLYEEELKELQSKVSDTSVVLSMDNSRSLDLEGIIAEVKAQYEEIANRSRAE
AEAWYQTKFETLQAQAGKHGDDLRRSRNEIAELNRAVQRLQAEIDSNKNQXXXXXXXXXXXXXXXXXXXX
XXXXXLVDLEEALQKAKQDMARQLREYQELMSTKLALDIEIATYRKLLEGEESRMTGAGVGAVNISVVGS
TGGAGSWLTFGGTMGSDALRFSSGGGPGAPKAYSIRTTSAPGRCTRN

>Tt_K8-h
MSIRVTQKSYKVSTSGPRSFSSRSYTSGPGTRISSSAFSRVGSSGSFRGGLGSSMSLAAGYSGAPGLGGI
TAVTVNQSLLSPLKLEVDPDIQAVRTQEKEQIKTLNNKFASFIDKVRHLEQQNKILETKWNLLQQQKTAR
SNIDNMFESYINNLRRQLETLAQEKLKLEVELGNMQGLVEDFKTKYEEEIQKHTDMENEFVIIKKDVDEA
YMNKVELESRLEGLTDEINFYRQLYEEEIREMQSQISDTSVVLSMDNSRSLDLDGIIAEVKAQYEEIANR
SRAEAETMYQMKYKELQTLAGKHGDDLRRTKTEISEMNRNINQLQAEIEGLKGQRASLEAAIADAEQRGE
LAVKDAQAKLAELEAALRTAKQDMARQLREYQELMNVKLALDVEIATYRKLLEGEESRLESGMQNMSIHT
KTTSGYSGGLTSPYGNPGFNYGLSSYQSSLGSGGGSGSFSRTSSKAVVVKKIETRDGKLVSESSDILPK

>Tt_K78-h
MSLSPCRAQRGFSACSACSARSGGRGRVNFSSRSLSSFGGCQGGSRGRAWGSRGRLGVRYGEGIGGPGLS
PCPPGGIQEVAINQNLLTPLKIEIDPQFQVVQTQQTQQIRTLNNQFASFIDKVRFLEQQNKVLETKWDLL
QQQELSDSPQALRSFFEACLFQLRKQLEQLQGERVSLDAELKSCQYQQEEYKAKYEREAHRCATLEKDFV
VLKKDVDGVLSSKMELEGKVEALKEYICFLRRLYEEELGQLQTQASDMSVVLSMENNRHLDFRDLITEVC
ARYEEIAGTSKAEAEMLYQTKYRELQVSAQLHGDRMKGTKVQITQLQQAIQKLQSQTENLKKQNAKLQAA
IAHAEQRGELALKDAQTKLAELEAALRTAKQDVARLLSEYQELMSSKLSLDVEIATYRRLLEGEESRTSG
EYTGQVTISVGQGSTIVSGGADGGLVGTCGLGGGNGSFGSSRSSIVTGDSNVILGSGQGPVLGSCSVSGS
GSSSTCHTILKKTVESSLKTSITY

>Tt_K80-c
MACRSCVVGFGNLNSCEVTQASEPWPGTSGWNNYRAPGPGFSSSSLTGCLTASTIPRVTVNPSLLVPLDL
KVDPATQQQESQKKEEMKVLNDKFASLIGKVRALEHRNQLLETRWRFLQSRDSAAFDLGHLYEEHQGRLR
EKLREVNQEGGQLETKLRQELETVKAFQIRYEDEISKRTDMEFTFAQLKKDVDAECLQRTGLENKLKGLK
SFVVLMKSIYEQELKDLAAQVKDVSVTVSMDSRCHVDLSGIMEEVMAQYDAVAARSLEEGKAYSQSQVEE
RAACSAEAGNSLQRSRGEIADLKVHIQKLRSQILSTKSHCMKLEENIKAAESHGELTLQDAKAKLAQLEA
ALQQAKQDMARQLRDYQELMSTKLALDVEIATYHKLVEGEESRMDLPSTTMIRSMQSSSRTAASKHGLSR
APSWKKKTRGDLVIKITETSEEFLSQESELSQ

>Tt_K81-h
MTYGSGFGGRAFSCFSACGTRPSRCSITAAPYRGISCYRGLTGGFGSRSLCEGFRTGSCGRSFGYRSGGV
CGPSPPCITTVSVNESLLAPLNLEIDPNVQCVKHEEKEQIKCLNNRFAAFIDKVRFLEQQNKLLETKWQF
YQNRQCCESNLEPLFQAYIQTLRREAECVEADSGRLASELSHMQEVLEGYKQKYEAEVALRVTAENEFVA
LKKDVDCAYLCKSDLEANSEALIQEIDFLRRLYEEEIRVLHTHISDASVIIKMDNSRDLNMDSIVAEIKA
NYDDIAGRSRAEAESWYRSNCEEMKATVLRHGETLRRAKEEINELNRVIQRLTAEVENAKCQNSKLEATV
TQAEQQGEAALNDARCKLAGLEEALQKAKRDMACLLKEYQEVMNSKLGLDIEIATYRRLLEGEEHRLCEG
VGAVNVCVSSSRGGVVCGDLCVSGSRPVTGSVCSVPCSGNLAVSTGLCGPYGPCSSVTSCGVGSRASSCR
KC

>Hs_K1
MSRQFSSRSGYRSGGGFSSGSAGIINYQRRTTSSSTRRSGGGGGRFSSCGGGGGSFGAGGGFGSRSLVNL
GGSKSISISVARGGGRGSGFGGGYGGGGFGGGGFGGGGFGGGGIGGGGFGGFGSGGGGFGGGGFGGGGYG

```
GGYGPVCPPGGIQEVTINQSLLQPLNVEIDPEIQKVKSREREQIKSLNNQFASFIDKVRFLEQQNQVLQT
KWELLQQVDTSTRTHNLEPYFESFINNLRRRVDQLKSDQSRLDSELKNMQDMVEDYRNKYEDEINKRTNA
ENEFVTIKKDVDGAYMTKVDLQAKLDNLQQEIDFLTALYQAELSQMQTQISETNVILSMDNNRSLDLDSI
IAEVKAQYEDIAQKSKAEAESLYQSKYEELQITAGRHGDSVRNSKIEISELNRVIQRLRSEIDNVKKQIS
NLQQSISDAEQRGENALKDAKNKLNDLEDALQQAKEDLARLLRDYQELMNTKLALDLEIATYRTLLEGEE
SRMSGECAPNVSVSVSTSHTTISGGGSRGGGGGGYGSGGSSYGSGGGSYGSGGGGGGGRGSYGSGGSSYG
SGGGSYGSGGGGGGHGSYGSGSSSGGYRGGSGGGGGGSSGGRGSGGGSSGGSIGGRGSSSGGVKSSGGSS
SVKFVSTTYSGVTR

>Hs_K2
MSCQISCKSRGRGGGGGGFRGFSSGSAVVSGGSRRSTSSFSCLSRHGGGGGGFGGGGFGSRSLVGLGGTK
SISISVAGGGGGFGAAGGFGGRGGGFGGGSSFGGGSGFSGGGFGGGGFGGGRFGGFGGPGGVGGLGGPGG
FGPGGYPGGIHEVSVNQSLLQPLNVKVDPEIQNVKAQEREQIKTLNNKFASFIDKVRFLEQQNQVLQTKW
ELLQQMNVGTRPINLEPIFQGYIDSLKRYLDGLTAERTSQNSELNNMQDLVEDYKKKYEDEINKRTAAEN
DFVTLKKDVDNAYMIKVELQSKVDLLNQEIEFLKVLYDAEISQIHQSVTDTNVILSMDNSRNLDLDSIIA
EVKAQYEEIAQRSKEEAEALYHSKYEELQVTVGRHGDSLKEIKIEISELNRVIQRLQGEIAHVKKQCKNV
QDAIADAEQRGEHALKDARNKLNDLEEALQQAKEDLARLLRDYQELMNVKLALDVEIATYRKLLEGEECR
MSGDLSSNVTVSVTSSTISSNVASKAAFGGSGGRGSSSGGGYSSGSSSYGSGGRQSGSRGGSGGGGSISG
GGYGSGGGSGGRYGSGGGSKGGSISGGGYGSGGGKHSSGGGSRGGSSSGGGYGSGGGGSSSVKGSSGEAF
GSSVTFSFR

>Hs_K3
MSRQASKTSGGGSQGFSGRSAVVSGSSRMSCVAHSGGAGGGAYGFRSGAGGFGSRSLYNLGGNKSISISV
AAGGSRAGGFGGGRSSCAFAGGYGGGFGSGYGGGFGGGFGGGRGMGGGFGGAGGFGGAGGFGGAGGFGGP
GGFGGSGGFGGPGSLGSPGGFGPGGFPGGIQEVTINQSLLQPLNVEIDPQIGQVKAQEREQIKTLNNKFA
SFIDKVRFLEQQNKVLETKWNLLQQQGTSSISGTNNLEPLFENHINYLRSYLDNILGERGRLDSELKNME
DLVEDFKKKYEDEINKRTAAENEFVTLKKDVDSAYMNKVELQAKVDALIDEIDFLRTLYDAELSQMQSHI
SDTSVVLSMDNNRSLDLDSIIAEVRAQYEDIAQRSKAEAEALYQTKLGELQTTAGRHGDDLRNTKSEIIE
LNRMIQRLRAEIEGVKKQNANLQTAIAEAEQHGEMALKDANAKLQELQAALQQAKDDLARLLRDYQELMN
VKLALDVEIATYRKLLEGEEYRMSGECPSAVSISVVSSSTTSASAGGYGGGYGGGMGGGLGGGFSAGGGS
GSGFGRGGGGGIGGGFGGGSSGFSGGSGFGSISGARYGVSGGGFSSASNRGGSIKFSQSSQSSQRYSR

>Hs_K4
MIARQQCVRGGPRGFSCGSAIVGGGKRGAFSSVSMSGGAGRCSSGGFGSRSLYNLRGNKSISMSVAGSRQ
GACFGGAGGFGTGGFGGGFGGSFSGKGGPGFPVCPAGGIQEVTINQSLLTPLHVEIDPEIQKVRTEEREQ
IKLLNNKFASFIDKVQFLEQQNKVLETKWNLLQQQTTTTSSKNLEPLFETYLSVLRKQLDTLGNDKGRLQ
SELKTMQDSVEDFKTKYEEEINKRTAAENDFVVLKKDVDAAYLNKVELEAKVDSLNDEINFLKVLYDAEL
SQMQTHVSDTSVVLSMDNNRNLDLDSIIAEVRAQYEEIAQRSKAEAEALYQTKVQQLQISVDQHGDNLKN
TKSEIAELNRMIQRLRAEIENIKKQCQTLQVSVADAEQRGENALKDAHSKRVELEAALQQAKEELARMLR
EYQELMSVKLALDIEIATYRKLLEGEEYRMSGECQSAVSISVVSGSTSTGGISGGLGSGSGFGLSSGFGS
GSGSGFGFGGSVSGSSSSKIISTTTLNKRR

>Hs_K5
MSRQSSVSFRSGGSRSFSTASAITPSVSRTSFTSVSRSGGGGGGGFGRVSLAGACGVGGYGSRSLYNLGG
SKRISISTSGGSFRNRFGAGAGGGYGFGGGAGSGFGFGGGAGGGFGLGGGAGFGGGFGGPGFPVCPPGGI
QEVTVNQSLLTPLNLQIDPSIQRVRTEEREQIKTLNNKFASFIDKVRFLEQQNKVLDTKWTLLQEQGTKT
VRQNLEPLFEQYINNLRRQLDSIVGERGRLDSELRNMQDLVEDFKNKYEDEINKRTTAENEFVMLKKDVD
AAYMNKVELEAKVDALMDEINFMKMFFDAELSQMQTHVSDTSVVLSMDNNRNLDLDSIIAEVKAQYEEIA
NRSRTEAESWYQTKYEELQQTAGRHGDDLRNTKHEISEMNRMIQRLRAEIDNVKKQCANLQNAIADAEQR
GELALKDARNKLAELEEALQKAKQDMARLLREYQELMNTKLALDVEIATYRKLLEGEECRLSGEGVGPVN
ISVVTSSVSSGYGSGSGYGGGLGGGLGGGLGGGLAGGSSGSYYSSSSGGVGLGGGLSVGGSGFSASSGRG
LGVGFGSGGGSSSSVKFVSTTSSSRKSFKS

>Hs_K6B
MASTSTTIRSHSSSRRGFSANSARLPGVSRSGFSSISVSRSRGSGGLGGACGGAGFGSRSLYGLGGSKRI
SIGGGSCAISGGYGSRAGGSYGFGGAGSGFGFGGGAGIGFGLGGGAGLAGGFGGPGFPVCPPGGIQEVTV
NQSLLTPLNLQIDPAIQRVRAEEREQIKTLNNKFASFIDKVRFLEQQNKVLDTKWTLLQEQGTKTVRQNL
EPLFEQYINNLRRQLDNIVGERGRLDSELRNMQDLVEDLKNKYEDEINKRTAAENEFVTLKKDVDAAYMN
KVELQAKADTLTDEINFLRALYDAELSQMQTHISDTSVVLSMDNNRNLDLDSIIAEVKAQYEEIAQRSRA
EAESWYQTKYEELQITAGRHGDDLRNTKQEIAEINRMIQRLRSEIDHVKKQCANLQAAIADAEQRGEMAL
KDAKNKLEGLEDALQKAKQDLARLLKEYQELMNVKLALDVEIATYRKLLEGEECRLNGEGVGQVNISVVQ
STVSSGYGGASGVGSGLGLGGGSSYSYGSGLGVGGGFSSSSGRATGGGLSSVGGGSSTIKYTTTSSSSRK
SYKH

>Hs_K6C
MASTSTTIRSHSSSRRGFSANSARLPGVSRSGFSSISVSRSRGSGGLGGACGGAGFGSRSLYGLGGSKRI
SIGGGSCAISGGYGSRAGGSYGFGGAGSGFGFGGGAGIGFGLGGGAGLAGGFGGPGFPVCPPGGIQEVTV
NQSLLTPLNLQIDPAIQRVRAEEREQIKTLNNKFASFIDKVRFLEQQNKVLDTKWTLLQEQGTKTVRQNL
EPLFEQYINNLRRQLDSIVGERGRLDSELRNMQDLVEDLKNKYEDEINKRTAAENEFVTLKKDVDAAYMN
KVELQAKADTLTDEINFLRALYDAELSQMQTHISDTSVVLSMDNNRNLDLDSIIAEVKAQYEEIAQRSRA
EAESWYQTKYEELQVTAGRHGDDLRNTKQEIAEINRMIQRLRSEIDHVKKQCASLQAAIADAEQRGEMAL
KDAKNKLEGLEDALQKAKQDLARLLKEYQELMNVKLALDVEIATYRKLLEGEECRLNGEGVGQVNVSVVQ
STISSGYGGASGVGSGLGLGGGSSYSYGSGLGIGGGFSSSSGRAIGGGLSSVGGGSSTIKYTTTSSSSRK
SYKH

>Hs_K6A
MASTSTTIRSHSSSRRGFSANSARLPGVSRSGFSSVSVSRSRGSGGLGGACGGAGFGSRSLYGLGGSKRI
SIGGGSCAISGGYGSRAGGSYGFGGAGSGFGFGGGAGIGFGLGGGAGLAGGFGGPGFPVCPPGGIQEVTV
NQSLLTPLNLQIDPTIQRVRAEEREQIKTLNNKFASFIDKVRFLEQQNKVLETKWTLLQEQGTKTVRQNL
EPLFEQYINNLRRQLDSIVGERGRLDSELRGMQDLVEDFKNKYEDEINKRTAAENEFVTLKKDVDAAYMN
```

KVELQAKADTLTDEINFLRALYDAELSQMQTHISDTSVVLSMDNNRNLDLDSIIAEVKAQYEEIAQRSRA
EAESWYQTKYEELQVTAGRHGDDLRNTKQEIAEINRMIQRLRSEIDHVKKQCANLQAAIADAEQRGEMAL
KDAKNKLEGLEDALQKAKQDLARLLKEYQELMNVKLALDVEIATYRKLLEGEECRLNGEGVGQVNISVVQ
STVSSGYGGASGVGSGLGLGGGSSYSYGSGLGVGGGFSSSSGRAIGGGLSSVGGGSSTIKYTTTSSSSRK
SYKH

>Hs_K7
MSIHFSSPVFTSRSAAFSGRGAQVRLSSARPGGLGSSSLYGLGASRPRVAVRSAYGGPVGAGIREVTINQ
SLLAPLRLDADPSLQRVRQEESEQIKTLNNKFASFIDKVRFLEQQNKLLETKWTLLQEQKSAKSSRLPDI
FEAQIAGLRGQLEALQVDGGRLEAELRSMQDVVEDFKNKYEDEINHRTAAENEFVVLKKDVDAAYMSKVE
LEAKVDALNDEINFLRTLNETELTELQSQISDTSVVLSMDNSRSLDLDGIIAEVKAQYEEMAKCSRAEAE
AWYQTKFETLQAQAGKHGDDLRNTRNEISEMNRAIQRLQAEIDNIKNQRAKLEAAIAEAEERGELALKDA
RAKQEELEAALQRGKQDMARQLREYQELMSVKLALDIEIATYRKLLEGEESRLAGDGVGAVNISVMNSTG
GSSSGGGIGLTLGGTMGSNALSFSSSAGPGLLKAYSIRTASASRRSARD

>Hs_K8
MSIRVTQKSYKVSTSGPRAFSSRSYTSGPGSRISSSSFSRVGSSNFRGGLGGGYGGASGMGGITAVTVNQ
SLLSPLVLEVDPNIQAVRTQEKEQIKTLNNKFASFIDKVRFLEQQNKMLETKWSLLQQQKTARSNMDNMF
ESYINNLRRQLETLGQEKLKLEAELGNMQGLVEDFKNKYEDEINKRTEMENEFVLIKKDVDEAYMNKVEL
ESRLEGLTDEINFLRQLYEEEIRELQSQISDTSVVLSMDNSRSLDMDSIIAEVKAQYEDIANRSRAEAES
MYQIKYEELQSLAGKHGDDLRRTKTEISEMNRNISRLQAEIEGLKGQRASLEAAIADAEQRGELAIKDAN
AKLSELEAALQRAKQDMARQLREYQELMNVKLALDIEIATYRKLLEGEESRLESGMQNMSIHTKTTSGYA
GGLSSAYGGLTSPGLSYSLGSSFGSGAGSSSFSRTSSSRAVVVKKIETRDGKLVSESSDVLPK

>Hs_K71
MSRQFTCKSGAAAKGGFSGCSAVLSGGSSSSFRAGSKGLSGGFGSRSLYSLGGVRSLNVASGSGKSGGYG
FGRGRASGFAGSMFGSVALGPVCPTVCPPGGIHQVTVNESLLAPLNVELDPEIQKVRAQEREQIKALNNK
FASFIDKVRFLEQQNQVLETKWELLQQLDLNNCKNNLEPILEGYISNLRKQLETLSGDRVRLDSELRNVR
DVVEDYKKRYEEEINKRTAAENEFVLLKKDVDAAYANKVELQAKVESMDQEIKFFRCLFEAEITQIQSHI
SDMSVILSMDNNRNLDLDSIIDEVRTQYEEIALKSKAEAEALYQTKFQELQLAAGRHGDDLKNTKNEISE
LTRLIQRIRSEIENVKKQASNLETAIADAEQRGDNALKDARAKLDELEGALHQAKEELARMLREYQELMS
LKLALDMEIATYRKLLESEECRMSGEFPSPVSISIISSTSGGSVYGFRPSMVSGGYVANSSNCISGVCSV
RGGEGRSRGSANDYKDTLGKGSSLSAPSKKTSR

>Hs_K72
MSRQLTHFPRGERLGFSGCSAVLSGGIGSSSASFRARVKGSASFGSKSLSCLGGSRSLALSAAARRGGGR
LGGFVGTAFGSAGLGPKCPSVCPPGGIPQVTVNKSLLAPLNVEMDPEIQRVRAQEREQIKALNNKFASFI
DKVRFLEQQNQVLETKWNLLQQLDLNNCRKNLEPIYEGYISNLQKQLEMLSGDGVRLDSELRNMQDLVED
YKKRYEVEINRRTAAENEFVVLKKDVDAAYMNKVELQAKVDSLTDEIKFFKCLYEGEITQIQSHISDTSI
VLSMDNNRDLDLDSIIAEVRAQYEEIALKSKAEAETLYQTKIQELQVTAGQHGDDLKLTKAEISELNRLI
QRIRSEIGNVKKQCADLETAIADAEQRGDCALKDARAKLDELEGALHQAKEELARMLREYQELVSLKLAL
DMEIATYRKLLESEECRMSGEYPNSVSISVISSTNAGAGGAGFSMGFGASSSYSYKTAAADVKTKGSCGS
ELKDPLAKTSGSSCATKKASR

>Hs_K73
MSRQFTYKSGAAAKGGFSGCSAVLSGGSSSSYRAGGKGLSGGFSSRSLYSLGGARSISFNVASGSGWAGG
YGFGRGRASGFAGSMFGSVALGSVCPSLCPPGGIHQVTINKSLLAPLNVELDPEIQKVRAQEREQIKVLN
NKFASFIDKVRFLEQQNQVLETKWELLQQLDLNNCKNNLEPILEGYISNLRKQLETLSGDRVRLDSELRS
VREVVEDYKKRYEEEINKRTTAENEFVVLKKDVDAAYTSKVELQAKVDALDGEIKFFKCLYEGETAQIQS
HISDTSIILSMDNNRNLDLDSIIAEVRAQYEEIARKSKAEAEALYQTKFQELQLAAGRHGDDLKHTKNEI
SELTRLIQRLRSEIESVKKQCANLETAIADAEQRGDCALKDARAKLDELEGALQQAKEELARMLREYQEL
LSVKLSLDIEIATYRKLLEGEECRMSGEYTNSVSISVINSSMAGMAGTGAGFGFSNAGTYGYWPSSVSGG
YSMLPGGCVTGSGNCSPRGEARTRLGSASEFRDSQGKTLALSSPTKKTMR

>Hs_K74
MSRQLNIKSSGDKGNFSVHSAVVPRKAVGSLASYCAAGRGAGAGFGSRSLYSLGGNRRISFNVAGGGVRA
GGYGFRPGSGYGGGRASGFAGSMFGSVALGPACLSVCPPGGIHQVTVNKSLLAPLNVELDPEIQKVRAQE
REQIKVLNDKFASFIDKVRFLEQQNQVLETKWELLQQLDLNNCKKNLEPILEGYISNLRKQLETLSGDRV
RLDSELRSMRDLVEDYKKRYEVEINRRTTAENEFVVLKKDADAAYAVKVELQAKVDSLDKEIKFLKCLYD
AEIAQIQTHASETSVILSMDNNRDLDLDSIIAEVRMHYEEIALKSKAEAEALYQTKIQELQLAASRHGDD
LKHTRSEMVELNRLIQRIRCEIGNVKKQRASLETAIADAEQRGDNALKDAQAKLDELEGALHQAKEELAR
MLREYQELMSLKLALDMEIATYRKLLEGEECRMSGENPSSVSISVISSSSYSYHHPSSAGVDLGASAVAG
SSGSTQSGQTKTTEARGGDLKDTQGKSTPASIPARKATR

>Hs_K75
MSRQSSITFQSGSRRGFSTTSAITPAAGRSRFSSVSVARSAAGSGGLGRISSAGASFGSRSLYNLGGAKR
VSINGCGSSCRSGFGGRASNRFGVNSGFGYGGGVGGGFSGPSFPVCPPGGIQEVTVNQSLLTPLHLQIDP
TIQRVRAEEREQIKTLNNKFASFIDKVRFLEQQNKVLETKWALLQEQGSRTVRQNLEPLFDSYTSELRRQ
LESITTERGRLEAELRNMQDVVEDFKVRYEDEINKRTAAENEFVALKKDVDAAYMNKVELEAKVKSLPEE
INFIHSVFDAELSQLQTQVGDTSVVLSMDNNRNLDLDSIIAEVKAQYEDIANRSRAEAESWYQTKYEELQ
VTAGRHGDDLRNTKQEISEMNRMIQRLRAEIDSVKKQCSSLQTAIADAEQRGELALKDARAKLVDLEEAL
QKAKQDMARLLREYQELMNIKLALDVEIATYRKLLEGEECRLSGEGVSPVNISVVTSTLSSGYGSGSSIG
GGNLGLGGGSGYSFTTSGGHSLGAGLGGSGFSATSNRGLGGSGSSVKFVSTTSSSQKSYTH

>Hs_K76
MNRQVCKKSFSGRSQGFSGRSAVVSGSSRMSCVARSGGAGGGACGFRSGAGSFGSRSLYNLGSNKSISIS
VAAGSSRAGGFGGGRSSCGFAGGYGGGFGGSYGGGFGGGRGVGSGFGGAGGFGGAGGFGGPGVFGGPGSF
GGPGGFGPGGFPGGIQEVIVNQSLLQPLNVEIDPQIGQVKAQEREQIKTLNNKFASFIDKVRFLEQQNKV
LETKWELLQQQTTGSGPSSLEPCFESYISFLCKQLDSLLGERGNLEGELKSMQDLVEDFKKKYEDEINKR
TAAENEFVGLKKDVDAAFMNKVELQAKVDSLTDEVSFLRTLYEMELSQMQSHASDTSVVLSMDNNRCLDL

GSIIAEVRAQYEEIAQRSKSEAEALYQTKLGELQTTAGRHGDDLRNTKSEIMELNRMIQRLRAEIENVKK
QNANLQTAIAEAEQRGEMALKDANAKLQDLQTALQKAKDDLARLLRDYQELMNVKLALDVEIATYRKLLE
GEECRMSGECQSAVCISVVSNVTSTSGSSGSSRGVFGGVSGSGSGGYKGGSSSSSSSGYGVSGGSGSGYG
GVSSGSTGGRGSSGSYQSSSSGSRLGGAGSISVSHSGMGSSSGSIQTSGGSGYKSGGGGSTSIRFSQTTS
SSQHSSTK

>Hs_K77
MSHQFSSQSAFSSMSRRVYSTSSSAGSGGGSPAVGSVCYARGRCGGGGYGIHGRGFGSRSLYNLGGSRSI
SINLMGRSTSGFCQGGGVGGFGGGRGFGVGSTGAGGFGGGGFGGAGFGTSNFGLGGFGPYCPPGGIQEVT
INQSLLEPLHLEVDPEIQRIKTQEREQIMVLNNKFASFIDKVRFLEQQNQVLQTKWELLQQVNTSTGTNN
LEPLLENYIGDLRRQVDLLSAEQMRQNAEVRSMQDVVEDYKSKYEDEINKRTGSENDFVVLKKDVDAAYV
SKVDLESRVDTLTGEVNFLKYLFLTELSQVQTHISDTNVILSMDNNRSLDLDSIIDAVRTQYELIAQRSK
DEAEALYQTKYQELQITAGRHGDDLKNSKMEIAELNRTVQRLQAEISNVKKQIEQMQSLISDAEERGEQA
LQDAWQKLQDLEEALQQSKEELARLLRDYQAMLGVKLSLDVEIATYRQLLEGEESRMSGELQSHVSISVQ
NSQVSVNGGAGGGGSYGSGGYGGGSGGGYGGGRSYRGGGARGRSGGGYGSGCGGGGGSYGGSGRSGRGSS
RVQIIQTSTNTSHRRILE

>Hs_K78
MSLSPCRAQRGFSARSACSARSRGRSRGGFSSRGGFSSRSLNSFGGCLEGSRGSTWGSGGRLGVRFGEWS
GGPGLSLCPPGGIQEVTINQNLLTPLKIEIDPQFQVVRTQETQEIRTLNNQFASFIDKVRFLEQQNKVLE
TKWHLLQQQGLSGSQQGLEPVFEACLDQLRKQLEQLQGERGALDAELKACRDQEEEYKSKYEEEAHRRAT
LENDFVVLKKDVDGVFLSKMELEGKLEALREYLYFLKHLNEEELGQLQTQASDTSVVLSMDNNRYLDFSS
IITEVRARYEEIARSSKAEAEALYQTKYQELQVSAQLHGDRMQETKVQISQLHQEIQRLQSQTENLKKQN
ASLQAAITDAEQRGELALKDAQAKVDELEAALRMAKQNLARLLCEYQELTSTKLSLDVEIATYRRLLEGE
ECRMSGECTSQVTISSVGGSAVMSGGVGGGLGSTCGLGSGKGSPGSCCTSIVTGGSNIILGSGKDPVLDS
CSVSGSSAGSSCHTILKKTVESSLKTSITY

>Hs_K79
MRSSVSRQTYSTKGGFSSNSASGGSGSQARTSFSSVTVSRSSGSGGGAHCGPGTGGFGSRSLYNLGGHKS
ISVSVAGGALLGRALGGFGFGSRAFMGQGAGRQTFGPACPPGGIQEVTVNQSLLTPLHVEIDPEIQRVRT
QEREQIKTLNNKFASFIDKVRFLEQQNKVLETKWALLQEQGQNLGVTRNNLEPLFEAYLGSMRSTLDRLQ
SERGRLDSELRNVQDLVEDFKNKYEDEINKHTAAENEFVVLKKDVDAAYMGRMDLHGKVGTLTQEIDFLQ
QLYEMELSQVQTHVSNTNVVLSMDNNRNLDLDSIIAEVKAQYELIAQRSRAEAEAWYQTKYEELQVTAGK
HGDNLRDTKNEIAELTRTIQRLQGEADAAKKQCQQLQTAIAEAEQRGELALKDAQKKLGDLDVALHQAKE
DLTRLLRDYQELMNVKLALDVEIATYRKLLESEESRMSGECPSAVSISVTGNSTTVCGGGAASFGGGISL
GGSGGATKGGFSTNVGYSTVKGGPVSAGTSILRKTTTVKTSSQRY

>Hs_K80
MACRSCVVGFSSLSSCEVTPVGSPRPGTSGWDSCRAPGPGFSSRSLTGCWSAGTISKVTVNPGLLVPLDV
KLDPAVQQLKNQEKEEMKALNDKFASLIGKVQALEQRNQLLETRWSFLQGQDSAIFDLGHLYEEYQGRLQ
EELRKVSQERGQLEANLLQVLEKVEEFRIRYEDEISKRTDMEFTFVQLKKDLDAECLHRTELETKLKSLE
SFVELMKTIYEQELKDLAAQVKDVSVTVGMDSRCHIDLSGIVEEVKAQYDAVAARSLEEAEAYSRSQLEE
QAARSAEYGSSLQSSRSEIADLNVRIQKLRSQILSVKSHCLKLEENIKTAEEQGELAFQDAKTKLAQLEA
ALQQAKQDMARQLRKYQELMNVKLALDIEIATYRKLVEGEEGRMDSPSATVVSAVQSRCKTAASRSGLSK
APSRKKKGSKGPVIKITEMSEKYFSQESEVSE

>Hs_K81
MTCGSGFGGRAFSCISACGPRPGRCCITAAPYRGISCYRGLTGGFGSHSVCGGFRAGSCGRSFGYRSGGV
CGPSPPCITTVSVNESLLTPLNLEIDPNAQCVKQEEKEQIKSLNSRFAAFIDKVRFLEQQNKLLETKLQF
YQNRECCQSNLEPLFEGYIETLRREAECVEADSGRLASELNHVQEVLEGYKKKYEEEVSLRATAENEFVA
LKKDVDCAYLRKSDLEANVEALIQEIDFLRRLYEEEILILQSHISDTSVVVKLDNSRDLNMDCIIAEIKA
QYDDIVTRSRAEAESWYRSKCEEMKATVIRHGETLRRTKEEINELNRMIQRLTAEVENAKCQNSKLEAAV
AQSEQQGEAALSDARCKLAELEGALQKAKQDMACLIREYQEVMNSKLGLDIEIATYRRLLEGEEQRLCEG
IGAVNVCVSSSRGGVVCGDLCVSGSRPVTGSVCSAPCNGNVAVSTGLCAPCGQLNTTCGGGSCGVGSCGI
SSLGVGSCGSSCRKC

>Hs_K82
MSYHSFQPGSRCGSQSFSSYSAVMPRMVTHYAVSKGPCRPGGGRGLRALGCLGSRSLCNVGFGRPRVASR
CGGTLPGFGYRLGATCGPSACITPVTINESLLVPLALEIDPTVQRVKRDEKEQIKCLNNRFASFINKVRF
LEQKNKLLETKWNFMQQQRCCQTNIEPIFEGYISALRRQLDCVSGDRVRLESELCSLQAALEGYKKKYEE
ELSLRPCVENEFVALKKDVDTAFLMKADLETNAEALVQEIDFLKSLYEEEICLLQSQISETSVIVKMDNS
RELDVDGIIAEIKAQYDDIASRSKAEAEAWYQCRYEELRVTAGNHCDNLRNRKNEILEMNKLIQRLQQET
ENVKAQRCKLEGAIAEAEQQGEAALNDAKCKLAGLEEALQKAKQDMACLLKEYQEVMNSKLGLDIEIATY
RRLLEGEEHRLCEGIGPVNISVSSSKGAFLYEPCGVSTPVLSTGVLRSNGGCSIVGTGELYVPCEPQGLL
SCGSGRKSSMTLGAGGSSPSHKH

>Hs_K83
MTCGFNSIGCGFRPGNFSCVSACGPRPSRCCITAAPYRGISCYRGLTGGFGSHSVCGGFRAGSCGRSFGY
RSGGVCGPSPPCITTVSVNESLLTPLNLEIDPNAQCVKQEEKEQIKSLNSRFAAFIDKVRFLEQQNKLLE
TKLQFYQNRECCQSNLEPLFAGYIETLRREAECVEADSGRLASELNHVQEVLEGYKKKYEEEVALRATAE
NEFVALKKDVDCAYLRKSDLEANVEALIQEIDFLRRLYEEEIRILQSHISDTSVVVKLDNSRDLNMDCIV
AEIKAQYDDIATRSRAEAESWYRSKCEEMKATVIRHGETLRRTKEEINELNRMIQRLTAEVENAKCQNSK
LEAAVAQSEQQGEAALSDARCKLAELEGALQKAKQDMACLIREYQEVMNSKLGLDIEIATYRRLLEGEEQ
RLCEGVEAVNVCVSSSRGGVVCGDLCVSGSRPVTGSVCSAPCNGNLVVSTGLCKPCGQLNTTCGGGSCGQ
GRH

>Hs_K84
MSCRSYRVSSGHRVGNFSSCSAMTPQNLNRFRANSVSCWSGPGFRGLGSFGSRSVITFGSYSPRIAAVGS
RPIHCGVRFGAGCGMGFGDGRGVGLGPRADSCVGLGFGAGSGIGYGFGGPGFGYRVGGVGVPAAPSITAV
TVNKSLLTPLNLEIDPNAQRVKKDEKEQIKTLNNKFASFIDKVRFLEQQNKLLETKWSFLQEQKCIRSNL

EPLFESYITNLRRQLEVLVSDQARLQAERNHLQDVLEGFKKKYEEEVVCRANAENEFVALKKDVDAAFMN
KSDLEANVDTLTQEIDFLKTLYMEEIQLLQSHISETSVIVKMDNSRDLNLDGIIAEVKAQYEEVARRSRA
DAEAWYQTKYEEMQVTAGQHCDNLRNIRNEINELTRLIQRLKAEIEHAKAQRAKLEAAVAEAEQQGEATL
SDAKCKLADLECALQQAKQDMARQLCEYQELMNAKLGLDIEIATYRRLLEGEESRLCEGVGPVNISVSSS
RGGLVCGPEPLVAGSTLSRGGVTFSGSSSVCATSGVLASCGPSLGGARVAPATGDLLSTGTRSGSMLISE
ACVPSVPCPLPTQGGFSSCSGGRSSSVRFVSTTTSCRTKY

>Hs_K85
MSCRSYRISSGCGVTRNFSSCSAVAPKTGNRCCISAAPYRGVSCYRGLTGFGSRSLCNLGSCGPRIAVGG
FRAGSCGRSFGYRSGGVCGPSPPCITTVSVNESLLTPLNLEIDPNAQCVKQEEKEQIKSLNSRFAAFIDK
VRFLEQQNKLLETKWQFYQNQRCCESNLEPLFSGYIETLRREAECVEADSGRLASELNHVQEVLEGYKKK
YEEEVALRATAENEFVVLKKDVDCAYLRKSDLEANVEALVEESSFLRRLYEEEIRVLQAHISDTSVIVKM
DNSRDLNMDCIIAEIKAQYDDVASRSRAEAESWYRSKCEEMKATVIRHGETLRRTKEEINELNRMIQRLT
AEIENAKCQRAKLEAAVAEAEQQGEAALSDARCKLAELEGALQKAKQDMACLLKEYQEVMNSKLGLDIEI
ATYRRLLEGEEHRLCEGVGSVNVCVSSSRGGVSCGGLSYSTTPGRQITSGPSAIGGSITVVAPDSCAPCQ
PRSSSFSCGSSRSVRFA

>Hs_K86
MTCGSYCGGRAFSCISACGPRPGRCCITAAPYRGISCYRGLTGGFGSHSVCGGFRAGSCGRSFGYRSGGV
CGPSPPCITTVSVNESLLTPLNLEIDPNAQCVKQEEKEQIKSLNSRFAAFIDKVRFLEQQNKLLETKLQF
YQNRECCQSNLEPLFEGYIETLRREAECVEADSGRLASELNHVQEVLEGYKKKYEEEVSLRATAENEFVA
LKKDVDCAYLRKSDLEANVEALIQEIDFLRRLYEEEIRVLQSHISDTSVVVKLDNSRDLNMDCIIAEIKA
QYDDIVTRSRAEAESWYRSKCEEMKATVIRHGETLRRTKEEINELNRMIQRLTAEVENAKCQNSKLEAAV
AQSEQQGEAALSDARCKLAELEGALQKAKQDMACLIREYQEVMNSKLGLDIEIATYRRLLEGEEQRLCEG
VGSVNVCVSSSRGGVVCGDLCASTTAPVVSTRVSSVPSNSNVVVGTTNACAPSARVGVCGGSCKRC

>Bt_K1
MSRHFSSRSGYRCGGGGGGFSSGSAGVVSYQRRSTSSSVRRSGGGGGRFSGGVCGGGAGGGFGSRSLINL
GGSKSISISVAGGGGRGGFGGGYGGGYGGGGFGGGGFGGGGFGAGGFGVGGIGGGFGGGGFGGGGFGGGG
FPVCPGGIQEVTINQSLLQPLNVEIDPEIQKIKSREREQIKSLNNQFASFIDKVRFLEQQNQVLQTKWEL
LQQINTSTRTYSLEPLFEAYISRLRRTVDQLKSDQSRLDSELKNMQDLVEDYRRKYEDEINKRTNAENEF
VGIKNDVDAAYITKVDLQAKFDNLLQEIDFYKTLYQAELSQMQTHISDTNVILSMDNNRNLDLNSIIDEV
KAQYEEIAQRSKAEAEALYQTKYEELQITAGQHGDSLKNTKVEISELNRVIQRLRSEIDSVKKQISSLQQ
AISDAEQRGENAIKDAQNKLNELEDALQKAKEDMARLLRDYQELMNTKLALDVEIATYRTLLEGEESRMS
GECVPNVSVSVSTSHTSISGGGGRGGVGFSSGGGGYGSGGGGGYGSGGGSSYGSRAGGSYGSGGGSSGSR
RGGSGGGGGGSSGGSFISSGGRASSTKTSGGSSSVKFVSTSYSRGPR

>Bt_K2
MSCQISYQSRGRGGGGGGFRGFSSGSAVVPGRSRLSGTSFSCLSRHGGGGGGFGGGGFGGGGFSSRSLVN
LGGSRSTSISVAGGGGSFGSYGGFGGRGGGFGGGLGVGGGSFGGGRFGGGGGFGGLGGPGGFGGPGGFGP
GGFPGGGIHEVSINQSLLQPLNVKVDPEIQNVKSQEREQIKALNNKFATFIDKVRFLEQQNQVLQTKWEL
LQQINVGTRTTNLEPLFQGYISQLQSHLDKLYSERMLQESELNNMQDLVEDFKKKYEDEVNKRTAAENEF
VTLKKDVDHAYTNKVELQAKVDLLRQDVEFTKMLFDAVSSEMQTXXXXXXXXXSMDNNRSLDLDSIITEV
RAQYEEIAQRSKAEAEAVYHSKYEELRITAGKHGDSLKEVKMEISELNRMIQRLQGEIAQVKKQCKNVQD
SIADAEQRGENALKDAQSKLNDLEEALQKAREDLARLLRDYQDLMNTKLSLDVEIATYRKLLEGEECRMS
GDLSSNVTVSVTSSSISSGVASRAGFGGYGSGGRGSSYGGGFSSGSRSYSSGGRRSGSRSGGGGSYGSGG
GSRGGSSSGGGYSSGGKHSSGGGARGGSSSGGGGYGSERGGSGSCEGGSSSVTFSFR

>Bt_K3
MNRQVRKTSGSGSQGFSGRSAVVSGSSRMSCVARSGGGGGGAFGFRSGAGGFGSRSLYNLGGNKSISISV
AGSRAGGFGGGRSSCVSGFGSGYGGGYGGGFGGGRGMGGGFGGAGGFGGAGGFGGAGGFGGLGGFGGPGG
FGPGGFGPGGFPGGIQEVTVNQSLLQPLNVEIDPQIGQVKTQEREQIKTLNNKFASFIDKVRFLEQQNKV
LETKWSLLQQQGTHPITGTNNLEPLFENYINSLRSYLDSILGERGRLDSELRNMQDLVEDFKKKYEDEIN
KRTAAENEFVTLKKDVDAAYMNKVELQAKVDALTDEINFLTTLYDMELSQMQSHVSDTSVVLSMDNNRSL
DLDSIIAEVKAQYEEIAQRSKAEAEALYQTKLGELQTTAGRHGDDLKSTKSEISELNRVIQRLRAEIENV
KKQIAKLQSAIAEAEQRGELALKDANAKLQELQAALQQAKDDLARLLRDYQELMNVKLALDVEIATYRKL
LEGEECRMSGECPSAVSISVVSSSSTTSASAGGFGGGYGGGVGVGGGARSGFGGGSGFGGGSGFGGGSGF
GGGSGISGSSGFGGGSGSGFGGGSGFSGSSGFGGGSSGFGSGSGGRSGVSGGGLSSGSSRGGSVRFSQSS
QRTSR

>Bt_K4
MIARQQYVRGGPRGFSCGSAIVGGVKKAAFSSASMSGGAGRCSSGGFGSRSLYNLGGNKSISISMAGCRQ
GAGFGAAGGFGAAGGFGSGFGAGGFGSGFGGSFGGRGGAGFPVCPAGGIQEVTINQSLLTPLHVEIDPEI
QKVRTEEREQIKLLNNKFASFIDKVRFLEQQNKVLETKWKLLQQQTTTTSVKNLEPFFEAYINALRKQVD
SLANDKGRLQSELKIMQDSVEDYKTKYEDEINKRAAAENDFVVLKKDVDAAYMNKVELEAKVDALNDEIN
FLRVLYAAELSQMQTQVSDTSVVLSMDNNRNLDLDSIIAEVRAQYEEIAQRSKAEAEALYQIKVQQLQTS
VDQHGDSLRNTKNEISELNRLIQRLRAEIENVKKQCQTLQGSVADAEQRGEVALKDAYSKRTELEAALQK
AKEELARVLREYQELMSVKLALDIEIATYRKLLEGEECRMSGECQSAVSISVVGGAASAGGLGGALGGSS
GFGLGSGSCAVGLGGGLGGSSGFGLGSGSGSGFGFGGGIGGSSSGKIISTTTVSKKSFR

>Bt_K5
MSRQSTVSFRSGGGRSFSTASAITPSVSRTSFTSVSRSGGGGGGGFGRVSLGGAYGAGGFGSRSLYNLGG
SKRISISASGGGFRNRFGAGAGGGYGFGGGAGSGFGFGGGAGGGGFGLGGGAGFGGGFGGPGFPVCPPGG
IQEVTVNQSLLTPLNLQIDPTIQRVRTEEREQIKTLNNKFASFIDKVRFLEQQNKVLDTKWALLQEQGTK
TVRQNLEPLLEQYINNLRRQLDGIVGERGRLDSELRNMQDLVEDFKNKYEDEINKRTTAENEFVMLKKDV
DAAYMNKVELEAKVDALMDEINFMKMFFDAELSQMQTHVSDTSVVLSMDNNRSLDLDSIIAEVKAQYEDI
ANRSRTEAESWYQTKYEELQQTAGRHGDDLRNTKHEISEMNRMIQRLRSEIDNVKKQCANLQNAIADAEQ
RGELALKDARSKLAELEDALQKAKQDMARLLREYQELMNTKLALDVEIATYRKLLEGEECRLSGEGVGPV
NISVVTNTVSSGYGGGSGFGGGLGGGLGGGLGGGLGGGLGGGLGSGLGGGGSSSFYSSSSGGVGLGGGLS
VGGSGFSASSGRSLGFGSGGGSSSSVKFVSTTSSSRKSFKS

>Bt_K6a
MSYKSTVKTQSISRKGFSAGSARVPGVCRSGFSSVSLSRSRGSGGLAGVCGGAGFGSRSLYGLGGSKRIS
IAGGSCAIGGGYGGRIGVGYGFGGGVGSGFGFGAGAGSGFGLGGGAGFGGGFGGPGFLVCPPGGIQEVTV
NQSLLTPLNLQIDPTIQRVRTEEREQIKTLNNKFASFIDKVRFLEQQNKVLDTKWTLLQEQGTKTVRQNL
EPLFEQYINNLRRQLDSILGERGRLDSELRGMQDTVEDFKNKYEDEINKRTVAENEFVNLKKDVDAAYMN
KVELQAKVDALTDEINFLRTFYDAELAQMQTHISDTSVVLSMDNNRNLDLDSIIAEVKAQYEEIAQRSRA
EAESWYQSKYEELQVTAGRHGDDLRNTKQEISEINRVIQRLRSEIDHVKKQCASLQSAIADAEQRGELAL
KDARSKLADLEDALQKAKQDMARLLKEYQELMNVKLALDVEIATYRKLLEGEECRLSGEGVGQVNISVVQ
STVSGGYGGAGGYGGASGLGSGLGVSGGSGYSYSSGHSLGGGFSSGSGRAIGCGFGSSGGSSSTIKYTTT
TSSSSRKGYKH

>Bt_K6b
MSCKSTVKTQSISRRGFSAGSARVPGVCRSGFSSVSLSRSRGSGGLAGVCGGAGFGSRSLYGLGGSKRIS
IAGGSCVIGGGYGGRIGVGYGFGGGAGSGFGFGAGAGSGFGLGGGAGFGGGFGGPGFPVCPPGGIQEVTV
NQSLLTPLNLQIDPTIQRVRTEEREQIKTLNNKFASFIDKVRFLEQQNKVLDTKWTLLQEQGTRTVRQNL
EPLFEQYINNLRRQLDSILGERGRLDSELRGMQDTVEDFKNKYEDEINKRTAAENEFVKLKKDVDIAYMN
KVELQAKVDALTDEINFLRTFYDAELAQMQTHISDTSVVLSMDNNRNLDLDSIIAEVKAQYEEIAQRSRA
EAESWYQSKYEELRVTAGRHGDDLRNTKQEISEINRVIQRLRSEIDHVKKQCASLQSAIADAEQRGELAL
KDARSKLADLEDALQKAKQDMARLLKEYQELMNVKLALDVEIATYRKLLEGEECRLSGEGVGQVNISVVQ
STVSGGYGGAGGYGGASGLGSGLGVSGGSGYSYSSGHSLGGGFSSGSGRAIGCGFGSSGGSSSTIKYTTT
TSSSSRKGYKH

>Bt_K6c
MSCKSTVKTQSISRRGFSAGSARVPGVCRSGFSSVSLSRSRGSGGLAGVCGGAGFGSRSLYGLGGSKRIS
IAGGSCVIGGGYGGRIGVGYGFGGGAGSGFGFGAGAGSGFGLGGGAGFGGGFGGPGFPVCPPGGIQEVTV
NQSLLTPLNLQIDPTIQRVRTEEREQIKTLNNKFASFIDKVRFLEQQNKVLDTKWTLLQEQGTRTVRQNL
EPLFEQYINNLRRQLDSILGERGRLDSELRGMQDTVEDFKNKYEDEINKRTAAENEFVKLKKDVDIAYMN
KVELQAKVDALTDEINFLRTFYDAELAQMQTHISDTSVVLSMDNNRNLDLDSIIAEVKAQYEEIAQRSRA
EAESWYQSKYEELRVTAGRHGDDLRNTKQEISEINRVIQRLRSEIDHVKKQCASLQSAIADAEQRGELAL
KDARNKLAGLEDALQKAKQDMARLLKEYQELMNVKLALDVEIATYRKLLEGEECRLSGEGVGQVNISVVQ
STVSGGYGGAGGYGGASGLGSGLGVSGGSGYSYSSGHSLGGGFSSGSGRAIGCGFGSSGGSSSTIKYTTT
TSSSSRKGYKH

>Bt_K7
MSLHFGSQVFSSRSAAFPGRGTQVRLSSVRPGGFGSSSSLYGLGASRPRVAARSSYGAPVGTGIRAVTIN
QSLLTPLQVDIDPSIQQVRQEEREQIKTLNNKFASFIDKVRFLEQQNKLLETKWALLQEQKSAKSNRLPG
IFEAQIAGLRKQLEALQLDGGRLEVELRNMQDVVEDFKNKYEDEINHRTAAENEFVVLKKDVDVAYMNKV
ELEAKVDTLNDEINFLRTLYEQELKELQSEVSDTSVVLSMDNNRSLDLDSIIAEVKAQYEEIANRSRAEA
EACYQTKFETLQAQAGKHGDDLQNTRNEIADMNRAVQRLQAEIDSVKNQRSKLEAAIADAEQRGELAVKD
ARAKQEDLEAALQKAKQDMTRQLREYQELMNVKLALDIEIATYRKLLEGEESRLTGDGVGAVNISVVSST
GGSGSLLTFGGTMGNNALRFSSGGGPGTLKAYSMRTTSATSRSPRK

>Bt_K8
MSIRVTQKSYKVSTSAPRSFSSRSYTSGPGSRISSSAFSRVGSSSSFRGGLGTGMSMAGSYGGAPGLGGI
TAVTVNQSLLSPLKLEVDPNIQAVRTQEKEQIKTLNNKFASFIDKVRHLEQQNKVLETKWNLLQQQKTAR
SNIDNMFESYINNLRRQLETLAQEKLKLEVELGNMQGLVEDFKTKYEDEIQKRTDMENEFVIIKKDVDEA
YMNKVELESRLEGLTDEINFYRQLYEEEIREMQSQISDTSVVLSMDNNRNLDLDGIIAEVKAQYEEIANR
SRAEAEAMYQIKYEELQTLAGKHGDDLRRTKTEISEMNRNINRLQAEIEGLKGQRASLEAAIADAEQRGE
MAVKDAQAKLAELEAALRNAKQDMARQLREYQELMNVKLALDVEIATYRKLLEGEESRLESGMQNMSIHT
KTTSGYAGGLTSSYGTPGFNYSLSPGSFSRTSSKPVVVKKIETRDGKLVSESSDVLSK

>Bt_K71
MSRQFTCKSGAAAKGGFSGCSAVLSGGSTSSYRAGGKGLSGGFGSRSLYNLGGVRSISFNVASGSGKSGG
YGFGRGRASGFAGSMFGSVALGPMCPTVCPPGGIHQVTVNESLLAPLNVELDPEIQKVRAQEREQIKALN
NKFASFIDKVRFLEQQNQVLETKWELLQQLDLNNCKNNLEPILEGYISNLRKQLETLSGDRVRLDSELRS
VRDVVEDYKKRYEEEINRRTAAENEFVLLKKDVDAAYANKVELQAKVDSMDQEIKFFKCLYEAEIAQIQS
HISDMSVILSMDNNRDLNLDSIIDEVRAQYEDIALKSKAEAEALYQTKFQELQLAAGRHGDDLKNTKNEI
SELTRLIQRIRSEIENVKKQASNLETAIADAEQRGDNALKDARAKLDELEAALHQSKEELARMMREYQEL
MSLKLALDMEIATYRKLLESEECRMSGEFPSPVSISIISSTSGSGGYGFRPSSVSGGYVANSGSCISGVC
SVRGGESRSRSSTTDYKDALGKGSSLSAPSKKASR

>Bt_K72
MSRQLNLYPGGERLAFSGCSAIISSRVSSSTASFRASGIKGTATFGSRSLFNCGGGRRPALSSAAGRGGS
ALGSCAATGGGRRGGFVGTVFGSAGLGPACPSVCPPGGIPQVTVNKSLLSPLNVELDPEIQKVRAQEREQ
IKALNNKFASFIDKVRFLEQQNQVLGTKWELLQQLDLNNCKNNLEPILEGYTSNLRKQLEMLSGDRVRLD
SELRSMRDVVEEYKKRYEVEINRRTAAENEFVMLKKDVDAAYMNKVELQAKVDSLTDEIKFLKCLYEGEI
AQLQSHISDTSVILSMDNNRDLDLDSIIAQVRAQYEEIALKSKAEAEALYQTKIQELQATAGQHGDDLKL
TKAEISDLNRMIQRIRSEIGNVKKQCSNLEMAIADAEQRGDCALKDARAKLDELDAALLQAKEELARMMR
EYQELMSTKLALDMEIATYRKLLEGEECRMSGEYPNSVSISVISNTSTGAGGTGFSMGFGALSSYSYKSS
AVDVKTKGSCGGSELKDAPAKTSGSSCATKKASR

>Bt_K73
MSRQFTYKSGAAAKGAFSGCSVVLSGSSSPSYRAGSKGLSGGFGSRSLYSLGCARSVSFNMASGSGRAGG
YGFSRGRASGFAGSMFGSVALGPMCPSLCPPGGIHQVIVNKSLLAPLNVELDPEIQKVCAQEREQIKALN
NKFASFIDKVRFLEQQNQVLGTKWELLQQQDLDNCKNNLEPILEGYISNLRKQLEMLSGDRVRLDSELRS
MRDVVEDYKKRYEEEINKRTTAENEFVVLKKDVDAAYMSKVELQAKVDALEREIKFFTCLYEGEIAQMQS
HISDTSVILSMDNNRNLDLNSIIAEVRAQYEDIALKSKAEAEALYQTKFQELQLAAGRHGDDLKHTKNEI
AELTRLIQRLRSETESVKKQCSNLETAITDAEQRGHCALKDAQAKLDELEAALLQAKEELARMMCEYQEL
MSTKLALDIEIATYRKLLEGEECRMSGEYTNSVSISVISSSMAGTAGTGAGFGYSGSGTYGYRPSSVGGG

YGFLLGGCVTGSGNCSPRGEAKTRLGSTSEIKDLLGKTPALSSPTKKTPR

>Bt_K74
MSRQLNIKSGGDKGGFSGHSAVVLRKVGGSAASYRAPSKGAGAAFGSRSLYSLCRGDLCVPLKVAGSSVR
TGGYNFRLGSGYGGVRASSFAGSMFGSVVLGPVCPSMCPPGGIHQVTVNKSLLAPLNVELDPEIQKVRAQ
EREQIMALNNKFASFIDKVRFLEQQNQVLGTKWELLQQMDLNNCRKNLEPILEGYIGNLRKQLEMLSGDR
LRLDSELKGMRDLVEDYKKRYEVEINQRTAAENDFVVLKKDADAAYTVKVELQAKVDSLDKDIKFLKCLY
DAEVAQIQTHTSETSVILSMDNNRYLDLDSIIAEVRAQYEDIALKSKAEAEALYQSKIQELQLAAGRHGD
DLKHTKNEMSELNRLIQRIRCEIANVKKQCANLETAIADAEQRGDSALKDARAKLDELEAAMHQAKEELA
RMLREYQELMSLKLALDMEIATYSKLLEGEECWMSGENPSSVSISVISSSASSFGYHPGSSASTDLGAST
MASTGTSSSSSTQSGQTRAKGARVGDPKDSQDKSTPVSSRARKAAR

>Bt_K75
MSRQSTITFQTSSRRGFSTASATTPATSRSRFSSASVTHSPAGSGGLGRISGFGSRSLYNLGGTKRVSIS
GCGSNFRSGFGGRASSGFGVSGGFGYGGGIGGGHGGCGFSVCPPGGIQEVTVNQSLLTPLNLQIDPNIQR
VRKEEREQIKTLNNKFASFIDKVRFLEQQNKVLETKWSLLQEQGTRTVRQSLEPFFEAYITDLRRQLDSI
TTERGRLDAELRTMQDVVEDFKVRYEDEINKRTAAENEFVALKKDVDAAYLNKVDLEAKANSLTDEINFL
QMLFEAELCQMQTRVSDTSVVLSMDNNRSLDLDSIIAEVKAQYEEIANRSRAEAESWYQTKYEELQVTAG
QHGDDLRNTKQEISETNRMIQRLRAEIDNVKKQCASLQTAIADAEQRGELALKDARAKLVDLEEALQKSK
QDMARLLREYQELMNIKLALDVEIATYRKLLEGEECRLSGEGVSPVNISVVTSTVSSGYGGGSSIGSGSL
GLSGGSGCSFMTSGGHSLGGSSFSNSSSRGLGGSGSSFKFVSTTSSSRKSYKH

>Bt_K76
MSRQAGYSQQSCRASSGSRRQGFSGHSAVVSGQSRVTSSKSSLASRSGGGGAGSAACALMGGGFGSQSLY
NLGGNKKISISLAGGSIRAGGFRSTSGGYSGGCGFGGGGRGMGGGFGGRAGGFGGGVGGFGGPGGFGGLG
GFGGPRGFGGPGGFGPGGFPGGIQEVTVNQSLLQPLNVEIDPQIGQVKAQEREQIKTLNNKFASFIDKVR
FLEQQNKVLETKWSLLQEQGSGTNTNNRNLEPFFENYISSLRAFLDGLHVEKDKLHEELRSMEGMVEDFK
KRYEEEINKRTAAENDFVVLKKDIDAAYMTKVELEAKVDSVTDEINFLKALYDAELSQMQLDTGDTSVVL
SMDNNRCLDLDSIIAEVRAQYEAIAQRSKAEAEALYQSKLGELQTTAGMHGDDLKSIKTEIMEFNRMIQR
LRAEIESVKKQNANLQTAIADAEQHGEVALKDANAKLQDLKAALQQAKEDLARLLKEYQELMNVKLALDI
EIATYRTLLEGEECRMSGECQSSVSIEMVHNTTSSSSGGSGALGGGAGGRGGLGSGGLGSGSLGSGRLGS
GGRDSRASRGGLGLDSSSGGGSAVRGGVSNSGGSCAVSGVGGRSSVRVTQSSSQSQRSHHKL

>Bt_K77
MSHQFSSQSAFSSRSRRVYSAGSSAGSGGGSRAVRSVCQARGRCGSGGGGYGSHARGFTSRSLYNLGGSK
SISISLVGRSASGFCQGGGAGGGFGGGRSFGGGGYGGGGLGGAGFRGGNLGLGGFGPSCPPGGIQEVTIN
QSLLQPLHLEVDPEIQRVKMQEREQIMVLNNKFASFIDKVRFLEQQNQVLQTKWELLQQVNTSTQTNNLE
PIFESYISKLQRQVDCLRAEQMRQSSEIRSMQDVVDDYKSKYEEEINRRTSSENDFVVLKKDVDSAYLSK
AELQSKADTLLEEVDFMKYLFQSELSQMQTNITDTNVILSMDNNRSLDLDSIIDAVRIQYEEIAQRSKDE
AEALYQTKFQELQITAGRHGDELKTSRMEISELNLTIQRLQAEIGNVKKQIEHMHTIISDAEERGQQALQ
DAQQKLQDLEAALQQSKEELARLLRDYQALVGAKLSLDVEIATYRKLLEGEESRMSGELQSQVSISVQSS
QLTIGGGARGSGGYSGGGGYGGGEGYAGGSSSGGYGGGGGSRGGSGGYGGGSGGASGGGYGSGGGSYGGS
SKSSSKYGGGSGDASRMQIIQTSTSTSHRRKLE

>Bt_K78
MSLSPCRARRGFSARSACSAQSVGRGRTGFSSRSLSSFGGCRGGSRGRTWGSWGRLGVRLGEGSGGPGLS
LCPPGGIQQVTINQSLLIPPKIEIDPQFQVVRTQETQQIRVLNNQFASFIDKVRFLEQQNKVLETKWHLL
QQQGLSDRPQGLESFFEAYLVRLRTQLEELQKKRGSLDAELKSCQGQEEEYKAKYEHEANRRATLENDFV
VLKKDADGVLLSKMEMESKVEDLKEYICFLKHLYEEELGQLQTQASDMSVVLSMDNNRCLDFRDLIAEVR
ARYEEIARTSKAEAEMLYQTKYRELQACAQLHGNSMKETKVQITQLQQTIKKLQSQIETVKSQNASLQVA
IADAEQRGELALKDAQTKLAELEAALRTAKQDIARLLHDYQELMSVKLSLDIEIATYRRLLEGEECRMSG
ECASQVTISAVGGGSTVVSGGADGGLAGTCGLGGVKGSFGSRCSSVVKGGSSIVKGGSSVVIGGSSIILG
SEQGPAVGSGSVSGSSSSSTSHTILKKTVESSLKTSVTY

>Bt_K79
MLGPVCPPGGIQQVTVNQSLLTPLHVEIDPEIQRVRTEEREQIKTLNNKFASFIDKVRFLEQQNKVLETK
WALLQEQGQKSGVTRNNLEPLFEHFINNLRGKLDNLQSERGRLDSELRNVQDLAEDFKTKYEDEINKRTA
AENEFVVLKKDVDAAYVGRMDLHGMVDHLMGEIDFLRHLYEEELSQVQTHVSDTSVILSMDNNRNLDLDS
IIAEVKAQYEQIAQRSRAEAESWYQTKYEELQVTAGKHGDNLRDTKNEIAELTRTVQRLQGEADAVKKQC
QQLQTAIADAEQHGELALKDAQKKLGDLDAALNQAKEDLARLLRDYQALMNVKLALDVEIATYRKLLESE
ESRMSGECPSAVSISVTGNSTTVCGGGAAGFGGGISLGGGGGASKGRFSTNAGYSTVKGGPVSGGTSILR
KTTTVKTSSRRY

>Bt_K80
MACRSCVVGFSSLSSCEVTPAGSPRPATAGWSSCGPPEPGLSSHSLTGCWTAGTVSKVTVNPSLLVPLDL
KVDPAIQQQKNNEKEEMKVLNDKFASLIGKVQALEQRNQLLETRWHFLQSQDSATFDLGHLYEEYQGRLQ
EELRKVSKERGQLEANLLQVLEKVEDFRIRYEDEISKRTDMEFTFVQLKKDLDAECLRRTELETKLKGLQ
SFVELMKSIYEQELKDLAAQLKDVSVTVGMDSRCHIDLSGIVEEVKAQYDAVAARSLEEAEAYSRSQLEE
RAACSAEFENSLQSSRSEIADLNVRIQKLRSQILSIKSHCLKLEENIKVAEEQGELAFQDAKAKLAQLED
ALQQAKKDMARQLREYQELMNTKLALDIEIATYRKLMEGEESRMDMPSATVVSAVQARCRTAVSKSGLSR
APSRKKKDKKGPVIKITEMSEKFLSQESEVSE

>Bt_K81
MTCGSGFRGRAFSCVSACGPRPGRCCITAAPYRGISCYRGLTGGFGSRSICGGFRAGSFGRSFGYRSGGV
GGLNPPCITTVSVNESLLTPLNLEIDPNAQCVKQEEKEQIKCLNNRFAAFIDKVRFLEQQNKLLETKLQF
YQNRQCCESNLEPLFNGYIETLRREAECVEADSGRLSSELNSLQEVLEGYKKKYEEEVALRATAENEFVA
LKKDVDCAYLRKSDLEANVEALIQEIDFLRRLYEEEIRVLQAHISDTSVIVKMDNSRDLNMDNIVAEIKA
QYDDIASRSRAEAESWYRSKCEEIKATVIRHGETLRRTKEEINELNRVIQRLTAEVENAKCQNSKLEAAV
TQAEQQGEAALNDAKCKLAGLEEALQKAKQDMACLLKEYQEVMNSKLGLDIEIATYRRLLEGEEQRLCEG
VGSVNVCVSSSRGGVVCGDLCVSGSRPVTGSVCSAPCSGNLAVSTGLCAPCGPCNSVTSCGLGGISSCGV

```
GSCASVCRKC

>Bt_K82
MSCRSFQQGFRCGGQSFSSCSAVLPRVVTHYAVSQGPCRTGGSGSFRALGCLGSRSLCNVGFGQPRVASR
CGLPGFGYRAGTTCGSSACIAPVTINESLLVPLELEVDPTVQRVKRDEKEQIKCLNNRFASFINKVRFLE
QKNKLLETKWNFMQQQKCCSSNIEPIFEGYISTLRRQLDFLTGDRDRLESEFHCLHDTLEGYKKKYEEEL
SLRPTAENEFVALKKDVDTAFLINSDLEINVEALIHEINFLKTLYTEETNLLQSQISDTSVTVKMDNSRE
LDTDGIIAQIKAQYDEIANRSKAEAEAWYQSRYEELQLTAGNHCDNLRNRKNEILEINKLIQRLQQDIEN
VKAQRCKLEAAVTQAEQQGEAALNDAKCKLAGLEEALQKAKQDMACLLKEYQEVMNSKLGLDIEIATYRR
LLEGEEQRLCEGVGPVNISVSSSKGAILYEPCVVSTPVYCPGSTSVLKSSGGCGIVGTSEIYIPCEPQGL
LVCGSRRSSGVKLGAGGSASCHKC

>Bt_K83
MTCGFSTVGSGFGSRAFSCVSACGPRPGRCCITAAPYRGISCYRGLTGGFGSRSVCGGFRAGYCSRSFGY
RSGGVGGLSPPCITTVSVNESLLTPLNLEIDPNAQCVKQEEKEQIKCLNNRFAAFIDKVRFLEQQNKLLE
TKLQFYQNRQCCESNLEPLFEGYIETLRREAECVEADSGRLSSELNHVQEVLEGYKKKYEEEVALRATAE
NEFVALKKDVDCAYIRKSDLEANSEALIQEIDFLRRLYEEEIRVLQANISDTSVIVKMDNSRGLNMDNIV
AEIKAQYDDIASRSRAEAESWYRSKCEEIKATVIRHGETLRRTKEEINELNRLIQRLTAEVENAKCQNSK
LEAAVTQAEQQGEVALNDARCKLAGLEEALQKAKQDMACLLKEYQEVMNSKLGLDIEIATYRRLLEGEEQ
RLCEGVGAVNVCVSSSRGGVVCGDLCVSGSRPVTGSVCSAPCSGNLAVSTGLCAPCGQLNTTCGGGSCSL
GRC

>Bt_K84
MSCRSYRVSSGRCVGNFSSCSAVTPQNLNRFRASSVSCRSGPSFRGLSSFGSRSVISFGSCSPRIAAVCP
RPSCYGVGFGDGSGAGLGFGVGSCVGLGFGARSGLGYGFCNPGFGYRVGGIGGPAAPSITAVTVNQSLLT
PLNLEIDPNAQRVKRDEKEQIKTLNNKFASFIDKVRFLEQQNKLLETKWNFLQEQKCAKSNLEPLFENYI
TNLRRQLDVVNSDQARLEAERNHMQDVLEGFKKKYEEEVGLRASAENEFVALKKDVDTAFLSKADLEANV
DTLIQEIDFLKTLYEAEIQLLQSHISETSVIVKMDNSRDLNVDGIIADIKAQYEEIARRSRADAEAWYQT
KYEEMRVTAGQHCDNLRNTRDEMNELNRLIQRLKAEIEHAKAQRCKLEAAVAEAEQQGEAALNDAKCKLA
ELEGALQQAKQDMARQLKEYQELMNVKLALDIEIATYRRLLEGEEIRICEGVGPVDIAVSSSRGGLVCGP
ESLYSSSSLCRGGVIISGSGSSSNIRSSGFCSSSVGGARVVGGGDLLGAGSRGGSVLVGEVCAPSVPCPL
PTEGGFSSCSGGRSSRSSTVRFVSTTTSHRTKH

>Bt_K85
MSCRSYRISPGYSVTRTFSSCSAVAPKTGSRCCITAAPYRGVSCYRGLTGFGSRSVSALGSCGPRIAVGG
FRAGSCNRSFGYRSGGVGGLSPPCITTVSVNESLLTPLNLEIDPNAQCVKHEEKEQIKNLNSRFAAFIDK
VRFLEQQNKLLETKWQFYQNQRCCESNLEPLFNGYIETLRREAEHVEADGGRLASELNHVQEVLEGYKKK
YEEEVALRATAENEFVVLKKDVDCAYLRKSDLEANVEALVEESNFLKRLYDEEIQILNAHISDTSVIVKM
DNSRDLNMDCVVAEIKAQYDDIASRSRAEAESWYRNKCEEMKATVIRHGETLRRTKEEINELNRIIQRLT
AEIENAKCQRTKLEAAVAEAEQQGEAALNDARCKLAGLEEALQKAKQDMACLLKEYQEVMNSKLGLDIEI
ATYRRLLEGEEQRLCEGVGSVNVCVSSSRGGVTCGGLTYSTTAGRQIASGPVATGGSITVLAPDSCVPCQ
PRASSFSCGSSRSVRFA

>Bt_K86
MTCGSYRALPAFSCVSACGPRPGRCCITAAPYRGISCYRGLTGGFGSRSVCGGFRAGYCGRSFGYRSGGV
GGLSPPCITTVSVNESLLTPLNLEIDPNAQCVKQEEKEQIKCLNNRFAAFIDKVRFLEQQNKLLETKLQF
YQNRQCCESNLEPLFNGYIETLRREAECVEADSGRLSSELNSLQEVLEGYKKKYEEEVSLRATAENEFVA
LKKDVDCAYLRKSDLEANVEALIQEIDFLRRLYEEEIRVLQAHISDTSVIVKMDNSRDLNMDNIVAEIKA
HYDDIASRSRAEAESWYRSKCEEIKATVIRHGETLRRTKEEINELNRTIQRLTAEVENAKCQNSKLEAAV
TQAEQQGEAALNDAKCKLAGLEEALQKAKQDMACLLKEYQEVMNSKLGLDIEIATYRRLLEGEEQRLCEG
VGSVNVCVSSSRGGVVCGDLCASGAAPAVTTSVCSAPCSGNVVVGTSDACGPCSRVGGSILGCKKC

>Bt_K88
MTCGSACFSSRSGVRSSSSCVTTQRYAPGRTFSCVSACGPRPSRCCITAAPYRGVSCYRGLTGGFSSRSV
CGGSRAGSSSRSFGYRSGGPSPPCITSVSVNQSLLKPLNLEIDPNAQRIKHQEKEQIKSLNSKFAAFIDK
VCFLEQQNKLLETKWKFYQNQRCCESNLEPLFNGYIETLRREAERVEADTGRLASELNSLQEVLQGYKKK
YEEEVALKTTAENEFVKIKQEVNRAYVLKGDLEANAHSLVEEVCVLGLKTLYEEELRVMQAHISDTSVVV
KMDNSRYLNMDSIVAEIKAHYDEIASRSRAEAESWYRSKYEEIKATVIRHGETLRRTKEEINELNRLIQR
LTAEIENAKSQNSKLEAAVTQAEQQGEAALNDARGKLAGLEKTLQKAKQDMACLLKEYQEVMSAKLGLDI
EIATYRRLLEGEEQRLCEGISAVNVRVSSSRGGVVCGDLSATRTCGIGSYGVGACGSSCKKC

>Bt_K89
MASHSYNSSSYRVRDFSSCSAVVPKPGVHGFANGLAFHGGSPGGPGYRRLGGFGSRSLCAVGSPRIAVSY
AWPLRGGGSFGYQAGGLYGPIPPCITTVSVNESLLAPLNLEIDPKAQCVKHEEKEQIKGLNNKFAAFIDK
VRFLEQQNKLLETKLQFYQNHQCCESNLEPLFNGYIETLRREAECVEANSGRLASELNHVEEVLEGYKKK
YEEEVALKTTAENEFVVLKKDIDCAYLRKADLEANVEALKEEMSFLQSLYDEEIYLLQSQISDTSVVVKM
DNSRELNMDSVVAEIKAQYDGIASRSRAEVESWYQTKCEEMKVTVTQQGENLRRTKEEINELNRMIQRLT
AEVENAKQQRCKLETALAEAEQQGEAALNDAKCKLAGLEEALQKAKQDMACLLKEYQEVMNSKLGLDVEI
ATYRKLLEGEESRLCEGVGSINICVSRSQGGVICGDLDSTVPRGLGGTAISSSALCSPSVGGFCSSVRSV
RFA
```

**Suppl. Fig. S2. Amino acid sequences of intact keratins of cetaceans, cattle and human.** (**A**) Sequences of intact type 1 keratins. (**B**) Sequences of intact type 2 keratins. Abbreviations in sequence names: Bas, *Balaenoptera acutorostrata scammoni*; Bt, *Bos taurus*; Hs, *Homo sapiens*; Lv, *Lipotes vexillifer*; Oo, *Orcinus orca*; Pc, *Physeter catodon*; Tt, *Tursiops truncatus*; c, corrected; h, hypothetical, at least one exon of the gene was found only in whole genome shotgun (WGS) but not in the current genome sequence assembly of the GenBank (June 2018); p, partial sequence (at least one exon of the gene is neither found in the current genome sequence assembly nor in WGS). For details, see Suppl. Tables S1-S5. Unidentified amino acid residues are indicated by "X" in the FASTA sequences. The number of missing amino acid residues (X) is unknown.

**A**

```
                                         V  R  F  L  E  Q  Q  N  Q  V  L  R  T  K  W  E  L  L  Q  Q  I  D
Elephant Krt1    GTGAT-GGGTCTTTTGTTCTTAGGTGAGGTTCCTGGAGCAGCAGAACCAGGTGCTGCGAACAAAATGGGAGCTGCTGCAGCAGATAGAT
Manatee Krt1 GB  GTAACAGGGTCTTGTGTTCTTAAGTGAGGTTCCTGGAGCAGCAGAACCAGGTGCTACAAACAAAATGGGAGCTGCTGCAGCAGAAAGAC
Manatee Krt1 PCR GTAACAGGGTCTTGTGTTCTTAAGTGAGGTTCCTGGAGCAGCAGAACCAGGTGCTACAAACAAAATGGGAGCTGCTGCAGCAGATAGAC
```

Inactivation or shift of splice acceptor site * (premature stop codon if shifted splice site is functional)

**B**

```
                  G  G  G  R  P  L  G  F  S  S  G  S  A  L  A  F  C  G  S  R  R  S  G  F  S  F  P  C  L  S
Elephant Krt2    GGTGGAGGAAGACCCCTGGGCTTCAGCAGCGGCTCGGCTCTGGCCTTCTGTGGGAGCCGAAGATCAGGCTTCAGCTTCCCCTGCTTGAGC
Manatee Krt2 GB  GGTGGAGGAAGATTCTGGGGCTTCAGCAGTGGCTTGGCTGTGGTCTCTGGTGGGAGCCGGAGAT-GGGCCTCAGCTTCTCCTGCTTGAGC
Manatee Krt2 PCR GGTGGAGGAAGATTCTGGGGCTTCAGCAGTGGCTTGGCTGTGGTCTCTGGTGGGAGCCGGAGAT-GGGCCTCAGCTTCTCCTGCTTGAGC
                  G  G  G  R  F  W  G  F  S  S  G  L  A  V  V  S  G  G  S  R  R   W  A  S  A  S  P  A  *
```

frame shift premature stop

**C**

```
                  S  W  R  V  Y  S  A  G              S  G  G  G  S  R  A  V          C  Q  S  R  G  R  C
Elephant Krt77   AGCTGGCGGGTTTACAGTGCAGGC------------TCTGGTGGTGGGAGCAGGGCTGTG--------TGTCAGTCCAGAGGGAGGTGTG
Manatee Krt77 GB AGCAGGCGGGTTTACAGTGCAGGCTGTTCTGCAGGCTCTGGTGGTGGGAGCCGGGCTGTGGGATCTGGTGTCAGGCCCGAGGGAGGTGTG
Manatee Krt77PCR AGCAGGCGGGTTTACAGTGCAGGCTGTTSTGCAGGCTCTGGTGGTGGGAGCCGGGCTGTGGGATCTGGTGTCAGGCCCGAGGGAGGTGTG
                  S  R  R  V  Y  S  A  G  C  S  A  G  S  G  G  G  S  R  A  V  G  S  G  V  R  P  E  G  G  V
```

frame shift

```
                 G  G  S  G  Y  A  S  Y  G  K  G  F  G  S  R  S  L  Y  N  L  G  G  S  K  S  I  F  I  S  L
Elephant Krt77   GCGGTAGTGGGTATGCGAGCTATGGGAAGGGGTTTGGCTCTAGGAGCCTCTACAATCTGGGTGGCAGTAAAAGCATCTTCATTAGTCTGG
Manatee Krt77 GB GTGGTGGTGGATATAGGGGCTATGGGAGGGGGGTTGGCTCTAGGAGCCTCTACAATCTGGGTGGCAGTAAAAGCATCTCCATTAGCCTAG
Manatee Krt77PCR GTGGTGGTGGATATAGGGGCTATGGGAGGGGGGTTGGCTCTAGGAGCCTCTACAATCTGGGTGGCAGTAAAAGCATCTCCATTAGCCTAG
                  V  V  V  D  I  G  A  M  G  G  G  L  A  L  G  A  S  T  I  W  V  A  V  K  A  S  P  L  A  *
```

premature stop

**Suppl. Fig. S3. Confirmation of inactivating mutations in suprabasal epidermal keratin genes of the manatee.** The investigation of genome sequences available in GenBank (Suppl. Table S6) suggested the presence of mutations that lead to frame shifts and premature stop codons in the genes *Krt1* (**A**), *Krt2* (**B**), and *Krt77* (**C**) of the manatee. The regions around some of these mutations were amplified by PCR from genomic DNA of the manatee and sequenced. The resulting sequences ("PCR") were aligned with the genomic sequence from GenBank ("GB") and with the sequence of the orthologous gene of a related terrestrial species (elephant). Blue fonts indicate nucleotide sequence identity. Red fonts indicate mutations that lead to gene inactivation. Amino acid sequences are shown above or below coding nucleotide sequences.

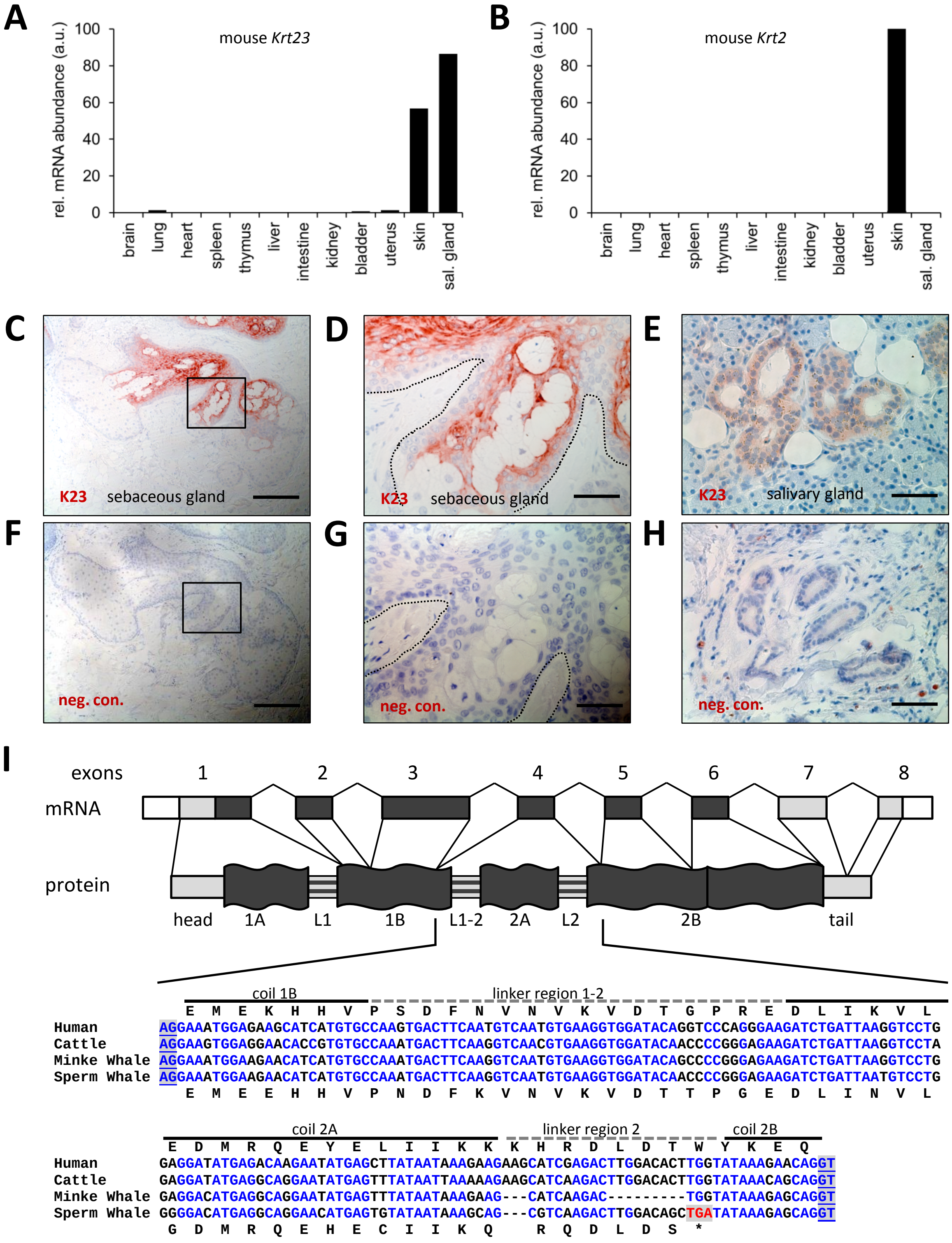


**Suppl. Fig. S4. Analysis of K23 gene expression and sequence conservation.** RT-PCR analysis of *Krt23* (**A**) and, for comparison, *Krt2* (**B**) expression in mouse organs. The mRNA abundance levels were normalized to the level of the house-keeping gene *B2m* and arbitrary units were calculated by assigning a value of 100 to the maximum expression level. (**C**-**H**) Immunohistochemical investigation of K23 in sebaceous glands (**C**, **D**, **F**, **G**) and salivary glands (**E**, **H**). Guinea pig anti-human K23 (**C**-**E**) or, for negative controls, an immunoglobulin preparation of non-immunized guinea pigs (**F**-**H**) were used as primary antibodies. Panels **D** and **G** show details of **C** and **F** (boxes), respectively, at higher magnification. The dermo-epidermal junction is indicated by dotted lines in **D** and **G**. Scale bars: 200 µm (**C**, **F**), 50 µm (**D**, **E** , **G** , **H**). (**I**) Scheme of *Krt23* exons, K23 protein structure (highlighting the α-helical domains) and nucleotide sequence alignment of *Krt23* exon 4 of human, cattle, minke whale and sperm whale. Sperm whale *Krt23* contains a premature stop codon (red) and minke whale *Krt23* shows nucleotide deletions in the region encoding linker 2.

# A

```
Minke whale K6   MTTTNRQFFSSSSIKGSSGLGGGLSLTSRQQSGSLGAGSCRLGSAGSLGNALGDGSYSSCYSFGSGGGYGSGGYGSGGYGSGGYGSGVYG
Gray whale K6    MTTTNRQFFSSNSIKGSSGLGGGLTLTSRQQSGSLGAGSCRLGSAGSLGNALGECSYSSCYSFGSGGGYGSGGYGSGGYGSGGYGSGGYG
                 *********** ************ ***************************** ******************************** **

Minke whale K6   SGSFGTGGYGSGLGGGDGLLVGSEKATMQNLNDRLASYLDKVHALEEANTELEVKIHNWYQKQTPGPATDYSHYFKTIEDLRNQILKATT
Gray whale K6    SGSFGTGGYGSGLGGGDGLLVGSEKATMQNLNDRLASYLDKVHALEEANTELEVKIHNWYQKQIPGPAPDYSHYFKTIEDLRNQILKATT
                 *************************************************************** **** *********************

Minke whale K6   DNANILLQIDNARLAADDFYTKFETEHALCMSVEADTNGLRRVLDELTLARADLEMQIENYKEELAYLRKNHEEEMNALRGQVGGEISVE
Gray whale K6    DNASILLQIDNARLAADDFWTKFETEHALCTSVEADINGLRRVLDELTLARADLEMQIENYKEELAYLQNNHEEEMNSLRGQVGGDINVE
                 *** *************** ********** ***** *******************************  ******* ********* **

Minke whale K6   MDAAPGVDLSRILNEMREQYEKMAEKNRKDAEDWFLSKTEELNREVTTNCELAQSSKCEVSELRRTMQALEIELQSQLSMKASLENSLAE
Gray whale K6    MDAAPGVDLSRILNEMREQYEKMAEKNRKDAEDWFLRKTEELNREVTTNCELAQSSKCEVSELRRTMQALEIELQSQLSMKASLENSLAE
                 ************************************ *****************************************************

Minke whale K6   TENRYCMQLGQIQGLICNVEEQLAQLRCETEQQNQEYKILLAVKTRLEQEIATYRRLLEGEDSQLIHHKLRESATTRQVRTIVEEVKDGR
Gray whale K6    TENRYCLQLGQIQGLICNVEEQLAQLRCETEQQNQEYKILLDVKTRLEQEIATYRCLLEGEDSQLTQQKLKESSTTRQVRTIVEEVKDGR
                 ***************************************** ************* *********   ** ** ****************

Minke whale K6   VISSHEQVHQTTH
Gray whale K6    VISSHEQVHQTTH
                 *************
```

# B

```
Minke whale K17  MTSKSTVKSQSSSRRVFSAGSARVPGVSRSGFSSVPVCRSRGSGGLAGVGGGAGFGSRSLYGVGGSKRISLGGGSCALGGGYGGSAGGGY
Gray whale K17   MTSKSTVKGQSCSRRVFSAGSARVPGVSCSGFSSVPMSRSRGSGGLTGVGGGAGFGSRSLYGVGGSKRISLEVGCVYRGGGYGGSAGGGY
                 ******** ** **************** *******  ******** *************************   *   ***********

Minke whale K17  SVGGGAGSGFGFGSGAGGGFGLGGGAGFGIGYWGPGMPVCPPGGIQEVTINQSLLTPLNLQIDPTIQRIKTEEREQIKTLNNRFASFIDK
Gray whale K17   GVGGGGGSGFGFGSGASGGFGLGGGAGFGIGYWGPGIPVCPPGGIQEVTINQSLLTPLNLQIDPNIQQIKTEEREQIKTLNNRFASFIDK
                 **** ********** ******************* *************************** ** **********************

Minke whale K17  VRFLEQQNKVLETKWTLLQEQGTKTVRHNLEPLFEQYINNLRRQLDSLVTERSRLDSEFRGMQDTVEDFKKKYEDEINRRMSAEHEFVNL
Gray whale K17   VRFLEQQNKVLETKWTLLQEQGTKTVRHDLEPLFEQYIYNLRRQLDSLVMERSCLDSEFRGMQDTVEDFKKKYEDEINRRMSAENEFVNL
                 ************************************** ********** *** ****************************** *****

Minke whale K17  KKDVDTAYMNKVELETKADALIDEINFLRALYEAELAQMQTHISDTSVVLSMDNNRTLDLDSIIAEVKAQYEVIACRSREEAECWYKCKY
Gray whale K17   KKDVDTAYMNKVEVETKADALIDEINFLRALYEAELAQMQTHISDTSVVLSMDNNRTLDLDSIIAEVKAQYEVIACRSREEAECWYKCKY
                 ************* ****************************************************************************

Minke whale K17  EELQLTAGRHGDDLRNTKQEITEINRMIQRLRSEIDHVKKQCANLQSAIADAEQRGELALRDAKNKLAELENALQKAKQDMAQLVKDYQE
Gray whale K17   EELQLTAGKHGDDLRNTKQEITEINRMIQRLRSEIDHVKKQCANLQSAIADAEQRGELALRDAKNKLAELENALQKAKQDMAQLVKDYQE
                 ******** *********************************************************************************

Minke whale K17  LMNVKLSLDVEIATYRKLLEGEECRLNGEGVGQVNISVVQSTVSSGYGGAGGVSGGLGLGGGSGYSYSSGHGIGGGFSSGGGFSSGSGRA
Gray whale K17   LMNVKLSLDVEIATYRKLLEGEECRLTGEGVGQVNISVVQSTVSSGYGGAGGVSGGLGLGGGSGYSYSSGHGIGGGFSSG------SGRA
                 ************************** *****************************************************      ****

Minke whale K17  IGGGLSSSGGSSSAIKYTTTSSSSRKGYRH
Gray whale K17   IGGGLSSSGGSCSAIKYTTTSSSSRKGYRH
                 ******************************
```

# C

```
Minke whale K23  MNSSHSFSQTPSGSLYGTGSSWGQPGRFPRAPSVHGGAGGVCISLSFSSPRCPPPGGSWGSGRGSSLLGGNGKAMMQNLNDRLASYLDKV
Gray whale K23   MNSSHSFSQTPSGSLYGTGSSWGQPGRFPRAPSVHGGAGGVCISLSFSSPRCPPPGGSWGSGRGSSLLGGNGKAMMQNLNDRLASYLDKV
                 ******************************************************************************************

Minke whale K23  RALEEANVKLESCILKWHQQRDPGSEQDYSQYEEKVSCLQEQIVDCKMTNAQITLLIDNARIAVDDFSHKYENEHSVKKNLEIEVESLRN
Gray whale K23   RALEEANVKLESCILKWHQQRDPGSEQDYSQYEEKVSCLQEQIVDGKMTNAQITLLIDNARIAVDDFSHKYENEHSVKKNLEIEVESLRN
                 ********************************************* ********************************************

Minke whale K23  NLDDLTIVTTDLEQEVEGMREELILMKKRHEQEMEEHHVPNDFKVNVKVDTAPGEDLIKVLEDMRQEYEFIIKKHQDWYKEQLAAMTQEV
Gray whale K23   NLDDLTIVTTDLEQEVEGMREELILMKKRHEQEMEEHHVPNDFKVNVKVDTAPGEDLIKVLEDKRQEYEFIIKKHQDWYKQQLAAMTQEV
                 *************************************************************** **************************

Minke whale K23  ARPTAVQSSQSDIRELKRTFQALEIDLQAQCNKKSALENTLSETQSRYSCQLQDIQRIISHYEEELMQLRHDLECLSNEYKVLLGIKTHL
Gray whale K23   ARPTAVQSSQSDIRELKLTFQALEIDLQAQCNTKSALENTLSETQSRYSCQLQDIQRIISHYEEELMQLRHDLECLSNEYKVLLGIKTHL
                 ***************** ************** *********************************************************

Minke whale K23  EKEISTYRQLLDGENGGTMEESKSSVKAPKIKVIIQESINGRIILSQVNEI
Gray whale K23   EKEIATYRQLLDGENGGTMEESKSSVKAPKIKVIIQESINGRIILSQVNEI
                 **** **********************************************
```

## D

```
Minke whale K80  MACRCCIVGFGNLNSCEVTQASSPWPGTSGWSNYRAPEPGFSSGSLTGCLTASTIPKVTVNPSLLVPLDLKVDPAIQQQKSQKKEEMKVL
Gray whale K80   MACRCCIVGFGNLNSCEVTQVSSPWPGTSGWSNYRAPEPGFSSGSLTGCLTASTIPKVTVNPSLLVPLDLKVDPAIQQQESQKKEEMKVL
                 ******************** ********************************************************** **********

Minke whale K80  NDKFASLIGKVQALEQRNQLLETHWHFLRSRDLAACDLGHLYEEYQDRLQEELRKVNQEGAQLEAKLLQELAMVQKFQIRYEDEVSKRTD
Gray whale K80   NDKFASLIGKVQALEQRNQLLETHWRFLRSRDSAACDLGHLYEEYQGRLQEELRKVNQEGGQLEAKLLQELAMVQKFQIRYEDEVSKRTD
                 ************************* ****** ************* ************* *****************************

Minke whale K80  MEFTAVQLKKDLNAERLRQTQLETKLKGLKSFAVMMKSIYEQELKDLAAQVKDVSVTVGMGSRCHTDLSGIVEKVMAQYGAVVARSLEEA
Gray whale K80   MEFTAAQLKKDLNAERLRQTQLETKLKGLKSFAVMMKSIYEQELKDLAAQVKDVSVTVGMDSRCHTDLSGIVEKVMAQYGAVVARSLEEA
                 ***** ****************************************************** *****************************

Minke whale K80  KAYSRSQVRSSAACSAESGNRLQRSRSKINDLSARIQKLRSQILSIKSHCLKLEENIKAAENQGELAFQDAKAKLAQLEAALQQAKKDMA
Gray whale K80   KAYSRSQLEERAACSAESGNSLQRSRSKINDLNARIQKLRSQILSIKSHCLKLEENIKAAENQGELAFQDAKAKLAQLEAALQQAKKDMA
                 *******    ********* *********** *********************************************************

Minke whale K80  RQLCDYQELMNTKLVLDIEIATYGKLVEGEESRMDLPAATVISCMQSRSRTAASKYGLSRSPSRKKKNREHPVIKITETSEEFLSQESEV
Gray whale K80   RQLRDYQELMNTKLVLDIEIATYGKLVEGEESRMDWPSATVISCMQSRSRTAASKYGLSRSPSRKKKNREHPVIKITETSEEFLSQESEV
                 *** ******************************* * ****************************************************

Minke whale K80  SQ
Gray whale K80   SQ
                 **
```

## E

```
                          coil 2A                       linker region 2    coil 2B
                       E  D  M  R  Q  E  Y  E  L  I  I  K  K  K  H  R  D  L  D  T  W  Y  K  E  Q
Human KRT23            GAGGATATGAGACAAGAATATGAGCTTATAATAAAGAAGAAGCATCGAGACTTGGACACTTGGTATAAAGAACAGGT
Cattle Krt23           GAGGATATGAGGCAGGAATATGAGTTTATAATTAAAAAGAAGCATCAAGACTTGGACACTTGGTATAAACAGCAGGT
Hippopotamus Krt23     GAGGACATGAGGCAAGAATACGAGTTTATAAGAAAGAAGAAGCATCAAGACTTGGATGCCTGGTATAAAGAGCAGGT
Sperm whale ψKrt23     GGGGACATGAGGCAGGAACATGAGTGTATAATAAAGCAG---CGTCAAGACTTGGACAGCTGATATAAAGAGCAGGT
Minke whale Krt23      GAGGACATGAGGCAGGAATATGAGTTTATAATAAAGAAG---CATCAAGAC---------TGGTATAAAGAGCAGGT
Gray whale Krt23       GAGGACAAGAGGCAGGAATATGAGTTTATAATAAAGAAG---CATCAAGAC---------TGGTATAAACAGCAGGT
Humpback whale Krt23   GAGGACAAGAGGCAGGAATATGAGTTTATAATAAAGAAG---CATCAAGAC---------TGGTATAAAGAGCAGGT
Blue whale Krt23       GAGGACAAGAGGCAGGAATATGAGTTTATAATAAAGAAG---CATCAAGAC---------TGGTATAAAGAGCAGGT
```

**Suppl. Fig. S5. Investigation of epidermal keratins in baleen whales.** (**A-D**) Amino acid sequences of K6 (**A**), K17 (**B**), K23 (**C**) and K80 (**D**) of the gray whale (*Eschrichtius robustus*) were predicted from genomic DNA sequences available as unassembled whole-genome shotgun sequence scaffolds (Moskalev et al. 2017) and aligned with sequences of orthogs of the minke whale (*Balaenoptera acutorostrata scammoni*). Positions of identical amino acids are indicated by asterisks below the sequences. Accession numbers of gray whale keratin genes: *Krt6*: NTJE010017954, nucleotide range of coding sequence (CDS) 7781- 14297; *Krt17*: NTJE010143275, CDS nucl. 10779-15854 (reverse complementary sequence); *Krt23*: NIPP01005962, CDS nucl. 95739-108635 (reverse complementary sequence); *Krt80*: NIPP01005781, CDS nucl. 76253-105489. Orthologs of *Krt1*, *Krt2*, *Krt77*, *Krt9*, and *Krt10* were not found by tBLASTn searches in the whole-genome shotgun sequence of the gray whale. (**E**) The nucleotide sequences encoding the linker region 2 of K23 of baleen whales were identified by tBLASTn searches of raw sequence reads (Senckenberg Gesellschaft fuer Naturforschung) (Árnason et al., 2018) that were available in the GenBank Sequence Read Archive (SRA), Project accession number: PRJNA389516. Sequence read accession numbers: Humpback whale, accession number SRR5665639.69002869.2, experiment SRX2901261); blue whale, acc. nr. SRR5665644.341793213.2, experiment SRX2901266; gray whale, acc. nr. SRR5665642.71591331.1, experiment SRX2901263). These sequences are aligned to nucleotide sequences of human, cattle, hippopotamus, minke whale *Krt23* as well as the sperm whale *Krt23* pseudogene (Suppl. Table S2). *Krt23* of *Hippopotamus amphibius* was identified by tBLASTn search on HIP001 scaffold17053 (WGS, GenBank acc. nr. NKPW01017053) (Arnason et al., 2018). Nucleotides conserved in all species are indicated by blue letters. Gray shading indicates an in-frame stop codon. Intronic sequences are underlined. The translation of th human *KRT23* sequence is shown above the alignment.

```
                 G  G  G  S  S  S  G  G  G  Y  G  G  G  S  S  S  G  G  H  K  S  S  S  S  G  S  V  G  E  S  S  S  K
    K10 cDNA    CGGCGGCGGCAGCAGCTCCGGCGGCGGATACGGCGGCGGCAGCTCCAGCGGAGGCCACAAGTCCTCCTCTTCCGGGTCCGTGGGCGAGTCTTCATCTAAG
  KRT10 gene    CGGCGGCGGCAGCAGCTCCGGCGGCGGATACGGCGGCGGCAGCTCCAGCGGAGGCCACAAGTCCTCCTCTTCCGGGTCCGTGGGCGAGTCTTCATCTAAG
   K10x1 EST    CGGCGGCGGCAGCAGCTCCGGCGGCGGATACGGTGGCGGCAGCTCCAGCGGAGGCCACAAGTCCTCCTCTTCCGGGTCCGTGGGCGAGTCTTCATCTAAG
                 G  G  G  S  S  S  G  G  G  Y  G  G  G  S  S  S  G  G  H  K  S  S  S  S  G  S  V  G  E  S  S  S  K

                 G  P  R
    K10 cDNA    GGACCAAG-------------------------------------------------------------------------------------------
  Krt10 gene    GGACCAAGGTCAGCAGAAACTAGCTGGGGTAATCAGAATTAGTTTTAACTTCCTGTGATGGTTTTTTTGCGCTTTAAGCTCTAGAGTTGTTTTAAAAAAT
   K10x1 EST    GGACCAAGGTCAGCAGAAACTAGCTGGG-----------------------------------------------------------------------
                 G  P  R  S  A  E  T  S  W

    K10 cDNA    ---------------------------------------------------------------------------------------------------
  KRT10 gene    TAAAAATCTTAGAGACGGTTCCGTTTGCATTTGTTCACAAACTACTCTTAACACCAGCCGTGAAAAATGGCATGATCAAAATGTCATACCTTAAGCATTT
   K10x1 EST    ---------------------------------------------------------------------------------------------------

    K10 cDNA    ---------------------------------------------------------------------------------------------------
  KRT10 gene    TTTTGGGCTTAACAATGTAAAGTTGAAATTTCCTTCTTTTTACAATATTTGCTTGTTAATTACTAAGGATCCCTACAGACTGTTTAAAATTTTTTTTCCA
   K10x1 EST    ---------------------------------------------------------------------------------------------------

                              Y  *
    K10 cDNA    ------------ATACTAACAAAACCAGAGTAATCAAGACAATTATTGAAGAGGTGGCGCCCGACGGTAGAGTTCTTTCATCTATGGTTGAATCAGAAAC
  KRT10 gene    TCATTCACACAGATACTAACAAAACCAGAGTAATCAAGACAATTATTGAAGAGGTGGCGCCCGACGGTAGAGTTCTTTCATCTATGGTTGAATCAGAAAC
   K10x1 EST    ------------ATACTAACAAAACCAGAGTAATCAAGACAATCATTGAAGAGGTGGCGCCCGACGGTAGAGTTCTTTCATCTATGGTTGAATCAGAAAC
                            D  T  N  K  T  R  V  I  K  T  I  I  E  E  V  A  P  D  G  R  V  L  S  S  M  V  E  S  E  T

    K10 cDNA    CAAGAAACACTACTATTAAACTGCATCAAGAGGAGAGAGTCTCCCTTCACACAGACCATTAATTTACAGATGCATGGAAAACAAAGTCTCCAAGAAAACA
  KRT10 gene    CAAGAAACACTACTATTAAACTGCATCAAGAGGAAAGAGTCTCCCTTCACACAGACCATTA-TTTACAGATGCATGGAAAACAAAGTCTCCAAGAAAACA
   K10x1 EST    CAAGAAACACTACTATTAAACTGCATCAAGAGGAAAGAGTCTCCCTTCACACAGACCATTA-TTTACAGATGCATGGAAAACAAAGTCTCCAAGAAAACA
                  K  K  H  Y  Y  *
```

**Suppl. Fig. S6. Nucleotide sequence alignment of the 3‘-ends of human *KRT10* and two mRNA variants.** Coding sequences (yellow shading) of two K10 mRNA variants were aligned to exons 7 and 8 of the human *KRT10* gene. The K10 variant (Genbank accession number M19156) was cloned from human epidermal keratinocytes (Darmon et al., 1987), and the sequence of the K10x1 variant corresponds to an expressed sequence tag (EST) (Accession number CU446017.1) that was cloned from a human epidermis sample enriched in granular keratinocytes (Toulza et al., 2007). Splice donor and acceptor signals are highlighted by blue shading. Stop codons are shown in red. Amino acid sequences encoded by the transcripts are indicated. *, end of protein.

```
human_K10           MSVRYSSSKHYSSSRSGGGGGGGGCGGGGGVSSLRISSSKGS-LGGGFSSGGF------S
seal_K10            MSVRYSSSKQYSSSRSGGGGGGGGG------SSHKISSSKSS-IGGGFSSGGF------S
opossum_K10L1       MSQRYGSSRHYSSSRSGGGAGGG--------SSIRISSTRGSGYGAGSIGGGF-------
opossum_K10L2       MSQRYGSSRHYSSSRSGGGAGGG--------SSIRISSTRGSGYGAGSIGGGF-------
opossum_K10         MSQRYGSSKNFSSSRSGG--GGS--------SSIRISSSRGSSYGGGSVGGRFISTGSSS
opossum_K10L1x1     MSQRYGSSRHYSSSRSGGGAGGG--------SSIRISSTRGSGYGAGSIGGGF-------
opossum_K10L2x1     MSQRYGSSRHYSSSRSGGGAGGG--------SSIRISSTRGSGYGAGSIGGGF-------
opossum_K10x1       MSQRYGSSKNFSSSRSGG--GGS--------SSIRISSSRGSSYGGGSVGGRFISTGSSS
seal_K10x1          MSVRYSSSKQYSSSRSGGGGGGGGG------SSHKISSSKSS-IGGGFSSGGF------S
human_K10x1         MSVRYSSSKHYSSSRSGGGGGGGGCGGGGGVSSLRISSSKGS-LGGGFSSGGF------S
alligator_K10       MSFFGCGSRQISSCHTGG-------------ASMKIPCWGVYGLGGGSGLGMY-------
anole_K10           MSYFGRGSRQVSSSHSGG-------------TSAKLSGCGAYGLGGGSSAGSL-------
snake_K10           MSYFGCGSRQMSSSRSSG-------------APLKLSSWGIYGLGGGSSTGSF-------
                    **    .*.: **..:.*             :. .:.       *.*   *

human_K10           GGSFSRGSSGGGCFGGSSGGYGGL-GGFGGGSFRG-SYGSSSFG-GSYGGSFGGGSFGGG
seal_K10            GGSFSRGSSGGGCFGGSSGGYGGLGGGFGGGNFGG-GCGSSSFGGGYGGGSFGGGSFGGG
opossum_K10L1       ---SSAGGFGSRSVGGFVGGFSS--SSYGGGSFGG-SYGGVSCGGGSFGGLFD-------
opossum_K10L2       ---SSAGGFGSRSVGGFVGGFSS--SSYGGGSFGG-SYGGVSCGGGSFGGLFD-------
opossum_K10         YGSCSRGGYGGGSLGGFGGGFGG--GSCGGGSFGG-GYGGGSCGGGSFGGGYGGGSFGGG
opossum_K10L1x1     ---SSAGGFGSRSVGGFVGGFSS--SSYGGGSFGG-SYGGVSCGGGSFGGLFD-------
opossum_K10L2x1     ---SSAGGFGSRSVGGFVGGFSS--SSYGGGSFGG-SYGGVSCGGGSFGGLFD-------
opossum_K10x1       YGSCSRGGYGGGSLGGFGGGFGG--GSCGGGSFGG-GYGGGSCGGGSFGGGYGGGSFGGG
seal_K10x1          GGSFSRGSSGGGCFGGSSGGYGGLGGGFGGGNFGG-GCGSSSFGGGYGGGSFGGGSFGGG
human_K10x1         GGSFSRGSSGGGCFGGSSGGYGGL-GGFGGGSFRG-SYGSSSFG-GSYGGIFGGGSFGGG
alligator_K10       ---ETGGNWCGGL------GWGGI-GGGTAGSFHNFSYGAVGNN----------------
anole_K10           ---CSRTTWSTGS------GWGGI-GGGTGGSFRIYSSSGSIC-----------------
snake_K10           ---SSGAVWGSGS------GWGKIGGGGTDGCFH--IYNSSPA-----------------
                        :              *:.   ..   * *     ..

human_K10           SFGGGSFGGGGFGGGGFGGGFGGGFGGDGGLLSGNEKVTMQNLNDRLASYLDKVRALEES
seal_K10            SFGGGSFGGGSFGGY--GGGF----GGDGGLLSGNEKVTMQNLNDRLASYLDKVRALEES
opossum_K10L1       -------------------------GSDGGLLSGNEKVTMQNLNDRLASYLDKVKALEDS
opossum_K10L2       -------------------------GSDGGLLSGNEKVTMQNLNDRLASYLDKVKALEDS
opossum_K10         SFGGGSFGGGSFGGGLLGGSFG---GGDGGLLSGNEKVTMQNLNDRLASYLDKVKALEDS
opossum_K10L1x1     -------------------------GSDGGLLSGNEKVTMQNLNDRLASYLDKVKALEDS
opossum_K10L2x1     -------------------------GSDGGLLSGNEKVTMQNLNDRLASYLDKVKALEDS
opossum_K10x1       SFGGGSFGGGSFGGGLLGGSFG---GGDGGLLSGNEKVTMQNLNDRLASYLDKVKALEDS
seal_K10x1          SFGGGSFGGGSFGGY--GGGF----GGDGGLLSGNEKVTMQNLNDRLASYLDKVRALEES
human_K10x1         SFGGGSFGGGGFGGGGFGGGFGGGFGGDGGLLSGNEKVTMQNLNDRLASYLDKVRALEES
alligator_K10       -------------------------SSDGRLLFGGEKETMQNLNDRLASYLGKVQALEEA
anole_K10           --------------------------IDGGLLFSGEKETMQNLNDRLASYLAKVHALEKA
snake_K10           -------------------------GGDVGLLAGGEKETMQNLNDRLASYLTKVHALEEA
                                               *  ** ..** ************* **.***.:

human_K10           NYELEGKIKEWYEKHGNSHQGEPRDYSKYYKTIDDLKNQILNLTTDNANILLQIDNARLA
seal_K10            NYELEGKIKEWYEKYGNSSQRERRDYSKYYQIIEDLKNQILRLTTDNANILLQIDNARLA
opossum_K10L1       NSQLEQKIKEWYEKYGNSRQREPRDYSHYYDVIKDLKDQILTLTTDNANVLLQIDNARLA
opossum_K10L2       NSQLEQKIKEWYEKYGNSRQREPRDYSHYYDVIKDLKDQILTLTTDNANVLLQIDNARLA
opossum_K10         NYELEQKIKEWYEKNGNSRQREPRDYSNYYDTIADLKNQILNLTNDNANVLLQIDNARLA
opossum_K10L1x1     NSQLEQKIKEWYEKYGNSRQREPRDYSHYYDVIKDLKDQILTLTTDNANVLLQIDNARLA
opossum_K10L2x1     NSQLEQKIKEWYEKYGNSRQREPRDYSHYYDVIKDLKDQILTLTTDNANVLLQIDNARLA
opossum_K10x1       NYELEQKIKEWYEKNGNSRQREPRDYSNYYDTIADLKNQILNLTNDNANVLLQIDNARLA
seal_K10x1          NYELEGKIKEWYEKYGNSSQRERRDYSKYYQIIEDLKNQILRLTTDNANILLQIDNARLA
human_K10x1         NYELEGKIKEWYEKHGNSHQGEPRDYSKYYKTIDDLKNQILNLTTDNANILLQIDNARLA
alligator_K10       NAELEHKIKDWYETLGSYTEGGSRDYSKYYTTIEDLRNQIIAATVENASIILQTDSARLA
anole_K10           NSELELKIKNWYEKNAFQADGGFRDYSKYYASIDELRNKIVSASVNNSNIILQIDNARLA
snake_K10           NSALEQKIRNWYEKSATHINEGLYDSSRYNSSIDDLRNKIISASVRNGSIILQIDNARLA
                    *  ** **.:***. .   :    * *.*   * :*.::*:  :  *..::** *.****

human_K10           ADDFRLKYENEVALRQSVEADINGLRRVLDELTLTKADLEMQIESLTEELAYLKKNHEEE
seal_K10            ADDFRLKYENEVALRQSVEADINGLRRVLDELTLSKTDLEMQIESLTEELAYLKKNHEEE
opossum_K10L1       ADDFRLKYENEVALHQSVEADINGLRRVLDDLTLAKTDLEVQLESLNEELAFLKKNHEEE
opossum_K10L2       ADDFRLKYENEVALHQSVEADINGLRRVLDDLTLAKTDLEVQLESLNEELAFLKKNHEEE
opossum_K10         ADDFRLKYENEVALRQSVEADINGLRRVLDDLTLAKTDLEMQLEGLSEEMAFLKKNHEEE
opossum_K10L1x1     ADDFRLKYENEVALHQSVEADINGLRRVLDDLTLAKTDLEVQLESLNEELAFLKKNHEEE
opossum_K10L2x1     ADDFRLKYENEVALHQSVEADINGLRRVLDDLTLAKTDLEVQLESLNEELAFLKKNHEEE
opossum_K10x1       ADDFRLKYENEVALRQSVEADINGLRRVLDDLTLAKTDLEMQLEGLSEEMAFLKKNHEEE
seal_K10x1          ADDFRLKYENEVALRQSVEADINGLRRVLDELTLSKTDLEMQIESLTEELAYLKKNHEEE
human_K10x1         ADDFRLKYENEVALRQSVEADINGLRRVLDELTLTKADLEMQIESLTEELAYLKKNHEEE
alligator_K10       VDDFKVKYENELYLWQNVEADIRDLRKVLDEQTLAKSNLEIQIESLADEVAYLKKNHEAE
anole_K10           ADDFRMKYENEVFFRQSIEADINGLHKVFDEVILSKSNLELQIESLIEELAFLKKNHDEE
snake_K10           ADDFRMKYENERFLHQNVEADINGLKKVQDELSLSKSDLEIQIESLMEELAFLKKNHEEE
                    .***.:*****  :.*.:****..*..* *:  *:*.:**:*:*.* :*:*:*****: *

human_K10           MKDLRNVSTGDVNVEMNAAPGVDLTQLLNNMRSQYEQLAEQNRKDAEAWFNEKSKELTTE
seal_K10            MKDLQNVSTGDVNVEMNAAPGIDLTELLNNMRNQYEQLAEQNRKDAEAWFNEKSKELTTE
opossum_K10L1       MKDLQNVSTGDVNVEMNAAPGVDLTENLNLLRSQYEQLAEQNRREAEAWFNEKSKELTTE
opossum_K10L2       MKDLQNVSTGDVNVEMNAAPGVDLTENLNLLRSQYEQLAEQNRKEAEAWFNEKSKELTTE
opossum_K10         MKDLQNVTTGDVNVEMNAAPGVDLTQYLNRMRNEYEQLAEQNRRDAEAWFNEKSKELTTE
opossum_K10L1x1     MKDLQNVSTGDVNVEMNAAPGVDLTENLNLLRSQYEQLAEQNRREAEAWFNEKSKELTTE
opossum_K10L2x1     MKDLQNVSTGDVNVEMNAAPGVDLTENLNLLRSQYEQLAEQNRKEAEAWFNEKSKELTTE
opossum_K10x1       MKDLQNVTTGDVNVEMNAAPGVDLTQYLNRMRNEYEQLAEQNRRDAEAWFNEKSKELTTE
seal_K10x1          MKDLQNVSTGDVNVEMNAAPGIDLTELLNNMRNQYEQLAEQNRKDAEAWFNEKSKELTTE
human_K10x1         MKDLRNVSTGDVNVEMNAAPGVDLTQLLNNMRSQYEQLAEQNRKDAEAWFNEKSKELTTE
alligator_K10       MNDLRSSASPDVTVEINTAPGIDLTTRLNKMRTEYEALAEQNRKDVETWFNEKSKELNVQ
anole_K10           IRGLRGPTSGNVSVEMNAVPGPDLTILLNKMREEYEALAEQNRQDAETWFVKKSNELNTT
snake_K10           MKSWQGTVSGDVNVEINTAPGVDLTTVLNKMRAEYEALAEQNRRDAETWFSEKSKELNMT
                    :.. ..  : :*.**:*:.** ***  ** :* :** ******.:.*:** :**:**.
```

Suppl. Fig. S7, page1

```
human_K10           IDNNIEQISSYKSEITELRRNVQALEIELQSQLALKQSLEASLAETEGRYCVQLSQIQAQ
seal_K10            INSNIEQMSSYKSEITELRRTVQALEIELQSQLALKQSLESSLAETEGRYCVQLSQIQGQ
opossum_K10L1       INSNVEQVSSQKSEITELRRNVQALEIELQSQLALKQSLEASLAETEGRYCSQLSEIQAQ
opossum_K10L2       INSNVEQVSSQKSEITELRRNVQALEIELQSQLALKQSLEASLAETEGRYCSQLSEIQAQ
opossum_K10         IDSNVEQMSSHKSEITELRRNVQALEIELQSQLALKQSLEASLAETEGRYCSQLSQIQVQ
opossum_K10L1x1     INSNVEQVSSQKSEITELRRNVQALEIELQSQLALKQSLEASLAETEGRYCSQLSEIQAQ
opossum_K10L2x1     INSNVEQVSSQKSEITELRRNVQALEIELQSQLALKQSLEASLAETEGRYCSQLSEIQAQ
opossum_K10x1       IDSNVEQMSSHKSEITELRRNVQALEIELQSQLALKQSLEASLAETEGRYCSQLSQIQVQ
seal_K10x1          INSNIEQMSSYKSEITELRRTVQALEIELQSQLALKQSLESSLAETEGRYCVQLSQIQGQ
human_K10x1         IDNNIEQISSYKSEITELRRNVQALEIELQSQLALKQSLEASLAETEGRYCVQLSQIQAQ
alligator_K10       INFSAEETNTNKSQITELKHTFQGLEIEMKSQLALKQSLEATLAETEARYCAQLSQIQVV
anole_K10           ISMSAKETSTNKKEIAELRRTLQGLEIDLKTQLALRQSLETTLAETEDGYCSQLLQMQGL
snake_K10           INVSAKETITNKKQIADLRHTLQGLEIDLKTQLSLRQSLESTLAETEGQYCAQLLQIQEM
                    *. . ::  : *.:*::*....*.***::::**:*.****::*****  ** ** ::*

human_K10           ISALEEQLQQIRAETECQNTEYQQLLDIKIRLENEIQTYRSLLEGEGSSGGGGRGGGSFG
seal_K10            ISSLEEQLQQIRAETECQNAEYQQLLDIKIRLENEIQTYRSLLEGEGSSGG---------
opossum_K10L1       ITALEEQLQQVRAETEYQNSEYQLLLDIKIRLENEIQTYRSLLEEGGSANS---------
opossum_K10L2       ITALEEQLQQVRAETEYQNSEYQLLLDIKIRLENEIQTYRSLLEEGGSANS---------
opossum_K10         ITALEEQLQQIRAETECQNSEYQMLLDIKIRLENEIQTYRSLLEEGGSGGS---------
opossum_K10L1x1     ITALEEQLQQVRAETEYQNSEYQLLLDIKIRLENEIQTYRSLLEEGGSANS---------
opossum_K10L2x1     ITALEEQLQQVRAETEYQNSEYQLLLDIKIRLENEIQTYRSLLEEGGSANS---------
opossum_K10x1       ITALEEQLQQIRAETECQNSEYQMLLDIKIRLENEIQTYRSLLEEGGSGGS---------
seal_K10x1          ISSLEEQLQQIRAETECQNAEYQQLLDIKIRLENEIQTYRSLLEGEGS------------
human_K10x1         ISALEEQLQQIRAETECQNTEYQQLLDIKIRLENEIQTYRSLLEGEGSSGGGGRGGGSFG
alligator_K10       ISSAETQIQQIRDDMEWQNAEYEQLLDIKIHLENEIETYRHLIDREESDSG---------
anole_K10           INNTEAQVKQVRDDMESQNADYRQLLDIKIHLENEIETYRHLIDSEKSEFV---------
snake_K10           INKVENQVQQIREDMECQNADYRHLLDIKIRLENEIDTYQRLIDGEASESG---------
                    *.  * *::*:* : * **::*  ******.*****:**. *::   *

human_K10           GGYGGGSSGGGSSGGGHGGGHGGSS-GGGYGGGSSGGGSSGGGYGGGSSSGGHGGSSSGG
seal_K10            --------------------HGGHGGHGGHGGH------GGHGGGQTPGSGGHGGGQTPG
opossum_K10L1       ----------------AAIGHGGSS-GGTYRGI------SGGVQGASSSSGAYGGSS-AG
opossum_K10L2       ----------------AAIGHGGSS-GGTYRGI------SGGVQGASSSSGAYGGSS-AG
opossum_K10         -------YGGGSRGGSSGGGYGGSSGGGGYGGS------SGGGQGGGSSSGGYGGSS-GG
opossum_K10L1x1     ----------------AAIGHGGSS-GGTYRGI------SGGVQGASSSSGAYGGSS-AG
opossum_K10L2x1     ----------------AAIGHGGSS-GGTYRGI------SGGVQGASSSSGAYGGSS-AG
opossum_K10x1       -------YGGGSRGGSSGGGYGGSSGGGGYGGS------SGGGQGGGSSSGGYGGSS-GG
seal_K10x1          -----------------SGGHGGHGGHGGHGGH------GGHGGGQTPGSGGHGGGQTPG
human_K10x1         GGYGGGSSGGGSSGGGHGGGHGGSS-GGGYGGGSSGGGSSGGGYGGGSSSGGHGGSSSGG
alligator_K10       --------------------YDTAGTGSGLRE----------------------------
anole_K10           -----------------------------YRDT----------KTLESGT-------DL-
snake_K10           --------------------------QRSSRNL--------GSQNLESGTG---------

human_K10           ---YGGGSSGGGGGGYGGGSSGGGSSSGGGYGGGSSSGGHKSSSSGSVGESSSKGPR---
seal_K10            GGHGGGQTPGGGGKG-----------SGGGYGGGIANGSYKSSSSGSVGEFSSKAPR---
opossum_K10L1       ---SGGVTSSGSRRG-----------SSVVQGGSSGSGGESTSKSSS-------GTR---
opossum_K10L2       ---SGGVTSSGPRRG-----------SSVVQGGSSGSGGESTSKSSS-------GTR---
opossum_K10         ---SGEFKSSGSRGG-----------SSGRQGGSSGSGGESPSKSSS-------GTR---
opossum_K10L1x1     ---SGGVTSSGSRRG-----------SSVVQGGSSGSGGESTSKSSS-------GTRSGE
opossum_K10L2x1     ---SGGVTSSGPRRG-----------SSVVQGGSSGSGGESTSKSSS-------GTRSGE
opossum_K10x1       ---SGEFKSSGSRGG-----------SSGRQGGSSGSGGESPSKSSS-------GTRSAE
seal_K10x1          GGHGGGQTPGGGGKG-----------SGGGYGGGIANGSYKSSSSGSVGEFSSKAPRSAE
human_K10x1         ---YGGGSSGGGGGGYGGGSSGGGSSSGGGYGGGSSSGGHKSSSSGSVGESSSKGPRSAE
alligator_K10       ---TGSATTSGSQGS--------------VSAADLGSRGSGSAR---------SESRSSS
anole_K10           ----KLRGTEYGTRGL---------GSGRDSISSGTEAIDTN-----------SESKE--
snake_K10           ---SGLRASGSDVRGPGTGTETSSRGSAA------------------------TNVESAT
                              .                      . .. .   ..  .

human_K10           ---Y----------------------------------
seal_K10            ---Y----------------------------------
opossum_K10L1       ---F----------------------------------
opossum_K10L2       ---F----------------------------------
opossum_K10         ---F----------------------------------
opossum_K10L1x1     TN------------------------------------
opossum_K10L2x1     TN------------------------------------
opossum_K10x1       TTWDSNRTPVIKTIIEEVAPDGRVLSSRVESEME-HYY
seal_K10x1          TSWDTNKPRVIKTIIEEVASDGRVLSSVVESETRKH-Y
human_K10x1         TSWDTNKTRVIKTIIEEVAPDGRVLSSMVESETKKHYY
alligator_K10       SSKKPTKTRVIKTIIEELDQHGRVTSSKVQSVEKKPII
anole_K10           KGKETKRTTVIKTIIEELDEHGRVTSSKVQSVEEKTVI
snake_K10           KDKEIKKTRVIKTIVEELDEYGRVTSSKVQSVEE-TQG
```

**Suppl. Fig. S7. Amino acid sequence alignment of K10 protein isoforms of opossum and other mammals and K10 of reptiles.** Amino acid sequences of K10 and K10x1 of the opossum (*Monodelphis domestica*), human (*Homo sapiens*), Hawaiian monk seal (*Neomonachus schauinslandi*), and sequences of K10 of alligator (*A. mississippiensis*), anole (*Anolis carolinensis*), and snake (*Python molurus*) were aligned using the MUSCLE algorithm. Amino acid residues are color-coded. Asterisks, ":", and "." below the alignments indicate identity, high and low conservation, respectively. Residues encoded by the distal portion of exon 7 (Figure 2) are underlined. The opossum has 3 paralogs of *Krt10* [gene IDs: 100015287 (K10), 100015328 (K10L1), 100015374 (K10L2); accession numbers: K10, XP_001369401.2; K10x1, XP_007482311.1; K10L1, XP_007482312.1; K10L2, XP_007482314.1; K10L1x1, XP_016285960.1; K10L2x1, XP_007482313.1]. Other proteins: alligator K10, XP_019355558.1 N-terminus based on extended exon 1; anole K10, XP_016850070.1; snake K10, XP_007440300.1.

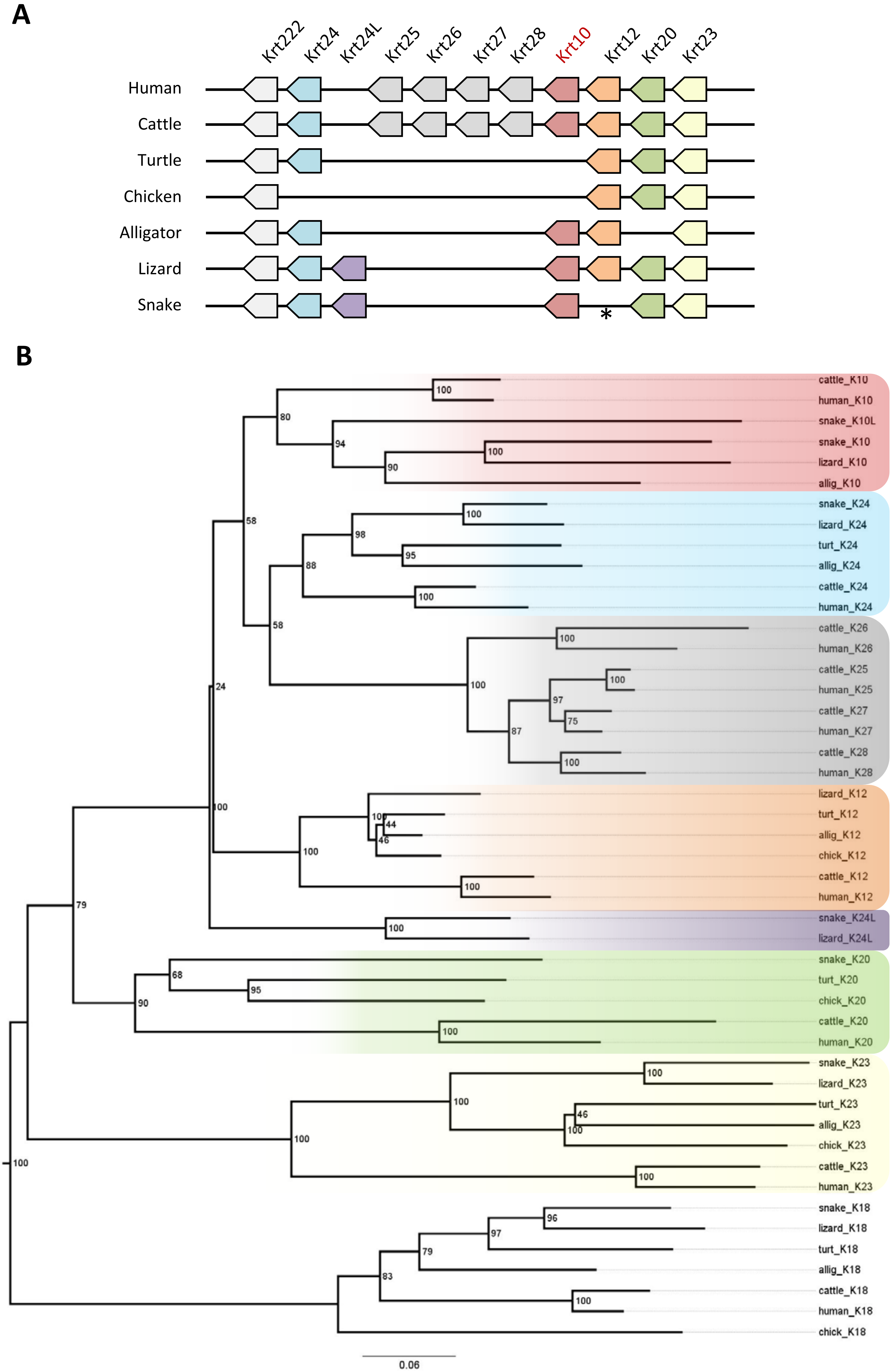


Suppl. Fig. S8 (continued on next page)

**C**

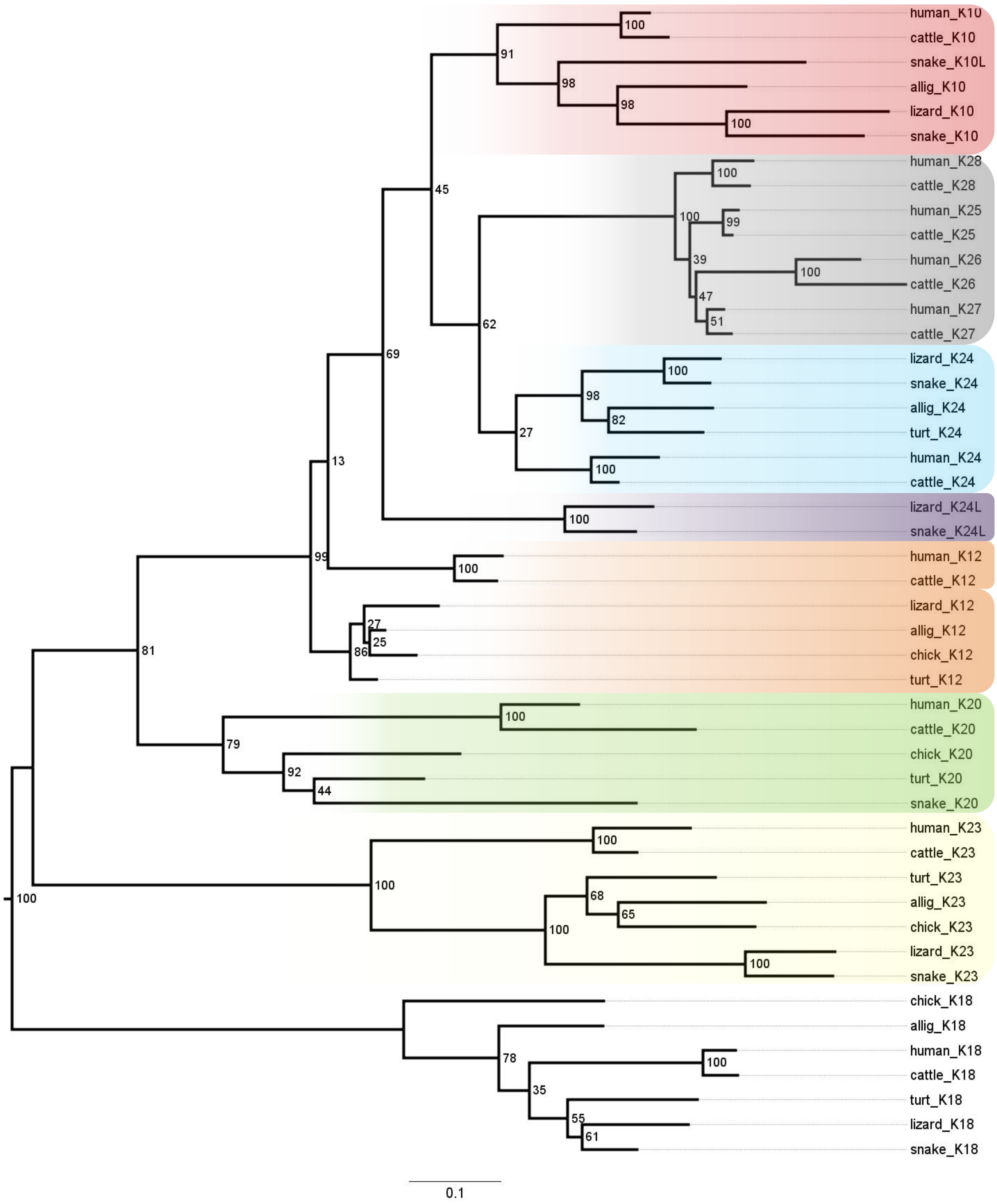


**D**

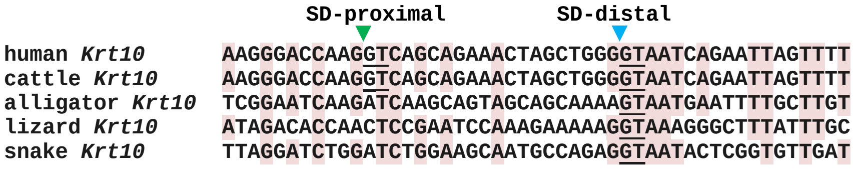


**Suppl. Fig. S8. Phylogenetics of *Krt10* in sauropsids and comparison of exon 7 splice donor sites in sauropsids and mammals.** (**A**) Comparison of the *Krt10* loci in representative mammals and sauropsids (*Chrysemys picta*, *Gallus gallus*, *Alligator mississippiensis*, *Anolis carolinensis*, *Python molurus*). Arrows indicate genes in the direction of transcription. Pseudogenes are not shown. An asterisk indicates that position of pseudogene that was predicted to encode snake K10L (included in phylogenetic analyses) in a previous version of the python genome assembly. (**B**) Neighbour-joining analysis of K10 and other type I keratins. Model: Poisson, bootstrap with 10000 replicates. (**C**) Maximum Likelyhood analysis of K10 and other type I keratins. Model: JTT, bootstrap with 100 replicates. Scale bars in **B** and **C** indicate substitutions per site. (**D**) Nucleotide sequence alignment of the end of exon 7 and beginning of intron 7 of human, cattle and sauropsid *Krt10*. Splice donor (SD) consensus sequences are underlined. Note that the splice of *Krt10* of sauropsids corresponds to the distal splice donor site of mammalian *Krt10* exon 7. Red shading highlights nucleotides that are conserved in mammals and in at least two sauropsids.

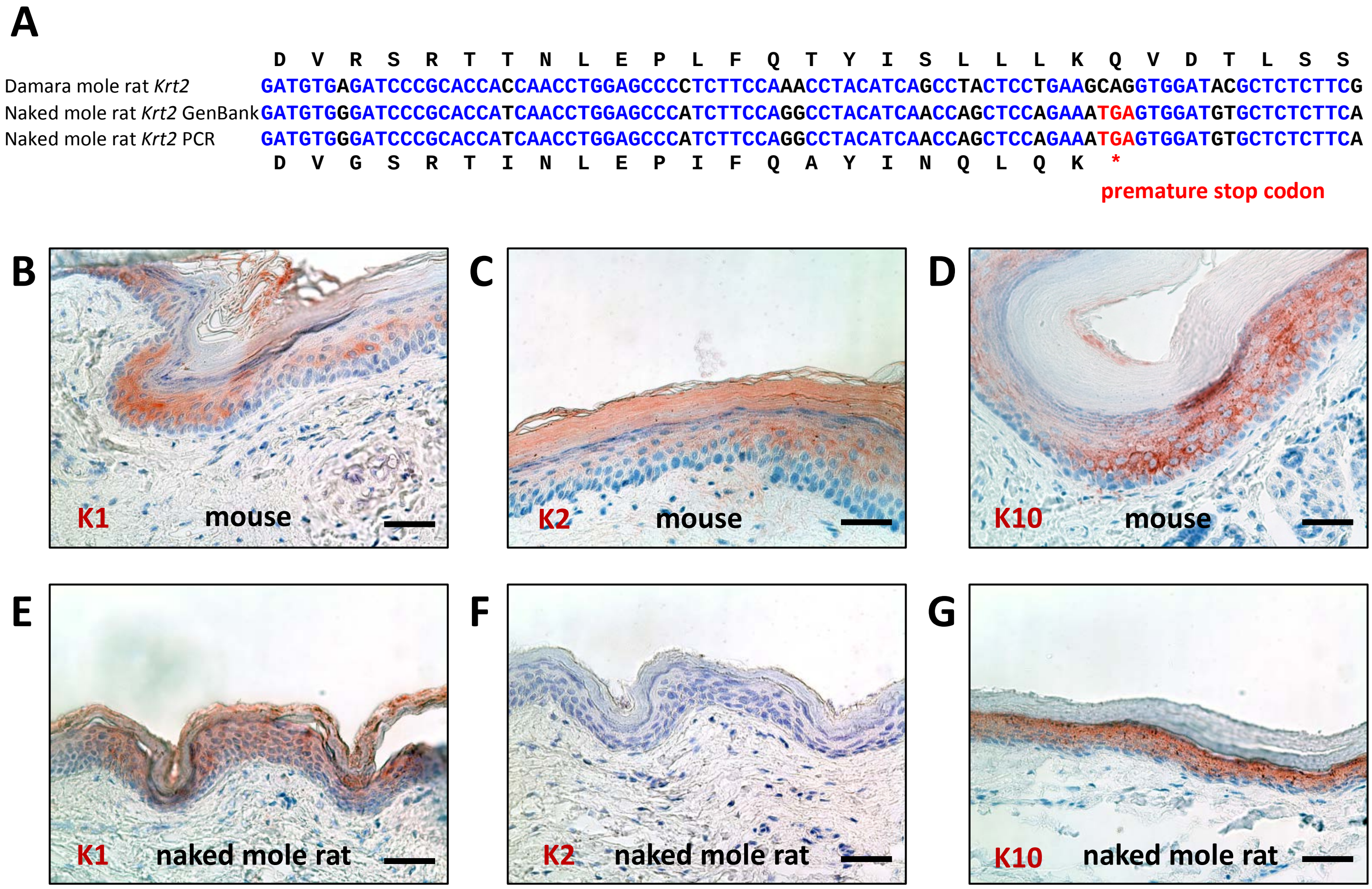


**Suppl. Fig. S9. *Krt2* is inactivated in the naked mole rat.** (**A**) Exon 2 of the *Krt2* gene of the naked mole rat was amplified by PCR and sequenced. The detection of a premature stop codon confirms a stop codon present in the GenBank sequence of the gene. An alignment with the orthologous sequence of the Damara mole rat is shown. (**B**-**G**) Immunohistochechemical detection of keratins K1, K2, K10 in sole skin of the mouse (*Mus musculus*) (**B**-**D**) and the naked mole rat (*Heterocephalus glaber*) (**E**-**G**). Formalin-fixed and paraffin-embedded skin samples from the soles and toes were subjected to immunohistochemical staining (red) with primary antibodies against K1 (**B**, **E**), K2 (**C**, **F**), and K10 (**D**, **G**). K2 was expressed in segments of mouse footpad skin in a manner mutually exclusive with K1, as reported previously (Fischer et al., 2014), whereas it was consistently absent on the entire sole and toe skin of the naked mole rat. Negative control experiments in which the primary antibody was replaced by unrelated antibodies did not yield signals and thereby confirmed the specificity of the stainings. Nuclei were counterstained with hematoxylin. Scale bars, 50 µm.

**Supplementary Table S1. Keratin genes of the minke whale (*Balaenoptera acuturostrata*)**

| Keratin type | Name[1] | Functional protein | Genomic DNA sequence (acc. nr.) | Gene locus[2] start (nucl. nr.) | Gene locus[2] end (nucl. nr.) | Gene name (GenBank) | Protein[3] (GenBank acc. nr.) | Sequence features and comparison between gene predictions[4] |
|---|---|---|---|---|---|---|---|---|
| **type I** | ***Krt222*** | unknown | NW_006726421 | 2229558 | 2238069 | *KRT222* | XP_007177666.1 | exon 4: stop codon, reading frame is intact in the closely related species *B. bonaerensis* (BAUQ01081180.1) |
| **type I** | ***Krt24*** | no | NW_006726421 | 2264357 | 2268176 | *KRT24* | XP_007177665.1 | mutation in exon 1: frameshift at nucl. 262 |
| **type I** | ***Krt12*** | **yes** | NW_006726421 | 2316941 | 2322522 | *KRT12* | XP_007177670.1 | |
| **type I** | ***Krt20*** | no | NW_006726421 | 2329208 | 2338487 | *KRT20* | XP_007177699.1 | exon 1: insertion at nucl. 136, stop at nucl. 290, exon 3: in-frame stops at nucl. 4667 and at nucl. 4775 |
| **type I** | ***Krt23*** | **yes** | NW_006726421 | 2384065 | 2397620 | *KRT23* | XP_007177669.1 | deletion of 12 nucleotides in exon 4 maintains reading frame |
| **type I** | ***Krt40*** | no | NW_006726421 | 2420781 | 2453754 | *KRT40* | XP_007177700.1 | in-frame stop at nucl.18558; sequence gap within gene |
| **type I** | ***Krt33a*** | no | NW_006726421 | 2606237 | 2610664 | *KRT33A* | XP_007177675.1 | in-frame stops at nucl. 2098 and at nucl. 4366 |
| **type I** | ***Krt31*** | **yes** | NW_006726421 | 2619849 | 2623671 | *LOC103015851* | XP_007177676.1 | |
| **type I** | ***Krt32*** | no | NW_006726421 | 2645718 | 2653936 | *KRT32* | XP_007177704.1 | exon 1: in-frame stop at nucl. 469; exon 2: in-frame stop at nucl. 1148; exon 3: in-frame stop at nucl. 1530 |
| **type I** | ***Krt36*** | **yes** | NW_006726421 | 2660034 | 2663887 | *KRT36* | XP_007177677.1 | |
| **type I** | ***Krt13*** | **yes** | NW_006726421 | 2677867 | 2680934 | *KRT13* | XP_007177678.1 | predicted exon 7 (nucl. 4025-4047), predicted exon 8 (nucl. 4381-4451) |
| **type I** | ***Krt15*** | **yes** | NW_006726421 | 2688674 | 2693158 | *KRT15* | XP_007177680.1 | |
| **type I** | ***Krt19*** | **yes** | NW_006726421 | 2698385 | 2702170 | *KRT19* | XP_007177679.1 | *Krt19* has 6 exons, same exon structure in *Bos taurus* |
| **type I** | ***Krt14*** | **yes** | NW_006726421 | 2743586 | 2747826 | *KRT14* | XP_007177705.1 | |
| **type I** | ***Krt16*** | no | NW_006726421 | 2785254 | 2788643 | *KRT16* | XP_007177706.1 | exon 1: insertion at nucl. 55, stop at nucl. 155 |
| **type I** | ***Krt17*** | **yes** | NW_006726421 | 2795495 | 2800750 | *KRT17* | XP_007177681.1 | corrected exon 4 (nucl. 3002-3163); exon 5 corrected by adding sequence from WGS, BAUQ01256725.1 |
| **type I** | ***Krt42*** | no | NW_006726421 | 2804167 | 2809901 | *LOC103017255* | pseudogene | pseudogene |
| **type I** | ***Krt18*** | **yes** | NW_006726687 | 7421032 | 7424720 | *KRT18* | XP_007179472.1 | |
| **type II** | ***Krt80*** | **yes** | NW_006726687 | 6900508 | 6921538 | *KRT80* | XP_007179459.1 | |
| **type II** | ***Krt7*** | **yes** | NW_006726687 | 6961001 | 6977411 | *LOC102998808* | XP_007179461.1 | insertion at nucl. 10382 but no insertion in *Balaenoptera bonaerensis* (acc. nr: BAUQ01711699.1, nucl. 760-580), likely sequence error |
| **type II** | ***Krt81L*** | no | NW_006726687 | 6978177 | 6997558 | *LOC103008741* | XP_007179861.1 | corrected exon 5: nucl. 3279-3432; corrected exon 6: splice site at nucl. 17250 is inactivated |
| **type II** | ***Krt86L*** | **yes** | NW_006726687 | 7006543 | 7013573 | *KRT86* | XP_007179463.1 | |
| **type II** | ***Krt85*** | **yes** | NW_006726687 | 7032482 | 7039998 | *KRT85* | XP_007179462.1 | corrected exon 1: nucl. 1-511 but with sequence gap, not in WGS; corrected last exon: nucl. 6521-6630 |
| **type II** | ***Krt84*** | **yes** | NW_006726687 | 7048765 | 7056362 | *KRT84* | XP_007179466.1 | sequence uncertain, gap in sequence of exon 9 likely due to incorrect assembly, corrected in prediction |
| **type II** | ***Krt75*** | no | NW_006726687 | 7065634 | 7098127 | n.a. | n.a. | in-frame stop at nucl. 18606, deletion at nucl. 18870 |
| **type II** | ***Krt6L1*** | no | NW_006726687 | 7065634 | 7098127 | *KRT6A* | XP_007179862.1 | exon 2: in-frame stop at nucl. 1515, deletion at nucl. 1705 |
| **type II** | ***Krt6L2*** | **yes** | NW_006726687 | 7103555 | 7109483 | *LOC102999913* | XP_007179465.1 | |
| **type II** | ***Krt6L3*** | **yes** | NW_006726687 | 7111138 | 7116146 | *LOC102999639* | XP_007179464.1 | |
| **type II** | ***Krt6L4*** | **yes** | NW_006726687 | 7124027 | 7130702 | *LOC103000457* | XP_007179467.1 | |
| **type II** | ***Krt5*** | **yes** | NW_006726687 | 7153077 | 7160057 | *KRT5* | XP_007179468.1 | splice donor site end of exon 6 is GA in the assembly but a functional GT splice signal is present in the sequence read archive (SRA): SRX316738, run: SRR924087, SRA study: SRP025154 |
| **type II** | ***Krt71*** | no | NW_006726687 | 7184112 | 7193001 | *KRT71* | XP_007198460.1 | exon 1: insertion at nucl. 381 |
| **type II** | ***Krt72*** | no | NW_006726687 | 7214242 | 7221639 | *KRT72* | XP_007198461.1 | exon 1: insertion at nucl. 55, deletion at nucl. 165 ; exon 5:in-frame stop at nucl. 7342 |
| **type II** | ***Krt73*** | no | NW_006726687 | 7252470 | 7261677 | *LOC103009901* | pseudogene | pseudogene |
| **type II** | ***Krt3*** | no | NW_006726687 | 7296585 | 7302440 | *KRT3* | XP_007198446.1 | exon 1: deletion at nucl. 180 leads to frameshift |
| **type II** | ***Krt4*** | **yes** | NW_006726687 | 7315218 | 7320790 | *KRT4* | XP_007179469.1 | partial sequence, last exons are missing |
| **type II** | ***Krt78*** | **yes** | NW_006726687 | 7340120 | 7347804 | *KRT78* | XP_007179470.1 | |
| **type II** | ***Krt8*** | **yes** | NW_006726687 | 7382784 | 7390223 | *KRT8* | XP_007179471.1 | |

Notes: [1] Keratins were named according to orthology to genes in a terrestrial relative (*B. taurus*). The genes are listed in the order of their loci in Figure 1. Genes not encoding a functional protein are included here but not in Figure 1.

[2] "Gene locus" refers to the range of the genome sequence that was investigated, including sequences up- and downstream of the coding region.

[3] Accession numbers (acc. nr.) of protein amino acid sequences predicted in GenBank. These sequences differ from the predictions of the present study (Suppl. Fig. S2), as indicated in the last column of this table.

[4] Nucleotide positions refer to the sequence range indicated under "Gene locus".

Genome sequences used for predictions: BalAcu1.0 and whole genome shotgun (WGS) sequence

n.a., not applicable; nucl., nucleotide

**Supplementary Table S2. Keratin genes of the sperm whale (*Physeter catodon*)**

| Keratin type | Name[1] | Functional protein | Genomic DNA sequence (acc. nr.) | Gene locus[2] start (nucl. nr.) | Gene locus[2] end (nucl. nr.) | Gene name (GenBank) | Protein[3] (GenBank acc. nr.) | Sequence features and comparison between gene predictions[4] |
|---|---|---|---|---|---|---|---|---|
| **type I** | ***Krt222*** | unknown | NW_006724343 | 35391 | 42490 | *KRT222* | XP_007112553.1 | |
| **type I** | ***Krt24*** | no | NW_006724343 | 3846 | 7967 | *KRT24* | XP_007112628.1 | exon 1: insertion at nucl. 259; exon 6: in-frame stop codon at nucl. 3410 |
| **type I** | ***Krt10*** | | | | | not predicted | | |
| **type I** | ***Krt12*** | **yes** | NW_006719488 | 32353 | 37670 | *KRT12* | XP_007124074.1 | |
| **type I** | ***Krt23*** | no | NW_006724343 | 782524 | 790231 | *KRT23* | XP_007131144.1 | exon 4: in-frame stop at nucl. 2573; exon 8: inactivated splice side at nucl. 7637, exon 1 missing |
| **type I** | ***Krt40*** | no | NW_006724343 | 760822 | 766829 | *KRT40* | XP_007130975.1 | exon 4: in-frame stop at nucl. 2486, exon 5: in-frame stop at nucl. 2486 and 2747 |
| **type I** | ***Krt31*** | **yes** | NW_006724343 | 673312 | 677137 | *LOC102992127* | XP_007129142.1 | |
| **type I** | ***Krt32*** | no | NW_006724343 | 650676 | 657927 | *KRT32* | XP_007129153.1 | mutated start codon at nucl. 401, in frame stop at nucl. 423 and nucl. 755 |
| **type I** | ***Krt36*** | no | NW_006724343 | 642933 | 646517 | *KRT36* | XP_007129141.1 | exon 2: in-frame stop at nucl. 1257, exon 6: in-frame stop at nucl. 3565 |
| **type I** | ***Krt13*** | | NW_006724343 | 627260 | 631875 | *KRT13* | XP_007129139.1 | |
| **type I** | ***Krt15*** | **yes** | NW_006724343 | 615240 | 619619 | *KRT15* | XP_007129152.1 | predicted exon 4+5+6 from WGS, AWZP01106219.1, exon 8 from WGS, AWZP01106218.1, exon 7 missing, no inactivating mutations |
| | | | AWZP01106219.1 | 1323 | 630 | n.a. | n.a. | exons 4-6 |
| | | | AWZP01106218.1 | 312 | 404 | n.a. | n.a. | exon 8 |
| **type I** | ***Krt9*** | no | NW_006724343 | 558001 | 615250 | not predicted | n.a. | remnant of exon 1: mutated start codon at nucl. 8850; frameshift mutations |
| **type I** | ***Krt14*** | **yes** | NW_006724343 | 553304 | 557889 | *KRT14* | XP_007129135.1 | |
| **type I** | ***Krt16*** | no | NW_006724343 | 537609 | 546975 | *KRT16* | XP_007129137.1 | corrected exon 1 (1-500), mutations in exon 1: insertions at nucl. 136 and nucl. 380, exon 2: mutated splice donor (predicted at wrong position and in wrong phase), exon 6: in-frame stop at nucl. 2500 |
| **type I** | ***Krt17*** | **yes** | NW_006724343 | 524979 | 530215 | *LOC102991095* | XP_007129138.1 | |
| **type I** | ***Krt42*** | no | NW_006724343 | 515401 | 522934 | *LOC102995741* | XP_007129151.1 | exon 1: deletion at nucl. 90, exon 6: 4 nucleotide deletion at nucl. 6185 |
| **type I** | ***Krt18*** | **yes** | NW_006724343 | 1855855 | 1859392 | *KRT18* | XP_007125856.1 | |
| **type II** | ***Krt80*** | **yes** | NW_006712811 | 150054 | 172743 | *KRT80* | XP_007100975.1 | |
| **type II** | ***Krt7*** | **yes** | NW_006712811 | 96276 | 109330 | *KRT7* | XP_007100972.1 | corrected exon 9: stop at nucl. 12832, encoded protein is shorter than the ortholog of cattle |
| **type II** | ***Krt81L*** | no | NW_006712811 | 87863 | 96149 | *LOC102990944* | pseudogene | LOC102990944 was termed Krt7c (centromere direction) = mutated Krt81 (pseudogene), exon 7 with in frame stop at nucl. 6558 (in IF domain) |
| **type II** | ***Krt86L*** | **yes** | NW_006712811 | 70929 | 78896 | *LOC102991235* | XP_007100973.1 | LOC102991235 was termed Krt7c (telomere direction) = ortholog of Krt81L in orca/dolphin, Krt86L of minke/sperm whale |
| **type II** | ***Krt85*** | **yes** | NW_006712811 | 46932 | 54034 | *KRT85* | XP_007100974.1 | last coding exon contains frameshift and encodes a tail sequence not similar to orthologous proteins |
| **type II** | ***Krt84*** | no | NW_006712811 | 30134 | 36438 | *KRT84* | XP_007130971.1 | exon 2: in-frame stop at nucl. 1878 |
| **type II** | ***Krt6L1*** | no | NW_006712811 | 9246 | 12578 | *LOC102973499* | pseudogene | pseudogene |
| **type II** | ***Krt6L2*** | **yes** | NW_006712811 | 1099 | 4803 | *KRT6A* | XP_007100969.1 | exon 1 is missing, no mutations detectable, considered as intact gene |
| **type II** | ***Krt5*** | **yes** | NW_006724129 | 2061530 | 2067945 | *KRT5* | XP_007125860.1 | |
| **type II** | ***Krt71*** | no | NW_006724129 | 2026032 | 2035975 | *KRT71* | XP_007125869.1 | exon 1: in-frame stop at nucl. 142, exon 2: in-frame stop at nucl. 1146 |
| **type II** | ***Krt3*** | no | NW_006724129 | 1981433 | 2016713 | *KRT3* | XP_007130966.1 | exon 1: insertion at nucl. 483 (frameshift with premature stop at nucl. 505) |
| **type II** | ***Krt4*** | **yes** | NW_006724129 | 1963171 | 1969258 | *KRT4* | XP_007125859.1 | |
| **type II** | ***Krt79*** | no | NW_006724129 | 1949456 | 1962126 | *KRT79* | XP_007125868.1 | exon 2: start at nucl. 3110, in-frame stop at nucl. 3148 |
| **type II** | ***Krt78*** | **yes** | NW_006724129 | 1936293 | 1943953 | *KRT78* | XP_007125858.1 | |
| **type II** | ***Krt8*** | **yes** | NW_006724129 | 1893458 | 1900309 | *KRT8* | XP_007125857.1 | |

Notes: [1] Keratins were named according to orthology to genes in a terrestrial relative (*B. taurus*). The genes are listed in the order of their loci in Figure 1. Genes not encoding a functional protein are included here but not in Figure 1.

[2] "Gene locus" refers to the range of the genome sequence that was investigated, including sequences up- and downstream of the coding region.

[3] Accession numbers (acc. nr.) of protein amino acid sequences predicted in GenBank. These sequences differ from the predictions of the present study (Suppl. Fig. S2), as indicated in the last column of this table.

[4] Nucleotide positions refer to the sequence range indicated under "Gene locus".

Genome sequences used for predictions: Physeter_macrocephalus-2.0.2 (GCF_000472045.1) and whole genome shotgun (WGS) sequence

n.a., not applicable; nucl., nucleotide

**Supplementary Table S3. Keratin genes of the baiji (*Lipotes vexillifer*)**

| Keratin type | Name[1] | Functional protein | Genomic DNA sequence (acc. nr.) | Gene locus[2] start (nucl. nr.) | Gene locus[2] end (nucl. nr.) | Gene name (GenBank) | Protein[3] (GenBank acc. nr.) | Sequence features and comparison between gene predictions[4] |
|---|---|---|---|---|---|---|---|---|
| **type I** | ***Krt222*** | unknown | NW_006791954 | 5038280 | 5045630 | *KRT222* | XP_007465269.1 | |
| **type I** | ***Krt24*** | no | NW_006791954 | 5059416 | 5063674 | *KRT24* | XP_007465270.1 | exon 1: in-frame stop at nucl. 265, exon 3: in-frame stop at nucl. 1938, exon 4: 4-nucleotide deletion at nucl. 2243 (frameshift), exon 8: in-frame stop at nucl. 4163, insertion at nucl. 4180 |
| **type I** | ***Krt12*** | **yes** | NW_006791954 | 5088450 | 5120003 | *KRT12* | XP_007465271.1 | isoform x1 |
| **type I** | ***Krt33a*** | no | NW_006791954 | 5146979 | 5158625 | *LOC103073254* | pseudogene | pseudogene |
| **type I** | ***Krt31*** | **yes** | NW_006791954 | 5169187 | 5173268 | *LOC103087122* | XP_007465276.1 | |
| **type I** | ***Krt32*** | no | NW_006791954 | 5181601 | 5185092 | *KRT32* | XP_007472104.1 | exon 1: in-frame stops at nucl. 67, 313 and 355 |
| **type I** | ***Krt35*** | no | NW_006791954 | 5190245 | 5194156 | *KRT35* | XP_007465300.1 | exon 1: 5-nucleotide insertion at nucl. 239 leads to frameshift |
| **type I** | ***Krt36*** | no | NW_006791954 | 5198489 | 5202028 | *KRT36* | XP_007465301.1 | exon 1: 1 nucleotide deletion at nucl. 100, 1-nucleotide deletion at nucl. 341 |
| **type I** | ***Krt13*** | **yes** | NW_006791954 | 5215654 | 5218898 | *KRT13* | XP_007465277.1 | exon 7 (nucl. 3862-3884), exon 8 (nucl. 4234-4313) |
| **type I** | ***Krt15*** | **yes** | NW_006791954 | 5226796 | 5230941 | *KRT15* | XP_007465278.1 | |
| **type I** | ***Krt19*** | **yes** | NW_006791954 | 5236522 | 5240243 | *KRT19* | XP_007465279.1 | *Krt19* has 6 exons, same exon structure in *Bos taurus* |
| **type I** | ***Krt14*** | **yes** | NW_006791954 | 5278388 | 5282849 | *KRT14* | XP_007465280.1 | corrected exon 4 (nucl. 2768-2986), insertion of 19 codons relative to orthologs |
| **type I** | ***Krt16*** | no | NW_006791954 | 5310774 | 5314001 | *KRT16* | XP_007472116.1 | corrected exon 1 (nucl. 452-942), deletion at nucl. 493, exon 4: in-frame stop at nucl. 2016 |
| **type I** | ***Krt17*** | **yes** | NW_006791954 | 5320445 | 5325112 | *KRT17* | XP_007465281.1 | |
| **type I** | ***Krt42*** | no | NW_006791954 | 5327477 | 5334741 | *LOC103074638* | XP_007465302.1 | exon 1: 1-nucleotide deletion at nucl. 90 |
| **type I** | ***Krt18*** | **yes** | NW_006773303 | 288529 | 292221 | *KRT18* | XP_007448331.1 | |
| **type II** | ***Krt80*** | **yes** | NW_006779087 | 3131797 | 3153539 | *KRT80* | XP_007452403.1 | isoform x1 |
| **type II** | ***Krt7*** | **yes** | NW_006779087 | 3191558 | 3205129 | *KRT7* | XP_007452407.1 | |
| **type II** | ***Krt81L1*** | no | NW_006779087 | 3206039 | 3217957 | *KRT83* | XP_007472113.1 | exon 1: deletion at nucl. 57 |
| **type II** | ***Krt81L2*** | no | NW_006779087 | 3220200 | 3226759 | *KRT86* | XP_007452406.1 | exon 1 in WGS, AUPI01045846.1 (nucl. 867-1237), deletion at nucl. 915, sequence gap in exon 5 (nucl. 9834-9857) |
| **type II** | ***Krt85*** | **yes** | NW_006779087 | 3244290 | 3251135 | *KRT85* | XP_007452405.1 | exon 9: 25-nucleotide insertion lead to shift in reading frame, 13 nucleotide insertion with new stop codon at nucl. 6764, different tail than predicted (compare other whales) |
| **type II** | ***Krt84*** | no | NW_006779087 | 3260477 | 3268844 | *KRT84* | XP_007452422.1 | exon 2: 2-nucleotide deletion at 2296, exon 9: insertion at nucl. 7731 altering C-terminus, corrected exon 1 (nucl. 185-859), corrected exon 4 (nucl. 3456-3551), no exons at nucl. 4976-4983 and 8256-8368 |
| **type II** | ***Krt75*** | no | NW_006779087 | 3277659 | 3313389 | n.a. | n.a. | exon 1: 18451-18947, insertion at nucl. 18579, in-frame stop at nucl. 18692 |
| **type II** | ***Krt6L1*** | no | NW_006779087 | 3277659 | 3313389 | *KRT6C* | XP_007472124.1 | exon 5: new prediction (nucl. 2531-2695), in-frame stop at nucl. 2681, predicted exons 3+4 (nucl. 2318-2710) |
| **type II** | ***Krt6L2*** | **yes** | NW_006779087 | 3319310 | 3324223 | *LOC103087354* | XP_007452423.1 | |
| **type II** | ***Krt6L3*** | **yes** | NW_006779087 | 3332507 | 3337420 | *KRT6A* | XP_007452424.1 | |
| **type II** | ***Krt5*** | **yes** | NW_006779087 | 3359028 | 3365973 | *KRT5* | XP_007452408.1 | isoform x1 |
| **type II** | ***Krt71*** | no | NW_006773303 | 426860 | 435310 | *LOC103082807* | pseudogene | pseudogene |
| **type II** | ***Krt4*** | **yes** | NW_006773303 | 405236 | 411561 | *KRT4* | XP_007448333.1 | |
| **type II** | ***Krt79*** | no | NW_006773303 | 384566 | 396116 | *KRT79* | XP_007472122.1 | corrected exon 1 (nucl. 922-1379), 7-nucleotide deletion at nucl. 1345 leads to frameshift, exon 2: in-frame stop at nucl. 4264, exon 6: 5-nucleotide deletion at nucl. 11201 |
| **type II** | ***Krt78*** | **yes** | NW_006773303 | 371371 | 379647 | *KRT78* | XP_007448332.1 | |
| **type II** | ***Krt8*** | **yes** | NW_006773303 | 291626 | 333312 | *KRT8* | XP_007448330.1 | |

Notes: [1] Keratins were named according to orthology to genes in a terrestrial relative (*B. taurus*). The genes are listed in the order of their loci in Figure 1. Genes not encoding a functional protein are included here but not in Figure 1.

[2] "Gene locus" refers to the range of the genome sequence that was investigated, including sequences up- and downstream of the coding region.

[3] Accession numbers (acc. nr.) of protein amino acid sequences predicted in GenBank. These sequences differ from the predictions of the present study (Suppl. Fig. S2), as indicated in the last column of this table.

[4] Nucleotide positions refer to the sequence range indicated under "Gene locus".

Genome sequences used for predictions: Lipotes_vexillifer_v1 (GCF_000442215.1) and whole genome shotgun (WGS) sequence

n.a., not applicable; nucl., nucleotide

**Supplementary Table S4. Keratin genes of the orca (*Orcinus orca*)**

| Keratin type | Name[1] | Functional protein | Genomic DNA sequence | Gene locus[2] | | Gene name | Protein[3] | Sequence features and comparison between gene predictions[4] |
|---|---|---|---|---|---|---|---|---|
| | | | (acc. nr.) | start (nucl. nr.) | end (nucl. nr.) | (GenBank) | (GenBank acc. nr.) | |
| **type I** | ***Krt222*** | unknown | NW_004438583 | 5115718 | 5123092 | *KRT222* | XP_004282823.1 | |
| **type I** | ***Krt24*** | no | NW_004438583 | 5138102 | 5142152 | *KRT24* | XP_004286808.1 | exon 1: insertion at nucl. 73 leading to in-frame stop at nucl. 178, deletion at nucl. 260; in-frame stop at nucl. 3955 |
| **type I** | ***Krt26*** | no | NW_004438583 | 5142501 | 5162794 | *LOC101274796* | XP_004282917.1 | incomplete, only 4 exons present |
| **type I** | ***Krt12*** | **yes** | NW_004438583 | 5166866 | 5189900 | *LOC101272223* | XP_004282824.1 | isoform x1 |
| **type I** | ***Krt33a*** | no | NW_004438583 | 5216627 | 5227577 | *LOC101275302* | pseudogene | pseudogene |
| **type I** | ***Krt31*** | **yes** | NW_004438583 | 5239237 | 5243298 | *LOC101272480* | XP_004282825.1 | |
| **type I** | ***Krt38*** | no | NW_004438583 | 5251128 | 5256610 | *LOC101275558* | XP_004282918.1 | mutated start codon at nucl. 164, 2-nucleotide insertion at nucl. 194, 61-nucleotide insertion at nucl. 221, 1-nucleotide deletion at nucl. 370 |
| **type I** | ***Krt32*** | no | NW_004438583 | 5259339 | 5264367 | *KRT32* | XP_004282919.1 | mutated start codon at nucl. 70, 59-nucleotide insertion compared to cattle at nucl. 160-218, in-frame stop at nucl. 426 |
| **type I** | ***Krt35*** | no | NW_004438583 | 5270001 | 5274560 | *KRT35* | XP_004282827.1 | mutated start codon at nucl. 706; 15-nucleotide deletion at nucl. 906, 5-nucleotide insertion at nucl. 944, exon 5: 1-nucleotide deletion at nucl. 3000, all compared to cattle |
| **type I** | ***Krt36*** | **yes** | NW_004438583 | 5277598 | 5281146 | *KRT36* | XP_004282826.1 | |
| **type I** | ***Krt13*** | **yes** | NW_004438583 | 5294553 | 5297810 | *LOC101273234* | XP_004282828.1 | exons 7+8 in WGS: ANOL02065426.1 (nucl. 47314-47292 and 46944-46883, complementary strand) |
| | | | ANOL02065426.1 | 47314 | 46883 | n.a. | n.a. | exon 7+8 |
| **type I** | ***Krt15*** | **yes** | NW_004438583 | 5305631 | 5309753 | *LOC101273493* | XP_004282829.1 | |
| **type I** | ***Krt19*** | **yes** | NW_004438583 | 5314994 | 5318744 | *KRT19* | XP_004282830.1 | *Krt19* has 6 exons, same exon structure in *Bos taurus* |
| **type I** | ***Krt14*** | **yes** | NW_004438583 | 5356506 | 5361501 | *LOC101276052* | XP_004282920.2 | corrected exon 1: starts at nucl. 621 (instead of nucl. 588) |
| **type I** | ***Krt16*** | no | NW_004438583 | 5375364 | 5400895 | *LOC101276301* | XP_004286807.1 | exon 1 prediction was incorrect (nucl. 1-202 and nucl. 4726-5251), mutated start codon at nucl. 4761, 1-nucleotide deletion at nucl. 4804, in-frame stop at nucl. 5168-5170, corrected exon 2 (nucl. 5728-5864) with 36-nucleotide insertion, exon 3: 1-nucleotide deletion at nucl. 6062, all compared to cattle |
| **type I** | ***Krt17*** | | NW_004438583 | 5402958 | 5408338 | *KRT17* | XP_004282831.1 | |
| **type I** | ***Krt42*** | no | NW_004438583 | 5411785 | 5417547 | *LOC101274387* | XP_012393201.1 | corrected exon 1: conserved start at nucl. 1252, 1-nucleotide deletion at nucl. 1341 with in-frame stop at nucl. 1546 |
| **type I** | ***Krt18*** | **yes** | NW_004438475 | 2662622 | 2666324 | *KRT18* | XP_004274332.1 | |
| **type II** | ***Krt80*** | **yes** | NW_004438475 | 3053004 | 3073028 | *KRT80* | XP_004274342.1 | isoform x1 |
| **type II** | ***Krt7*** | **yes** | NW_004438475 | 2999636 | 3012809 | *KRT7* | XP_004274339.1 | |
| **type II** | ***Krt7L*** | no | NW_004438475 | 2993735 | 2998884 | *LOC101269396* | pseudogene | pseudogene |
| **type II** | ***Krt81L*** | **yes** | NW_004438475 | 2985920 | 2991422 | *LOC101272192* | XP_004274340.1 | |
| **type II** | ***Krt85*** | **yes** | NW_004438475 | 2960308 | 2967795 | *KRT85* | XP_004274338.1 | corrected exon 9 (nucl. 6492-6726), 2-nucleotide insertion at nucl. 6531, 35-nucleotide insertion at nucl. 6595, 8-nucleotide deletion at nucl. 6681 - different tail sequence, all compared to cattle |
| **type II** | ***Krt84*** | no | NW_004438475 | 2939297 | 2952374 | *KRT84* | XP_012390031.1 | mutated start codon at nucl. 5035, splice donor at nucl. 7600 mutated to AC, incorrect prediction (nucl. 10760-10796) deleted, corrected exon 9 (nucl. 12331-12437) |
| **type II** | ***Krt75*** | no | NW_004438475 | 2881341 | 2935090 | *LOC101290218* | XP_012390030.1 | prediction LOC101290218 was incorrect, included exons of other keratin genes, frameshift at nucl. 43848 |
| **type II** | ***Krt6L2*** | **yes** | NW_004439276 | 400 | 5234 | *LOC101288809* | XP_004286753.1 | partial gene predicted in LOC101288809 with exons 1, 2 - 7, 8, 9, prediction was corrected by inclusion of exons 3+4+5 at NW_004438475, no inactivating mutations |
| | | | NW_004438475 | 2881341 | 2885000 | n.a. | n.a. | exons 3+4+5 (nucl. 4146-4206,4351-4446 and 4573-4744) |
| **type II** | ***Krt5*** | **yes** | NW_004438475 | 2840568 | 2848207 | *LOC101271277* | XP_004274337.1 | |
| **type II** | ***Krt4*** | **yes** | NW_004438475 | 2771840 | 2780579 | *LOC101270796* | XP_004274335.1 | predicted exons 8 (nucl. 5990-6024) and 9 (nucl. 6133-6314) |
| **type II** | ***Krt79*** | no | NW_004438475 | 2752259 | 2762812 | *KRT79* | XP_004286816.2 | mutated exon 2: 2-nucleotide deletion at nucl. 3336 |
| **type II** | ***Krt78*** | **yes** | NW_004438475 | 2738712 | 2746581 | *KRT78* | XP_004274334.1 | |
| **type II** | ***Krt8*** | **yes** | NW_004438475 | 2665729 | 2704776 | *KRT8* | XP_004274331.1 | |

Notes: [1] Keratins were named according to orthology to genes in a terrestrial relative (*B. taurus*). The genes are listed in the order of their loci in Figure 1. Genes not encoding a functional protein are included here but not in Figure 1.

[2] "Gene locus" refers to the range of the genome sequence that was investigated, including sequences up- and downstream of the coding region.

[3] Accession numbers (acc. nr.) of protein amino acid sequences predicted in GenBank. These sequences differ from the predictions of the present study (Suppl. Fig. S2), as indicated in the last column of this table.

[4] Nucleotide positions refer to the sequence range indicated under "Gene locus".

Genome sequences used for predictions: Oorc_1.1 (GCF_000331955.1) and whole genome shotgun (WGS) sequence

n.a., not applicable; nucl., nucleotide

**Supplementary Table S5. Keratin genes of the bottlenose dolphin (*Tursiops truncatus*)**

| Keratin type | Name[1] | Functional protein | Genomic DNA sequence (acc. nr.) | Gene locus[2] start (nucl. nr.) | end (nucl. nr.) | Gene name (GenBank) | Protein[3] (GenBank acc. nr.) | Sequence features and comparison between gene predictions[4] |
|---|---|---|---|---|---|---|---|---|
| **type I** | ***Krt222*** | unknown | NW_017843467 | 567653 | 575621 | *KRT222* | XP_004326131.2 | |
| **type I** | ***Krt24*** | no | NW_017843467 | 589980 | 595159 | *KRT24* | XP_004332181.2 | exon 1: in-frame stop at nucl. 997, exon 4: 1-nucleotide deletion at nucl. 2969, 6-nucleotide at nucl. 2989, 4-nucleotide deletion at nucl. 3003, exon 7: 3-nucleotide insertion at nucl. 4523, 14-nucleotide insertion at nucl. 4455, exon 8: in-frame stop at nucl. 4749 |
| **type I** | ***Krt12*** | **yes** | NW_017843467 | 628895 | 667222 | *LOC101324642* | XP_019794941.1 | isoform x1 is intact |
| **type I** | ***Krt33a*** | no | NW_017843467 | 675916 | 677851 | *LOC101337846* | XP_019794939.1 | only 1 exon predicted |
| **type I** | ***Krt31*** | **yes** | ABRN02229438.1 | 1 | 10462 | n.a. | n.a. | WGS scaffold: ABRN02229438.1, predictions: exon 1 (nucl. 3032-3373), exon 2 (nucl. 3578-3600), exon 3 (nucl. 3997-4153), exon 4 (nucl. 5174-5345), exon 5 (5440-5566), exon 6 (nucl. 5808-6028), exon 7 (nucl. 6783-6836) |
| **type I** | ***Krt38*** | no | NW_017843467 | 678571 | 688007 | *LOC109550599* | XP_019794944.1 | exon 1: in-frame stops at nucl. 1429 and nucl. 1522 |
| **type I** | ***Krt32*** | no | NW_017843467 | 688765 | 696445 | *LOC101329462* | XP_019794943.1 | exon 1: start prediction is wrong (nucl. 1595-2109), mutated start codon at nucl. 1712, 59-nucleotide insertion at nucl. 1798 with in-frame stop at nucl. 1827, exon 4: in-frame stop at nucl. 4472, 1-nucleotide insertion at nucl. 4583 |
| **type I** | ***Krt36*** | **yes** | NW_017843467 | 704975 | 708795 | *LOC101328015* | XP_004323140.1 | |
| **type I** | ***Krt13*** | **yes** | ABRN02449594.1 | 1898 | 222 | n.a. | n.a. | exons 1+2 in WGS, ABRN02449594.1 (nucl. 222-1898, complement), exons 3-6 in WGS, ABRN02449593.1 (nucl. 2720-1683, compl.), exons 7+8 in WGS, ABRN02449593.1, (nucl. 1082-649, compl.) |
| | | | ABRN02449593.1 | 2720 | 649 | n.a. | n.a. | exons 3-8 |
| **type I** | ***Krt15*** | **yes** | ABRN02449595.1 | 2528 | 76 | n.a. | n.a. | exon 1-5 in WGS, ABRN02449595.1 (nucl. 2528-76, compl.), other exons not present on scaffold, no inactivating mutations |
| **type I** | ***Krt19*** | **yes** | ABRN02449596.1 | 8622 | 4622 | n.a. | n.a. | intact gene in WGS, ABRN02449596.1 (nucl. 8622-4622) and in previous genome sequence assembly: GeneID:101328882 |
| **type I** | ***Krt14*** | **yes** | NW_017843467 | 751341 | 772519 | *LOC101329169* | XP_019794945.1 | partly in Krt17 prediction; exon 1+2 in WGS, ABRN02449602.1, corrected exon 3 (in Krt17), nucl. 27231-27317, correct exon 4: nucl. 27910-28066 |
| | | | ABRN02449602.1 | 2634 | 2137 | n.a. | n.a. | exon 1+2 |
| **type I** | ***Krt17*** | **yes** | NW_017843467 | 751341 | 781189 | *LOC101322615* | XP_019794865.1 | incompletely present in available genome sequence, exon 3 corrected: nucl. 128-284, no inactivating mutations in exons identified, expression confirmed by skin transcriptome (SRA) |
| **type I** | ***Krt18*** | **yes** | ABRN02218059.1 | 8051 | 4515 | n.a. | n.a. | gene intact in WGS ABRN02218059.1 (nucl. 8051-4515, compl.) |
| **type II** | ***Krt80*** | **yes** | NW_017844103 | 822625 | 839513 | *KRT80* | XP_019802724.1 | exon 3+4 was incorrect (nucl. 6897-7331), corrected exon 3: nucl. 6897-6957 and exon 4: nucl. 7236-7331 |
| **type II** | ***Krt7*** | **yes** | NW_017844103 | 853741 | 866191 | *LOC101322677* | XP_019802713.1 | deleted one wrong prediction (nucl. 9686-9781), exon 4: WGS, ABRN02539991.1, exon 6: WGS, MRVK01001060.1 |
| | | | ABRN02539991.1 | 15100 | 15002 | n.a. | n.a. | exon 4 |
| | | | MRVK01001060.1 | 73791189 | 73791106 | n.a. | n.a. | exon 6 |
| **type II** | ***Krt81L1*** | no | NW_017844103 | 874129 | 875834 | *LOC101323852* | XP_019802727.1 | only 4 exons predicted |
| **type II** | ***Krt81L2*** | **yes** | ABRN02539990 | 1 | 8110 | n.a. | n.a. | intact gene in WGS, ABRN02539990 (nucl. 1468-6510) |
| **type II** | ***Krt86L*** | no | NW_017844103 | 877731 | 884804 | *LOC101324147* | XP_019802707.1 | in-frame stop at nucl. 3327 |
| **type II** | ***Krt85*** | no | NW_017844103 | 885001 | 893051 | *LOC109547101* | XP_019802728.1 | only exon 1 present, rest not in WGS |
| **type II** | ***Krt84*** | no | NW_017844103 | 894886 | 911316 | *KRT84* | XP_019802708.1 | exon 1 wrong: predictions (nucl. 1-89, 319-454, 8841-9488), mutated start at nucl. 8952 with shift in reading frame, possible exon 1 (nucl. 9035-9488), splice acceptor site of exon 3 inactivated, mutated exon 9 |
| **type II** | ***Krt82*** | no | NW_017844103 | 908351 | 918148 | *LOC109552089* | XP_019802730.1 | corrected exon 1 (8841-9488) - shortened, compare orca, splice donor at exon 3 inactivated, mutated splice site after exon 6 at nucl. 14219, in-frame stop at nucl. 14224 |
| **type II** | ***Krt75*** | no | NW_017844103 | 918812 | 926310 | *LOC101323265* | XP_019802729.1 | exon 1: 2-nucleotide insertion at nucl. 200, leading to shift in reading frame, 2-nucleotide deletion at nucl. 456; exon 6: 1-nucleotide deletion at nucl. 5608 leading to in-frame stop at nucl. 5617 |
| **type II** | ***Krt6L1*** | no | NW_017844103 | 941465 | 943477 | *LOC101322971* | XP_019802726.1 | only 2 exons present, in-frame stop at nucl. 1951 |
| | | | MRVK01000453.1 | 943422 | 942880 | n.a. | n.a. | exon 1 |
| **type II** | ***Krt6L2*** | **yes** | NW_017844103 | 869774 | 968370 | *LOC101321922* | XP_019802723.1 | LOC101321922 prediction was incorrect; exon 1 in WGS: ABRN02539984.1 |
| | | | ABRN02539984.1 | 8446 | 9015 | n.a. | n.a. | exon 1 |
| **type II** | ***Krt5*** | **yes** | NW_017844103 | 978519 | 1026446 | *KRT5* | XP_019802767.1 | predicted exon 1 (nucl. 16-474) is exon 1 of *Krt4*; correct exon 1 in WGS, ABRN02185542. |
| | | | ABRN02185542.1 | 21319 | 21882 | n.a. | n.a. | exon 1 |
| **type II** | ***Krt4*** | **yes** | ABRN02218069.1 | 1 | 33361 | n.a. | n.a. | exon 1 in NW_017844103, nucl. 16-474), exons 1-7 in WGS, ABRN02218069.1 (nucl. 17559-23566), exon 8+9 not in WGS |
| **type II** | ***Krt79*** | no | NW_017844103 | 1044151 | 1045770 | *LOC109552092* | XP_019802770.1 | only exon 6 and 7 present |
| **type II** | ***Krt78*** | **yes** | NW_017844103 | 1025465 | 1076458 | *LOC101323332* | XP_019802768.1 | partly in *Krt8* prediction (nucl. 27853-31998) |
| **type II** | ***Krt8*** | **yes** | NW_017844103 | 1025465 | 1076458 | *LOC101323922* | XP_019802769.1 | exon 1 is predicted too short (nucl. 16-360) in LOC101323922, exon 2-3 correct until sequence gap at nucl. 3613, exon 7 (incomplete) right after gap at nucl. 3715, all exons intact in WGS, ABRN02218060.1 |
| | | | ABRN02218060.1 | 41722 | 48560 | n.a. | n.a. | complete *Krt8* gene |

Notes: [1] Keratins were named according to orthology to genes in a terrestrial relative (*B. taurus*). The genes are listed in the order of their loci in Figure 1. Genes not encoding a functional protein are included here but not in Figure 1.

[2] "Gene locus" refers to the range of the genome sequence that was investigated, including sequences up- and downstream of the coding region.

[3] Accession numbers (acc. nr.) of protein amino acid sequences predicted in GenBank. These sequences differ from the predictions of the present study (Suppl. Fig. S2), as indicated in the last column of this table.

[4] Nucleotide positions refer to the sequence range indicated under "Gene locus".

Genome sequences used for predictions: NIST Tur_tru v1 (GCF_001922835.1) and whole genome shotgun (WGS) sequence

n.a., not applicable; nucl., nucleotide

**Supplementary Table S6. Keratin genes of the manatee (*Trichechus manatus latirostris*)**

| Keratin type | Name[1] | Functional protein | Genomic DNA sequence (acc. nr.) | Gene locus[2] start (nucl. nr.) | Gene locus[2] end (nucl. nr.) | Gene name (GenBank) | Protein[3] (GenBank acc. nr.) | Sequence features and comparison between gene predictions[4] |
|---|---|---|---|---|---|---|---|---|
| **type I** | ***Krt222*** | unknown | NW_004443990 | 16878754 | 16885992 | *LOC101343256* | XP_004378137.1 | |
| **type I** | ***Krt24*** | **yes** | NW_004443990 | 16928165 | 16932207 | *LOC101343511* | XP_004378138.1 | |
| **type I** | ***Krt23*** | no | NW_004443990 | 16944209 | 16945094 | *LOC101357562* | XP_004378185.1 | |
| **type I** | ***Krt25*** | **yes** | NW_004443990 | 16977393 | 16983737 | *LOC101343779* | XP_004378139.1 | |
| **type I** | ***Krt26*** | **yes** | NW_004443990 | 16991758 | 16996817 | *LOC101344036* | XP_004378140.2 | |
| **type I** | ***Krt27*** | **yes** | NW_004443990 | 17001126 | 17006251 | *LOC101344301* | XP_004378141.1 | |
| **type I** | ***Krt28*** | no | NW_004443990 | 17016902 | 17026775 | *LOC101344543* | XP_004378142.1 | exon 1: 35-nucleotide insertion at nucl. 102 leads to frame shift and in-frame stop at nucl. 283 |
| **type I** | ***Krt10*** | no | NW_004443990 | 17047489 | 17050754 | *LOC101357804* | XP_004378186.1 | exon 7 is not conserved (nucl. 3056-3245), splice site predictions were corrected, splice donor site mutated to AT at nucl. 3120, mutated splice acceptor for shorter K10 protein variant at nucl. 3236, 9 nucleotide deletion for longer variant K10x1, exon 8; nucl. 2578-3615 |
| **type I** | ***Krt12*** | **yes** | NW_004443990 | 17103586 | 17108966 | *LOC101344788* | XP_004378143.1 | |
| **type I** | ***Krt20*** | **yes** | NW_004443990 | 17115698 | 17125218 | *LOC101358059* | XP_004378187.1 | |
| **type I** | ***Krt23*** | **yes** | NW_004443990 | 17173248 | 17184247 | *LOC101345036* | XP_004378144.1 | |
| **type I** | ***Krt40*** | **yes** | NW_004443990 | 17215144 | 17221607 | *LOC101358314* | XP_012411409.1 | exon 1 in WGS, AHIN01062152.1, nucl. 2-229, complement, predicted first 6 nucl. deleted, exon 2: nucl. 2712-2794 |
| | | | AHIN01062152.1 | 1894 | 1 | n.a. | n.a. | exon 1 |
| **type I** | ***Krt33a*** | **yes** | NW_004444230 | 1448987 | 1453672 | *LOC101350778* | XP_004390390.1 | |
| **type I** | ***Krt33b*** | **yes** | NW_004444230 | 1429044 | 1436586 | *LOC101350527* | XP_004390389.1 | LOC101350527 prediction was incorrect, nucl. 10-29 deleted, corrected exon 1 (nucl. 1185-1532) |
| **type I** | ***Krt34*** | **yes** | NW_004444230 | 1417504 | 1422962 | *LOC101350270* | XP_004390388.1 | |
| **type I** | ***Krt31*** | **yes** | NW_004444230 | 1402249 | 1405705 | *LOC101357437* | XP_004390412.1 | |
| **type I** | ***Krt38*** | **yes** | NW_004444230 | 1389237 | 1394256 | *LOC101357187* | XP_004390411.1 | |
| **type I** | ***Krt37*** | no | NW_004444230 | 1377038 | 1380673 | *LOC101356941* | XP_012415128.1 | exon 5: in-frame stop at nucl. 2401 |
| **type I** | ***Krt32*** | **yes** | NW_004444230 | 1357961 | 1364826 | *LOC101356699* | XP_004390410.1 | |
| **type I** | ***Krt35*** | **yes** | NW_004444230 | 1348076 | 1351763 | *LOC101349771* | XP_004390386.1 | |
| **type I** | ***Krt36*** | **yes** | NW_004444230 | 1338059 | 1341722 | *LOC101350024* | XP_012415127.1 | exon 1 sequence incomplete, also in WGS, AHIN01146251 (nucl. 909-1331), exon 2 and 3 in WGS, AHIN01146255.1 and AHIN01146256.1, no inactivating mutations |
| | | | AHIN01146255.1 | 1776 | 1855 | n.a. | n.a. | exon 2 |
| | | | AHIN01146256.1 | 11324 | 11492 | n.a. | n.a. | exon 3 |
| **type I** | ***Krt13*** | **yes** | NW_004444230 | 1320121 | 1324500 | *LOC105756953* | XP_012415143.1 | corrected exon 6 (nucl. 2596-2716), predicted exon 7 (nucl. 3470-3496), predicted exon 8 (nucl. 3820-3884) |
| **type I** | ***Krt15*** | **yes** | NW_004444230 | 1307047 | 1311347 | *LOC101349511* | XP_004390385.1 | |
| **type I** | ***Krt19*** | **yes** | NW_004444230 | 1297656 | 1301503 | *LOC101356439* | XP_004390409.1 | |
| **type I** | ***Krt14*** | **yes** | NW_004444230 | 1254197 | 1258394 | *LOC101349261* | XP_004390384.1 | |
| **type I** | ***Krt16*** | **yes** | NW_004444230 | 1234984 | 1237910 | *LOC101349001* | XP_004390383.1 | |
| **type I** | ***Krt17*** | **yes** | NW_004444230 | 1222133 | 1227208 | *LOC101348742* | XP_004390382.1 | |
| **type I** | ***Krt42*** | no | NW_004444230 | 1212358 | 1218624 | *LOC101356185* | pseudogene | |
| **type I** | ***Krt18*** | **yes** | NW_004443963 | 23529999 | 23538203 | *LOC101355511* | XP_004374057.1 | |
| **type II** | ***Krt80*** | **yes** | NW_004444145 | 3656305 | 3679334 | *LOC101347121* | XP_004388413.1 | |
| **type II** | ***Krt7*** | **yes** | NW_004444145 | 3738932 | 3753092 | *LOC101358971* | XP_004388445.1 | |
| **type II** | ***Krt83L*** | **yes** | NW_004444145 | 3754970 | 3762732 | *LOC101347377* | XP_004388414.1 | |
| **type II** | ***Krt88*** | **yes** | NW_004444145 | 3779779 | 3783575 | *LOC101359236* | XP_012414525.1 | |
| **type II** | ***Krt81*** | **yes** | NW_004444145 | 3791007 | 3796711 | *LOC101347627* | XP_004388415.1 | |
| **type II** | ***Krt86*** | **yes** | NW_004444145 | 3805977 | 3812510 | *LOC101347877* | XP_004388416.1 | |
| **type II** | ***Krt83*** | **yes** | NW_004444145 | 3822256 | 3832147 | *LOC101348134* | XP_004388417.1 | |
| **type II** | ***Krt89*** | no | NW_004444145 | 3843897 | 3859575 | *LOC101348390* | XP_012414534.1 | exon 1: 1-nucleotide substitution at nucl. 326 leading to in-frame stop at nucl. 358 and nucl. 382 |
| **type II** | ***Krt85*** | **yes** | NW_004444145 | 3866076 | 3873487 | *LOC101348645* | XP_004388418.1 | |
| **type II** | ***Krt84*** | **yes** | NW_004444145 | 3890414 | 3898534 | *LOC101348904* | XP_004388419.1 | |
| **type II** | ***Krt82*** | **yes** | NW_004444145 | 3907100 | 3918838 | *LOC101349158* | XP_004388420.1 | |
| **type II** | ***Krt124p*** | no | NW_004444145 | 3923679 | 3936095 | *LOC101359503* | XP_012414528.1 | exon 1: in-frame stop at nucl. 10, according to position it is considered as human KRT124P ortholog |
| **type II** | ***Krt75*** | no | NW_004444145 | 3938549 | 3948914 | *LOC101349414* | XP_012414527.1 | exon 1: in-frame stop at nucl. 376 and nucl. 460 |
| **type II** | ***Krt6*** | **yes** | NW_004444145 | 3961324 | 4036587 | *LOC101349666* | XP_004388421.1 | sequence gaps in introns |
| **type II** | ***Krt5*** | **yes** | NW_004444145 | 4056067 | 4062551 | *LOC101349917* | XP_004388422.1 | |
| **type II** | ***Krt71*** | **yes** | NW_004444145 | 4084370 | 4093029 | *LOC101350512* | XP_004388425.1 | |
| **type II** | ***Krt74*** | no | NW_004444145 | 4106882 | 4114243 | *LOC101350763* | XP_004391295.2 | exon 2: in-frame stop at nucl. 1128, exon 4: in-frame stop at nucl. 2279, exon 6: deletion at nucl. 6315 |
| **type II** | ***Krt72*** | no | NW_004444145 | 4132719 | 4149067 | *LOC101359763* | XP_004388447.2 | LOC101359763 prediction contained errors, mutated start codon at nucl. 2541, no in-frame start codon upstream; in-frame stop at nucl. 2937, in-frame stop at nucl. 11973 |
| **type II** | ***Krt73*** | **yes** | NW_004444145 | 4155016 | 4165404 | *LOC101351019* | XP_004388426.1 | |
| **type II** | ***n.a.*** | no | NW_004444145 | 4178703 | 4184845 | *LOC101360019* | XP_012414526.1 | position not orthologous to Krt1, in-frame stops at nucleotides 441, 2422, 3532 and 4449 |
| **type II** | ***Krt2*** | no | NW_004444145 | 4198400 | 4204844 | *LOC101351286* | XP_004391331.1 | exon 1: 1-nucleotide deletion at nucl. 113 leading to in-frame stops at nucl. 133, 213 and 561, exon 5 with in-frame stops at nucl. 3819 and 3867 |
| **type II** | ***Krt1*** | no | NW_00444396 | 23784772 | 23787412 | *LOC105756172* | XP_012410280.1 | corrected exon 1: nucl. 1-522, exon 2: mutated splice donor at nucl. 1553, alternative splice donor at nucl. 1554 shifts reading frame by 1 nucleotide, corrected exon 4: nucl. 2546-2641 |
| **type II** | ***Krt77*** | no | NW_00444396 | 23760779 | 23774593 | *LOC101357457* | XP_004391296.1 | exon 1: 8-nucleotide insertion at nucl. 103-110 leading to in-frame stop at nucl. 220, in-frame stops at nucl. 9501 and 12584 |
| **type II** | ***Krt126*** | no | NW_00444396 | 23735410 | 23744699 | *LOC101358657* | XP_012410279.1 | |
| **type II** | ***Krt127*** | no | NW_00444396 | 23718888 | 23725017 | *LOC101358390* | XP_012410278.1 | in-frame stop at nucl. 3978 |
| **type II** | ***Krt76*** | no | NW_00444396 | 23700001 | 23718815 | *LOC101358134* | XP_004374124.1 | incomplete, predicted first exon is orthologous to exon 6 of type II keratin genes |
| **type II** | ***Krt2L*** | no | NW_00444396 | 23685420 | 23688293 | *LOC101356959* | XP_012410325.1 | exon 1 (nucl. 954-1031) was predicted incorrectly in LOC101356959, exon 4 (nucl. 1577-1741): in-frame stop at nucl. 1685 |
| **type II** | ***Krt3*** | **yes** | NW_00444396 | 23672875 | 23689584 | *LOC101356457* | XP_004374060.2 | corrected exon 8 (nucl. 5451-5722), corrected exon 9 (nucl. 5451-5722), LOC101356457 prediction was incorrect (nucl. 16464-16710), this prediction includes K2-like (LOC101356959) |
| **type II** | ***Krt4*** | **yes** | NW_00444396 | 23652449 | 23658032 | *LOC101356717* | XP_004374061.1 | |
| **type II** | ***Krt79*** | no | NW_00444396 | 23630332 | 23643919 | *LOC101357874* | XP_004374123.1 | exon 1: in-frame stop at nucl. 307 |
| **type II** | ***Krt78*** | **yes** | NW_00444396 | 23616833 | 23623978 | *LOC101356203* | XP_004374059.1 | |
| **type II** | ***Krt8*** | **yes** | NW_00444396 | 23568076 | 23575466 | *LOC101355946* | XP_004374058.1 | |

Notes: [1] Keratins were named according to orthology to genes in a terrestrial relative (*L. africana*). The genes are listed in the order of their loci in Figure 1. Genes not encoding a functional protein are included here but not in Figure 1.

[2] "Gene locus" refers to the range of the genome sequence that was investigated, including sequences up- and downstream of the coding region.

[3] Accession numbers (acc. nr.) of protein amino acid sequences predicted in GenBank. These sequences differ from the predictions of the present study (Suppl. Fig. S2), as indicated in the last column of this table.

[4] Nucleotide positions refer to the sequence range indicated under "Gene locus".

Genome sequences used for predictions: TriManLat1.0 (GCF_000243295.1) and whole genome shotgun (WGS) sequence

n.a., not applicable; nucl., nucleotide

**Supplementary Table S7. Keratin genes of mammalian species included in Figure 5**

| Species | Genome sequence assembly | Name[1] | Functional protein | Gene name (GenBank) | Genomic DNA sequence (acc. nr) | Gene locus[2] start (nucl. nr) | Gene locus[2] end (nucl. nr) | Protein[3] (acc. nr) | Sequence features and comparison between gene predictions[4] |
|---|---|---|---|---|---|---|---|---|---|
| ***Nannospalax galili*** | S.galili_v1.0 (GCF_000622305.1) | *Krt1* | **yes** | *LOC103739561* | NW_008343671 | 230000 | 235402 | XP_008838189.1 | |
| | | *Krt2* | no | *Krt2* | NW_008343671 | 261583 | 270417 | XP_008838193.1 | 133-nucleotide insertion at nucl. 52 leading to frameshift |
| | | *Krt9* | no | *Krt9* | NW_008340203 | 1259760 | 1265432 | XP_008833593.1 | 5-nucleotide insertion at nucl. 357, 1-nucleotide insertion at nucl. 495; premature stop at nucl. 1683 |
| | | *Krt10* | **yes** | *Krt10* | NW_008340203 | 324725 | 329464 | XP_008833412.1 | |
| | | *Krt23* | **yes** | *Krt23* | NW_008340203 | 416647 | 433862 | XP_008833416.1 | |
| | | *Krt77* | **yes** | *Krt77* | NW_008343671 | 208595 | 220699 | XP_017655346.1 | isoform x1 |
| ***Fukomys damarensis*** | DMR_v1.0 (GCF_000743615.1) | *Krt1* | **yes** | *LOC104851891* | NW_011047010 | 170253 | 175462 | XP_010607566.1 | |
| | | *Krt2* | **yes** | *Krt2* | NW_011047010 | 123342 | 131078 | XP_010607562.1 | isoform x1 with 9 exons intact |
| | | *Krt9* | no | n.a. | NW_011045366 | 225000 | 253500 | n.a. | only small remnant of exon 6 identified at nucl. 8203-8232 |
| | | *Krt10* | **yes** | *Krt10* | NW_011047935 | 249749 | 255597 | XP_010613357.1 | corrected exon 6: nucl. 2708-2925; predicted exon 7, partial, begins after sequence gap (<4994) and ends at nucl. 5165; predicted exon 8: nucl. 5472-5576 |
| | | *Krt23* | **yes** | *Krt23* | NW_011047935 | 299272 | 311602 | XP_010613358.1 | |
| | | *Krt77* | **yes** | *Krt77* | NW_011047010 | 180754 | 195032 | XP_010607703.1 | corrected exon 9: nucl. 9916->10054 (followed by sequence gap), also WGS sequence (AYUG01147347.1) |
| ***Heterocephalus glaber*** | HetGla_female_1.0 (GCF_000247695.1) | *Krt1* | **yes** | *Krt1* | XP_004872754.1 | 801999 | 807480 | XP_004872754.1 | |
| | | *Krt2* | no | *Krt2* | NW_004624904 | 761073 | 768153 | XP_004872753.1 | premature stop in exon 2 at nucl. 1628-1630 |
| | | *Krt9* | no | n.a. | NW_004624795 | 2062000 | 2085000 | n.a. | exon 1 remnant at nucl. 4595-5118 with several inactivating mutations |
| | | *Krt10* | **yes** | *Krt10* | NW_004624795 | 2594973 | 2599433 | XP_004859479.1 | |
| | | *Krt23* | **yes** | *Krt23* | NW_004624795 | 2530613 | 2543398 | XP_004859476.1 | isoform x1 |
| | | *Krt77* | **yes** | *LOC101726785* | NW_004624904 | 816622 | 826988 | XP_021106281.1 | corrected exon 1: nucl. 1-558 |
| ***Chrysochloris asiatica*** | OryAfe1.0 (GCF_000298275.1) | *Krt1* | **yes** | *KRT1* | NW_006408656 | 3531415 | 3536667 | XP_006870190.1 | |
| | | *Krt2* | **yes** | *KRT2* | NW_006408656 | 3569007 | 3575929 | XP_006870058.1 | |
| | | *Krt9* | no | *KRT9* | NW_006408525 | 27283499 | 27287719 | XP_006878247.1 | exon 1: 8-nucleotide insertion at nucl. 18; 7-nucleotide insertion at nucl. 55-61, and others; exon 3: 1-nucleotide insertion at nucl. 1650, 4-nucleotide insertion at nucl. 1719, corrected exon 3: nucl. 1571-1732 |
| | | *Krt10* | **yes** | *KRT10* | NW_006408525 | 26088981 | 26092907 | XP_006833735.1 | |
| | | *Krt23* | **yes** | *KRT23* | NW_006408525 | 26203225 | 26218644 | XP_006832663.1 | |
| | | *Krt77* | **yes** | *KRT77* | NW_006408656 | 3491654 | 3506435 | XP_006870057.1 | |
| ***Sarcophilus harrisii*** | Devil_ref v7.0 (GCF_000189315.1) | *Krt1* | **yes** | *LOC100917101* | NW_003843731 | 1804049 | 1814465 | XP_003772276.1 | |
| | | *Krt2* | **yes** | *LOC100933339* | NW_003843731 | 1837640 | 1848004 | XP_003772255.1 | |
| | | *Krt9* | **yes** | *KRT9* | NW_003838836 | 8588 | 14590 | XP_023358108.1 | |
| | | *Krt10* | **yes** | *LOC105750380* | NW_003838837 | 385688 | 391231 | XP_012403757.1 | incomplete genome sequence, exon 1 ends in sequence gap, incorrectly predicted exon (nucl. 1274-1322) was deleted, no inactivating mutations, both splice forms intact |
| | | *Krt23* | **yes** | *KRT23* | NW_003838837 | 220315 | 241571 | XP_012403837.1 | |
| | | *Krt77* | **yes** | *LOC100930808* | NW_003844111 | 13606 | 25427 | XP_003772681.1 | |

Notes: [1] Only suprabasal epidermal keratin genes *Krt1, Krt2, Krt9, Krt23,* and *Krt77* are listed here. Other keratin genes of Figure 5 are conserved and available in GenBank.

[2] "Gene locus" refers to the range of the genome sequence that was investigated, including sequences up- and downstream of the coding region.

[3] Accession numbers (acc. nr.) of protein amino acid sequences predicted in GenBank. Differences between these sequences predictions used in the present study are indicated in the last column of this table.

[4] Nucleotide positions refer to the sequence range indicated under "Gene locus".

As a representative of marsupials, the tasmanian devil (*S. harrisii*) is included. In the opossum, the organization of the *Krt1-Krt2-Krt77* locus is uncertain and information about Krt10 is provided in Suppl. Fig. S7.

n.a., not applicable; nucl., nucleotide; WGS, whole genome shotgun sequence
